# Anticarcinogenic effects of ursodeoxycholic acid in pancreatic adenocarcinoma cell models

**DOI:** 10.3389/fcell.2024.1487685

**Published:** 2024-12-11

**Authors:** Patrik Kovács, Szandra Schwarcz, Petra Nyerges, Tímea Ingrid Bíró, Gyula Ujlaki, Péter Bai, Edit Mikó

**Affiliations:** ^1^ Department of Medical Chemistry, Faculty of Medicine, University of Debrecen, Debrecen, Hungary; ^2^ MTA-DE Lendület Laboratory of Cellular Metabolism, University of Debrecen, Debrecen, Hungary; ^3^ HUN-REN-UD Cell Biology and Signaling Research Group, Debrecen, Hungary; ^4^ Research Center for Molecular Medicine, Faculty of Medicine, University of Debrecen, Debrecen, Hungary

**Keywords:** ursodeoxycholic acid, bile acid, epithelial mesenchymal transition, oxidative stress, cancer stem cell, oxidative phosphorylation

## Abstract

Changes to the composition of the microbiome in neoplasia, is termed oncobiosis, may affect tumor behavior through the changes to the secretion of bacterial metabolites. In this study we show, that ursodeoxycholic acid (UDCA), a bacterial metabolite, has cytostatic properties in pancreatic adenocarcinoma cell (PDAC) models. UDCA in concentrations corresponding to the human serum reference range suppressed PDAC cell proliferation. UDCA inhibited the expression of epithelial mesenchymal transition (EMT)-related markers and invasion capacity of PDAC cells. UDCA treatment increased oxidative/nitrosative stress by reducing the expression of nuclear factor, erythroid 2-like 2 (NRF2), inducing inducible nitric oxide synthase (iNOS) and nitrotyrosine levels and enhancing lipid peroxidation. Furthermore, UDCA reduced the expression of cancer stem cell markers and the proportion of cancer stem cells. Suppression of oxidative stress by antioxidants, blunted the UDCA-induced reduction in cancer stemness. Finally, we showed that UDCA induced mitochondrial oxidative metabolism. UDCA did not modulate the effectiveness of chemotherapy agents used in the chemotherapy treatment of pancreatic adenocarcinoma. The antineoplastic effects of UDCA, observed here, may contribute to the induction of cytostasis in PDAC cell models by providing a more oxidative/nitrosative environment.

## 1 Introduction

Pancreatic adenocarcinoma (PDAC) is one of the most malignant neoplastic disease due to its invasiveness, frequent metastasis and recurrence. Symptoms may not appear until the cancer is advanced. Due to the development of distant metastases and lymphatic infiltrates, the 5-year survival rate is less than 5% (Ducreux et al., 2015). Treatment options for pancreatic adenocarcinoma include surgery, chemotherapy, radiotherapy and targeted therapy depending on the stage and characteristics of the cancer (Chun et al., 2018).

Neoplastic diseases, as PDAC, are characterized by changes to multiple microbiome compartments (Kiss et al., 2020), including the gut microbiome (Aykut et al., 2019; Kartal et al., 2022; Sharma et al., 2024) that has the biggest biosynthetic and biotransformation capacity among all microbiome compartment. The transformed microbiome is often referred as the oncobiome (Thomas and Jobin, 2015). The oncobiome has multiple modalities to influence the behavior of tumors, one of which is the production of metabolites or toxins (Mikó et al., 2024) that was evidenced in PDAC [e.g., trimethylamine N-oxide (Mirji et al., 2022)]. This mechanism is similar to hormonal regulation, where the bacteria of the microbiome produce metabolites that are resorbed and enter the circulation to exert their effects on distantly located (tumor) cells through receptorial pathways (Mikó et al., 2024). The same metabolites can act as paracrine regulators if bacteria colonize the tumor (Mikó et al., 2024).

One such metabolite group is the class of bile acids. Primary bile acids are synthesized in the liver and enter the GI through the bile (Režen et al., 2022). Bacteria transform primary bile acids to secondary bile acids, such as ursodeoxycholic acid (UDCA). Subsequently, secondary bile acids are resorbed to the portal circulation and the bulk is taken back up to the liver. A minority of the secondary bile acids remain in the circulation, the total bile acids levels being lower than 5 µM. The reference serum levels of UDCA is around 0.1–1 µM (Wei et al., 2020; James et al., 2021).

Ursodeoxycholic acid (UDCA) is a non-toxic hydrophilic bile acid derived from chenodeoxycholic acid (CDCA). UDCA, as a drug, is approved for the treatment of primary biliary cholangitis and cholesterol gallstone dissolution [summarized in (Cabrera et al., 2019; Režen et al., 2022; Achufusi et al., 2024)]. Numerous studies have reported that UDCA exert antitumor effects in multiple cancers [reviewed in (Režen et al., 2022)].

UDCA was implicated in multiple cancers, mostly as a beneficial compound (Režen et al., 2022). Among these neoplasias, UDCA was connected to PDAC, namely, UDCA was shown to suppress epithelial-to-mesenchymal transition and cancer stem cell formation by reducing ROS level in pancreatic cancer (Kim Y. J. et al., 2017). Furthermore, oxidative stress is an important inducer of pancreatic acinar cell apoptosis (Booth et al., 2011). These observations prompted us to widen the understanding of the role of UDCA in pancreatic adenocarcinoma using cell models.

## 2 Materials and Methods

### 2.1 Chemical compounds

Ursodeoxycholic acid (UDCA, cat #U5127) was purchased from Sigma-Aldrich (St. Louis, MI, United States) and was dissolved in dimethyl sulphoxide (DMSO) to achieve the final concentration of 100 mM. For cell proliferation study, UDCA was used in varied doses for the treatment of Capan-2 PDAC cell model for 24 or 48 h. In subsequent experiments, UDCA was used at a concentration of 0.3 µM, which corresponds to the human serum reference concentration of UDCA (Wei et al., 2020; James et al., 2021). Control cells received 0.001% DMSO in medium as a vehicle. ROS scavangers, MitoTEMPO (cat # SML0737) and pegylated catalase (pegCAT; cat #C4963) were obtained from Sigma-Aldrich. MitoTEMPO was used at a final concentration of 5 µM. PegCAT was used at a concentration of 500 U/mL. Chemotherapy drugs, 5-fluorouracil (5FU, cat #F6627), and oxaliplatin (OXA, cat #O9512) were purchased from Sigma-Aldrich and were dissolved in DMSO at a concentration of 100 mM.

### 2.2 Cell lines

Capan-2 cells were cultured in MEM (Sigma-Aldrich, cat #M8042) medium containing 10% fetal bovine serum (FBS), 2 mM glutamine and 1% penicillin/streptomycin at 37°C with 5% CO_2_. BxPC-3 and PancTu-1 cells were cultured in RPMI 1640 (Sigma-Aldrich; cat #R5886) medium supplemented with 10% FBS, 2 mM glutamine and 1% penicillin/streptomycin at 37°C with 5% CO_2_. The human primary fibroblast cells were maintained in DMEM (Sigma-Aldrich, cat #D5546) containing 20% FBS, 1% penicillin/streptomycin, 2 mM L-glutamine at 37°C with 5% CO_2_. Cell lines were regularly checked for *mycoplasma* contamination.

### 2.3 Sulforhodamine B (SRB) assay

Capan-2 cells were seeded in 96-well plates at a density of 3,000 cells/well in 200 µL of complete medium and incubated overnight. Cells were treated with different concentrations (0.01-1 µM) of UDCA for 24 h and 48 h. Then, cells were fixed in PBS by the addition of trichloroacetic acid (TCA) at a final concentration of 10% for 1 h at 4°C. After fixation, cells were washed with water and stained with SRB solution (0.4% in 1% acetic acid) for 10 min. Unbound dye was removed by washing with 1% acetic acid. Bound stain was solubilized with 10 mM Tris base and the absorbance at 540 nm were determined with a plate reader.

### 2.4 3-(4,5-dimethylthiazol-2-yl)-2,5-diphenyltetrazolium bromide (MTT) assay

Capan-2 cells were seeded in 96-well plates at a density of 3,000 cells/well in 200 µL of complete medium and incubated overnight. Cells were treated with various concentration of chemotherapy drugs and UDCA (0.3 µM) for 48 h. Then, 20 µL of MTT solution (5 mg/mL) was added to each well and incubated for 1.5 h at 37°C. The supernatant was discarded, and 100 µL of DMSO was added to dissolve the precipitate. Absorbance at 540 nm was measured spectrophotometrically. Considerations and calculations are described in (Kacsir et al., 2023; Schwarcz et al., 2024b).

### 2.5 Detection of cell death

To evaluate changes in necrotic and apoptotic cell death, we used an Annexin V/PI double staining assay kit (Thermo Fisher Scientific, cat #V13242). Capan-2 cells were seeded in 6-well plates (150,000 cells/well) and treated with UDCA (0.3 µM) for 48 h. Then, cells were stained with 100 μg/mL PI solution and 5 µL FITC Annexin V for 15 min at room temperature. The numbers of apoptotic and necrotic cells were measured using a FACS Calibur flow cytometer (Beckton Dickinson Franklin Lakes, NJ, United States).

### 2.6 Measurement of superoxide production (DHE staining)

Superoxide production was measured using dihydroethidium (DHE, Sigma Aldrich, cat #D7008) staining. Capan-2 cells were seeded into 6-well plates (100,000 cells/well) and treated with UDCA (0.3 µM) for 48 h. Cells were stained with 2.5 µM DHE for 30 min at 37°C, and fluorescence was analyzed by flow cytometry (FACS Calibur; Becton Dickinson). For data evaluation, BD CellQuest Pro software v5.2 (Becton Dickinson) was used.

### 2.7 Cell invasion

Cell invasion was determined using Corning BioCoat Matrigel Invasion Chambers (Corning, NY, United States) (cat # 354480) described by (Schwarcz et al., 2024a).

### 2.8 Western blotting

Pancreatic adenocarcinoma cells were seeded into 6-well cell culture plates (150.000 cells/well) and treated with UDCA (0.3 µM) and vehicle (DMSO) for 48 h. After 2 days of treatment, protein was extracted using RIPA lysis buffer containing 50 mM Tris pH 7.5, 150 mM NaCl, 0.1% sodium dodecyl sulfate (SDS), 1% Triton X-100, 0.5% sodium deoxycholate, 1 mM EDTA, 1 mM Na_3_VO_4_, 1 mM NaF, 1 mM PMSF and a protease inhibitor cocktail. Protein concentration was determined using a BCA protein assay kit (Pierce Biotechnologies, Rockford, IL, United States). SDS-polyacrylamide gel electrophoresis was performed on 10% gels. Proteins were transferred onto nitrocellulose membrane and blocked for 1 h at room temperature with 5% BSA in Tris-buffered saline containing 0.1% Tween 20 (TBST). Membranes were then incubated with primary antibodies overnight at 4°C. Membranes were washed three times in TBST before being incubated for 1 h incubation at room temperature with HRP-conjugated secondary antibody (Cell Signaling Technology, Inc. Beverly, MA, 1:2,000). Membranes were washed with TBST and proteins were visualized using SuperSignal West Pico Solutions (Thermo Fisher Scientific) on ChemiDoc Imaging System (BioRad, Hercules, CA, United States) Blots were quantified by densitometry using Image Lab 6.1 software. Primary antibodies used in this study are listed in Table 1.

**TABLE 1:** Primary antibodies used in Western blot analyses.

Antibody symbol	Vendor	Dilution
β-catenin	Cell Signaling Technology (8,480)	1:1,000
Snail	Cell Signaling Technology (3,879)	1:1,000
Slug	Cell Signaling Technology (C19G7)	1:1,000
Vimentin	Cell Signaling Technology (5,741)	1:1,000
Claudin-1	Cell Signaling Technology (13,255)	1:1,000
E-cadherin	Cell Signaling Technology (3,195)	1:1,000
ZO1	Cell Signaling Technology (8,193)	1:1,000
NRF2	Abcam (ab31163)	1:1,000
iNOS	Novus (NB300-605)	1:1,000
4HNE	Abcam (ab46545)	1:1,000
Nitrotyrosine	Thermo Fisher Scientific (A21285)	1:1,000
ALDH1	Abcam (ab227948)	1:1,000
CD24	Thermo Fisher Scientific (MA5-11828)	1:1,000
CD44	Abcam (ab157107)	1:1,000
β-actin	Sigma-Aldrich (A3854)	1:20,000

### 2.9 Aldefluor assay

To identify aldehyde dehydrogenase (ALDH) positive cells, the Aldefluor assay (Aldefluor Stem Cell kit; StemCell Technologies, Vancouver, Canada) was used as described by (Schwarcz et al., 2024a).

### 2.10 Thiobarbituric acid-reactive substances (TBARS) assay

Capan-2 cells were cultured in T150 flasks and treated with UDCA for 48 h. Cells were washed with PBS, scraped and collected by centrifugation. After adding 8.1% SDS, 20% acetic acid, 0.8% thiobarbituric acid (TBA), and distilled water to the cell pellet, the sample was incubated at 96°C for 1 h. Samples were cooled down and centrifuged, and then the absorbance of the supernatant was measured at 540 nm.

### 2.11 Measurement of mitochondrial activity

Mitochondrial function was measured using the Seahorse XF96 analyzer (Agilent Technologies, Santa Clara, CA, United States) as described by (Kovács et al., 2023; Schwarcz et al., 2024a). Briefly, cells were seeded into a 96-well Seahorse assay plates (5,000 cells/well) and treated with UDCA or vehicle for 48 h. After 2 days of treatment, cells were washed and incubated in pre-warmed XF assay media in a CO_2_-free incubator at 37°C for 1 h before the measurement. A XF96 sensor cartridge was hydrated overnight in XF Calibrant Solution. Mitochondrial function was analyzed by performing five baseline oxygen consumption rate (OCR) measurements before a subsequent five measurements following injection of etomoxir (CPT-1 inhibitor; 50 μM final concentration), oligomycin (ATP synthase inhibitor; 10 μM final concentration), and antimycin A (mitochondrial complex III inhibitor; 10 μM final concentration). Data was normalized to protein content which was determined using SRB assay (as described in 2.3 section). Details of calculations for metabolic parameters are described in (Kovács et al., 2023). The following parameters were calculated: Basal respiration was calculated from baseline after subtracting antimycin respiration. Etomoxir-resistant OCR (etomoxir - antimycin) refers to oxygen consumption related to glucose and amino acid oxidation. Etomoxir-sensitive OCR (baseline - etomoxir) refers to fatty acid oxidation. Oligomycin-resistant respiration (oligomycin - antimycin) corresponds to uncoupled respiration. Oligomycin-sensitive OCR (baseline - oligomycin) refers to ATP-linked respiration.

### 2.12 Measurement of mitochondrial membrane potential

Mitochondrial membrane potential was determined by 3,3′-dihexyloxacarbocyanine iodide (DioC6) staining. Capan-2 cells were seeded in 6-well plate (150.000 cells/well). After 2 day UDCA treatment, cells were stained with 40 nM DioC6 (MedChemExpress, cat # HY-D0084) for 30 min. Then cells were washed with phosphate-buffered saline (PBS), harvested by trypsinization and subjected to flow cytometric analysis (FACSCalibur, BD Biosciences). Control cells were treated with 10 μM Carbonyl cyanide-4-(trifluoromethoxy)phenylhydrazone (FCCP) to dissipate mitochondrial membrane potential. The value measured in the FCCP-treated cells were subtracted from all groups.

### 2.13 Database search

Patient survival data was retrieved from the GEPIA2 database (Tang et al., 2019).

### 2.14 Statistical analysis

Statistical analyses were performed using GraphPad Prism 8.0.1 software. The results are presented as the means ± SD and p < 0.05 was considered statistically significant. Normality was tested using the D’Agostino and Pearson normality test. For comparison of UDCA and vehicle treated groups paired t-test was used. One- or two-way analysis of variance test (ANOVA) followed by Dunnett’s or Tukey’s honestly significant *post hoc* test were used for multiple comparisons. Statistical tests and *post hoc* tests are indicated in the corresponding figure captions. Nonlinear regression was performed using the GraphPad program “[Inhibitor] vs. response-variable slope (four parameters)” utility to determine IC_50_ values.

## 3 Results

### 3.1 Ursodeoxycholic acid reduces proliferation of pancreatic adenocarcinoma cells

**FIGURE 1:**
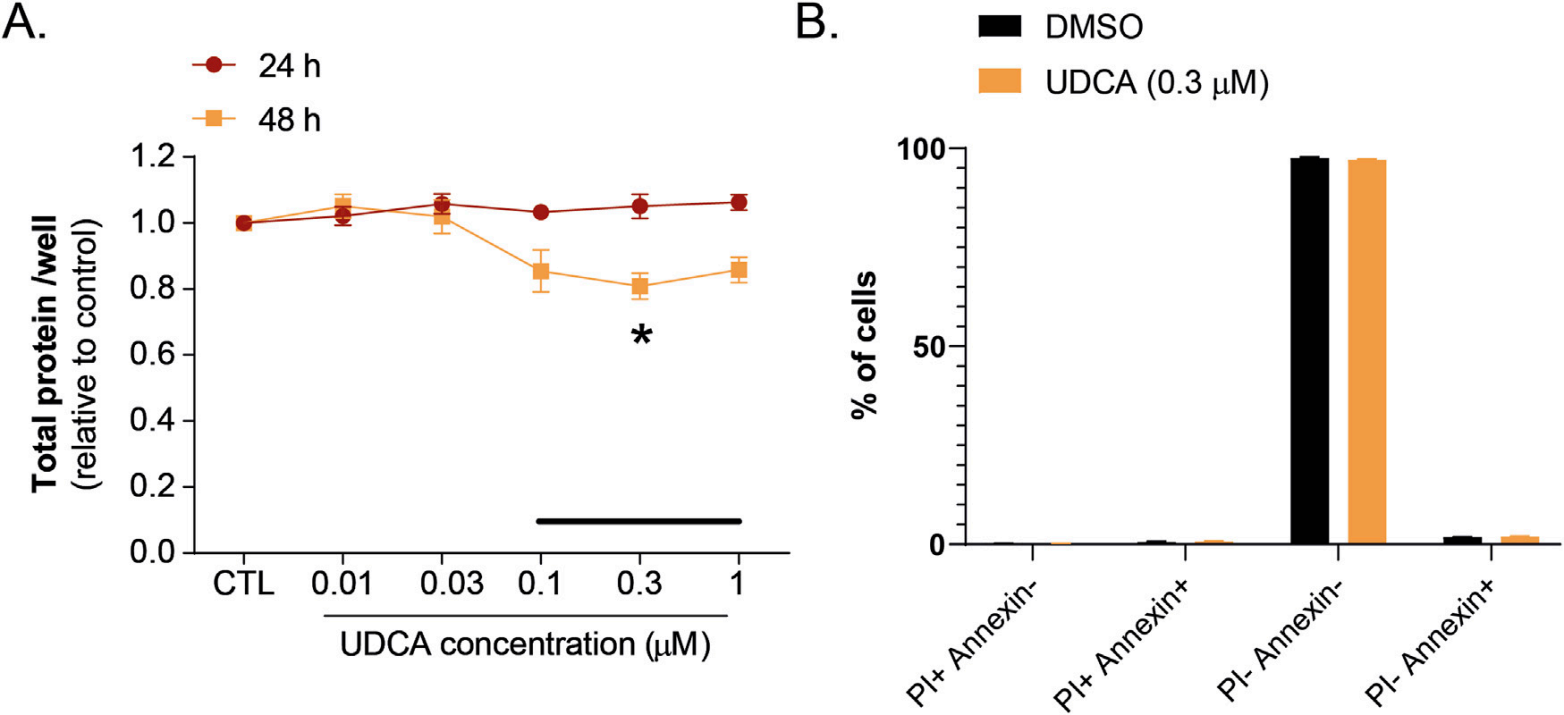
UDCA inhibits the proliferation of Capan-2 cells without affecting apoptosis. **(A)** Capan-2 cells (3,000 cells/well) were plated in 96 well plates and were treated with UDCA for the indicated concentrations and time. Cell count was determined using SRB assay. **(B)** Capan-2 cells (150,000 cells/well in a 6-well plates) were treated with UDCA (0.3 µM) for 48 h; then cells were stained with Annexin-FITC-PI Apoptosis Kit and analyzed by flow cytometry (n = 3). Values in SRB assay were expressed as compared to vehicle-treated controls. Statistical difference was assessed using One-way ANOVA followed by Dunnett’s *post hoc* test. Black line indicate the human serum reference concentration range of UDCA. * indicate p < 0.05, vehicle-treated vs. UDCA-treated groups. Abbreviation: DMSO–dimethyl sulfoxide, PI–propidium iodide, UDCA–ursodeoxycholic acid.

Capan-2 cells were treated with different concentrations of UDCA (from 0.01 µM to 1 µM) and SRB assay was conducted. UDCA inhibited the proliferation of Capan-2 cells in a concentration and time-dependent manner. After 2 days treatment, the effect of UDCA on cell proliferation was significant at a concentration of 0.3 µM which corresponds to the normal human serum concentration of UDCA (Figure 1A). This UDCA concentration was used in the subsequent experiments. We assessed whether the slower proliferation was due to the toxicity of UDCA on PDAC cells. Our results showed that UDCA did not increase either the proportions of propidium–iodide positive, the Annexin–FITC–propidium–iodide double-positive (necrotic) and the Annexin-FITC positive (apoptotic) cells (Figure 1B)

### 3.2 Ursodeoxycholic acid inhibits EMT in Capan-2 cells

After finding that UDCA is cytostatic in pancreatic adenocarcinoma cells, we investigated how UDCA affects different properties of pancreatic adenocarcinoma by assessing the classical hallmarks of cancer known to be affected by bacterial metabolites (Kovács et al., 2021).

**FIGURE 2:**
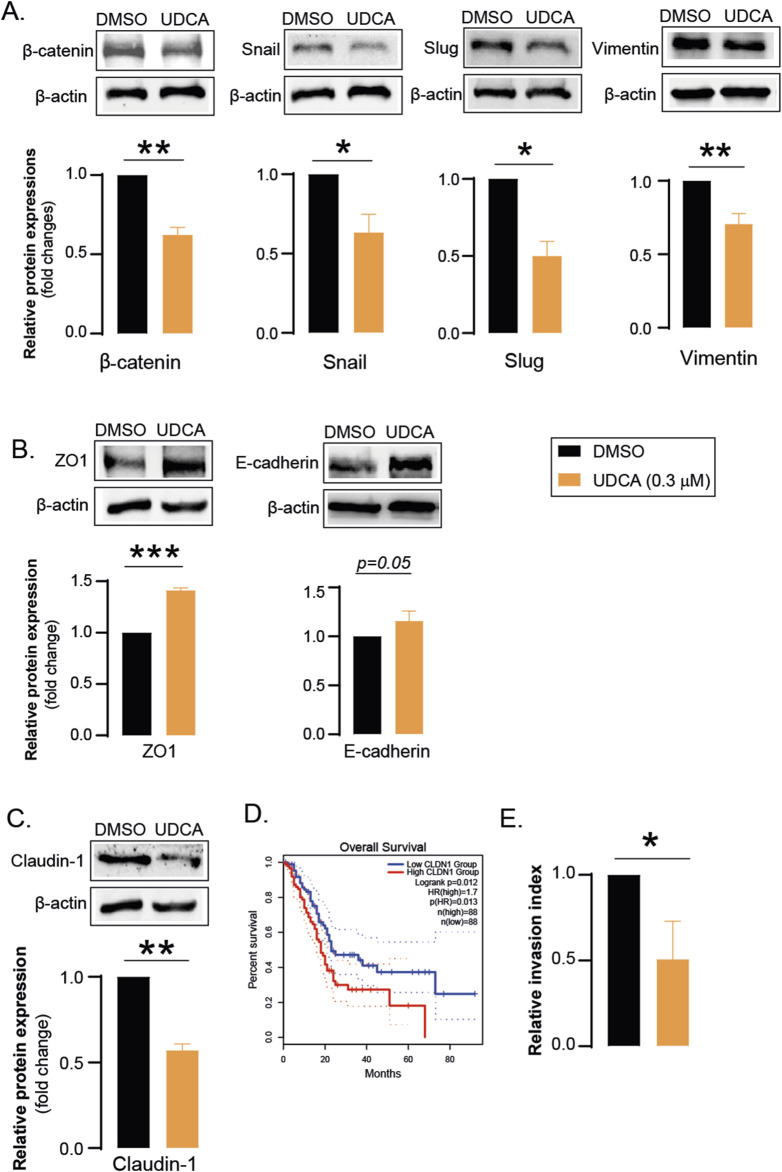
UDCA inhibits EMT and invasion. **(A–C)** Capan-2 cells (150.000 cell/well) were seeded in 6-well plate and were treated with 0.3 µM UDCA for 48 h, then cells were harvested. Cellular lysates were analyzed by SDS-PAGE followed by Western blot. The blots were probed with the antibodies indicated and blots were subjected to densitometry. **(D)** The effect of tumoral claudin-1 expression was retrieved from the GEPIA2 database. Data was assessed on 16 April 2024. **(E)** Tumor cell invasion was assessed using matrigel invasion chambers as described in Materials and Methods. Statistical difference was assessed using t-test. *, ** and *** denotes statistically significant difference between the vehicle-treated and UDCA-treated groups at p < 0.05, p < 0.01 and p < 0.001, respectively. Abbreviations: DMSO–dimethyl sulfoxide, UDCA–ursodeoxycholic acid, ZO1 – zona occludens 1, CLDN1– claudin-1.

UDCA reduced the expression of genes associated with EMT. The protein levels of mesenchymal markers including β-catenin, Snail, Slug and Vimentin were suppressed after UDCA treatment (Figure 2A) while the level of epithelial ZO1 and E-cadherin were increased (Figure 2B). Interestingly, UDCA reduced the expression of tight junction protein, Claudin-1 (Figure 2C) whose reduced expression correlates with better survival in pancreatic adenocarcinoma patients (Figure 2D). In line with the decreased expression of EMT-related genes, we found that UDCA reduces the invasiveness of Capan-2 cells (Figure 2E).

### 3.3 Ursodeoxycholic acid reduces stem cell properties of Capan-2 cells through inducing oxidative and nitrosative stress

**FIGURE 3:**
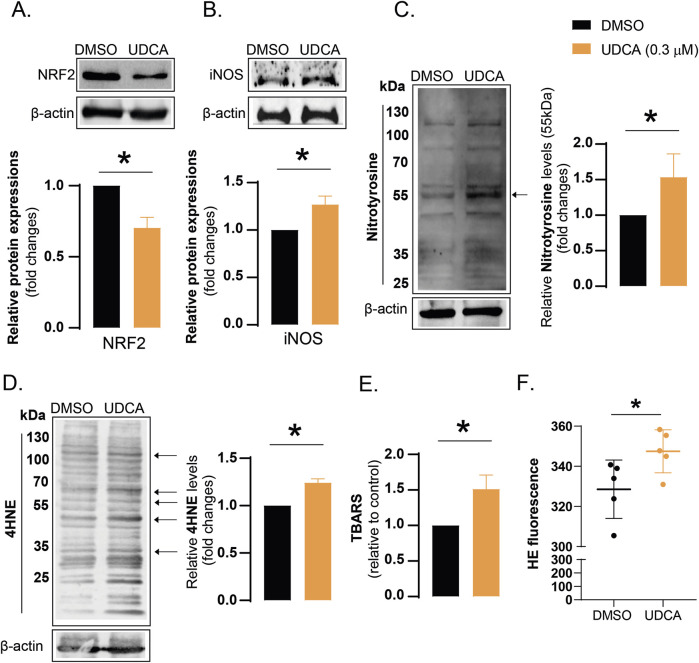
UDCA treatment induces oxidative and nitrosative stress in Capan-2 cells. **(A–D)** Capan-2 cells (150.000 cells/well) were seeded in 6-well plate and were treated with 0.3 µM UDCA for 48 h, then cells were harvested. Cellular lysates were analyzed by SDS-PAGE followed by Western blot. The blots were probed with the antibodies indicated and blots were subjected to densitometry. **(E)** Capan-2 cells were cultured in T150 flasks and were treated with 0.3 µM UDCA for 48 h, then cells were harvested and TBARS assay was performed as described in Materials and methods. **(F)** Capan-2 cells were plated in 6-well plates (150.000 cells/well) and were treated with 0.3 µM UDCA or DMSO for 48 h. Cells were stained with 2.5 µM hydroethidine for 30 min and fluorescence signal was measured by flow cytometry. Statistical difference was assessed using t-test. * denotes statistically significant difference between the vehicle-treated control and the UDCA-treated groups at p < 0.05. Abbreviations: HE–hydroethidine, iNOS–inducible nitric oxide synthase, NRF2 – nuclear factor, erythroid-derived 2-like 2, DMSO–dimethyl sulfoxide, TBARS–thiobarbituric acid reactive substances, UDCA–ursodeoxycholic acid, 4HNE – 4-hydroxynonenal.

Oxidative stress is an imbalance between the production and elimination of free radicals. UDCA treatment reduced the protein expression of NRF2, that is a key regulator of the expression of proteins with antioxidant properties (Figure 3A). The mRNA expression level of NRF2 target genes was also decreased after UDCA treatment (see Supplementary Figure S1). The production of nitric oxide (NO), another reactive species, is catalyzed by the nitric oxide synthase enzymes, of which inducible nitric oxide synthase (iNOS) can produce the largest quantities. UDCA increased iNOS protein expression (Figure 3B), that likely comes along with high NO levels (Pacher et al., 2007). Peroxynitrite (ONOO^−^), formed in the reaction of superoxide (O_2_^−^) and NO, is a destructive reactive species. Peroxynitrite can modify aromatic amino acids, therefore, nitrotyrosine is a footprint of peroxynitrite formation. Nitrotyrosine levels were increased in UDCA-treated cells (Figure 3C). 4-hydroxynonenal (4HNE) and thiobarbituric acid reactive substances (TBARS), by-products of lipid peroxidation, are widely used markers for oxidative stress, increased after UDCA treatment in Capan-2 cells (Figures 3D,E). In line with that, we observed increased hydroethidine signal after UDCA treatment indicating increased superoxide production (Figure 3F).

**FIGURE 4:**
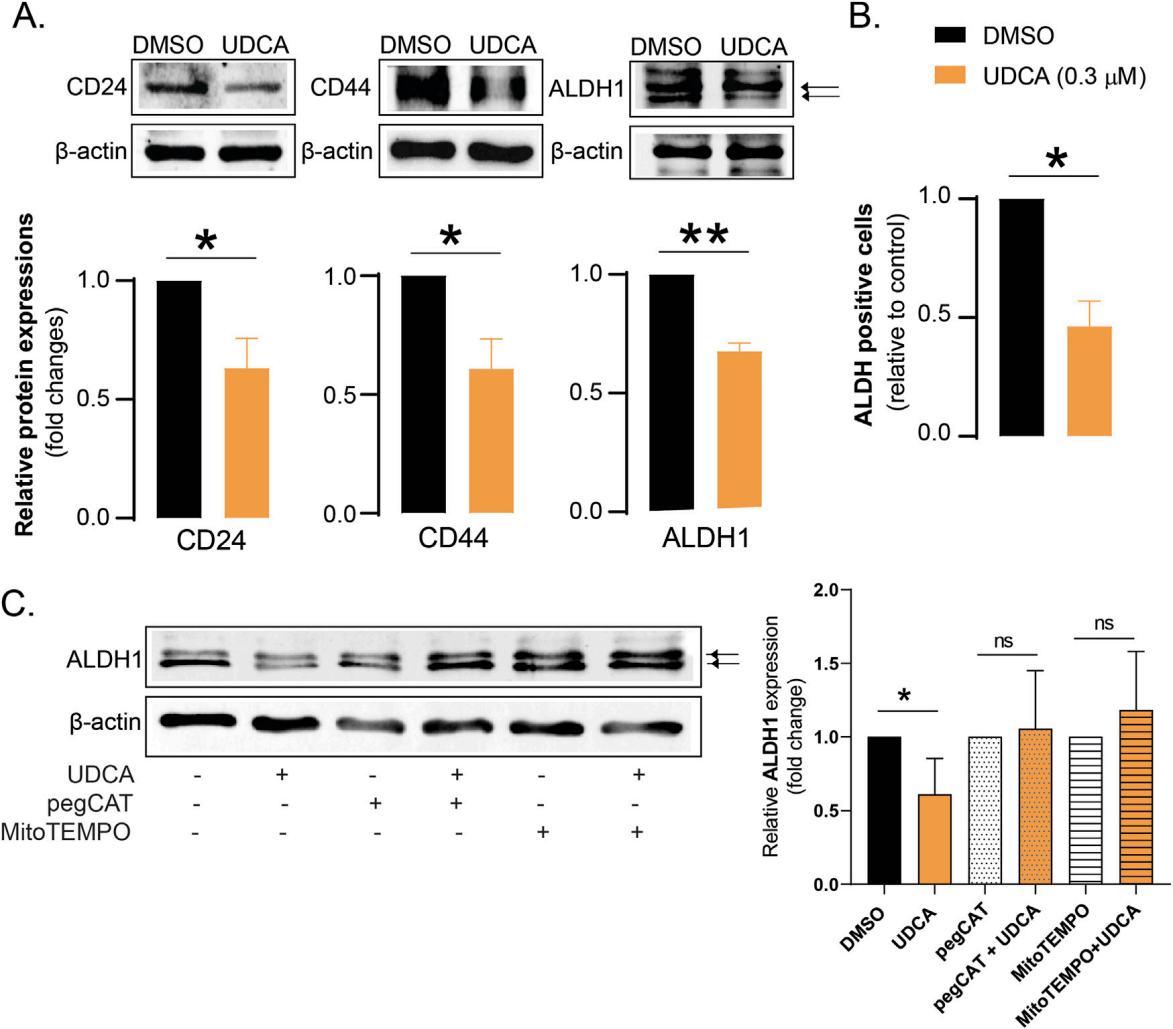
UDCA reduces the proportions of cancer stem cells through bringing about a more oxidative/nitrosative environment **(A)**. Capan-2 cells (150.000 cells/well) were seeded in 6-well plates and were treated with 0.3 µM UDCA for 48 h, then cells were harvested. Cellular lysates were analyzed by SDS-PAGE followed by Western blot. The blots were probed with the antibodies indicated and blots were subjected to densitometry. **(B)** Capan-2 cells (150.000 cells/well) were seeded into 6-well plates and treated with UDCA (0.3 µM) for 48 h, then cells were subjected for a vital stain for ALDH activity. The proportions of ALDH positive cells were determined by flow cytometry. **(C)** Capan-2 cells (150.000 cells/well) were seeded in 6-well plates and were treated with UDCA, pegCAT and MitoTEMPO alone or in combination for 48 h, then cells were harvested. Cellular lysates were analyzed by SDS-PAGE followed by Western blot. The blots were probed with the ALDH1 antibody and blots were subjected to densitometry. * and ** indicates statistically significant difference between vehicle-treated and UDCA-treated groups at p < 0.05, p < 0.01, respectively. Abbreviations: ALDH1 – aldehyde dehydrogenase 1, DMSO–dimethyl sulfoxide, pegCAT–pegylated catalase, UDCA–ursodeoxycholic acid.

Bile acid species can interfere with stem cell properties of cancer cells in multiple carcinomas, as breast cancer (Schwarcz et al., 2024b), or PDAC (Kim E. K. et al., 2017; Schwarcz et al., 2024a), therefore, we assessed stem cell markers in UDCA-treated Capan-2 cells. The protein expression of CD24, CD44 or aldehyde dehydrogenase-1 (ALDH1) was determined in Capan-2 cells treated with UDCA. UDCA treatment reduced the protein expression of CD24, CD44 and ALDH1 (Figure 4A) and decreased the proportions of ALDH-positive cells (Figure 4B). These changes collectively suggest that UDCA can reduce the proportions of cancer stem cells.

Next, we assessed whether there is a connection between a more oxidative/nitrosative environment and reduction of the proportions of cancer stem cells. Pegylated catalase (pegCAT) was used for the elimination of hydrogen peroxide and MitoTEMPO was used for the scavenging of reactive species of mitochondrial origin. Both pegCAT and MitoTEMPO inhibited decreases in the expression of ALDH1 upon UDCA treatment (Figure 4C) pointing out that increases a more oxidative/nitrosative environment can reduce the proportions of cancer stem cells.

### 3.4 Ursodeoxycholic acid induces mitochondrial activity

**FIGURE 5:**
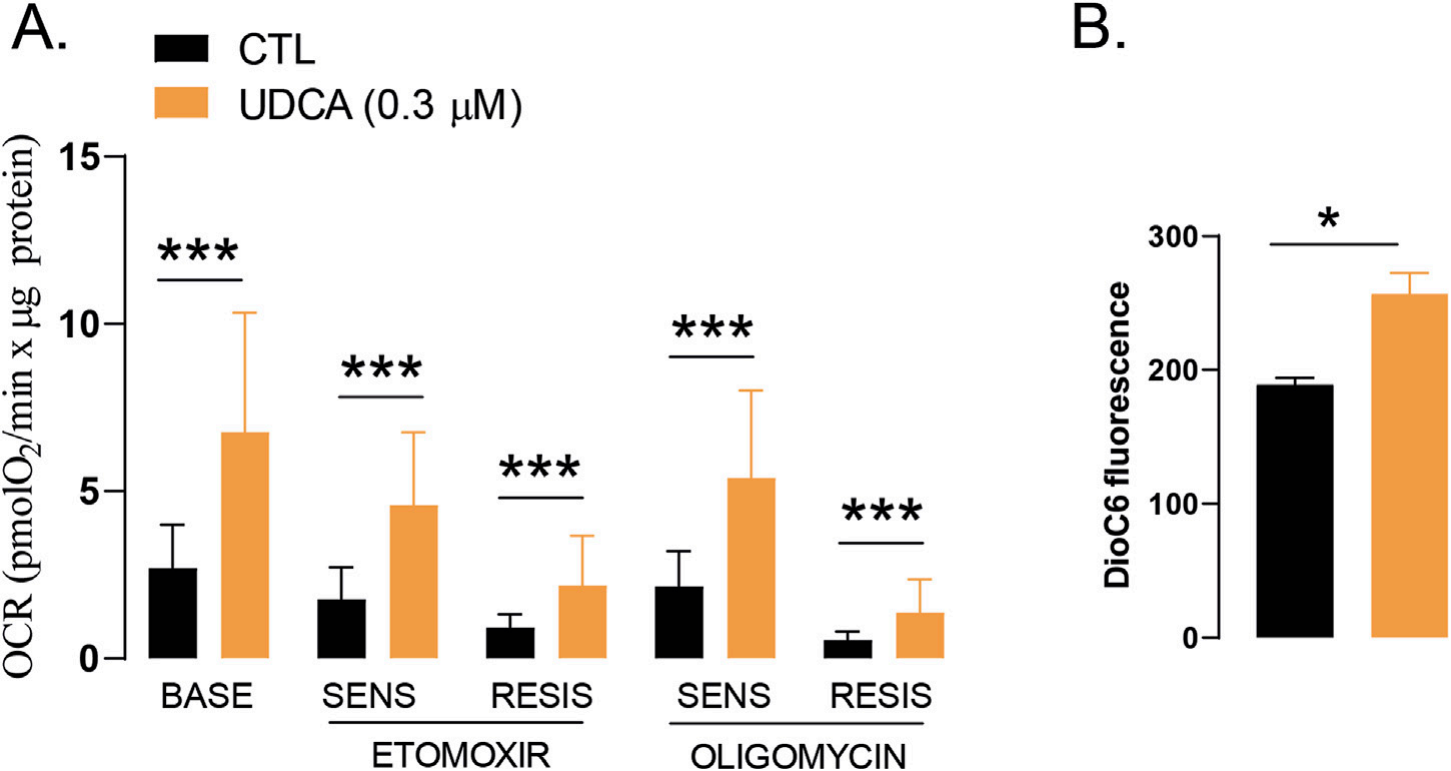
UDCA induces mitochondrial activity. **(A)** Capan-2 cells (5,000 cells/well) were seeded into Seahorse plates and were treated with 0.3 µM UDCA for 48 h, then oximetry, protein content determination and, subsequently, calculations took place as described in the Materials and Methods. Data were log2 transformed to achieve normal distribution. **(B)** Mitochondrial membrane potential was measured by DioC6 staining. Capan-2 cells were seeded in 6-well culture plate and were treated with UDCA or DMSO for 48 h. Then cells were stained with DioC6 (40 nM) for 30 min and were analyzed by flow cytometry. * and *** denotes statistically significant difference between the control and the UDCA-treated groups at p < 0.05 and p < 0.001. Abbreviations: BASE–baseline respiration, CTL–control, OCR–oxygen consumption rate, RESIS–resistant, SENS–sensitive, UDCA–ursodeoxycholic acid.

Changes to cellular metabolism are also considered as a cancer hallmark in which changes to mitochondrial oxidative metabolism plays key role (Hanahan and Weinberg, 2011; Hanahan, 2022). Therefore, we investigated whether UDCA can influence mitochondrial metabolism. Seahorse analysis demonstrated that UDCA enhances mitochondrial oxidation, namely, basal respiration and its subfractions, etomoxir-sensitive respiration (representing fatty acid oxidation), etomoxir-resistant respiration (representing glucose and amino acid oxidation), oligomycin-sensitive, ATP-linked respiration and oligomycin-resistant respiration (Figure 5A). UDCA induces DioC6 fluorescence suggesting increases in mitochondrial membrane potential (Figure 5B). This observation together with the lack of the induction of cell death (Figure 1B), suggest a more coupled mitochondrial system that may in fact be prone for ROS production.

### 3.5 The effects of ursodeoxycholic acid can be elicited in another PDAC cell line, BxPC-3

**FIGURE 6:**
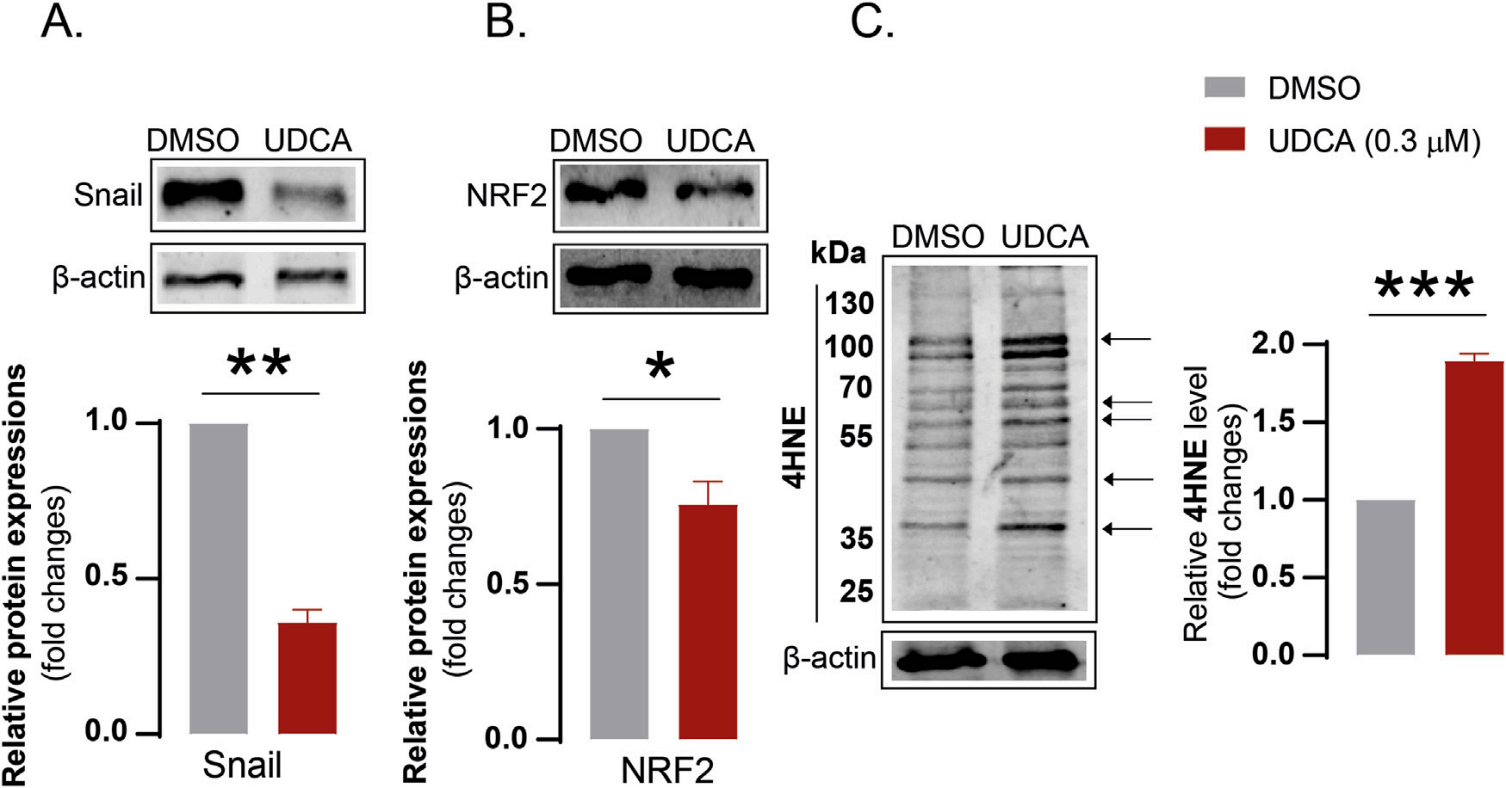
The effects of UDCA can be elicited in BxPC-3 cells. **(A–C)** BxPC-3 cells (100.000 cells/well) were seeded into 6-well plates and were treated with 0.3 µM UDCA for 48 h, then were harvested. Cellular lysates were analyzed by SDS-PAGE followed by Western blot. The blots were probed with the antibodies indicated and blots were subjected to densitometry. *, ** and *** denotes statistically significant difference between vehicle-treated and UDCA-treated groups at p < 0.05, p < 0.01 or p < 0.001, respectively. Abbreviations: NRF2 – nuclear factor, erythroid-derived 2-like 2, DMSO–dimethyl sulfoxide, UDCA–ursodeoxycholic acid, 4HNE – 4-hydroxynonenal.

The effects of UDCA were studied on another two pancreatic adenocarcinoma cell lines, BxPC-3 and PancTu-1. UDCA treatment decreased Snail (Figure 6A) and NRF2 (Figure 6B) protein expression and increased 4HNE levels (Figure 6C) in BxPC-3 cells, similar to our observation in Capan-2 cells. The effects of UDCA on Snail, E-cadherin and iNOS protein level were also verified in PancTu-1 cell line (see Supplementary Figure S2).

### 3.6 Ursodeoxycholic acid-induced responses are not observed in normal human fibroblasts

**FIGURE 7:**
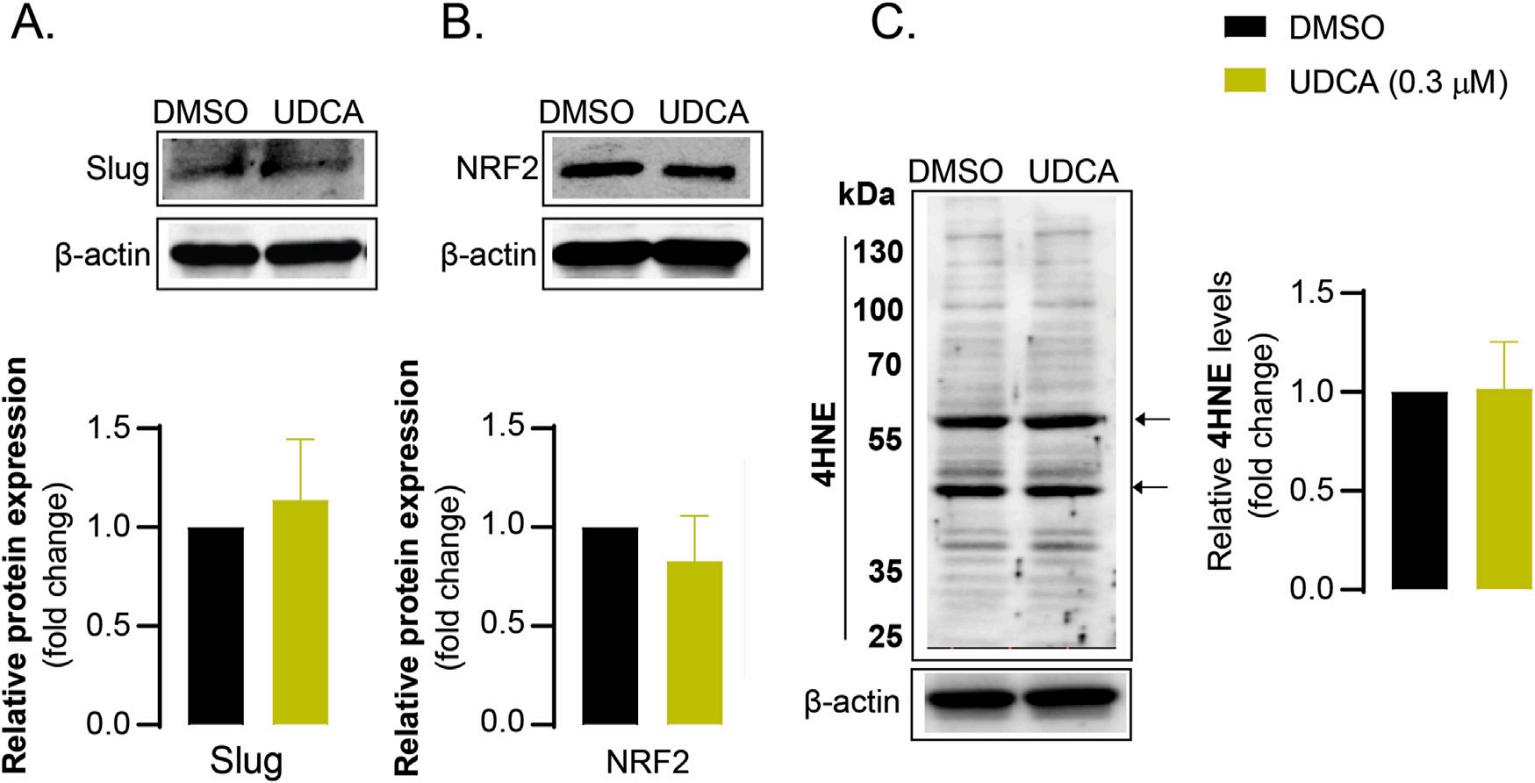
The effects of UDCA are not observed in fibroblast cells **(A–C)** Human dermal fibroblast cells (200.000 cells/well) were seeded into 6-well plates and were treated with 0.3 µM UDCA for 48 h, then were harvested. Cellular lysates were analyzed by SDS-PAGE followed by Western blot. The blots were probed with the antibodies indicated and blots were subjected to densitometry. Abbreviations: DMSO–dimethyl sulfoxide, NRF2 – nuclear factor, erythroid-derived 2-like 2, UDCA–ursodeoxycholic acid, 4HNE – 4-hydroxynonenal.

Finally, we found that UDCA had no effect on Slug (Figure 7A), NRF2 (Figure 7B) and 4HNE (Figure 7C) expression levels in human fibroblast cells. These results indicate that UDCA does not modulate EMT and oxidative stress responses in primary, non-transformed human cells, suggesting that the effects of UDCA are selective for PDAC cells.

### 3.7 Ursodeoxycholic acid does not interfere with chemotherapy agents

**FIGURE 8:**
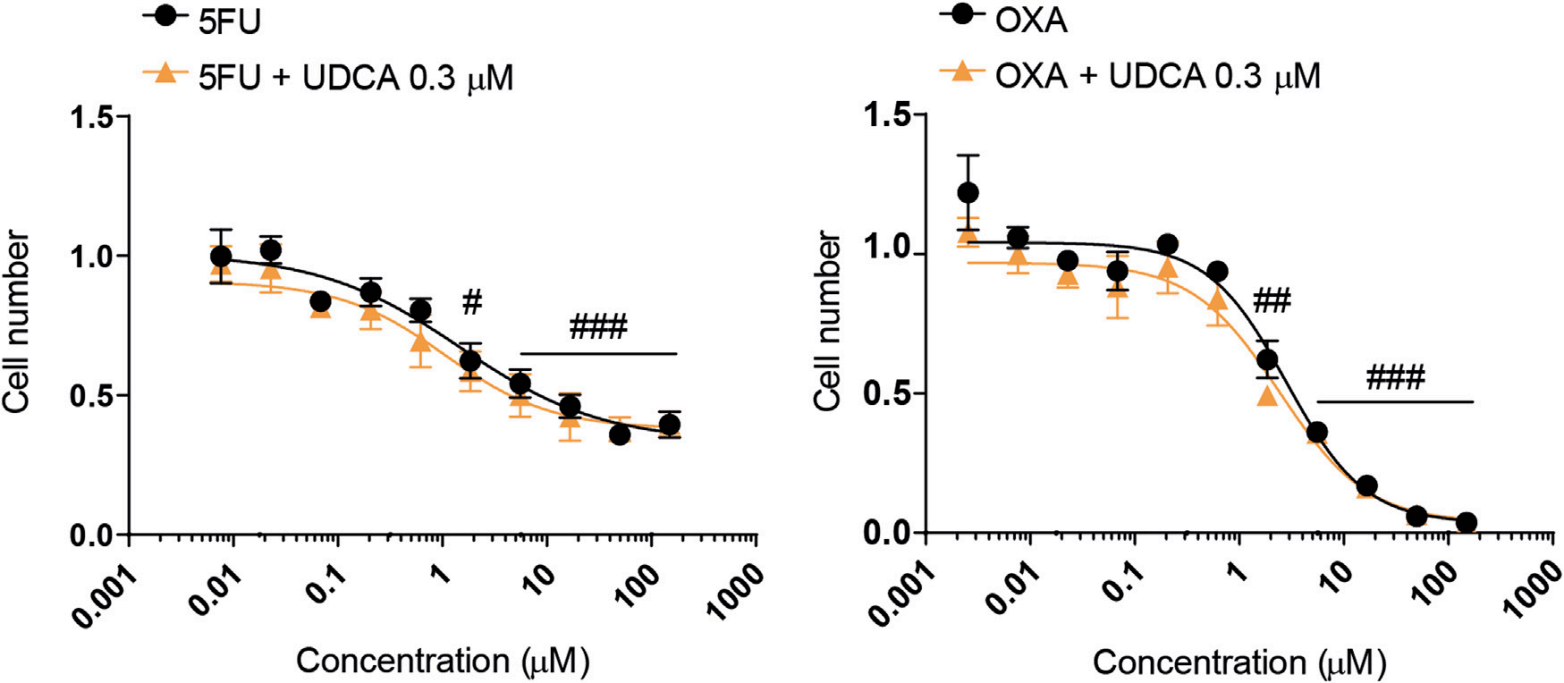
UDCA does not interfere with 5FU and OXA drugs. Capan-2 cells. (5,000 cells/well) were plated to 96-well plates and were treated with one of the cytostatic agents in the indicated concentration range with 0.3 µM UDCA or vehicle for 48 h. Then MTT assay was performed. The results are expressed compared to the vehicle-treated control. Statistical difference was valuated using two-way ANOVA test followed by Tukey *post hoc* test. #, ## and ### denotes statistically significant difference between the cells treated with the lowest concentration of the chemotherapy agents versus the indicated concentration at p < 0.05, p < 0.01 or p < 0.001, respectively. Abbreviations: 5FU - 5-fluorouracil, OXA–oxaliplatin, UDCA–ursodeoxycholic acid.

Finally, as multiple reports have shown an interaction between bacterial metabolites and chemotherapy agents (Donohoe et al., 2012; Lee et al., 2018; Ghosh et al., 2022; Tintelnot et al., 2023; Schwarcz et al., 2024b), we tested the interactions between UDCA and chemotherapy agents used in the management of PDAC (Ducreux et al., 2015; Springfeld et al., 2024). The drugs, 5-fluorouracil and oxaliplatin were tested in different concentrations alone or in combination with UDCA (0.3 µM) using MTT assay similar to (Kacsir et al., 2023; Schwarcz et al., 2024b). UDCA did not impact the kinetic properties, IC_50_ and Hill coefficient (collaborative binding or effect) of chemotherapy agents in modulating the proliferation of Capan-2 cells (Figure 8; Table 2).

**TABLE 2:** The kinetic values of the chemotherapy agent and chemotherapy agent/UDCA combinations.

Chemotherapeutic drug	Metabolite	IC_50_ (±SD)	Hill coefficient (±SD)
5-fluorouracil	—	1.478 (±0.789)	0.643 (±0.049)
	UDCA	1.067 (±0.576)	0.854 (±0.312)
Oxaliplatin	—	2.839 (±0.513)	1.158 (±0.037)
	UDCA	2.450 (±0.579)	1.036 (±0.475)

## 4 Discussion

Hereby, we showed that UDCA, a bacterially produced secondary metabolite, exerts cytostatic effects in models of PDAC. UDCA was found to be beneficial in models of glioblastoma (Yao et al., 2020), neuroblastoma (Fonseca et al., 2017), PDAC (Kim Y. J. et al., 2017), prostate cancer (Lee et al., 2017), melanoma (Yu et al., 2019), hepatocellular carcinoma (Liu et al., 2007; Lim et al., 2010; Chung et al., 2011; Zhu et al., 2014; Liu et al., 2015; Lee et al., 2018), oral squamous carcinoma (Pang et al., 2015), leukemia (Fimognari et al., 2009), gastric cancer (Lim et al., 2011; Lim et al., 2012; Lim and Han, 2015; Wu et al., 2018), oesophageal cancer/Barett’s esophagus (Goldman et al., 2010; Peng et al., 2014; Abdel-Latif et al., 2016), colon cancer (Im and Martinez, 2004; Im et al., 2005; Shah et al., 2006; Feldman and Martinez, 2009; Peiró-Jordán et al., 2012; Saeki et al., 2012; Kim E. K. et al., 2017; Kim et al., 2019), cholangiocarcinoma (Alpini et al., 2004), similar to our findings presented hereby. Importantly, most of these studies applied supraphysiological concentrations of UDCA, 2-4 magnitudes higher than the human serum reference concentration of UDCA. Despite the fact that UDCA is a registered medication, these concentrations cannot be achieved even with repeated administration of UDCA at peak concentration. Therefore, such results may represent effects that are unachievable by the currently available delivery methods of UDCA. However, in models of other cancers, low, close to the physiological concentrations, of bile acids (other than UDCA) were shown to be able to elicit biological effects (Hong et al., 2010; Miko et al., 2018; Kovács et al., 2019; Schwarcz et al., 2023; Schwarcz et al., 2024a; Schwarcz et al., 2024b). A higher number of bile acid species modulate the behavior of PDAC cells (UDCA hereby and (Kim Y. J. et al., 2017), DCA (Schwarcz et al., 2023) and LCA (Booth et al., 2011; Schwarcz et al., 2024a)) than other malignancies, as ovarian or breast cancer for example, (Kovács et al., 2021; Sipos et al., 2021).

Reactive species play key role in regulating tumor cell proliferation and cell death (Hegedus et al., 2018). UDCA elicits oxidative and nitrosative stress in line with the previous observations on UDCA (Booth et al., 2011; Kim Y. J. et al., 2017) or other bile acids (Lechner et al., 2002; Barrasa et al., 2011; Booth et al., 2011; Masubuchi et al., 2016; Kim Y. J. et al., 2017; Kovács et al., 2019; Schwarcz et al., 2023; Schwarcz et al., 2024a). Previous observations on PDAC and the pancreas showed that UDCA downregulated the expression of peroxiredoxin II that subsequently led to oxidative stress (Kim Y. J. et al., 2017). Another bile acid, lithocholic acid, also induced oxidative and nitrosative stress in PDAC cells through reducing the expression of nuclear factor erythroid 2 (NFE2)-related factor 2 (Nfe2l2, or NRF2) (Schwarcz et al., 2024a). NRF2 is a transcription factor that regulates the expression of a set of antioxidant and detoxification genes (Venugopal and Jaiswal, 1996; Nguyen et al., 2009; Malhotra et al., 2010); there are over 200 NRF2-dependent genes in humans (Zhu and Fahl, 2001; Malhotra et al., 2010; Tonelli et al., 2018). The NRF2 system is linked to the apoptotic machinery, wherein either detoxification or cell death induction is active (e.g., PARP1/2 (Wu et al., 2014; Jankó et al., 2023)). NRF2 overactivation usually plays major role in supporting cancer cell growth that is termed NRF2 addiction (Smolková et al., 2020).

Besides the downregulation of NRF2 we identified other sources of the reactive species upon UDCA treatment, namely, the mitochondrial production of reactive species (see MitoTEMPO effects) and iNOS for the production of NO that upon combining with superoxide form peroxynitrite that is a detrimental reactive species (Pacher et al., 2007).

Reactive species production led to a downregulation of stem-ness markers (ALDH1, CD24, CD44). This result suggests that a more oxidative/less reductive environment can inhibit the formation or sustainment of cancer stem cells and, therefore, reduce the risk of metastasis formation and therapy resistance (Batlle and Clevers, 2017). In line with that, metabolite-induced reactive species production was linked to cytostasis (Kovács et al., 2019; Sári et al., 2020a; Sári et al., 2020b). In other words, the mild oxidative stress induced by bacterial metabolites appears to be a central mechanism in metabolite-elicited antineoplastic effects (Kovács et al., 2021).

We observed the induction of mitochondrial oxidative phosphorylation upon UDCA treatment. This metabolic change was observed in the case of multiple bile acids (Watanabe et al., 2006; Goldberg et al., 2011; Miko et al., 2018; Schwarcz et al., 2023; Schwarcz et al., 2024a) and was linked to the induction of cytostasis and apoptosis (Goldberg et al., 2011; Miko et al., 2018; Schwarcz et al., 2023; Schwarcz et al., 2024a). The key role of (mitochondrial) metabolic changes were shown to be implicated in (re)programing cancer stem cells (Sancho et al., 2016; De Francesco et al., 2018). Apparently, multi-pronged effects partake in the UDCA-induced benign reprograming of PDAC cells.

We also assessed whether UDCA may interfere with chemotherapy agents used in the management of PDAC. In fact, multiple studies have shown a multifaceted connection between the microbiome and chemotherapy agents, where 1) bacteria may metabolize chemotherapy agents (Sandrini et al., 2007a; Sandrini et al., 2007b; Vande Voorde et al., 2014; Lehouritis et al., 2015; Alexander et al., 2017; Garcia-Gonzalez et al., 2017; Geller et al., 2017; Geller and Straussman, 2018) and efficacy (Sivan et al., 2015), 2) chemotherapy agents modulate the composition of the microbiome (Fan et al., 2023; Tian et al., 2024) and 3) bacterial metabolites interfere with the activity of chemotherapy agents in other cancers (Donohoe et al., 2012; Lee et al., 2018; Ghosh et al., 2022; Schwarcz et al., 2023; Schwarcz et al., 2024a) and in PDAC (Tintelnot et al., 2023; Wei et al., 2023) similar to other bile acids (Schwarcz et al., 2023; Schwarcz et al., 2024a). In contrast, indole-derivates improved the outcome of chemotherapy (Tintelnot et al., 2023) and interfered with the activity of 5-fluorouracil, doxorubicine and paclitaxel (Schwarcz et al., 2024b).

Overall, we demonstrated that UDCA has beneficial effects on PDAC cells in low concentrations, close to the serum reference range without potentially interfering with chemotherapy. UDCA can be used as a single agent, not only in combination with chemotherapy drugs. UDCA has low toxicity even in pharmacologically relevant concentrations, pointing towards a pathway for reposition. These results suggest the use of UDCA, that is a licensed medication for primary biliary cholangitis or cholesterol gallstone dissolution (Cabrera et al., 2019; Režen et al., 2022; Achufusi et al., 2024), in the management of PDAC.

## Data Availability

The datasets presented in this study can be found in online repositories. The names of the repository/repositories and accession number(s) can be found below: https://figshare.com/s/c051c8a106c42a9a6318.
