# Supplementary material for: Anticarcinogenic effects of ursodeoxycholic acid in pancreatic adenocarcinoma cell models
Source: Front Cell Dev Biol. 2024 Dec 11;12:1487685. doi: 10.3389/fcell.2024.1487685 (PMC11668698; doi:10.3389/fcell.2024.1487685)
Supplement: Supplementary file 1 [file DataSheet3.zip › Western blots_1.2.pptx]

## Slide 1
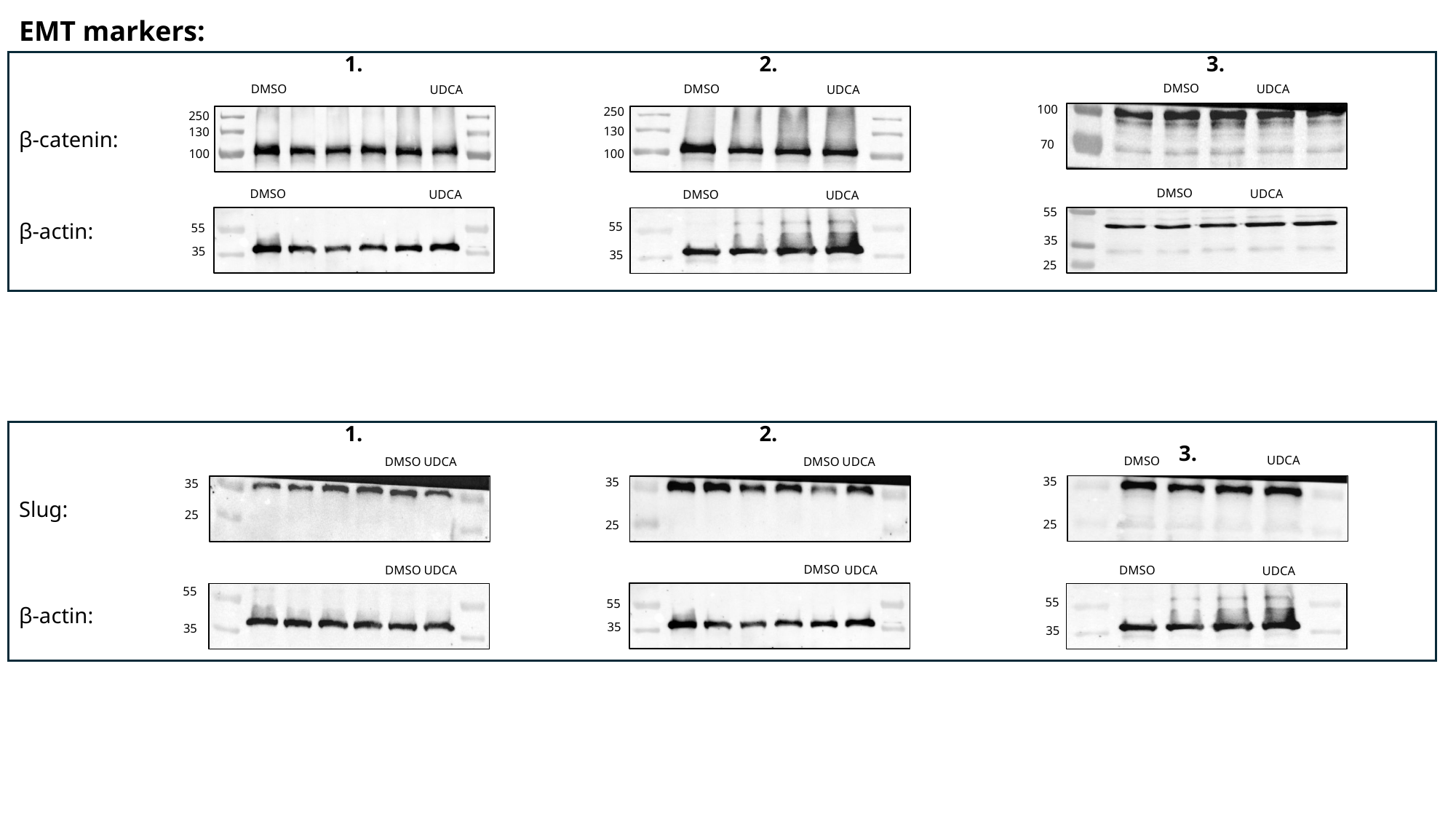

EMT markers:
3.
1.
DMSO
UDCA
2.
β-catenin:
β-actin:
250
130
100
55
35
250
130
100
55
35
DMSO
UDCA
DMSO
UDCA
100
70
55
35
25
DMSO
UDCA
DMSO
DMSO
UDCA
UDCA
2.
1.
3.
Slug:
β-actin:
UDCA
DMSO
35
25
UDCA
DMSO
55
35
UDCA
DMSO
35
25
DMSO
UDCA
55
35
DMSO
UDCA
55
35
UDCA
DMSO
35
25

## Slide 2
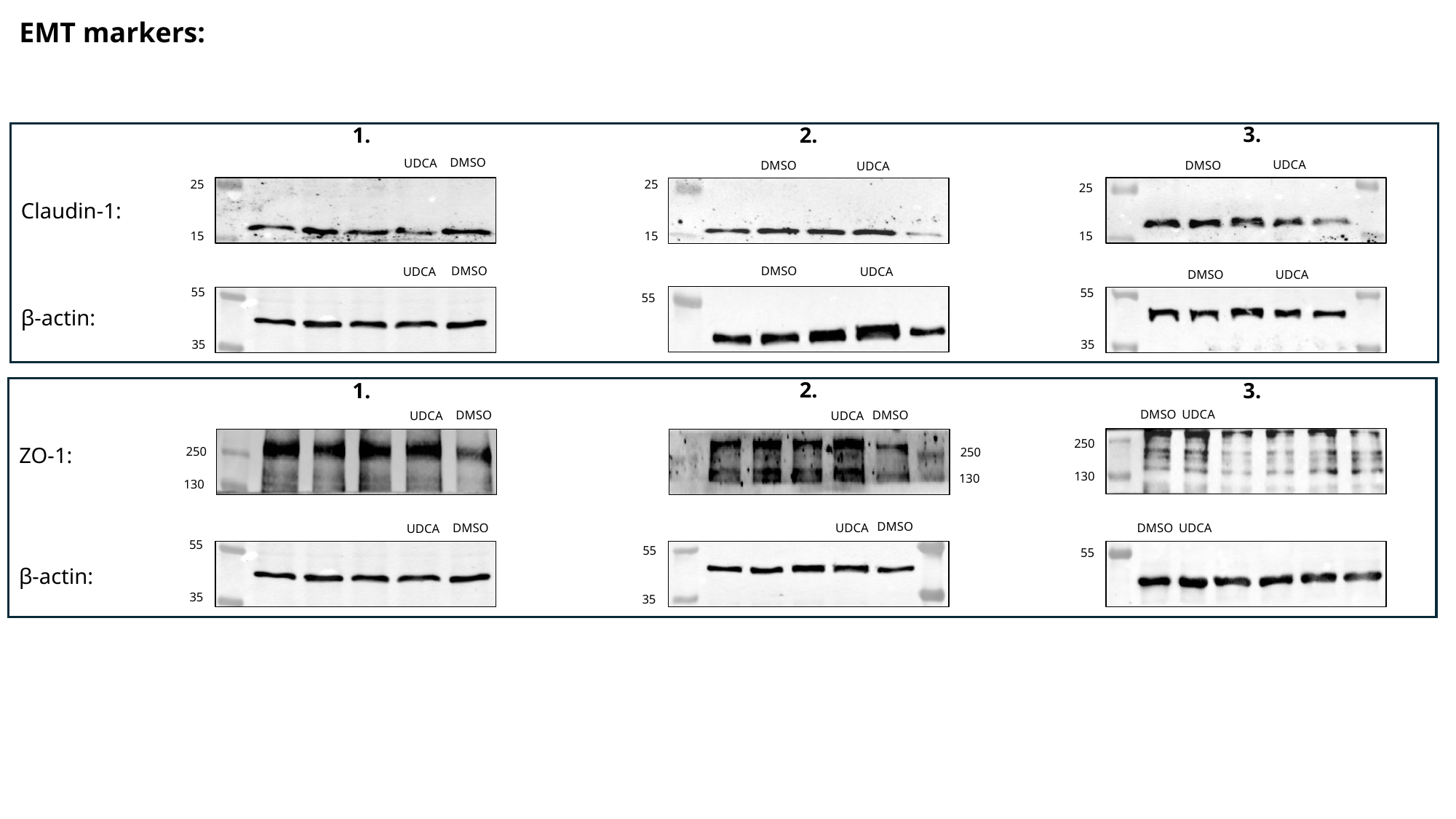

EMT markers:
3.
1.
2.
Claudin-1:
β-actin:
DMSO
UDCA
25
15
DMSO
UDCA
55
35
25
15
DMSO
UDCA
DMSO
UDCA
55
25
15
UDCA
DMSO
55
35
UDCA
DMSO
2.
1.
3.
ZO-1:
β-actin:
DMSO
UDCA
250
130
DMSO
UDCA
55
35
DMSO
UDCA
250
130
DMSO
UDCA
55
35
UDCA
DMSO
250
130
UDCA
DMSO
55
