# Supplementary figures and images for: Anticarcinogenic effects of ursodeoxycholic acid in pancreatic adenocarcinoma cell models (part 1 of 2)
Source: Front Cell Dev Biol. 2024 Dec 11;12:1487685. doi: 10.3389/fcell.2024.1487685 (PMC11668698; doi:10.3389/fcell.2024.1487685)

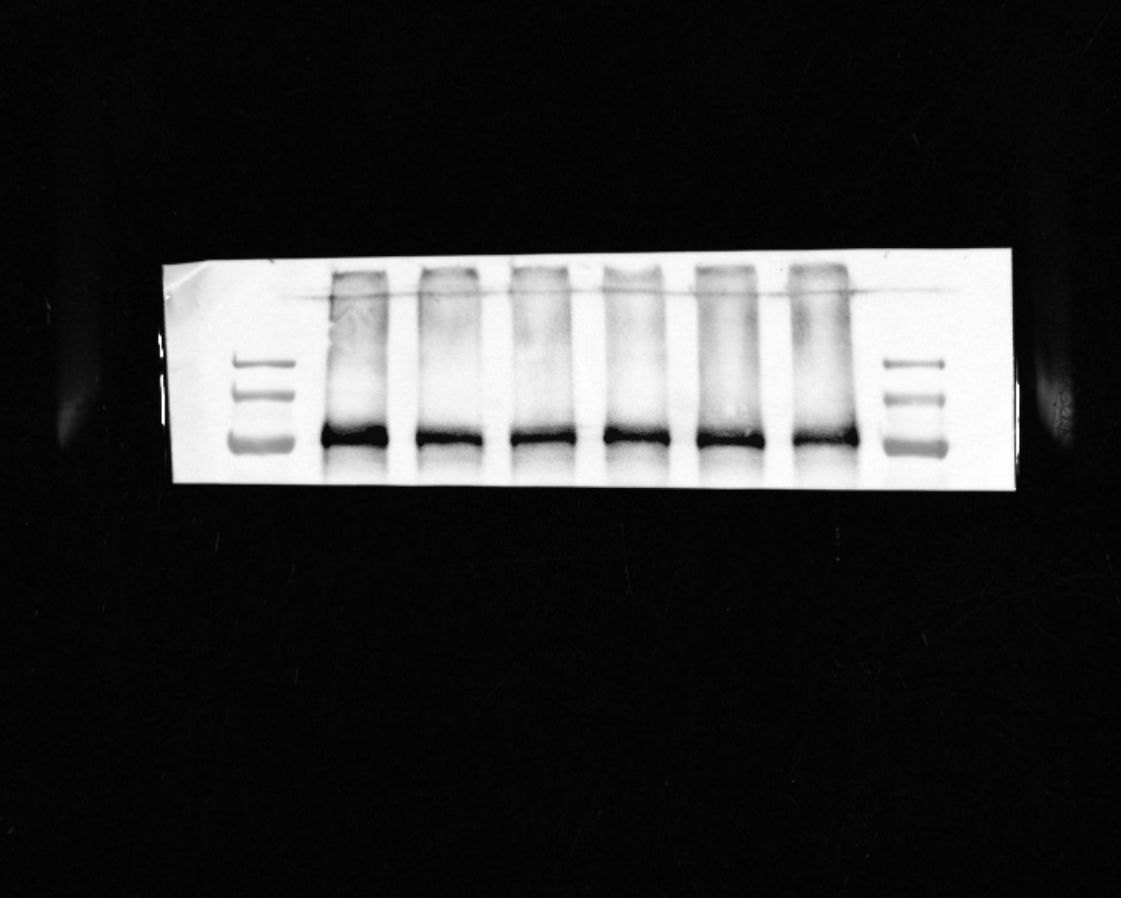

Supplement: Supplementary file 1 [file DataSheet3.zip › Capan2_B-catenin/TIF/B-catenin 1. (2020. 08. 06. Edit II. B-catenin_5).tif]

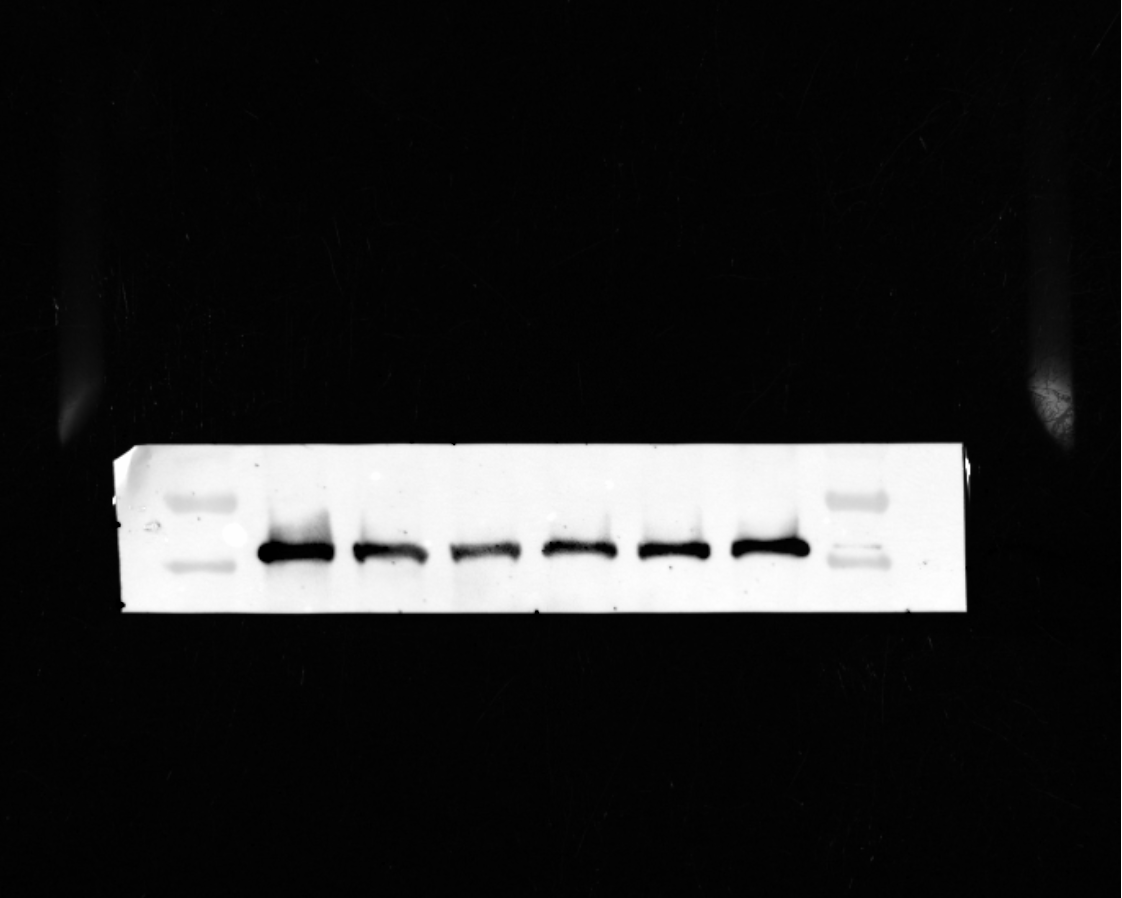

Supplement: Supplementary file 1 [file DataSheet3.zip › Capan2_B-catenin/TIF/B-catenin 1. Actin (2020. 08. 07. Edit II. Aktin B-catenin, Slug_4).tif]

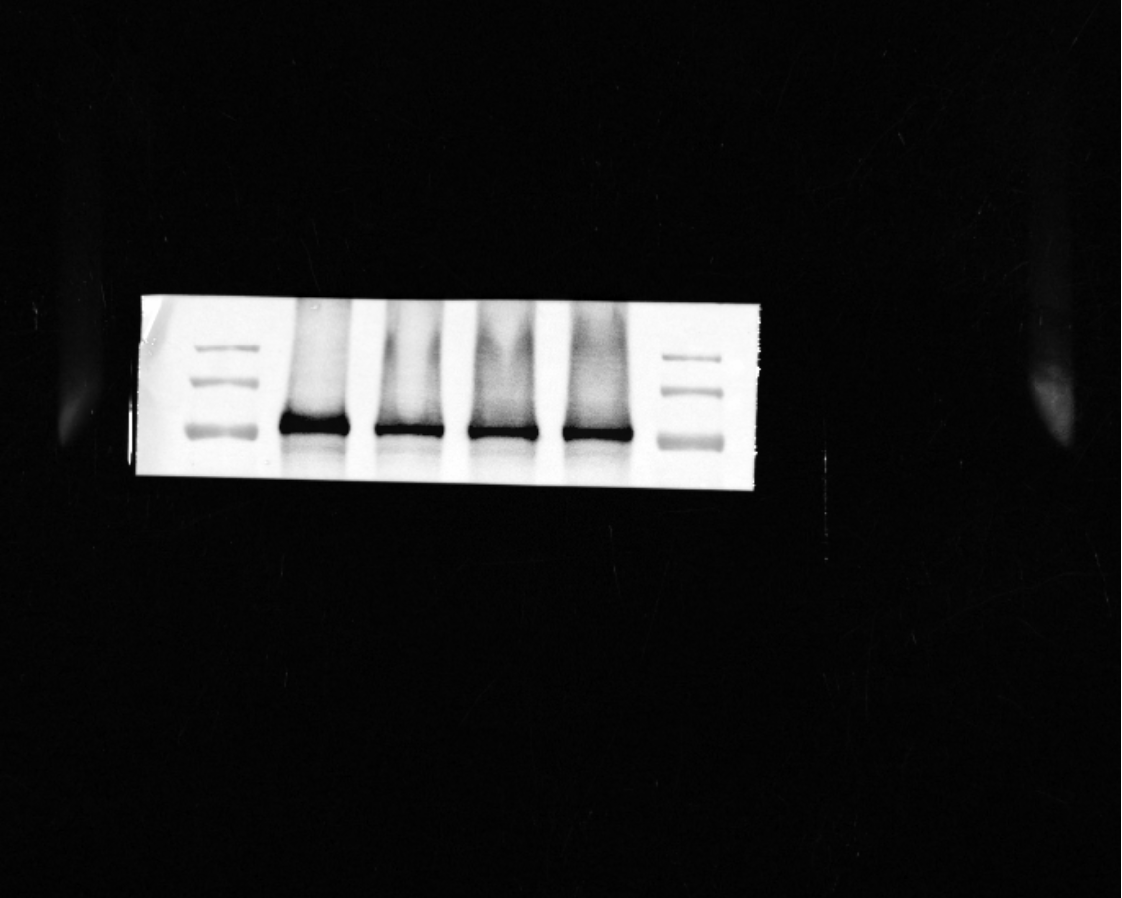

Supplement: Supplementary file 1 [file DataSheet3.zip › Capan2_B-catenin/TIF/B-catenin 2. (2020. 08. 06. 32 B-catenin_4).tif]

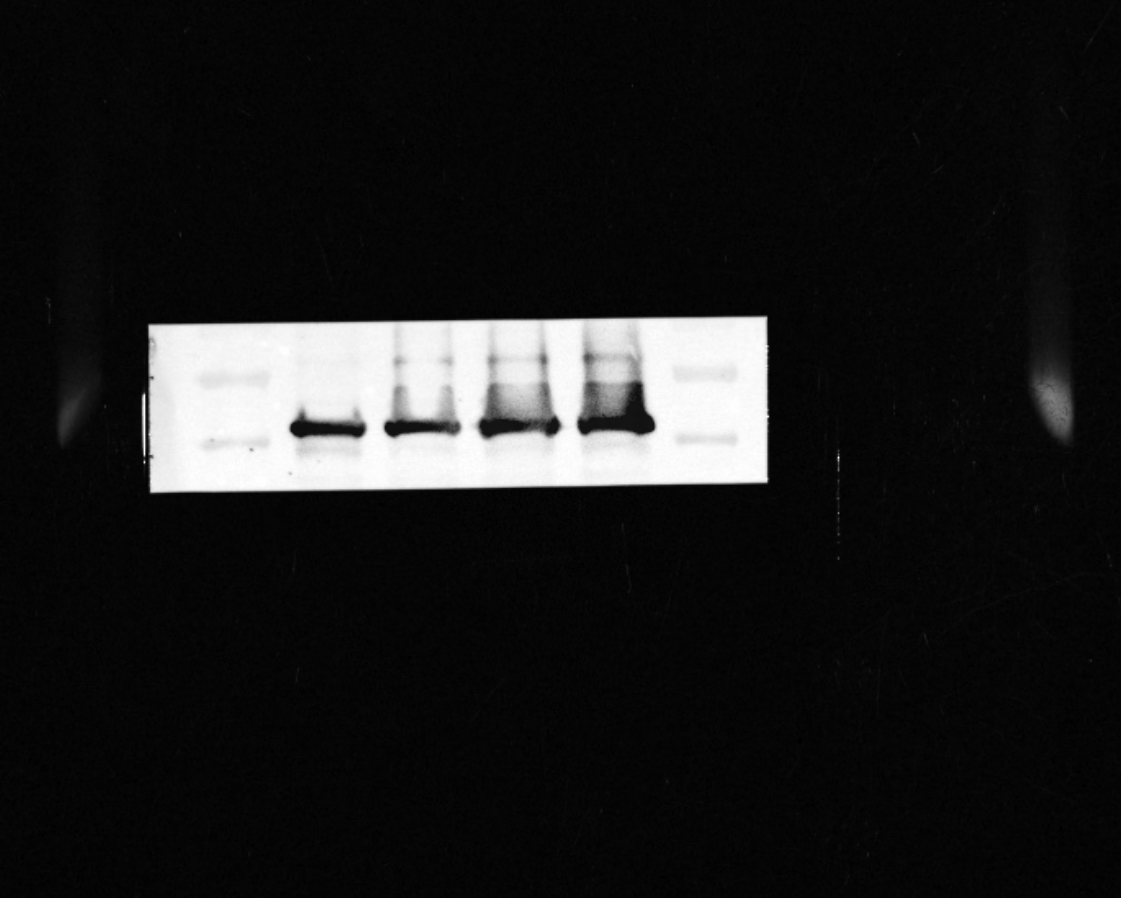

Supplement: Supplementary file 1 [file DataSheet3.zip › Capan2_B-catenin/TIF/B-catenin 2. Actin (2020. 08. 07. X32 Aktin Bcat, Slug_3+2020. 08. 07. X32 Aktin Bcat, Slug_2).tif]

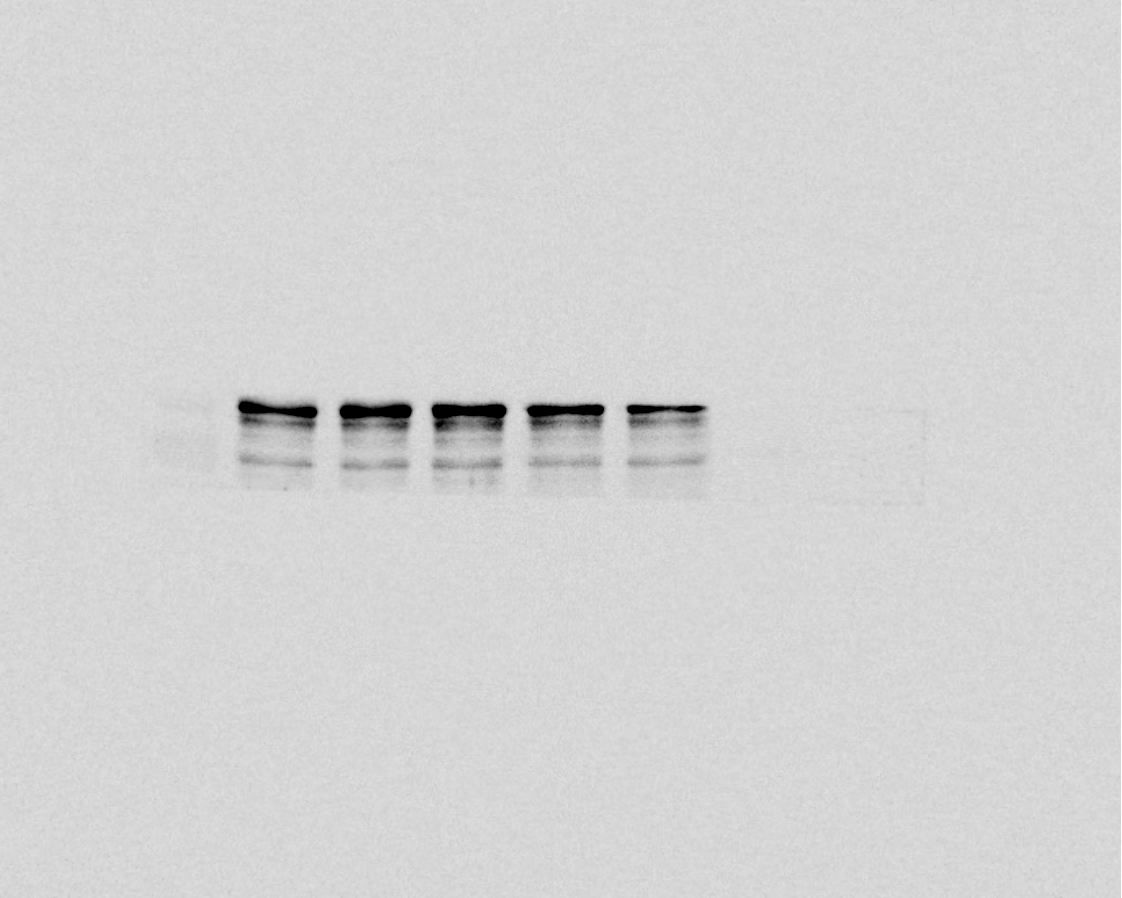

Supplement: Supplementary file 1 [file DataSheet3.zip › Capan2_B-catenin/TIF/B-catenin 3. (20191010 B catenin zs._2).tif]

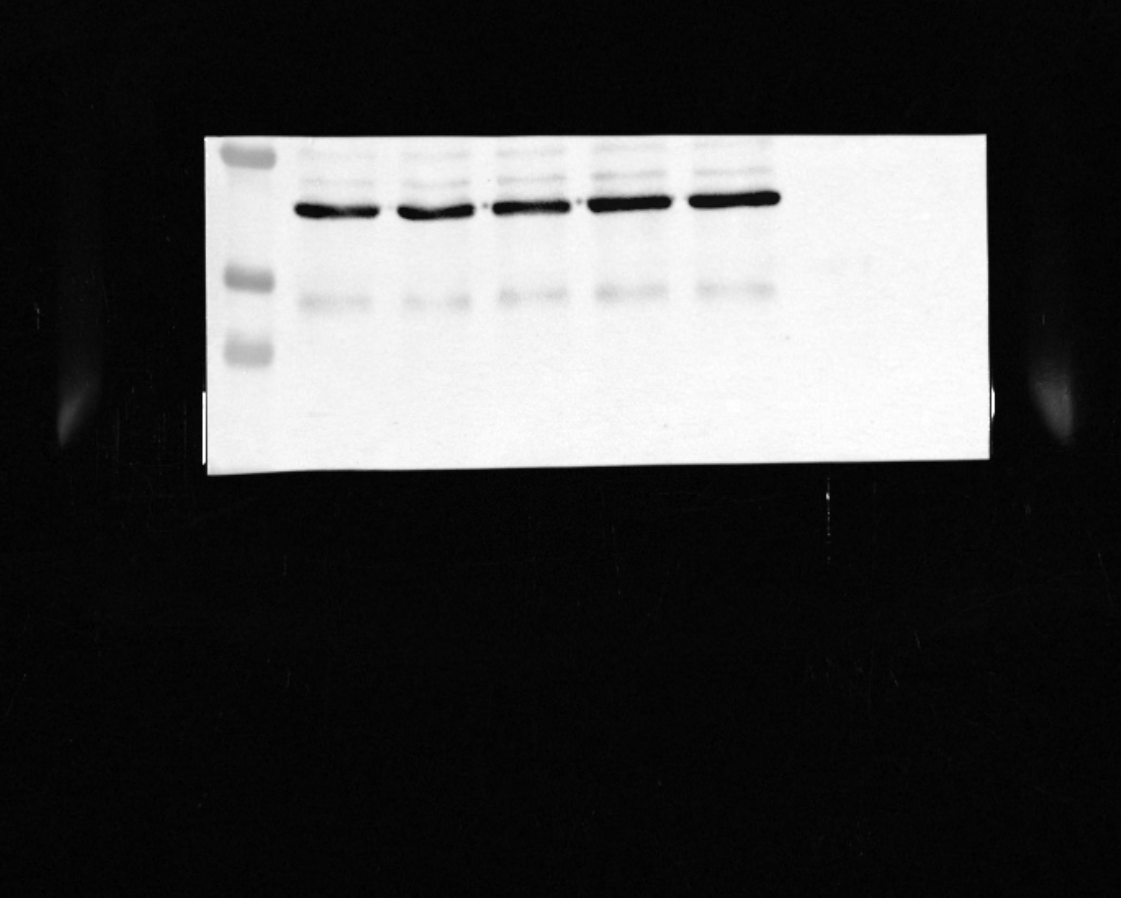

Supplement: Supplementary file 1 [file DataSheet3.zip › Capan2_B-catenin/TIF/B-catenin 3. Actin (2019. 10. 11. Zsani Actin_4).tif]

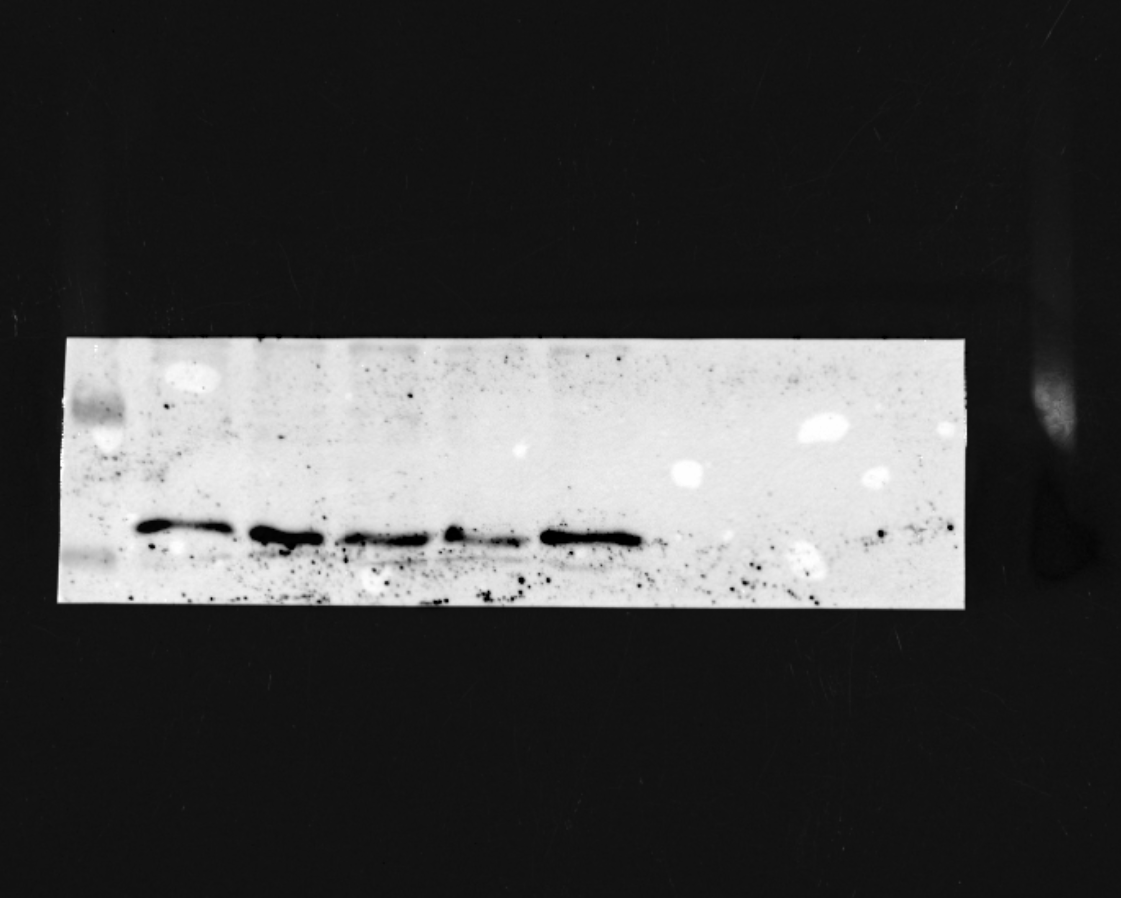

Supplement: Supplementary file 1 [file DataSheet3.zip › Capan2_Claudin1/TIF/Claudi1 1. (2019. 11. 27. Capan2 Claudin_4).tif]

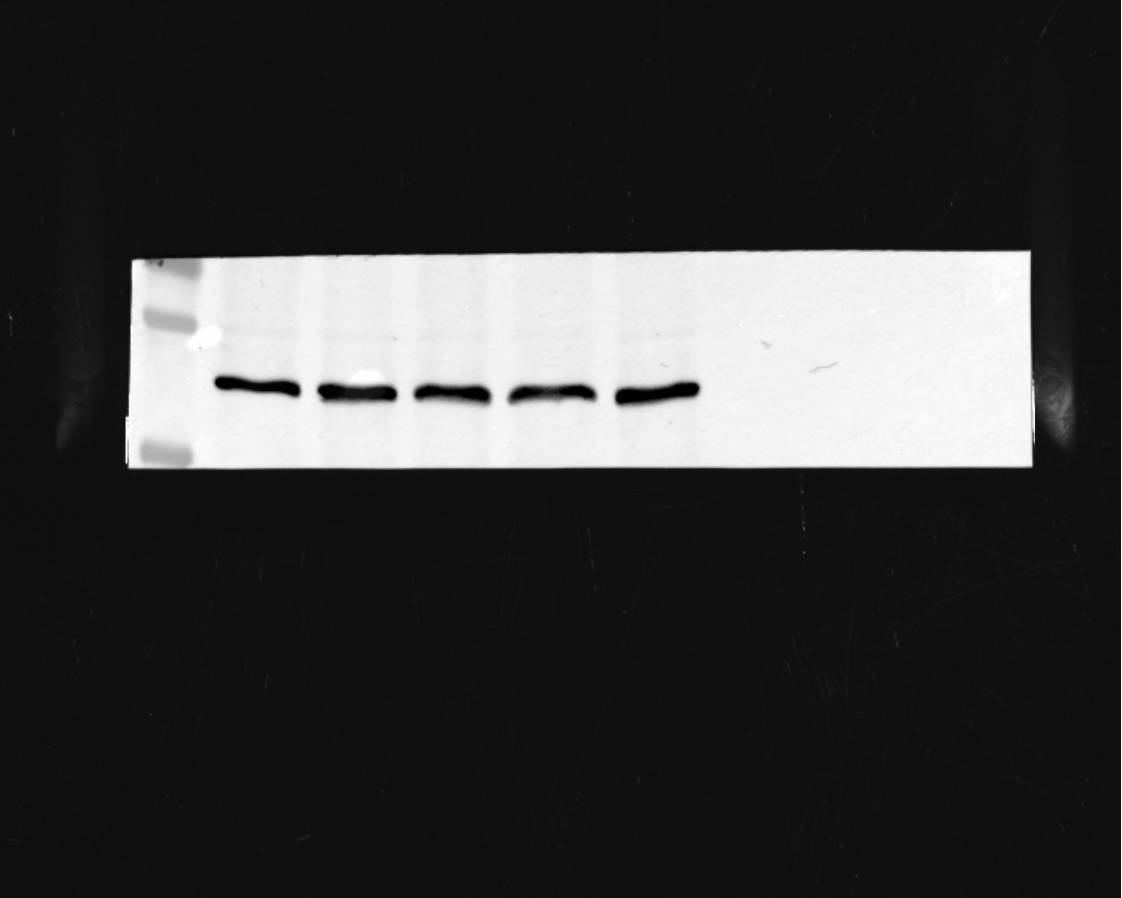

Supplement: Supplementary file 1 [file DataSheet3.zip › Capan2_Claudin1/TIF/Claudin1 1. Actin (Claudi1 1. (2019. 11. 27. Capan2 Claudin_4).tif]

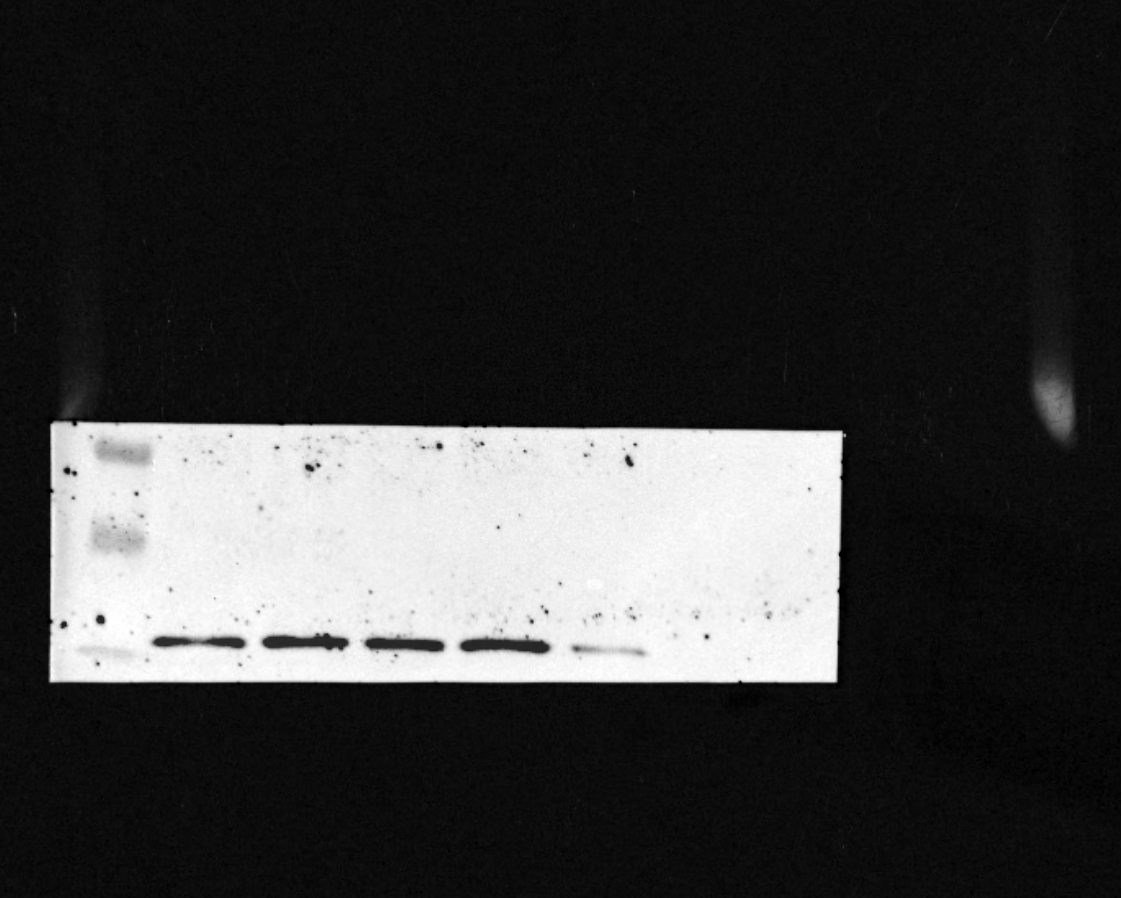

Supplement: Supplementary file 1 [file DataSheet3.zip › Capan2_Claudin1/TIF/Claudin1 2. (2019. 10. 22. Claudin1_3).tif]

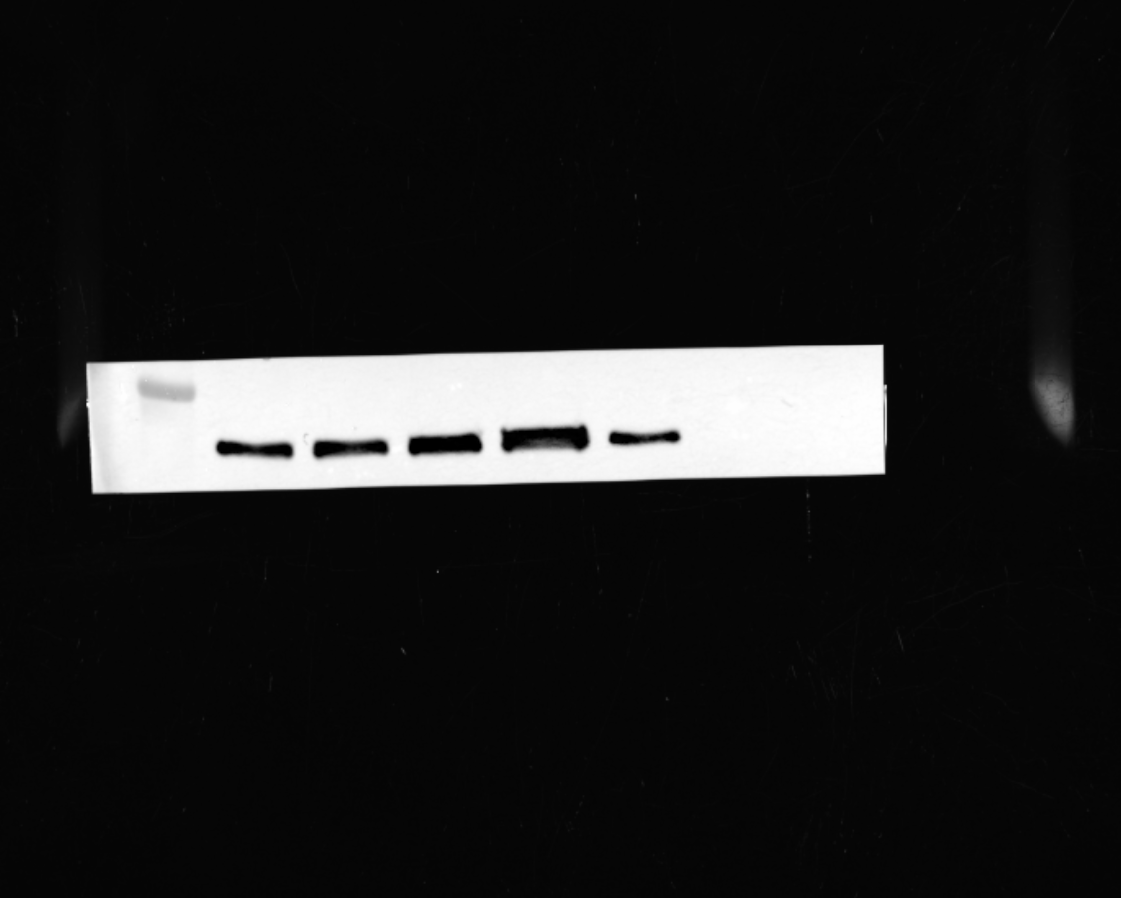

Supplement: Supplementary file 1 [file DataSheet3.zip › Capan2_Claudin1/TIF/Claudin1 2. Actin (2019. 10. 25. Aktin TCF8, CD44, Vimentin, Claudin_4).tif]

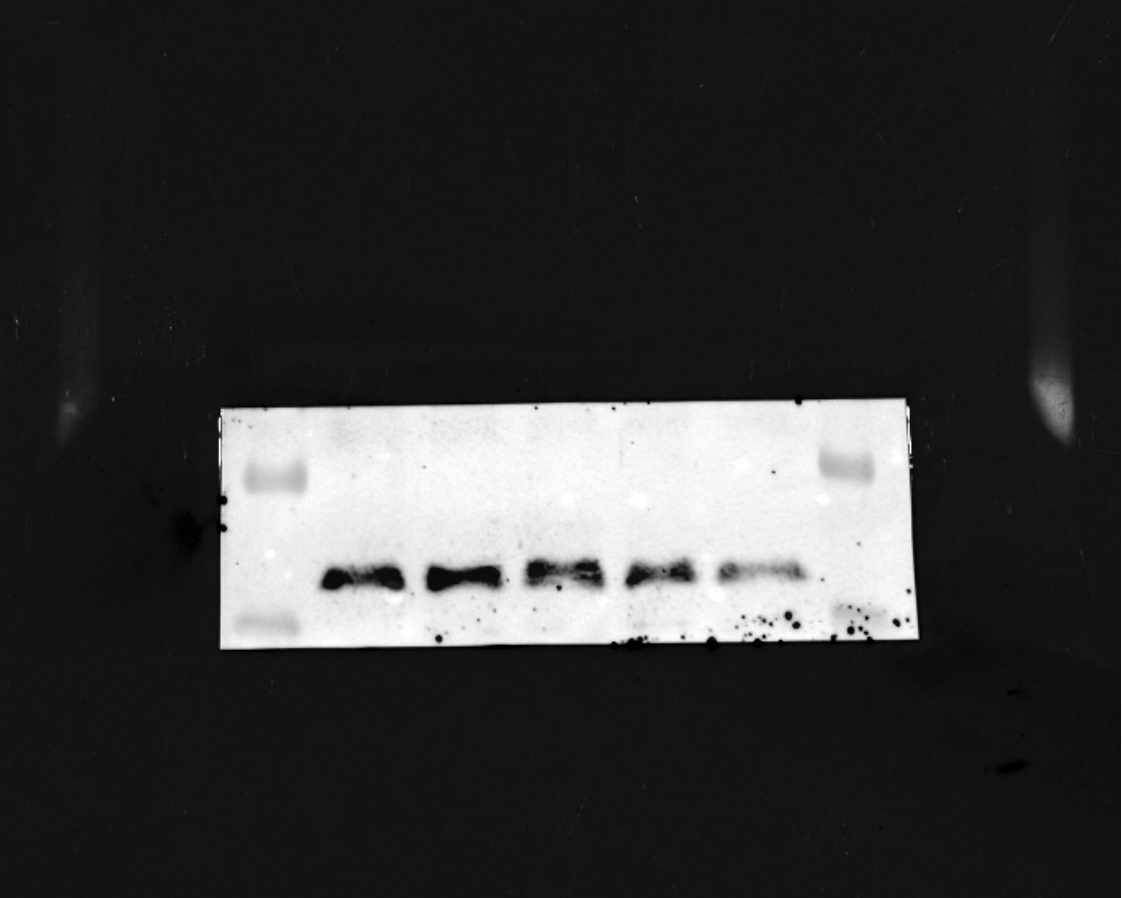

Supplement: Supplementary file 1 [file DataSheet3.zip › Capan2_Claudin1/TIF/Claudin1 3. (2019. 11. 07. Claudin_6).tif]

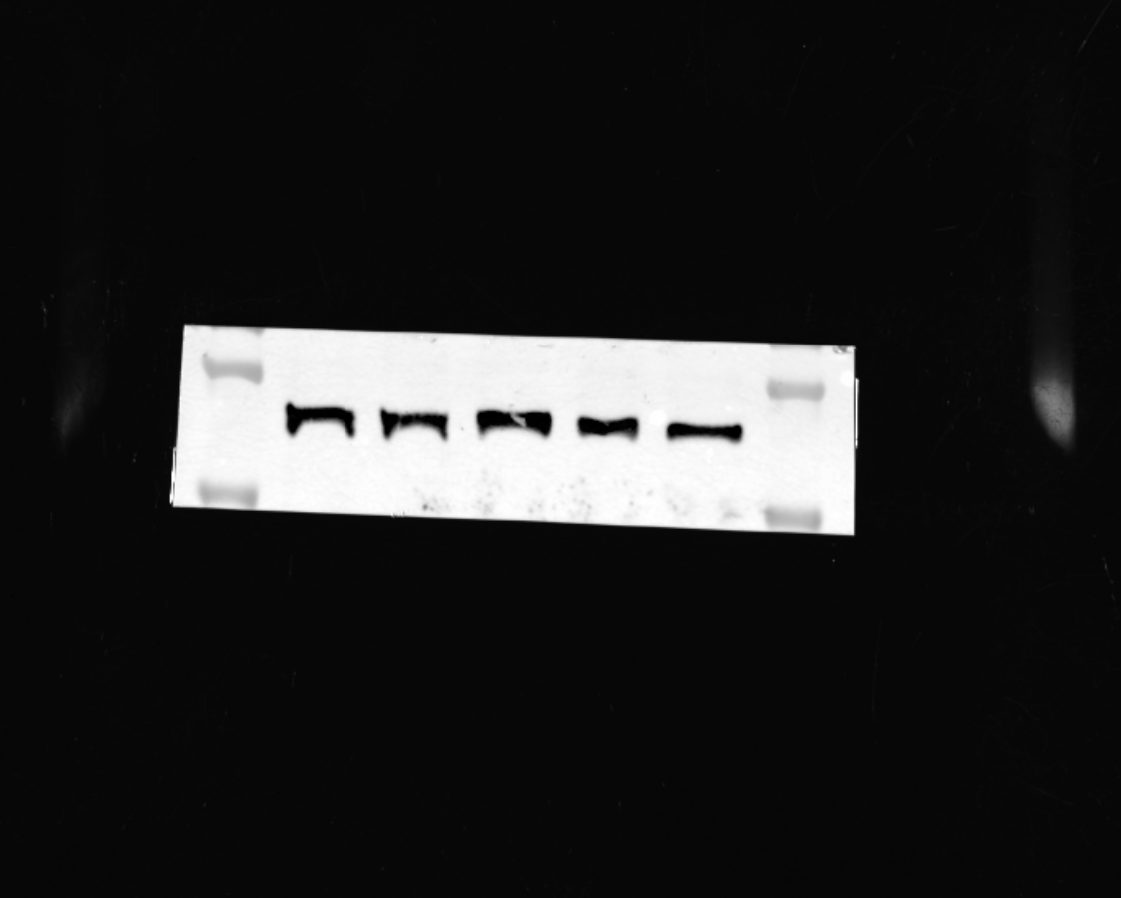

Supplement: Supplementary file 1 [file DataSheet3.zip › Capan2_Claudin1/TIF/Claudin1 3. Actin (2019. 11. 08. Aktin Zo1, CD44, Vimentin, Claudin_4).tif]

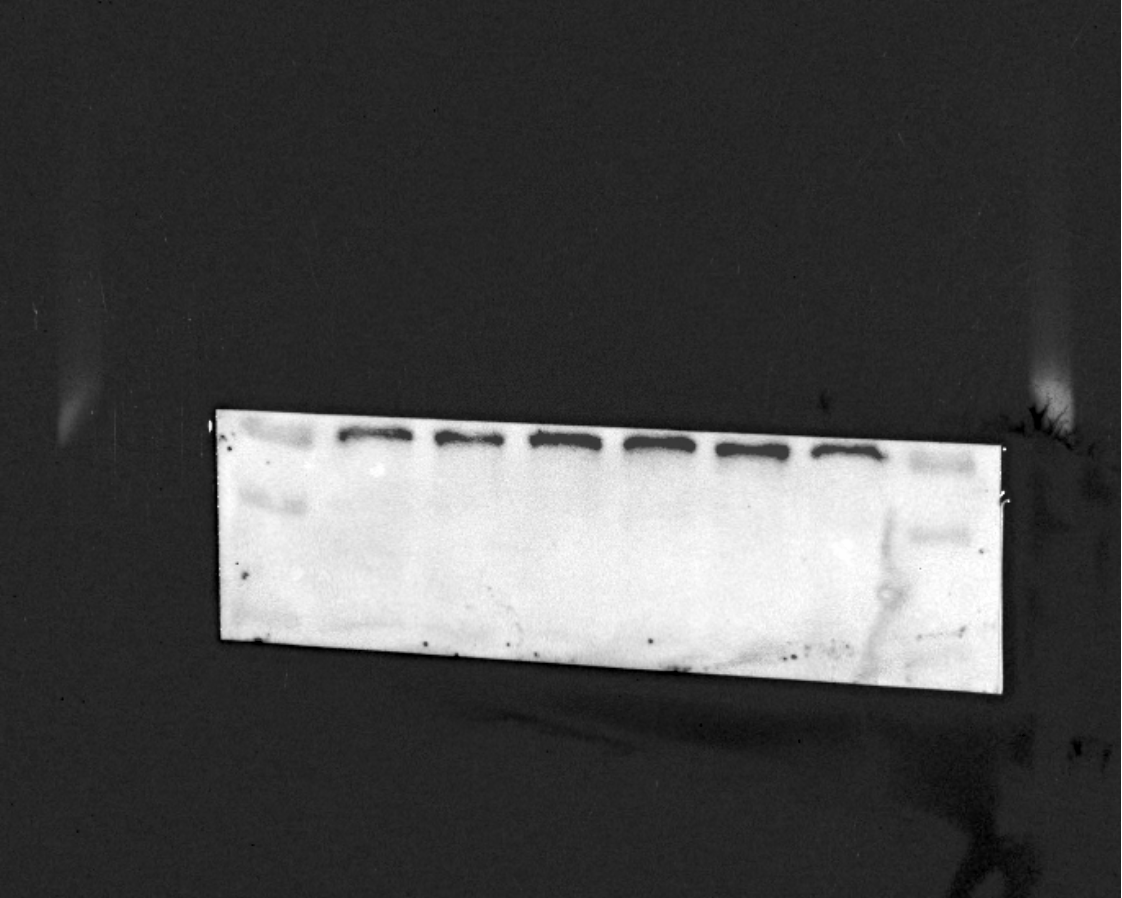

Supplement: Supplementary file 1 [file DataSheet3.zip › Capan2_Slug/TIF/Slug 1. (2020. 08. 06. Edit I. Slug_5).tif]

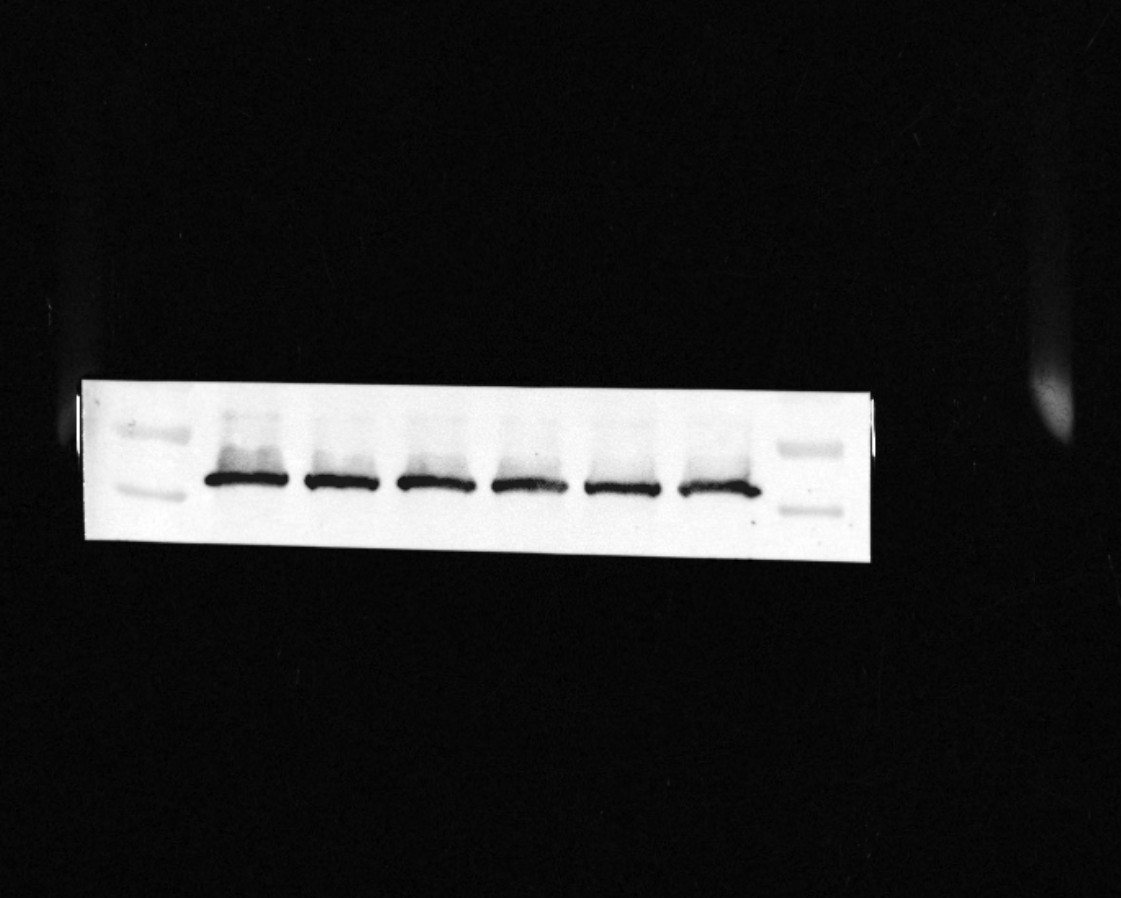

Supplement: Supplementary file 1 [file DataSheet3.zip › Capan2_Slug/TIF/Slug 1. Actin (2020. 08. 07. Edit I. Aktin Bcat, Slug_2+2020. 08. 07. Edit I. Aktin Bcat, Slug_1).tif]

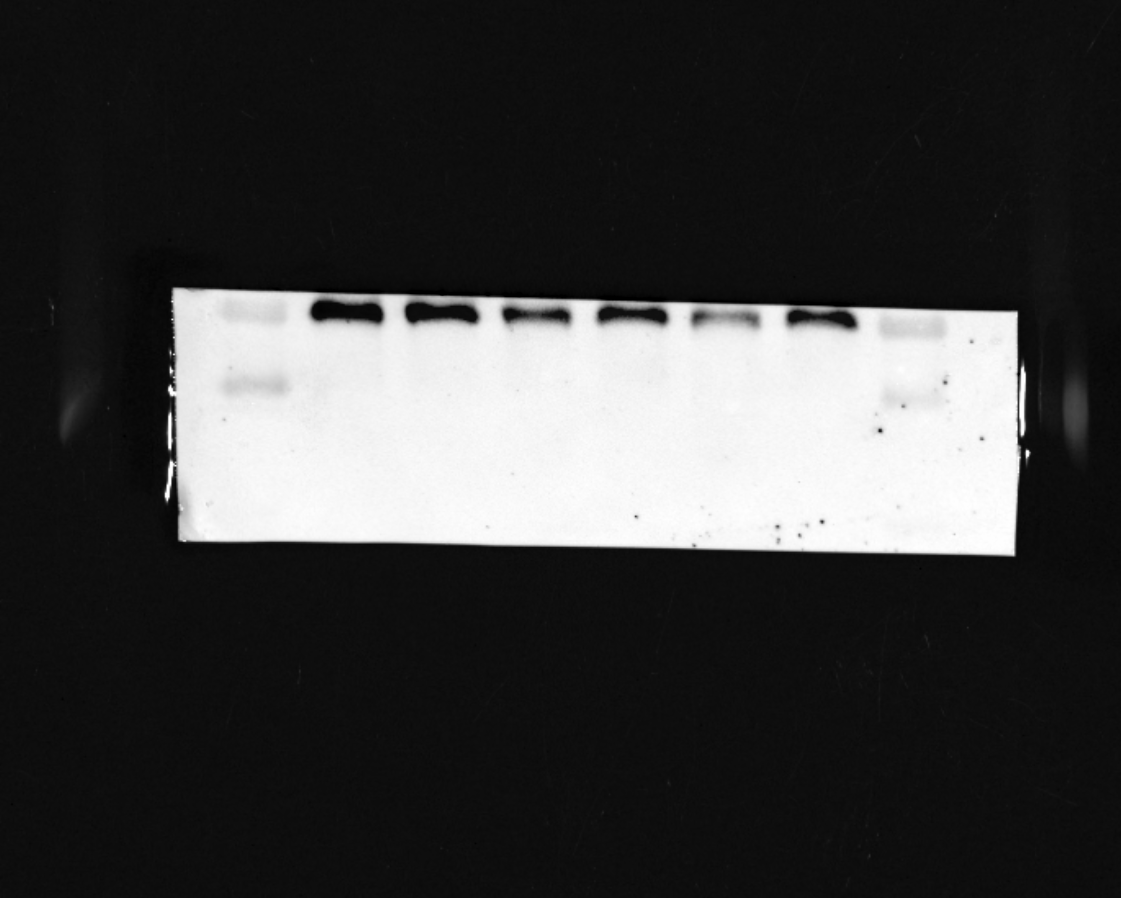

Supplement: Supplementary file 1 [file DataSheet3.zip › Capan2_Slug/TIF/Slug 2. (2020. 08. 07. Edit II. Slug_6).tif]

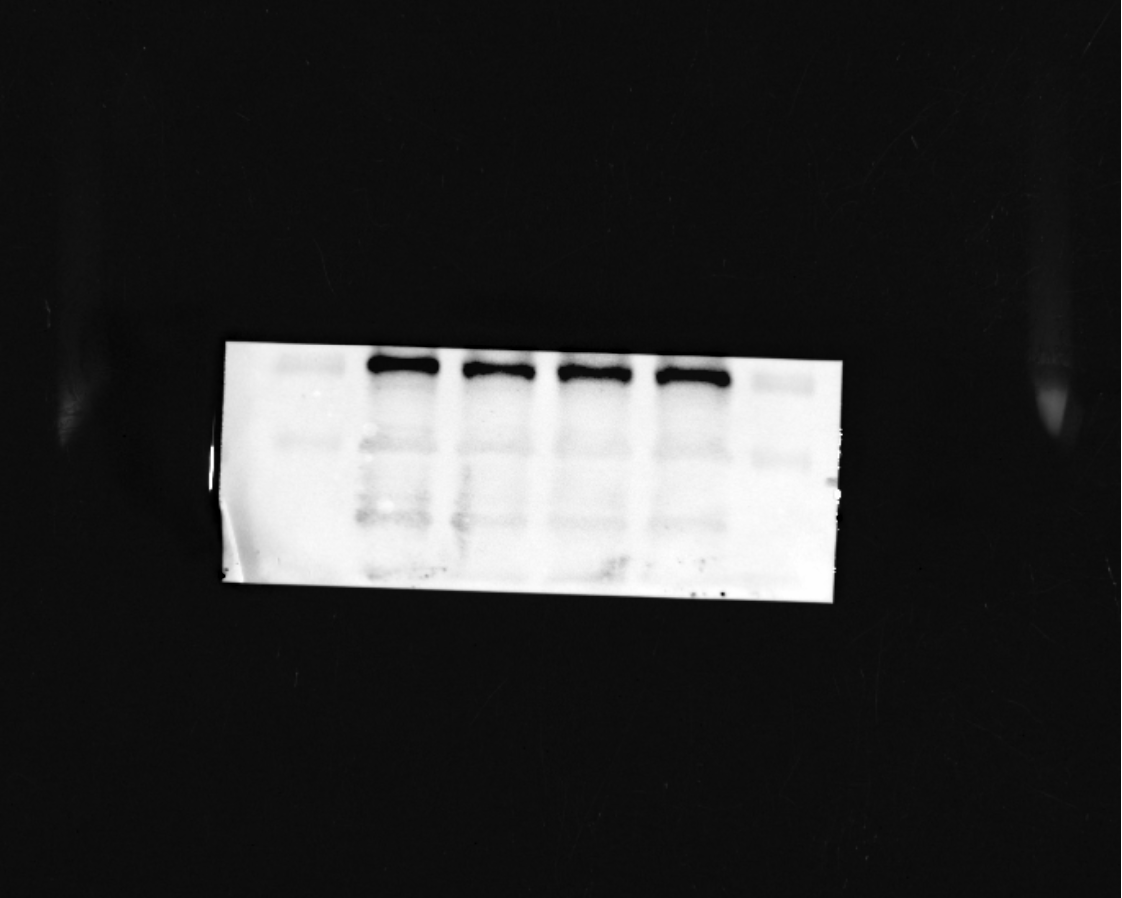

Supplement: Supplementary file 1 [file DataSheet3.zip › Capan2_Slug/TIF/Slug 3. (2020. 08. 07. X32 Slug_1+2020. 08. 07. X32 Slug_2).tif]

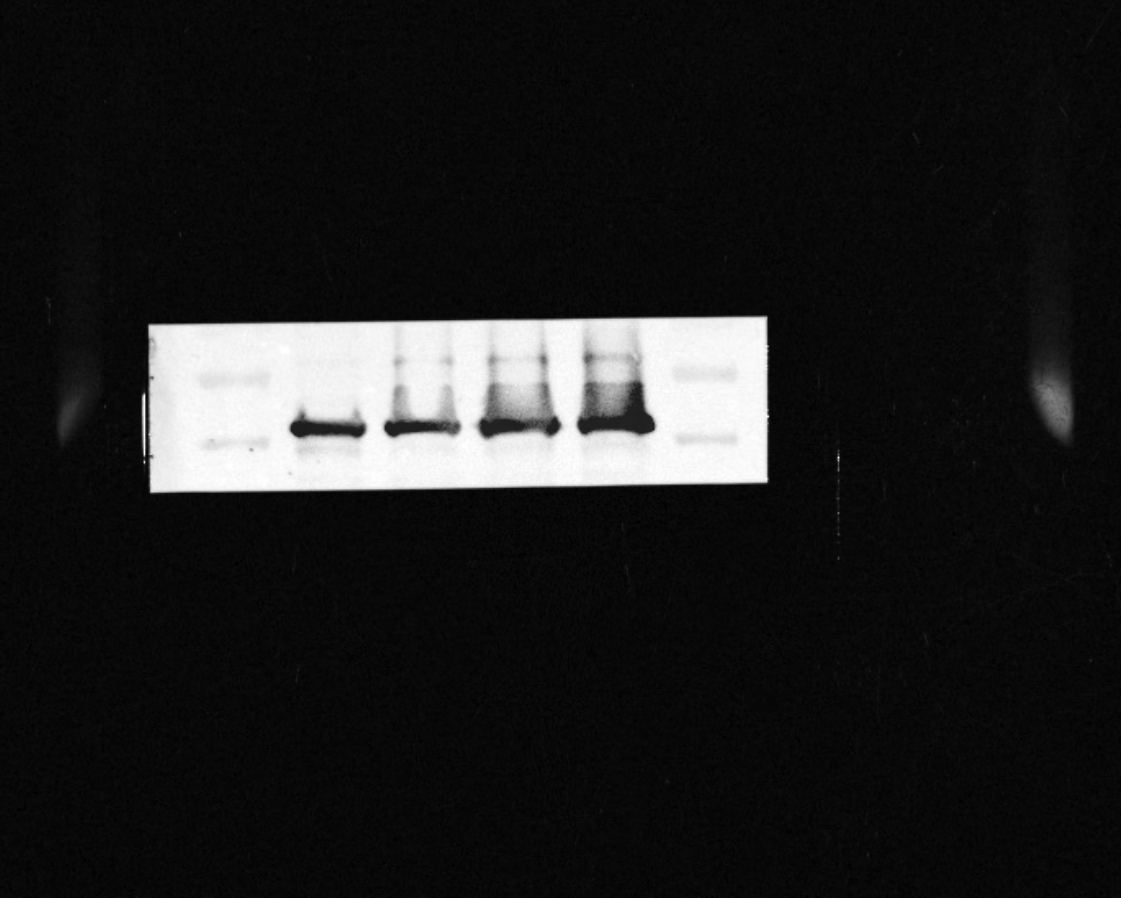

Supplement: Supplementary file 1 [file DataSheet3.zip › Capan2_Slug/TIF/Slug 3. Actin (2020. 08. 07. X32 Aktin Bcat, Slug_3+2020. 08. 07. X32 Aktin Bcat, Slug_1).tif]

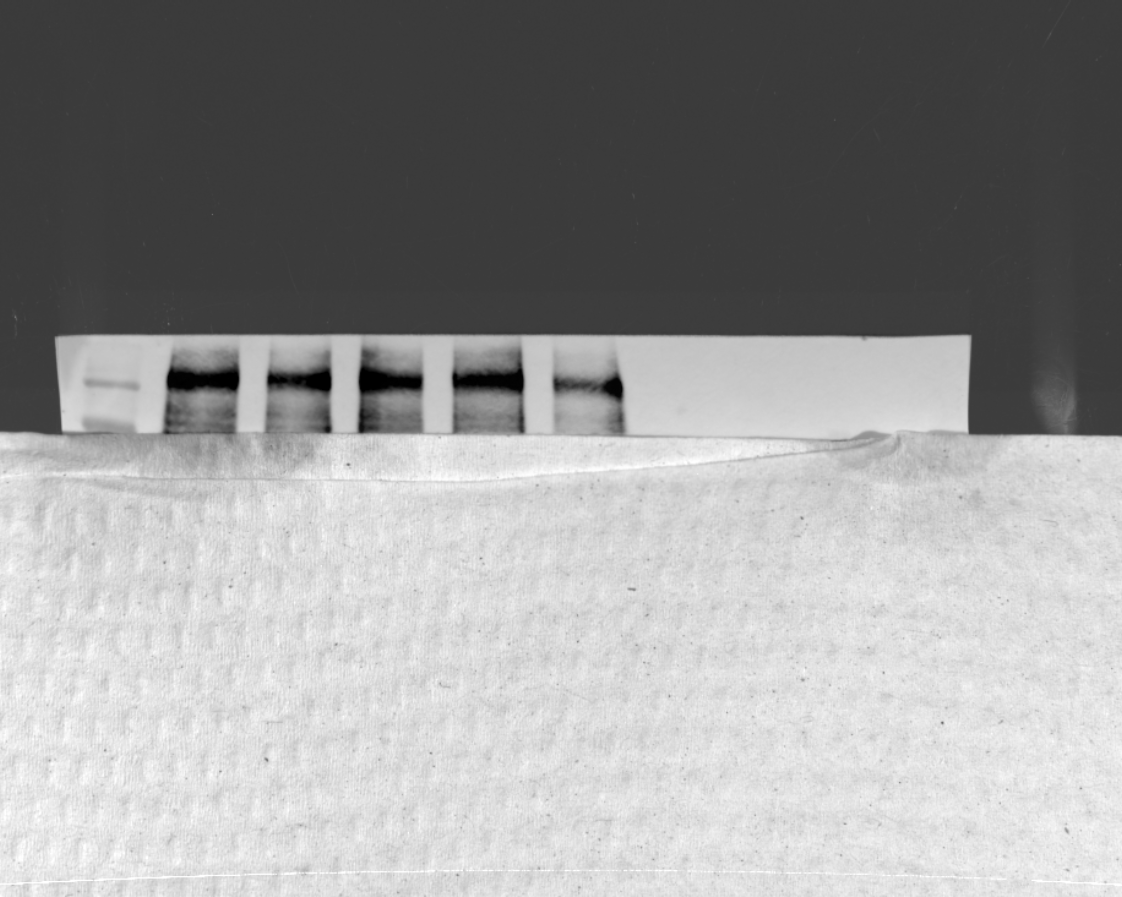

Supplement: Supplementary file 1 [file DataSheet3.zip › Capan2_ZO1/TIF/ZO1 1. (2019. 11. 28. Capan2 ZO1_5).tif]

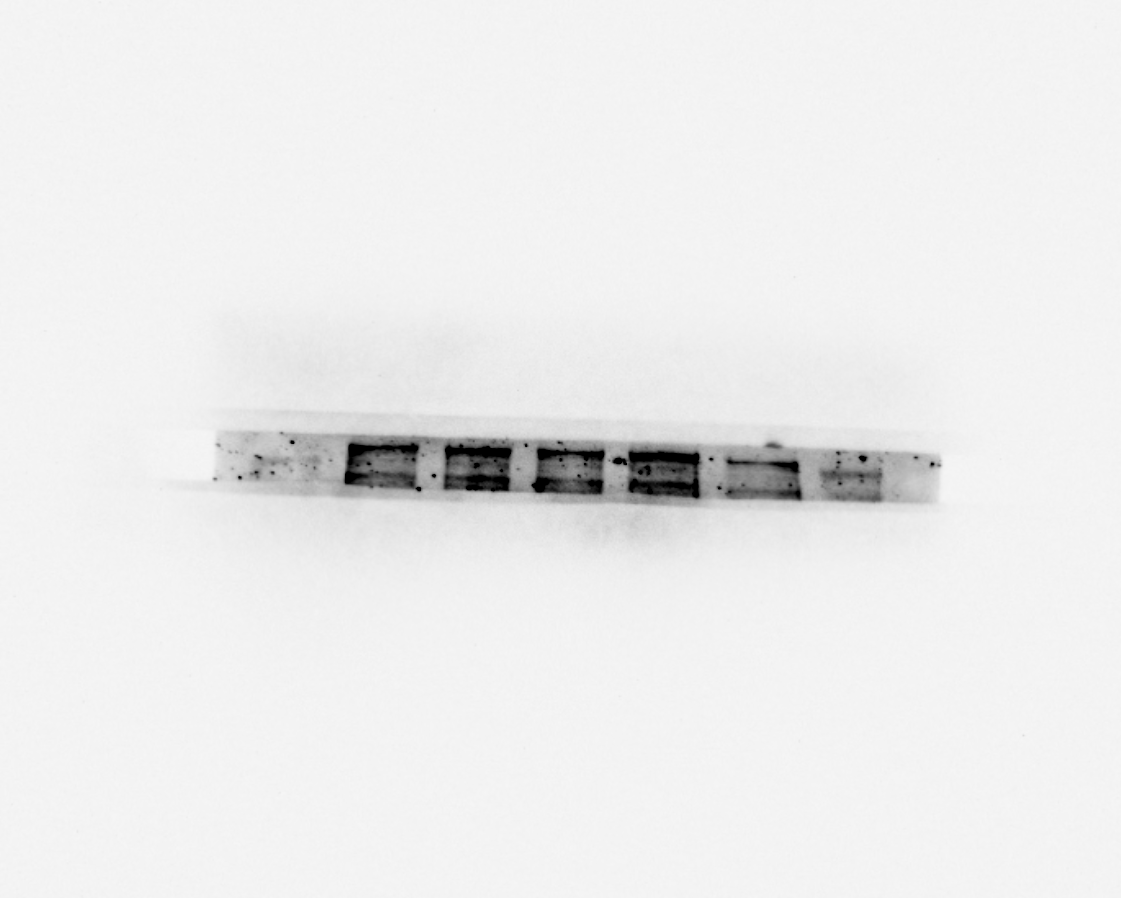

Supplement: Supplementary file 1 [file DataSheet3.zip › Capan2_ZO1/TIF/ZO1 2. (2019. 10. 22. ZO1 FEMTO_2).tif]

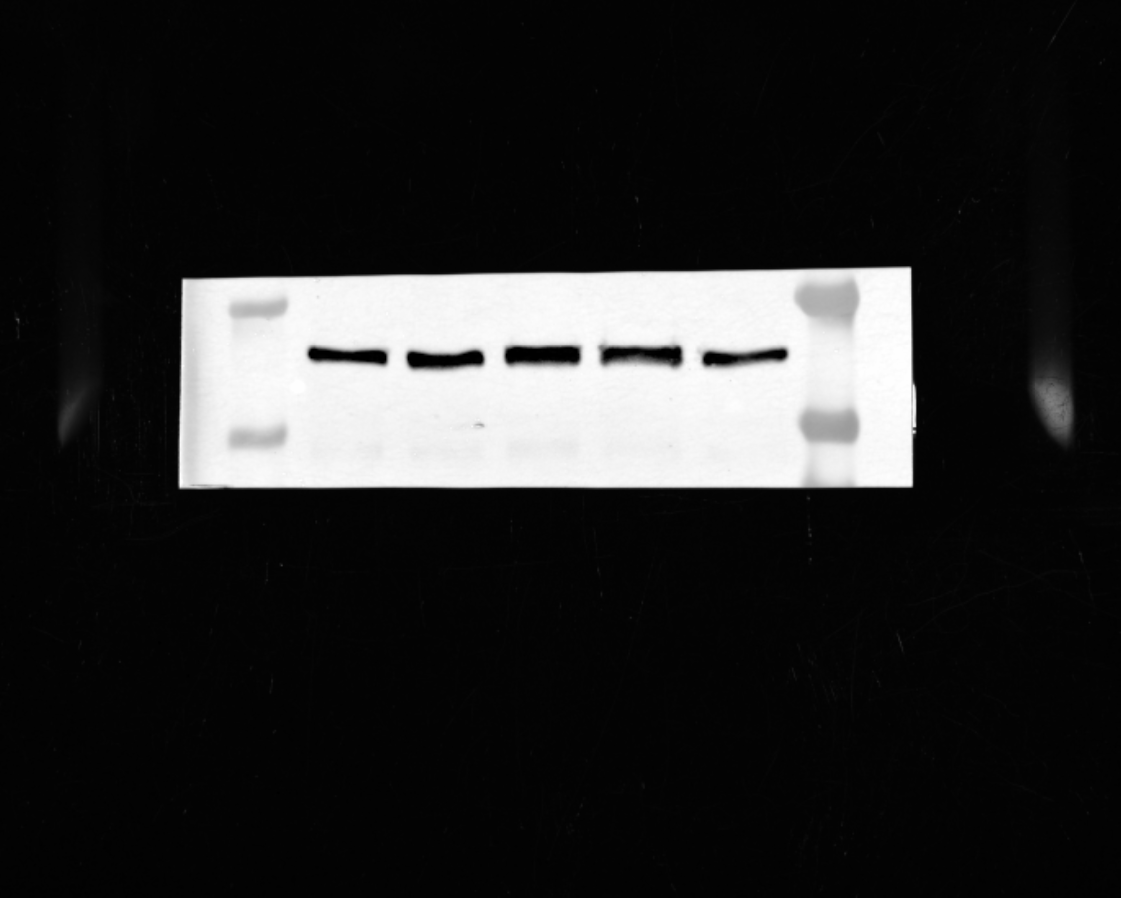

Supplement: Supplementary file 1 [file DataSheet3.zip › Capan2_ZO1/TIF/ZO1 2. Actin (2019. 10. 25. Aktin Ecad, ZO1, Bcat, Snai1_3).tif]

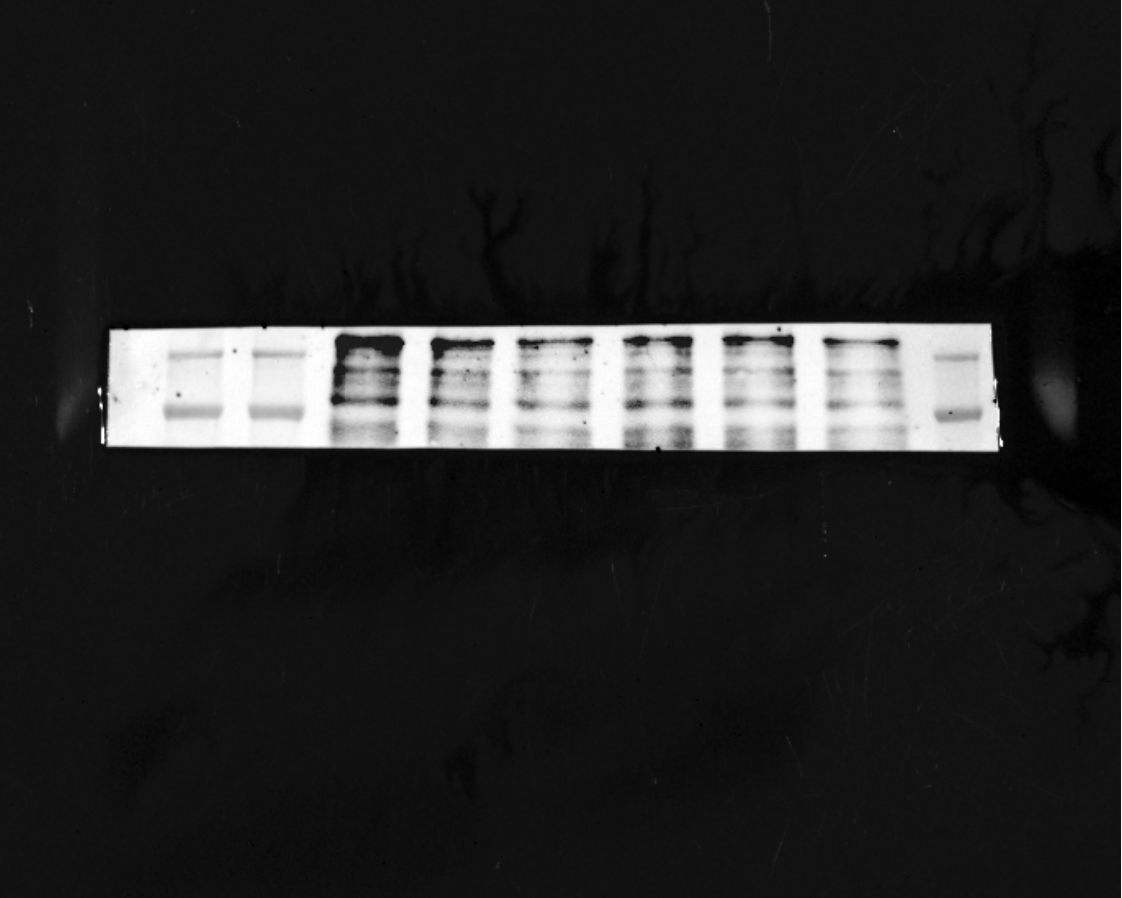

Supplement: Supplementary file 1 [file DataSheet3.zip › Capan2_ZO1/TIF/ZO1 3. (2022. 11. 09. II. ZO1_3).tif]

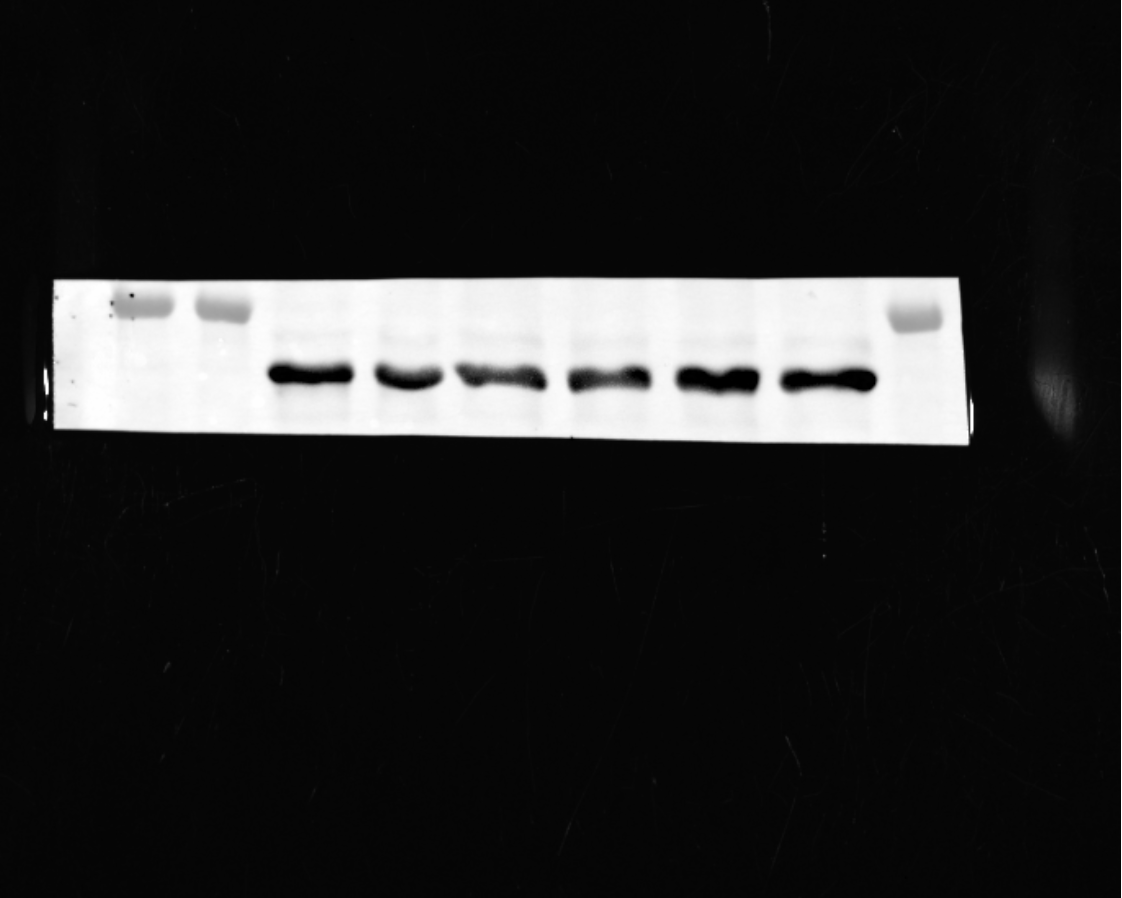

Supplement: Supplementary file 1 [file DataSheet3.zip › Capan2_ZO1/TIF/ZO1 3. Actin (2022. 11. 09. II. Aktin_3).tif]

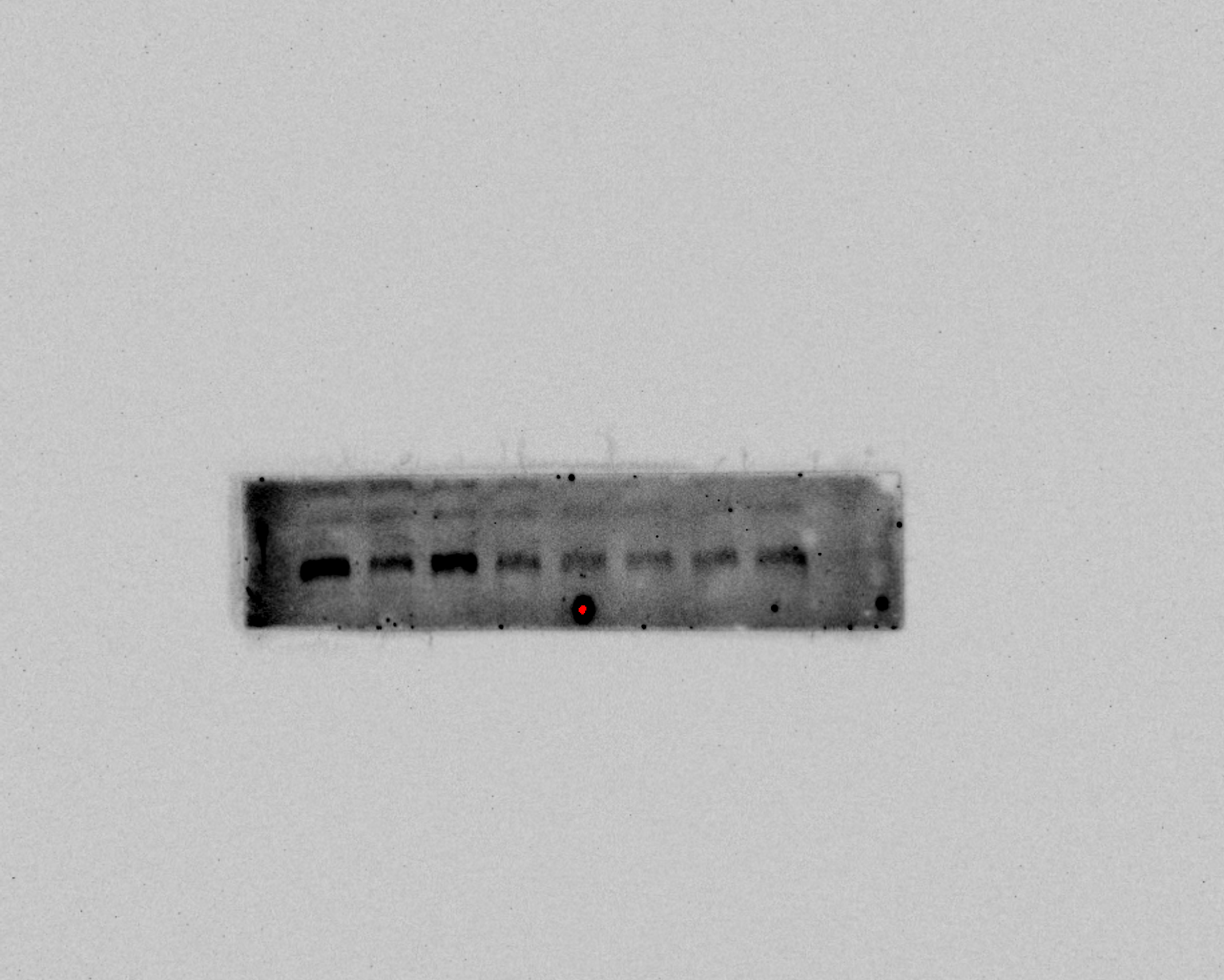

Supplement: Supplementary file 3 [file DataSheet4.zip › BxPC3_Snail/TIF/Snail 1. (kiértékelt_snai_1207).tif]

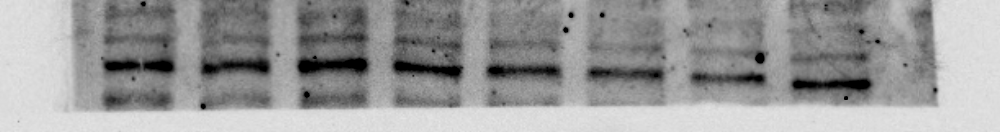

Supplement: Supplementary file 3 [file DataSheet4.zip › BxPC3_Snail/TIF/Snail 1. Actin (kiértékelt_aktin_snai_nrf2_1207).tif]

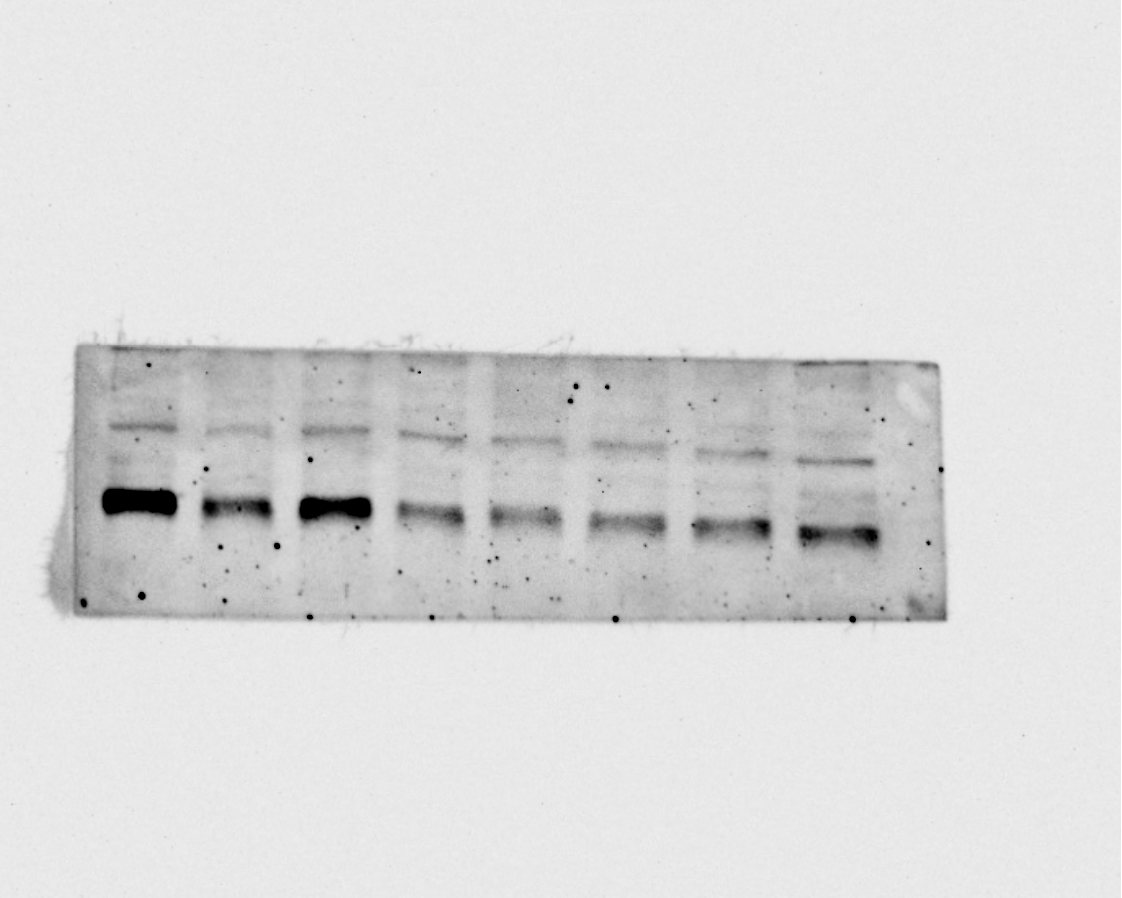

Supplement: Supplementary file 3 [file DataSheet4.zip › BxPC3_Snail/TIF/Snail 2. (kiértékelt_snai_1213).tif]

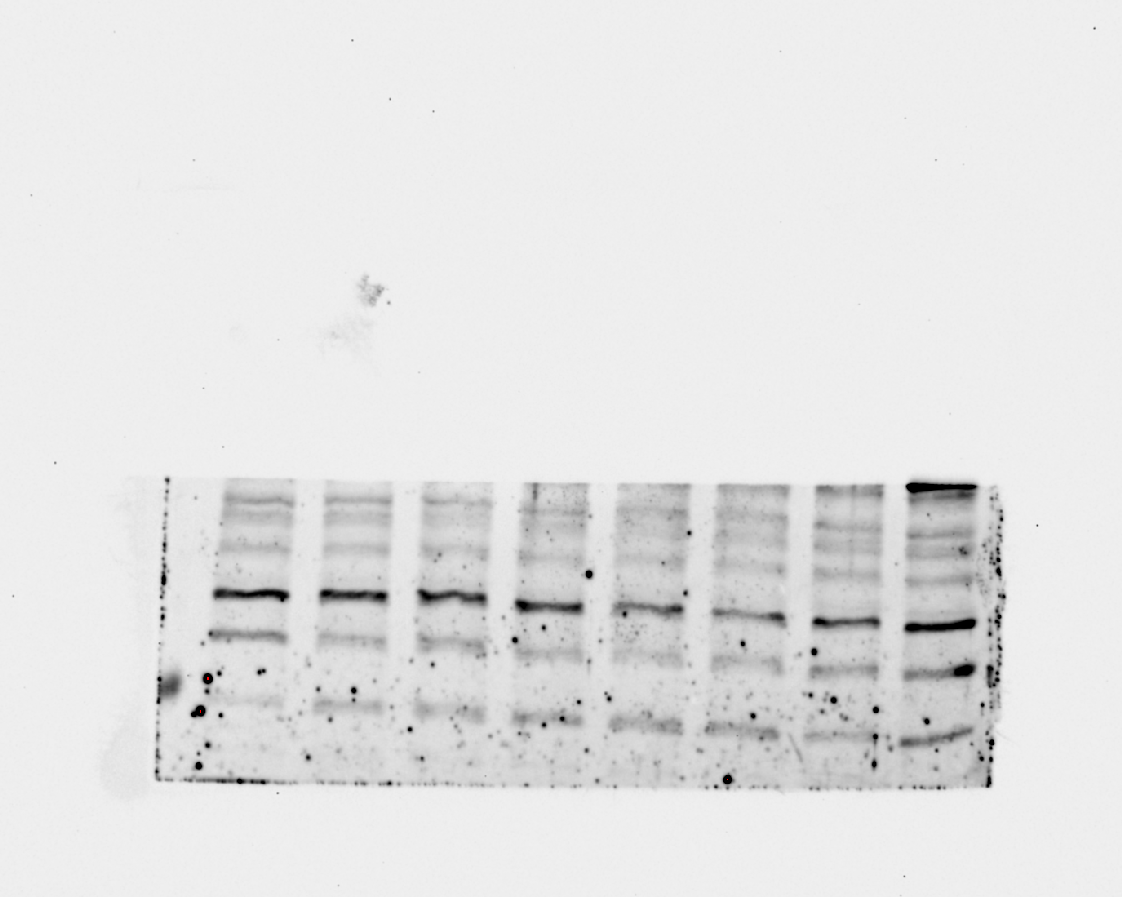

Supplement: Supplementary file 3 [file DataSheet4.zip › BxPC3_Snail/TIF/Snail 2. Actin (kiértékelt_aktin_snai_56_1213).tif]

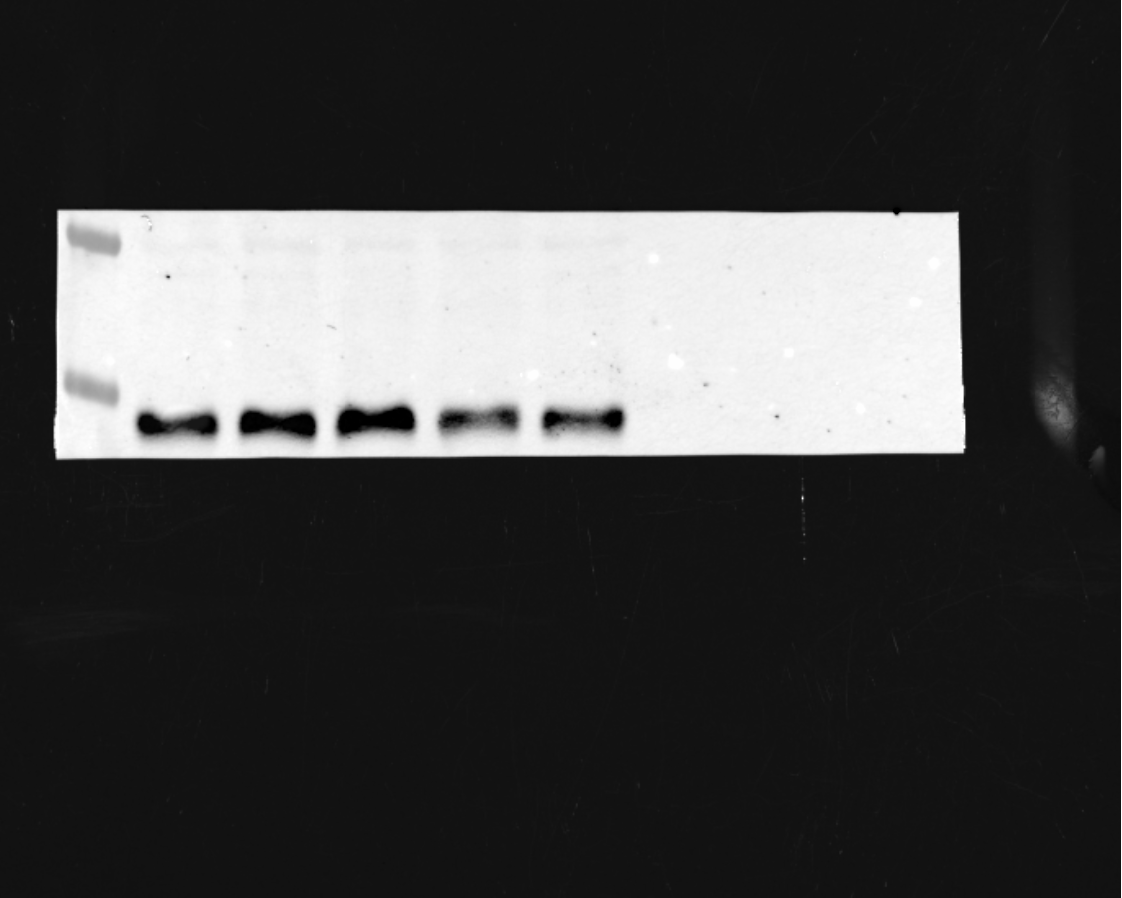

Supplement: Supplementary file 3 [file DataSheet4.zip › Capan2_Snail/TIF/Snail 1. (2019. 11. 27. Capan2 Snai1_4).tif]

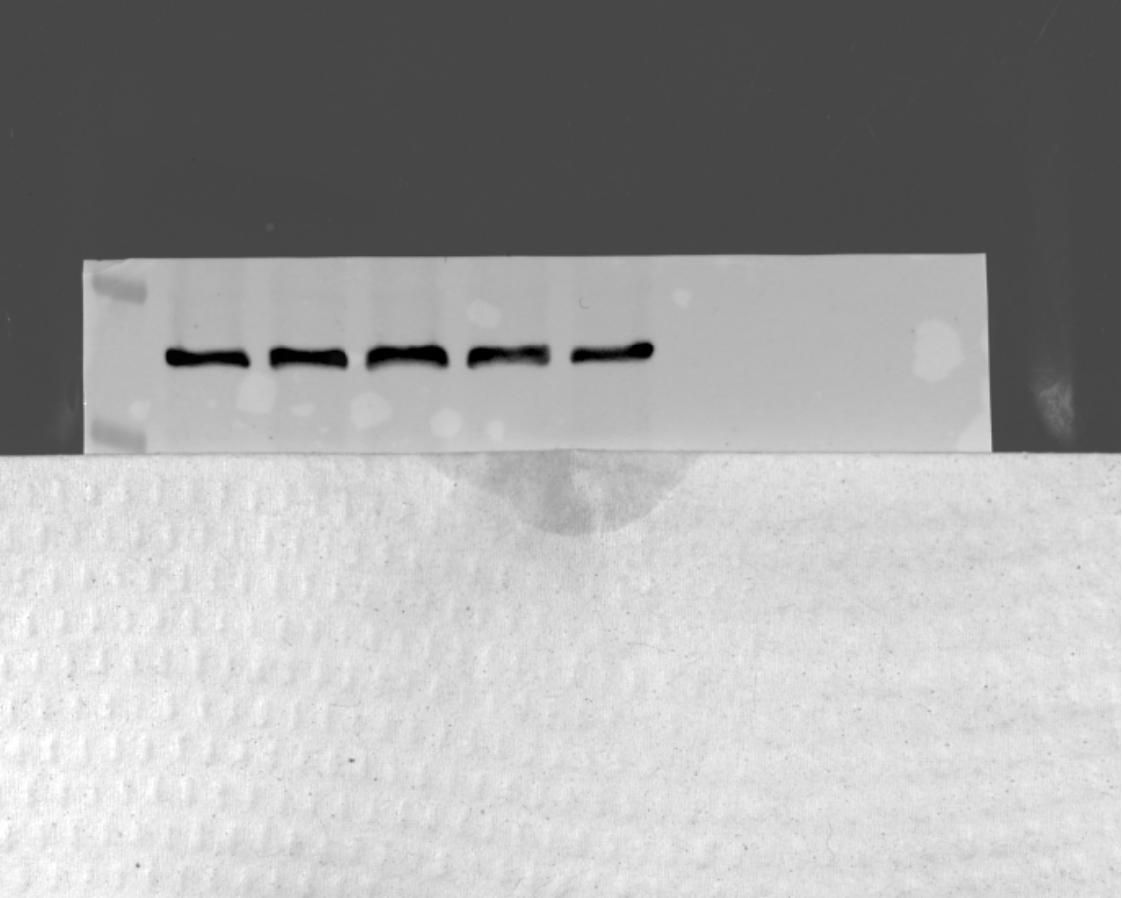

Supplement: Supplementary file 3 [file DataSheet4.zip › Capan2_Snail/TIF/Snail 1. Actin (2019. 11. 28. Aktin E-cadherin, B-cateini, Snai1_4).tif]

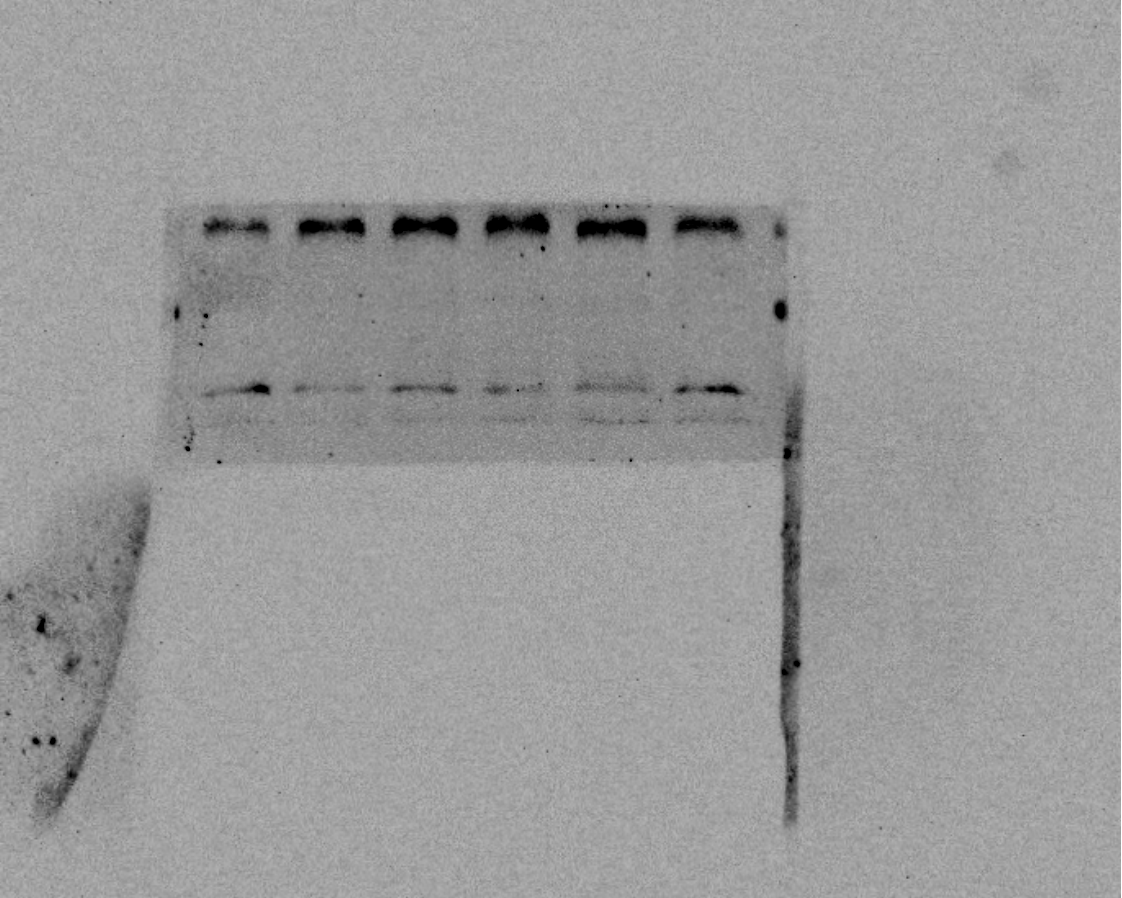

Supplement: Supplementary file 3 [file DataSheet4.zip › Capan2_Snail/TIF/Snail 2. (2020. 08. 07. Edit I. Snai_2).tif]

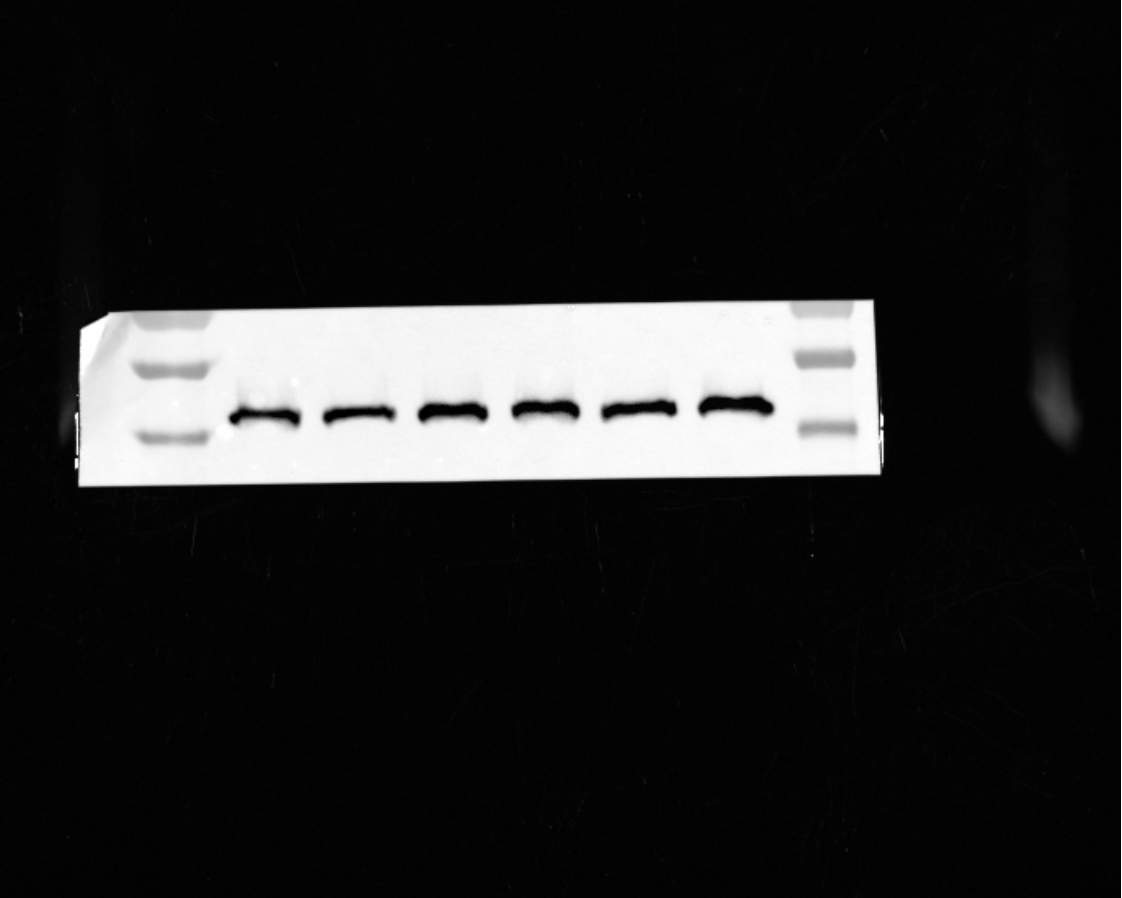

Supplement: Supplementary file 3 [file DataSheet4.zip › Capan2_Snail/TIF/Snail 2. Actin (2020. 08. 07. Edit I. Aktin ZO1 Vimentin Snai_1+2020. 08. 07. Edit I. Aktin ZO1 Vimentin Snai_2).tif]

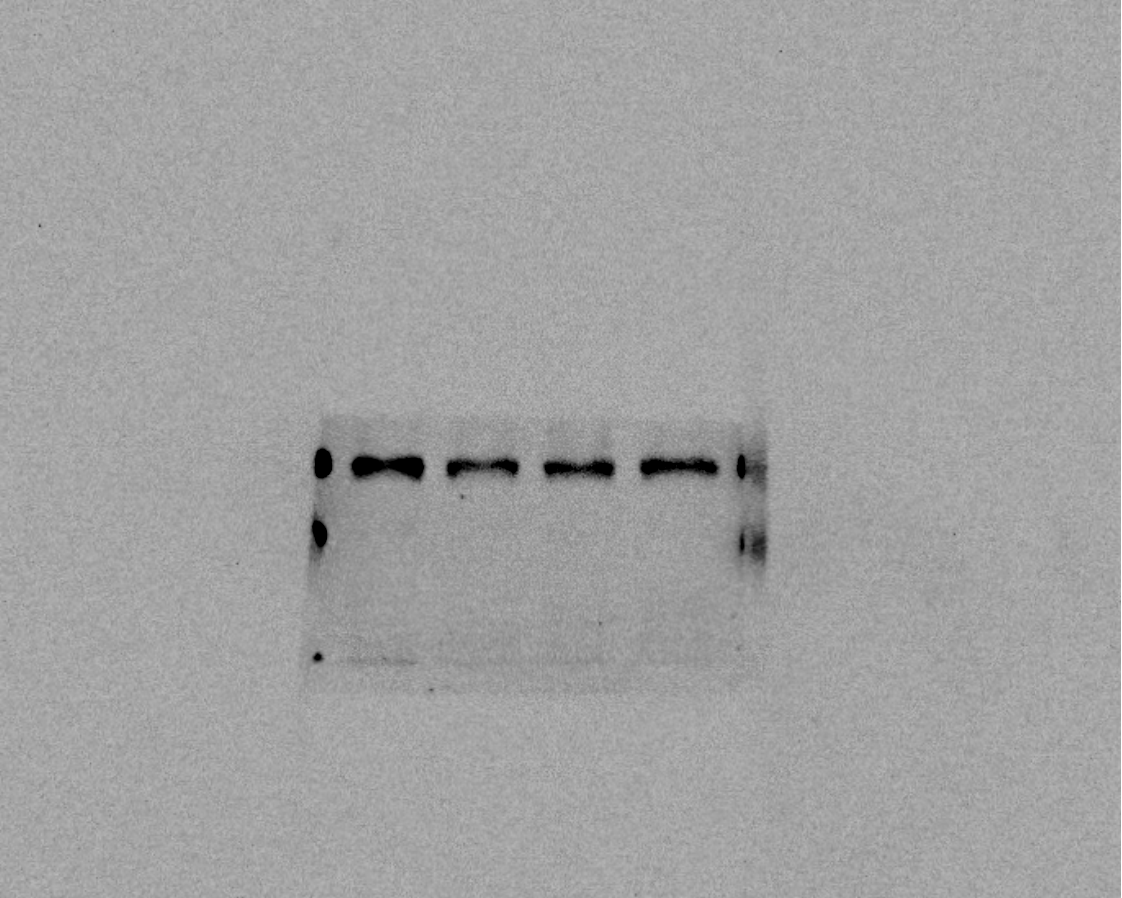

Supplement: Supplementary file 3 [file DataSheet4.zip › Capan2_Snail/TIF/Snail 3. (2020. 08. 07. X32 Snai_2).tif]

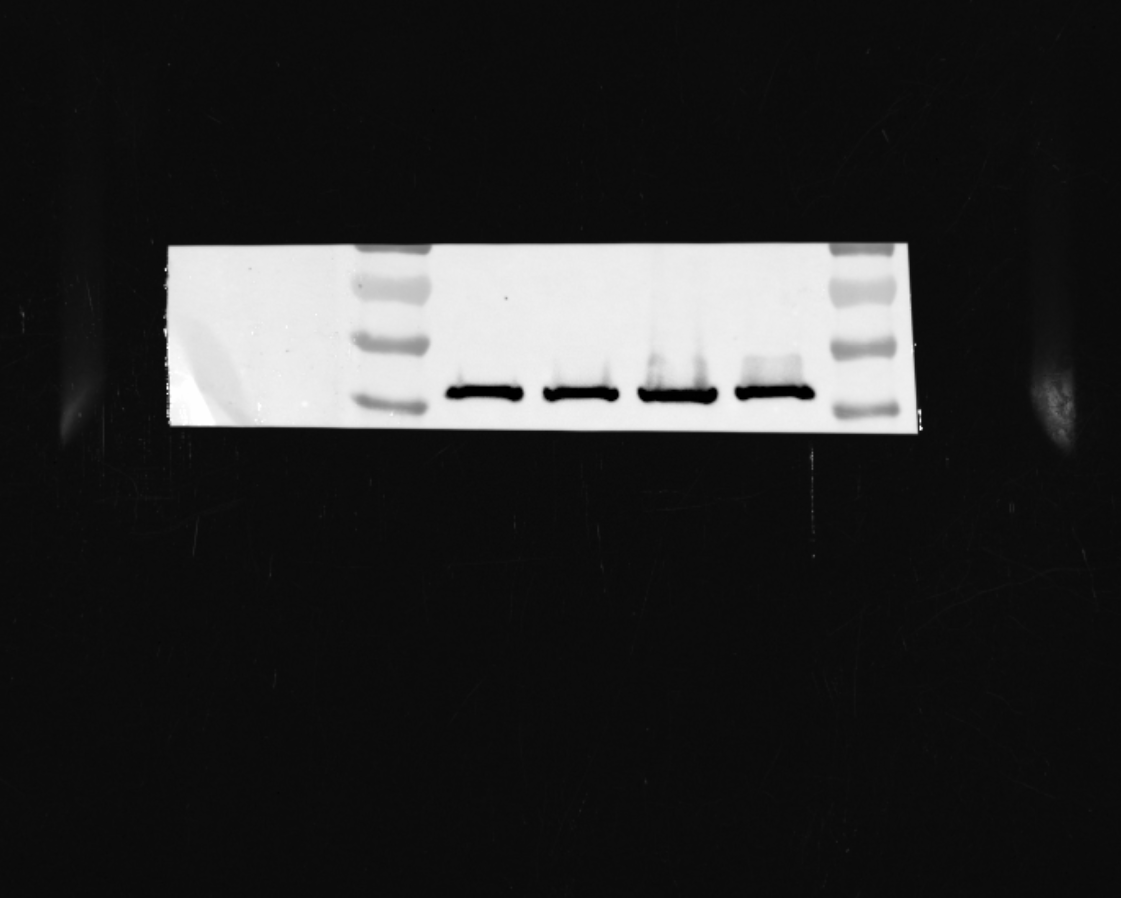

Supplement: Supplementary file 3 [file DataSheet4.zip › Capan2_Snail/TIF/Snail 3. Actin (2020. 08. 07. X32 Aktin Vimentin ZO1 Snai_1+2020. 08. 07. X32 Aktin Vimentin ZO1 Snai_2).tif]

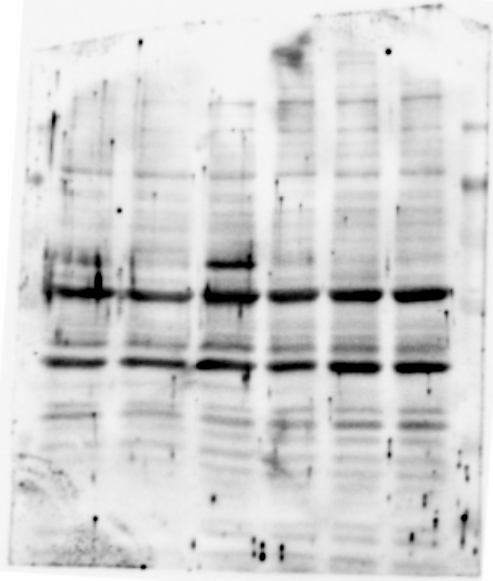

Supplement: Supplementary file 3 [file DataSheet4.zip › Fibroblast_4HNE/Fibro 4HNE.tif]

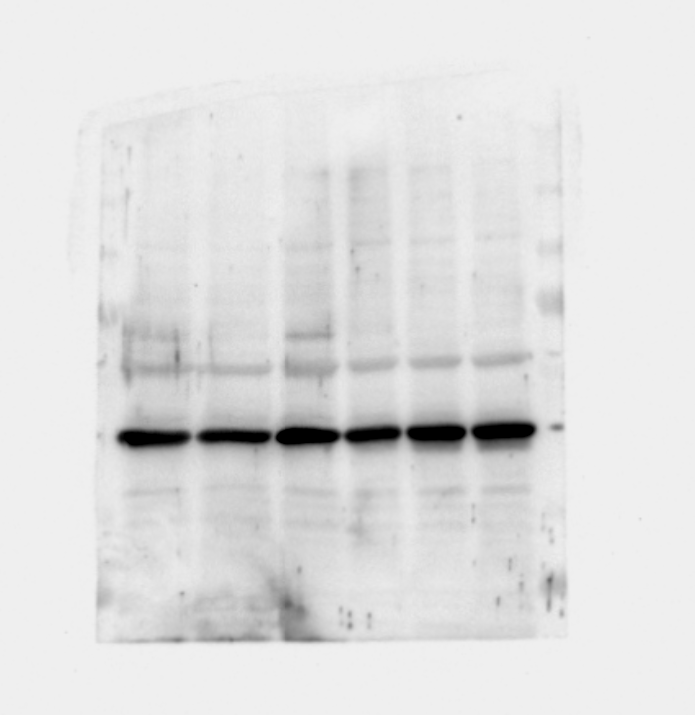

Supplement: Supplementary file 3 [file DataSheet4.zip › Fibroblast_4HNE/Fibro Aktin.tif]

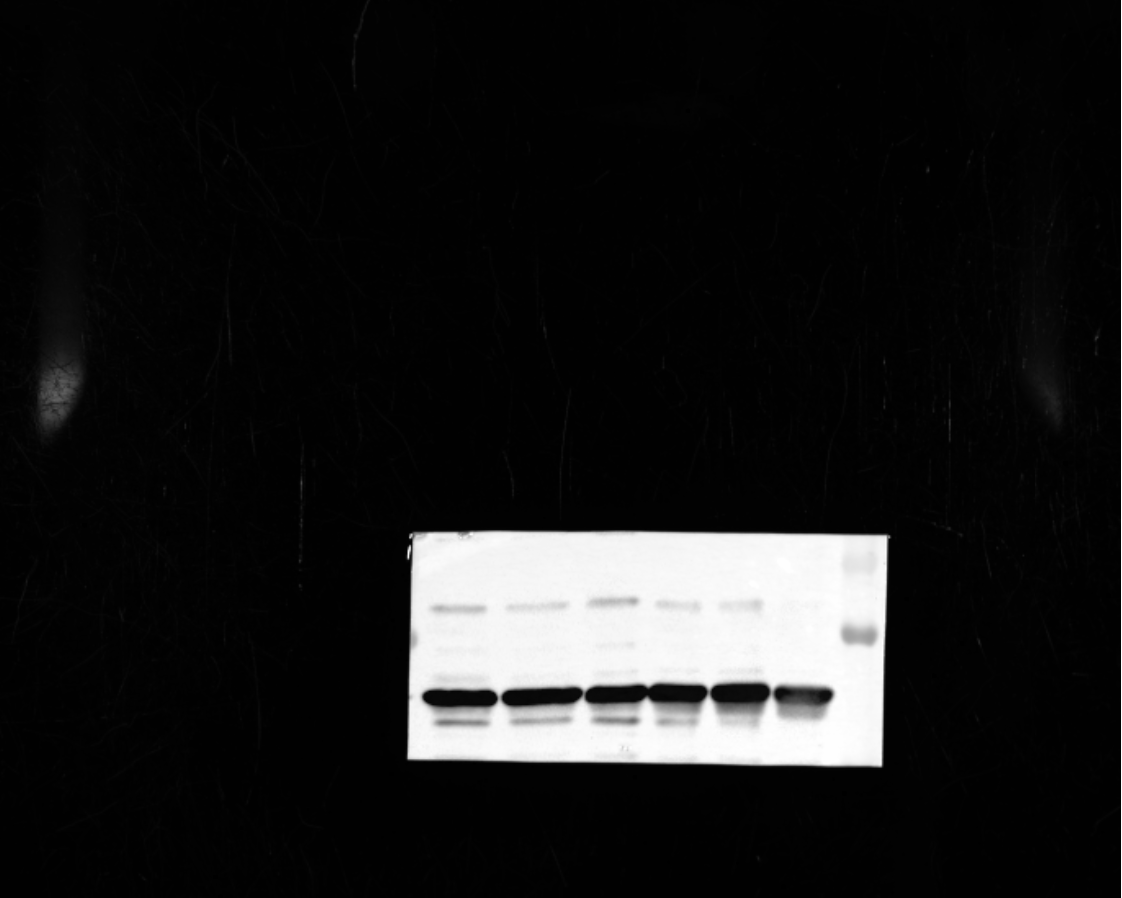

Supplement: Supplementary file 3 [file DataSheet4.zip › Fibroblast_Nrf2/Fibro Aktin.tif]

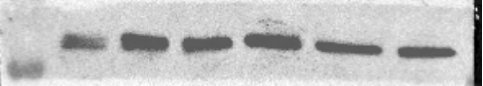

Supplement: Supplementary file 3 [file DataSheet4.zip › Fibroblast_Nrf2/Fibro Nrf2.tif]

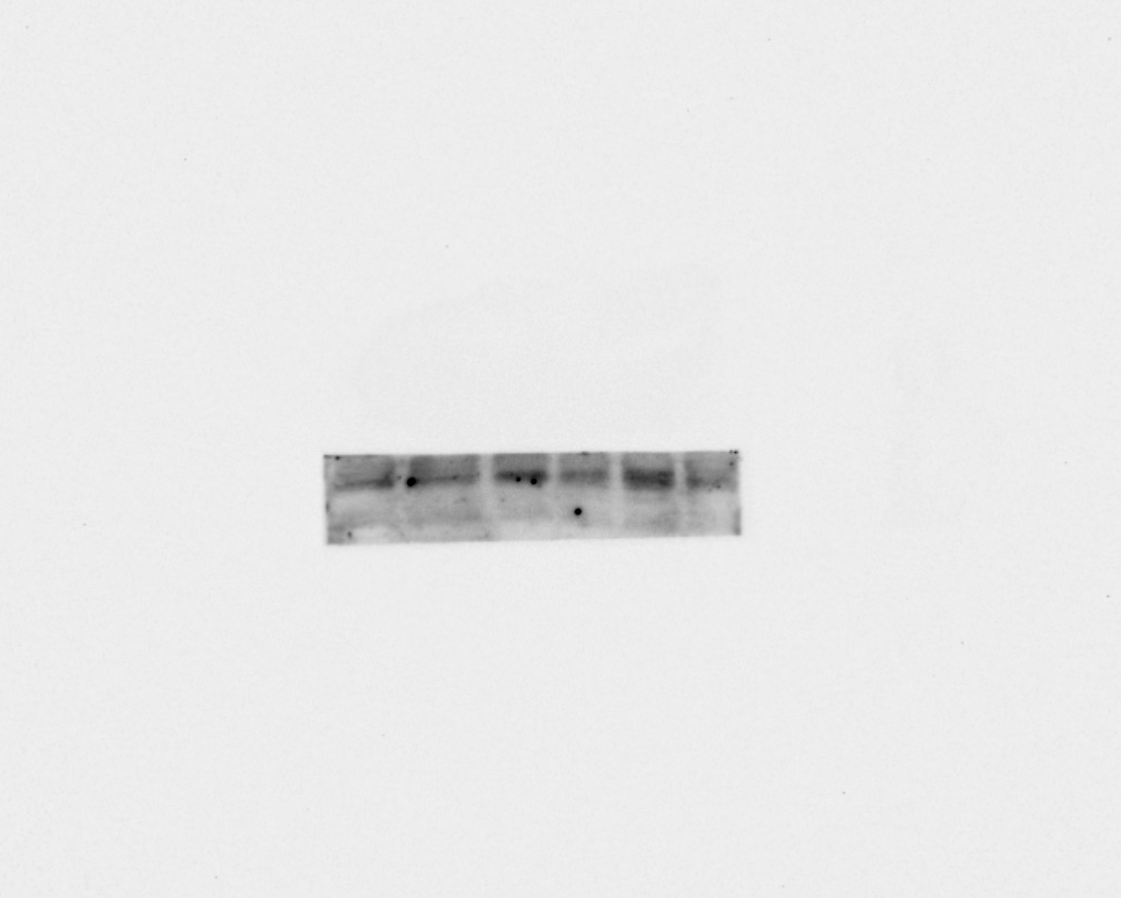

Supplement: Supplementary file 3 [file DataSheet4.zip › Fibroblast_Slug/Fibro Slug.tif]

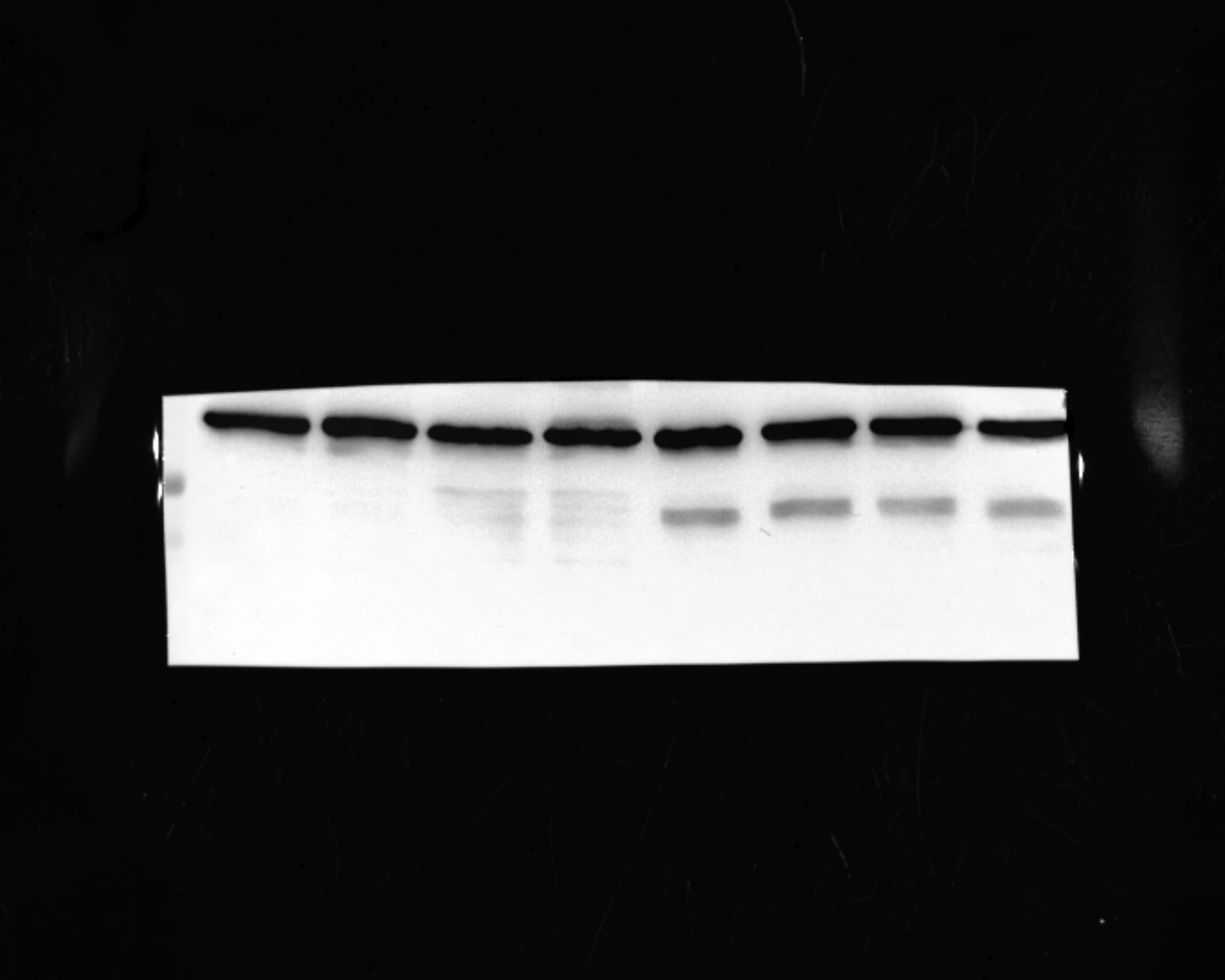

Supplement: Supplementary file 4 [file DataSheet1.zip › Capan2_Ecad/TIF/E cadherin V Aktin.tif]

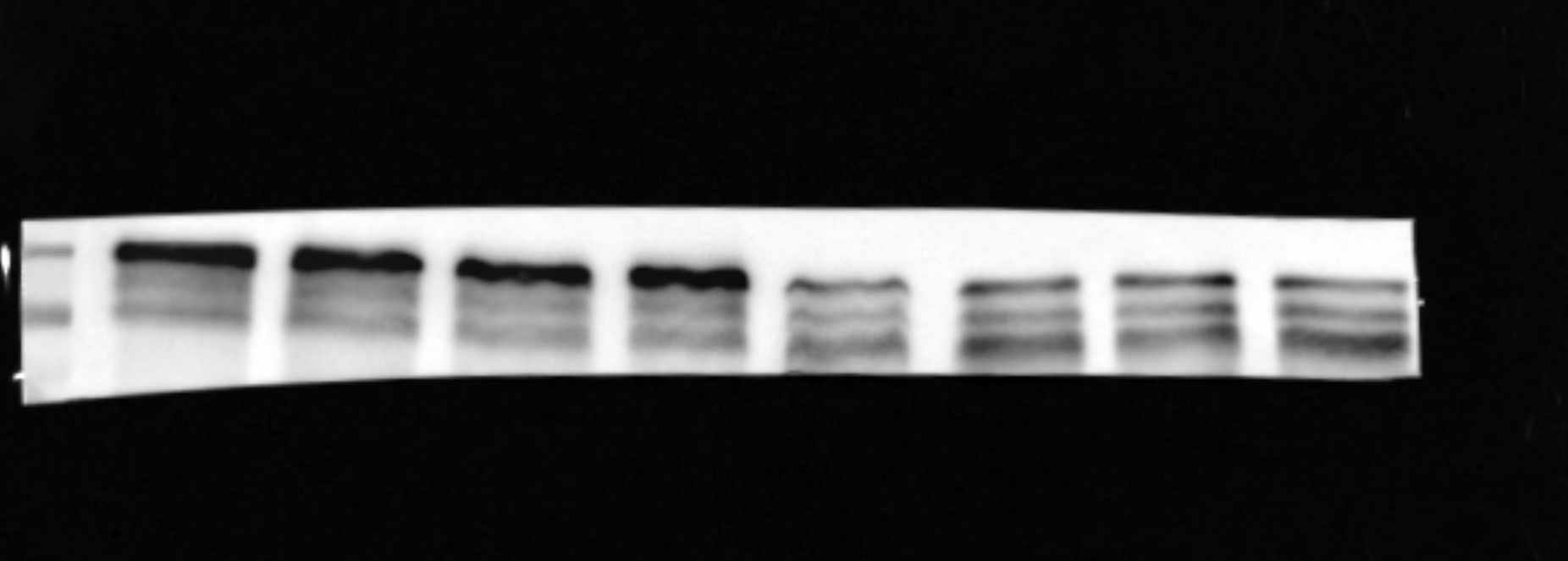

Supplement: Supplementary file 4 [file DataSheet1.zip › Capan2_Ecad/TIF/E cadherin V.tif]

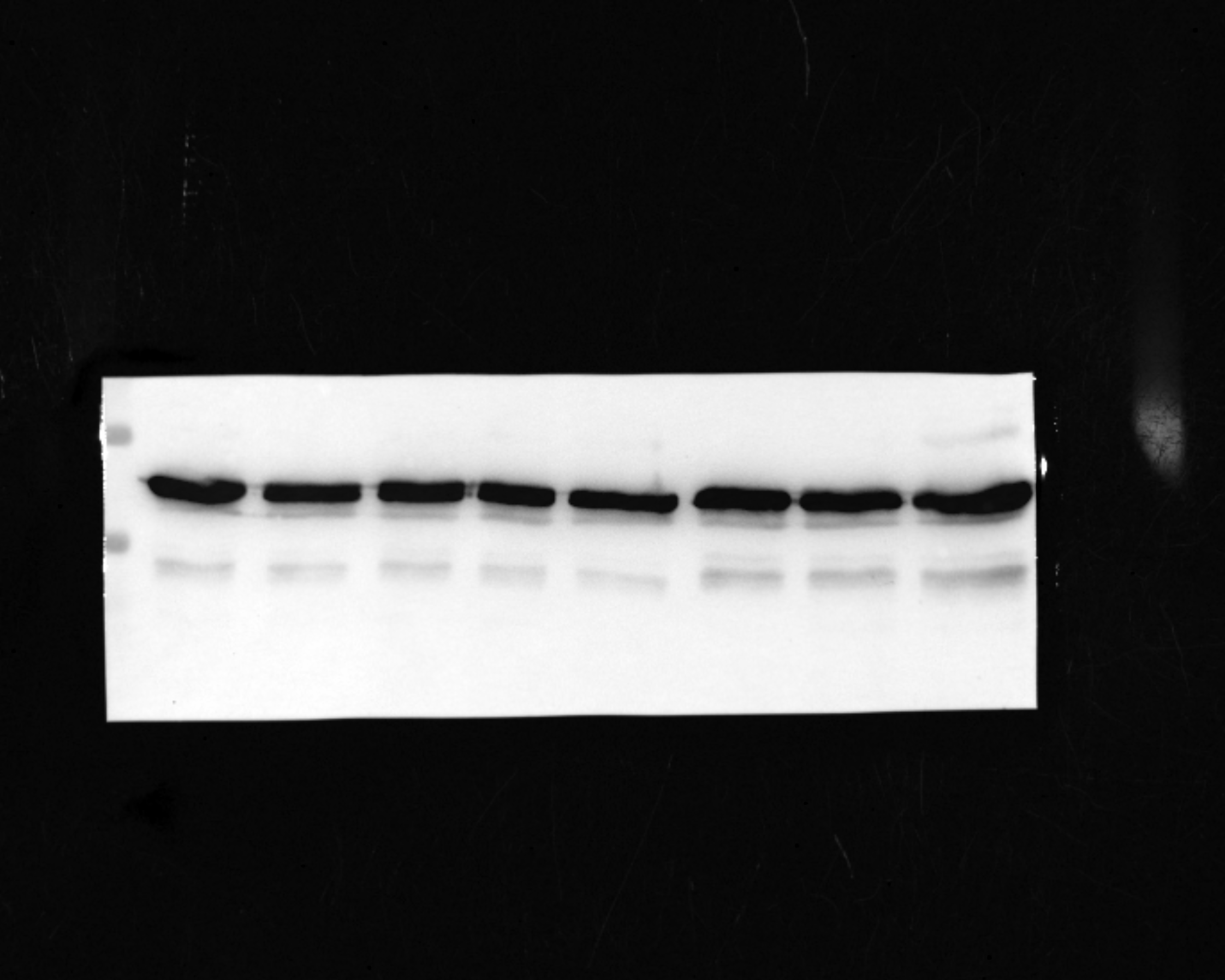

Supplement: Supplementary file 4 [file DataSheet1.zip › Capan2_Ecad/TIF/E-cadherin I-IV Aktin.tif]

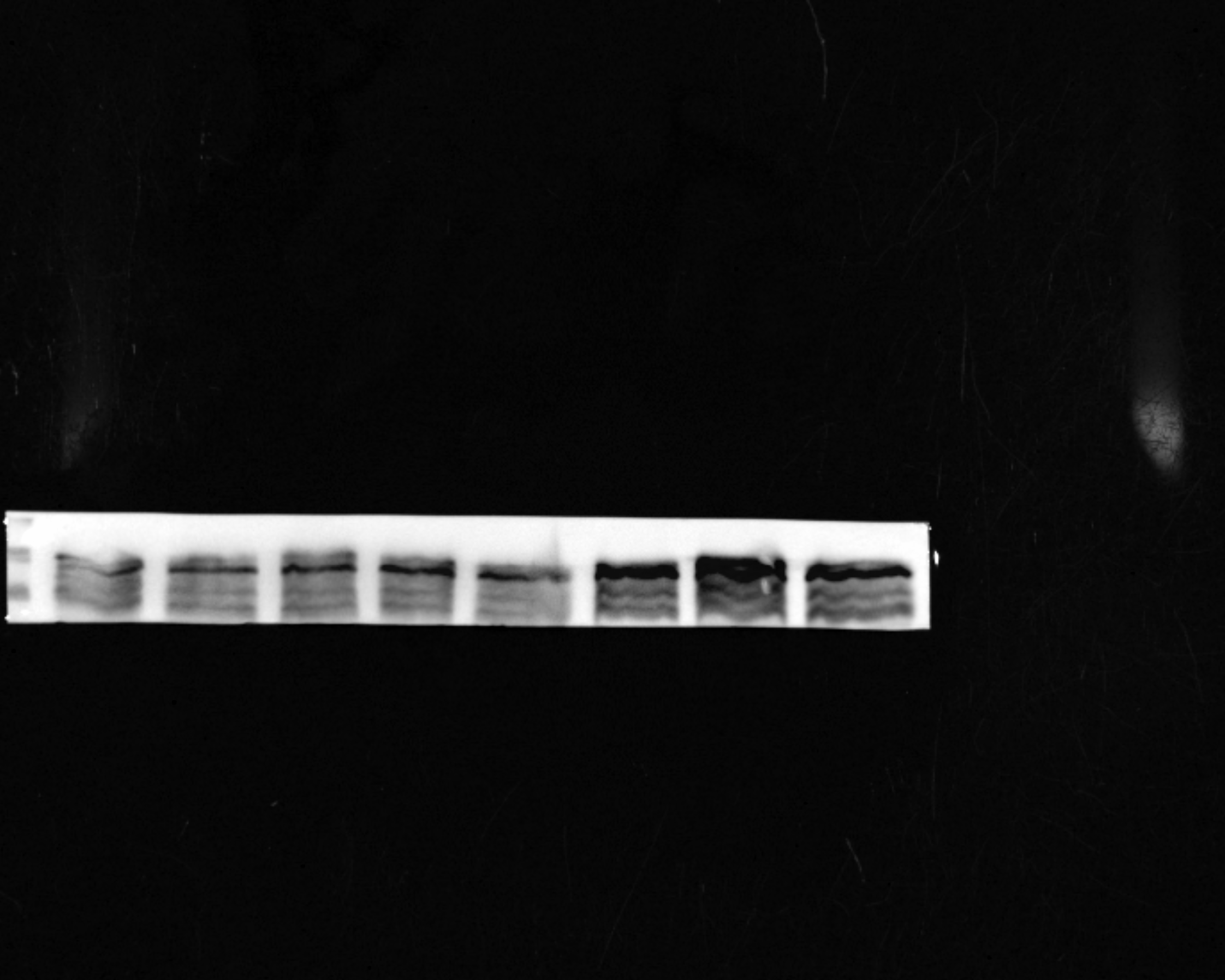

Supplement: Supplementary file 4 [file DataSheet1.zip › Capan2_Ecad/TIF/E-cadherin I-IV.tif]

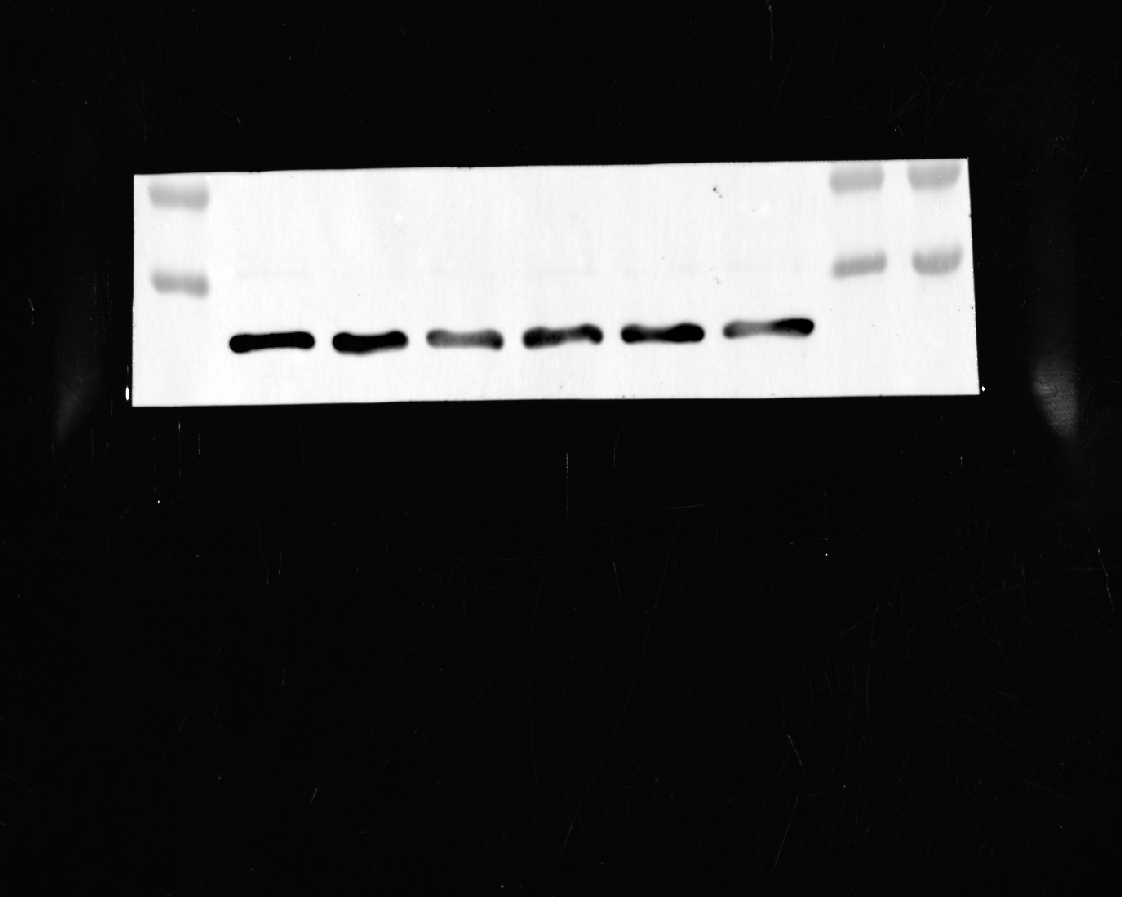

Supplement: Supplementary file 5 [file DataSheet6.zip › Capan2_ALDH1/TIF/ALDH 2. Actin (#23.19 II. Aktin_3).tif]

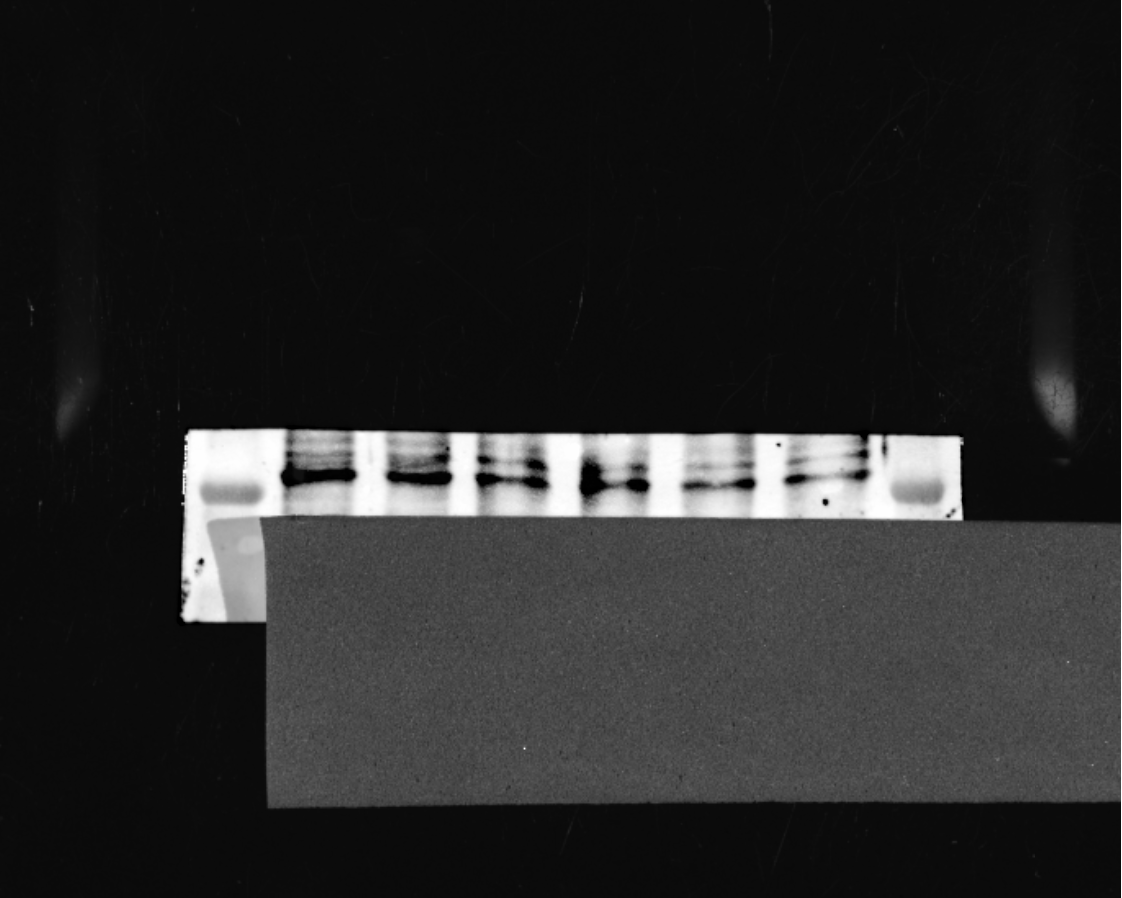

Supplement: Supplementary file 5 [file DataSheet6.zip › Capan2_ALDH1/TIF/ALDH1 1. (2022. 11. 21. Capan2 I. ALDH1_4).tif]

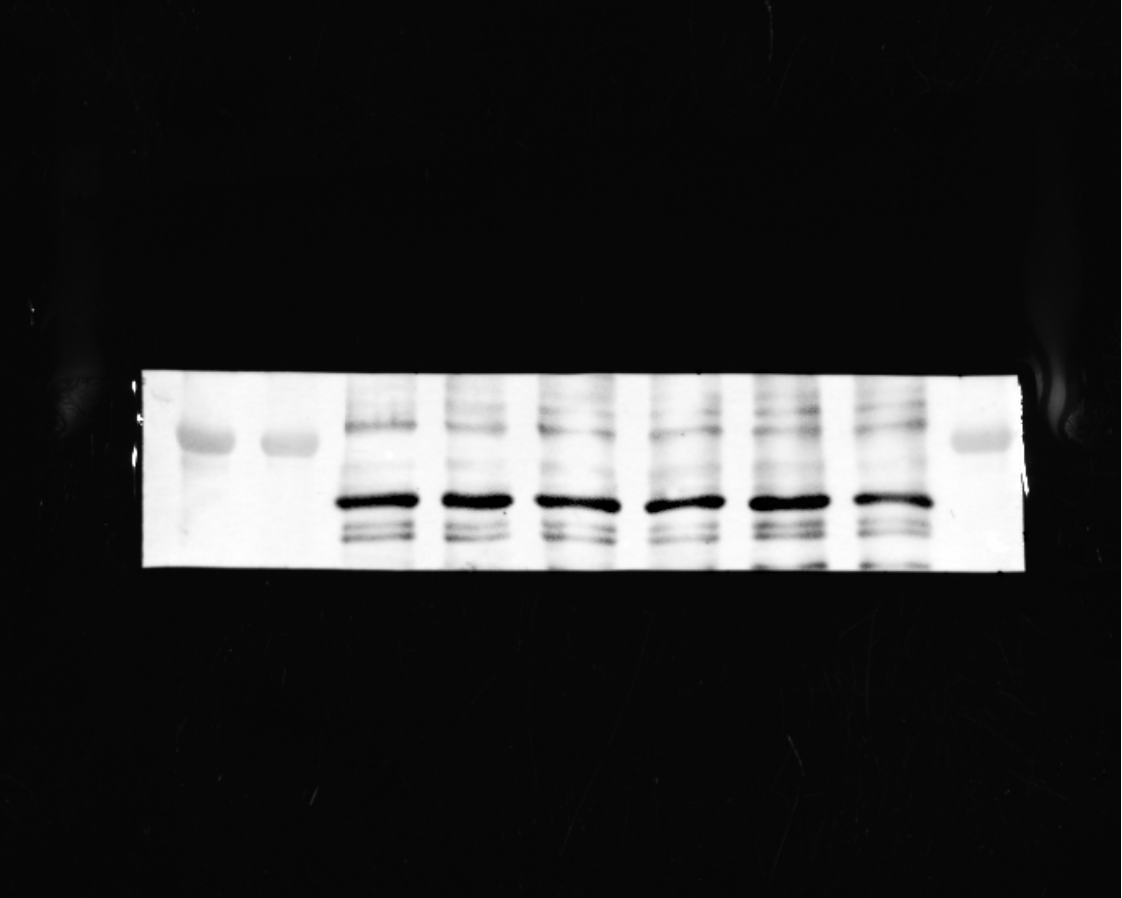

Supplement: Supplementary file 5 [file DataSheet6.zip › Capan2_ALDH1/TIF/ALDH1 1. Actin (2022. 11. 21. Capan2 II. Aktin_3).tif]

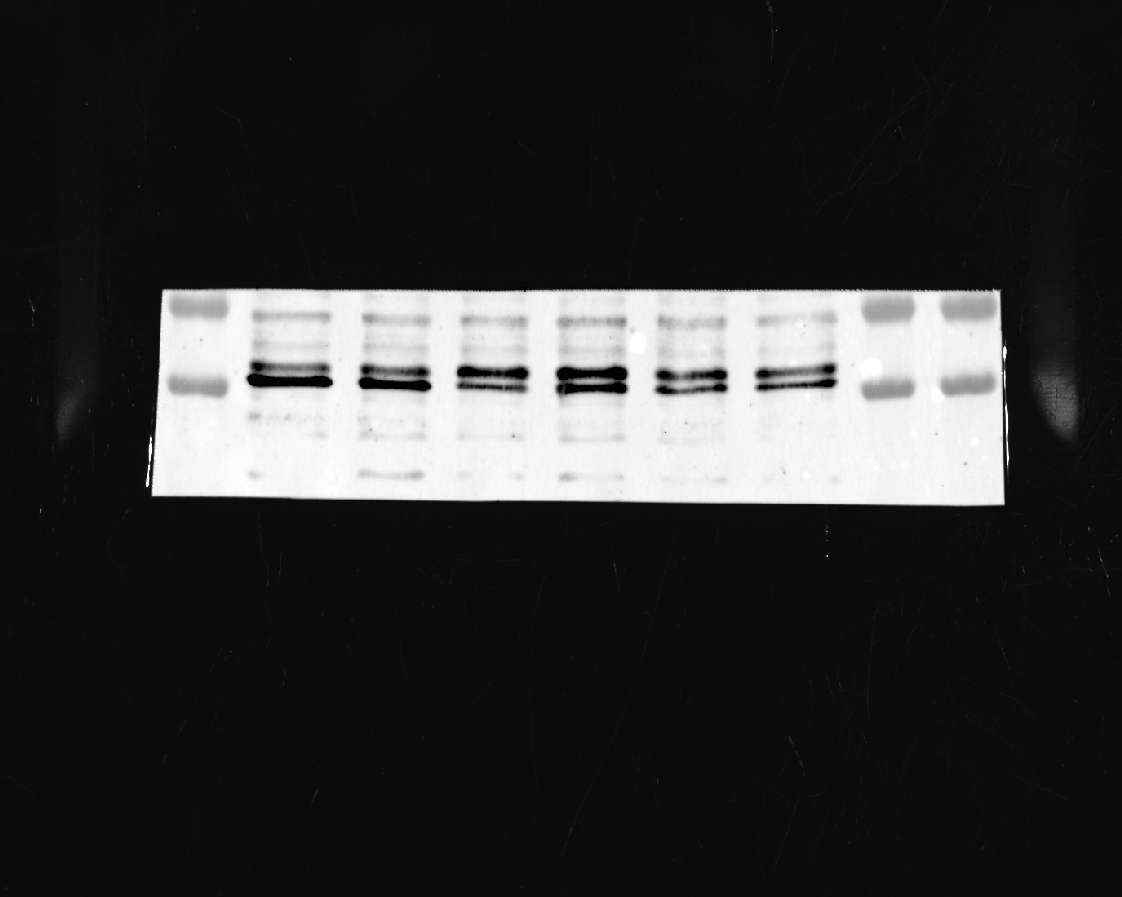

Supplement: Supplementary file 5 [file DataSheet6.zip › Capan2_ALDH1/TIF/ALDH1 2. (#23.19 I. ALDH1_3).tif]

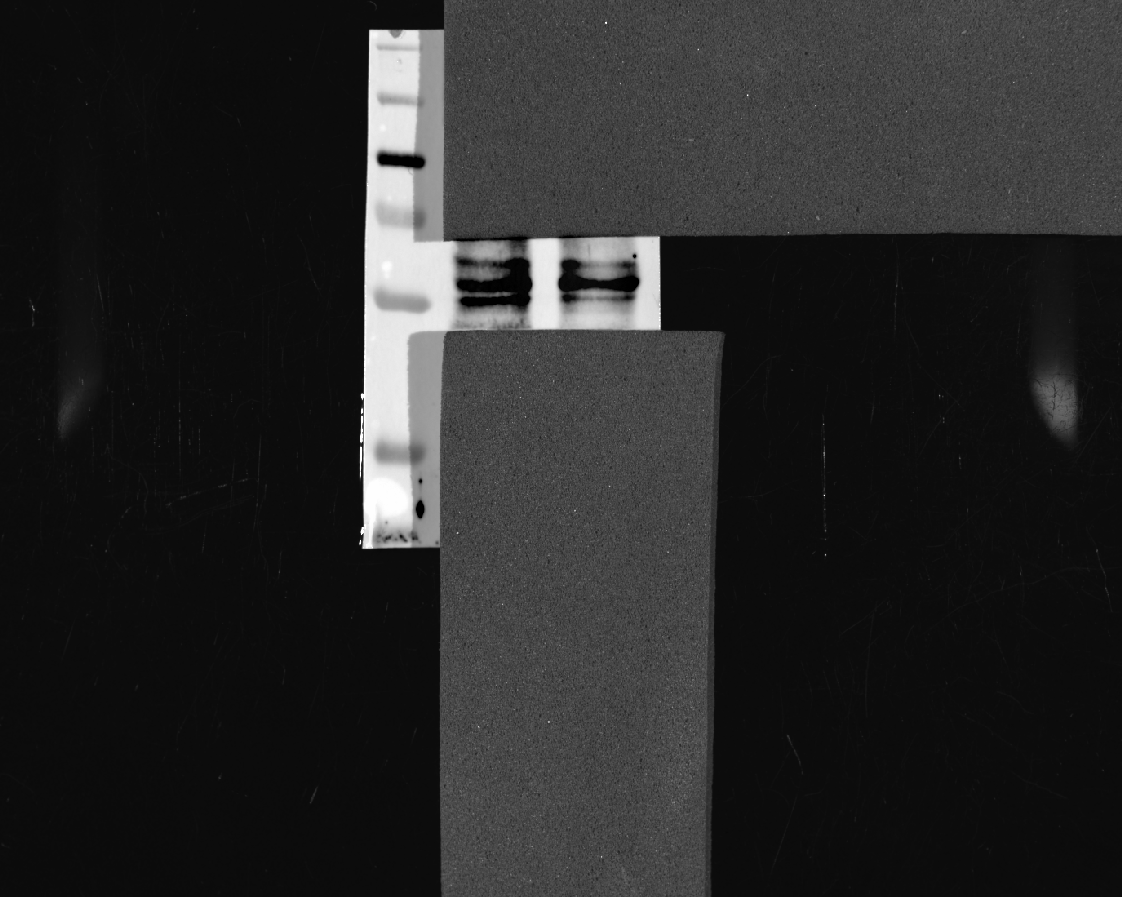

Supplement: Supplementary file 5 [file DataSheet6.zip › Capan2_ALDH1/TIF/ALDH1 3. (#23.02 Capan2 ALDH1 #22.27_4).tif]

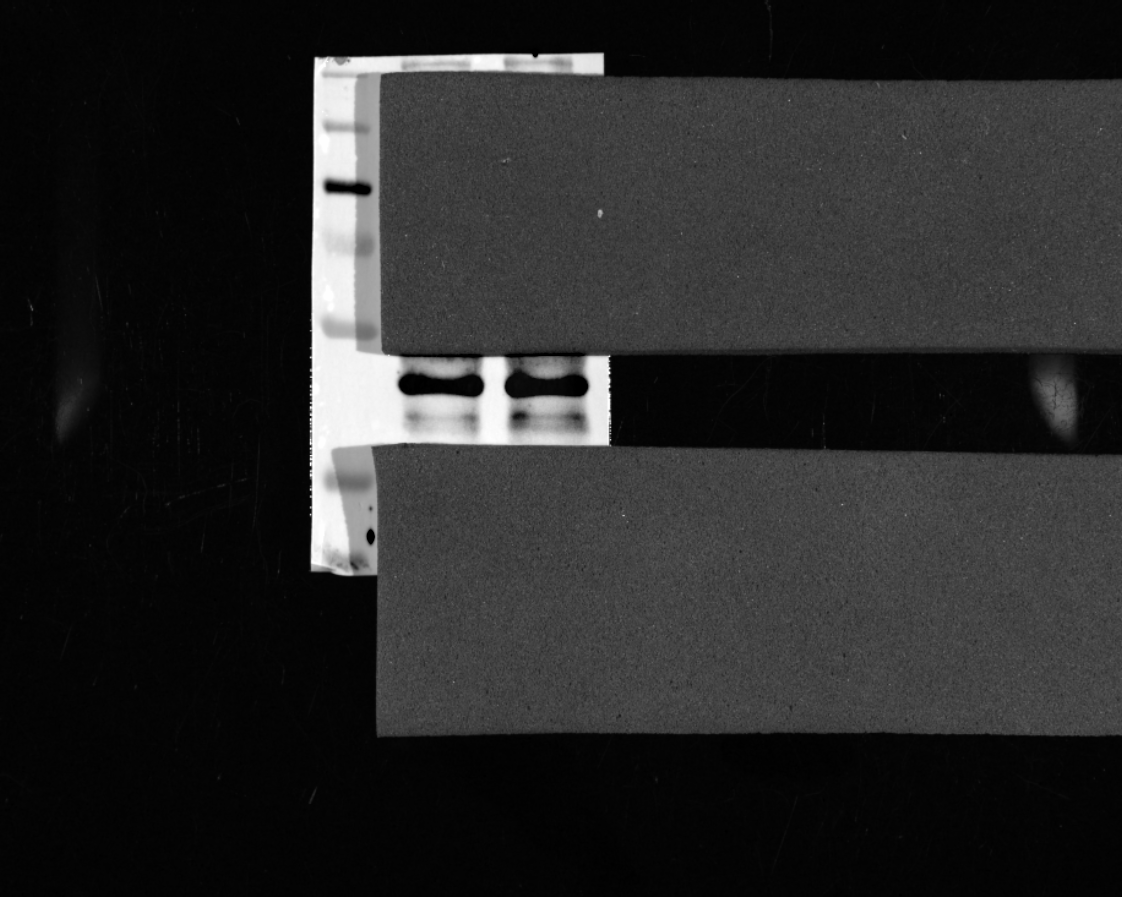

Supplement: Supplementary file 5 [file DataSheet6.zip › Capan2_ALDH1/TIF/ALDH1 3. Actin (#22.31 Capan2 ALDH1 Aktin #22.27_3).tif]

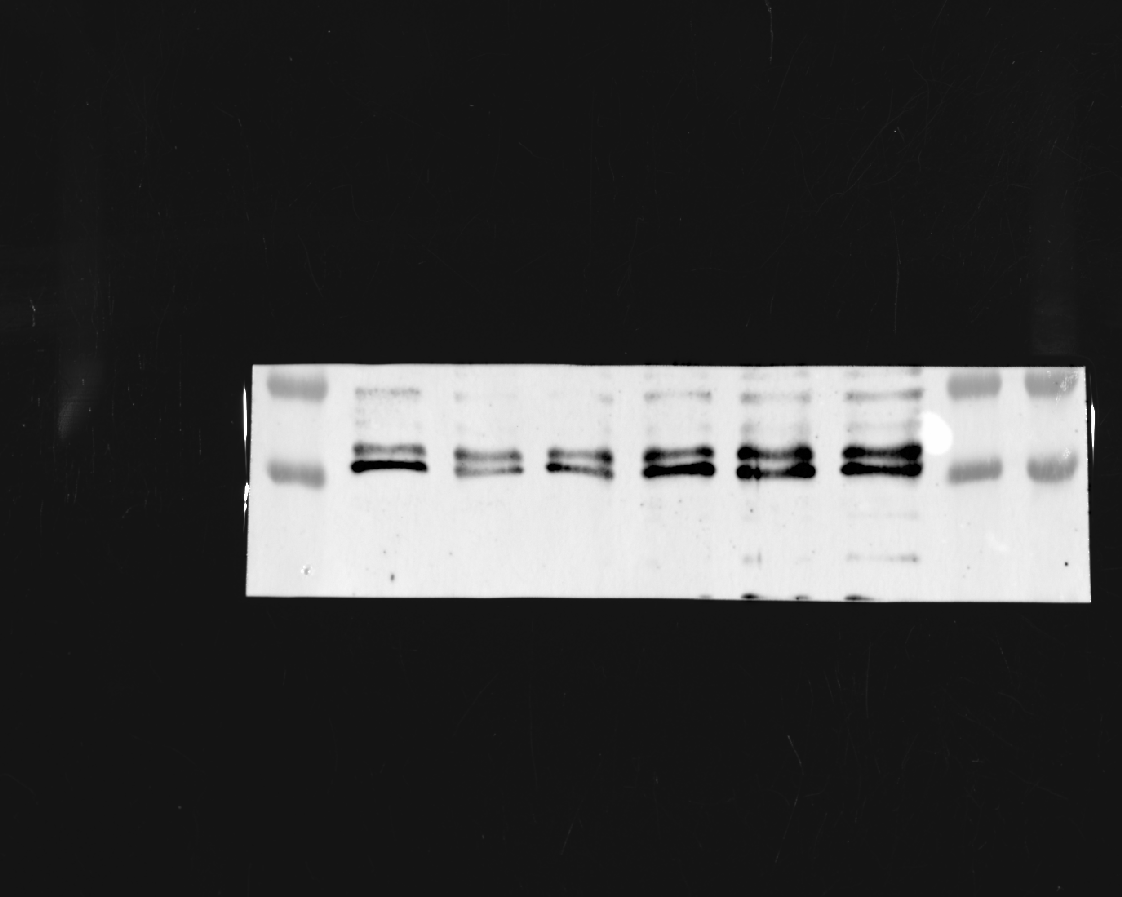

Supplement: Supplementary file 5 [file DataSheet6.zip › Capan2_ALDH1_antioxidants/TIF/ALDH1 antioxidants Patrik 1. (#23.19 II. ALDH1_1+#23.19 II. ALDH1_2).tif]

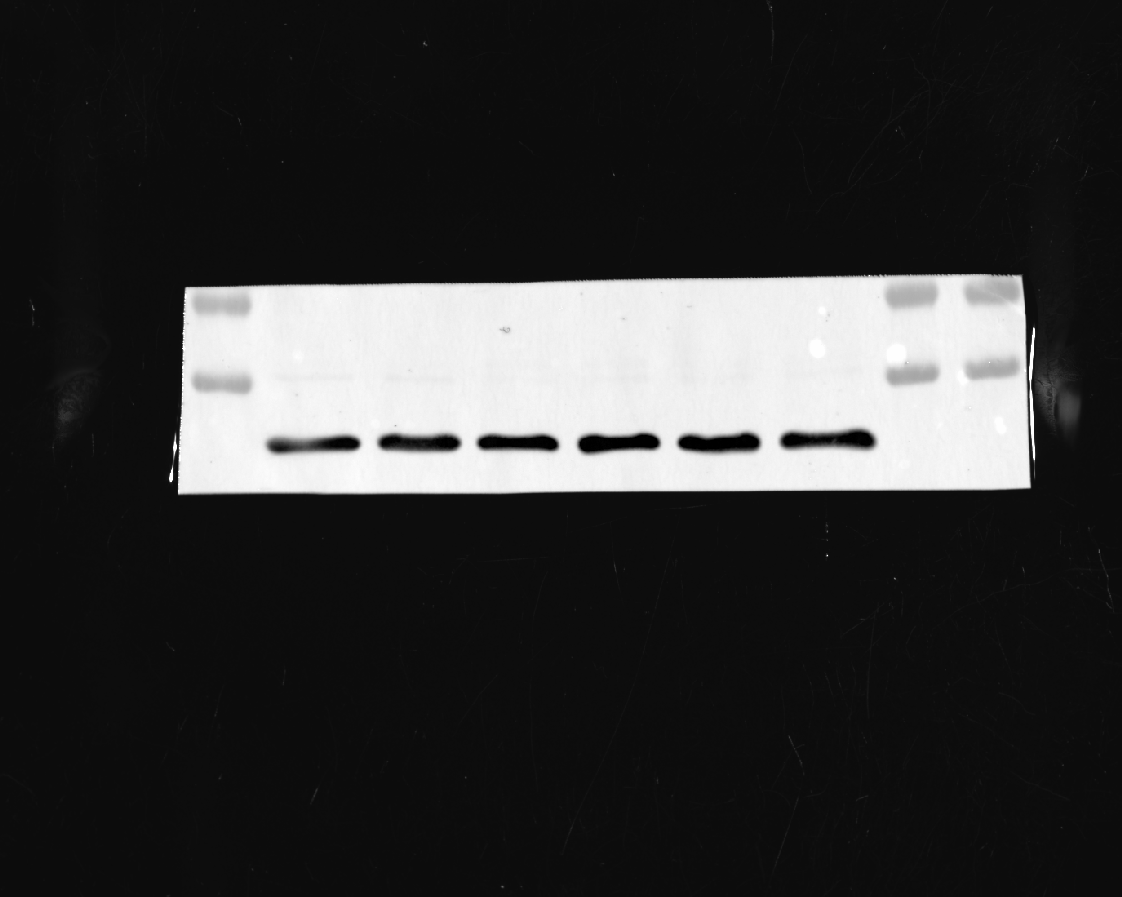

Supplement: Supplementary file 5 [file DataSheet6.zip › Capan2_ALDH1_antioxidants/TIF/ALDH1 antioxidants Patrik 1. Actin (#23.19 I. Aktin_3).tif]

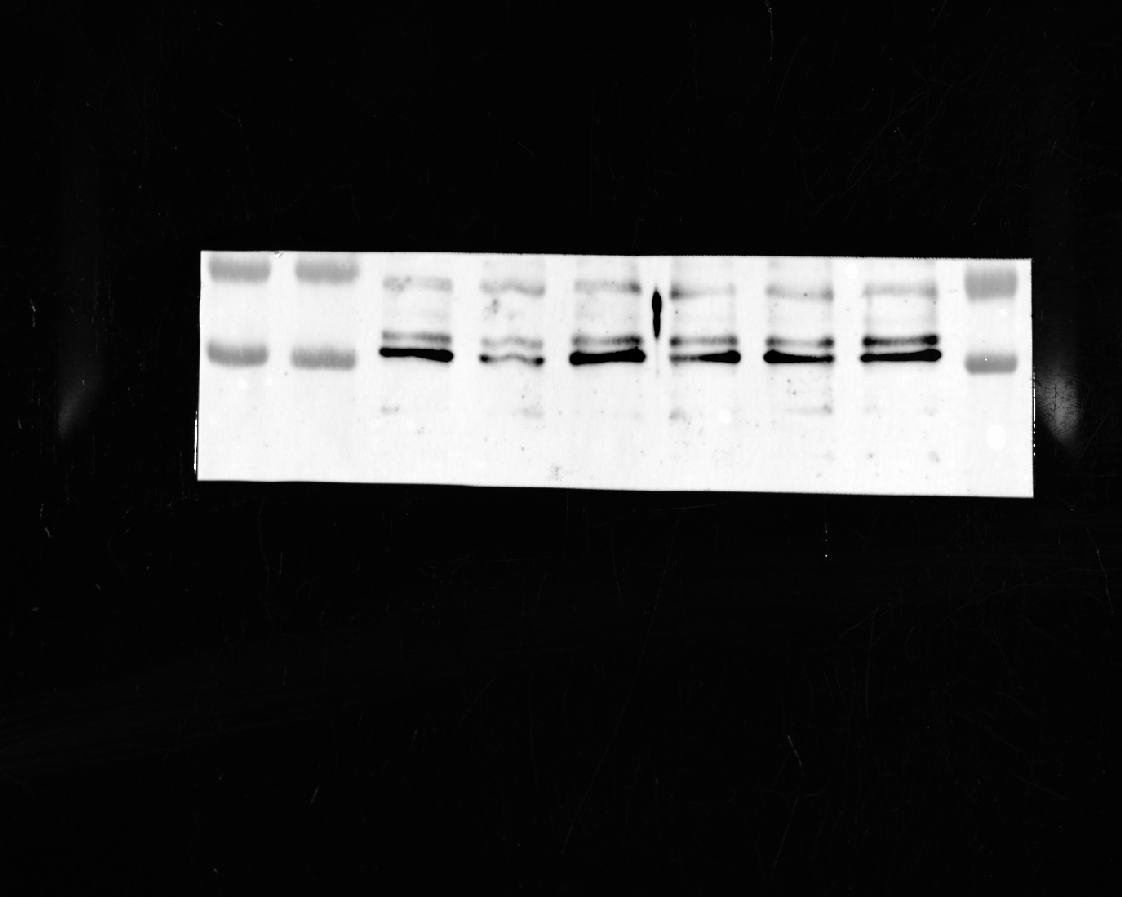

Supplement: Supplementary file 5 [file DataSheet6.zip › Capan2_ALDH1_antioxidants/TIF/ALDH1 antioxidants Patrik 2. (#23.18 II. ALDH1_3).tif]

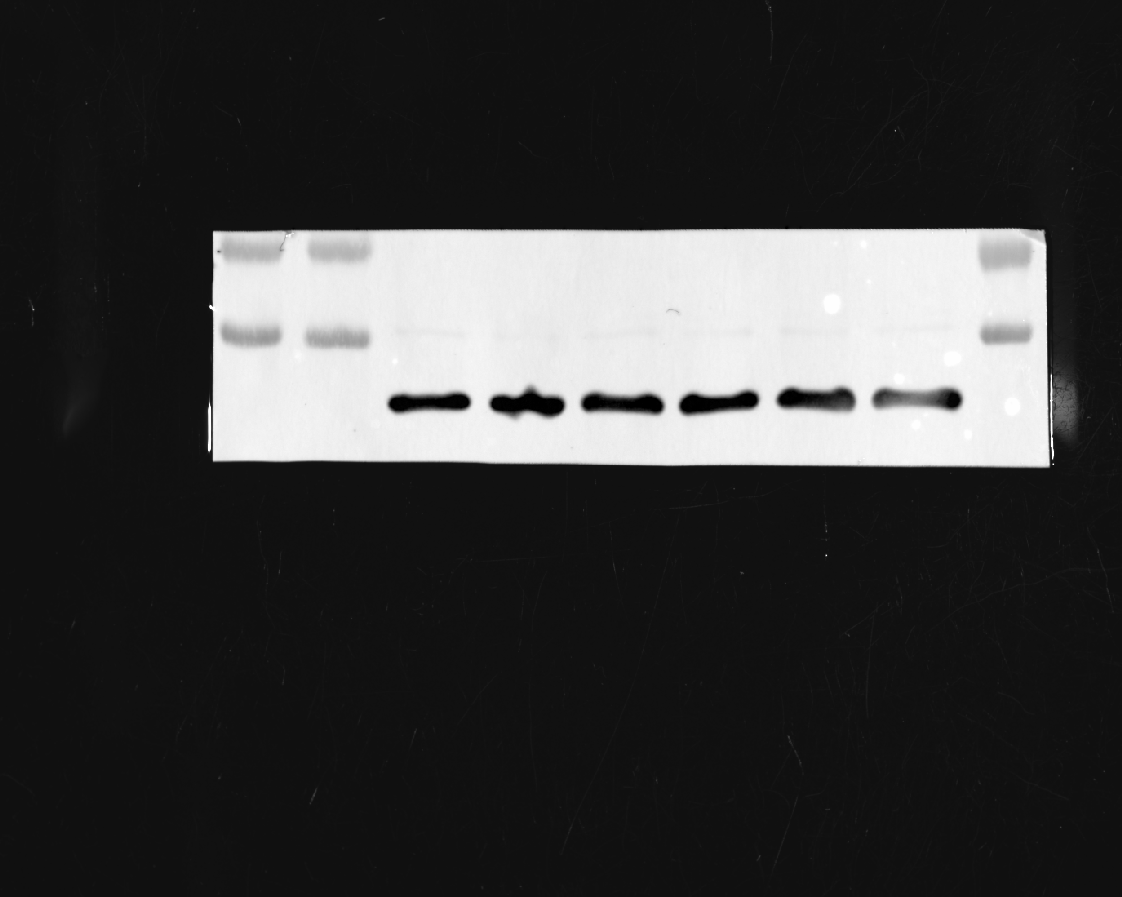

Supplement: Supplementary file 5 [file DataSheet6.zip › Capan2_ALDH1_antioxidants/TIF/ALDH1 antioxidants Patrik 2. Actin ( #23.18 II. Aktin_3).tif]

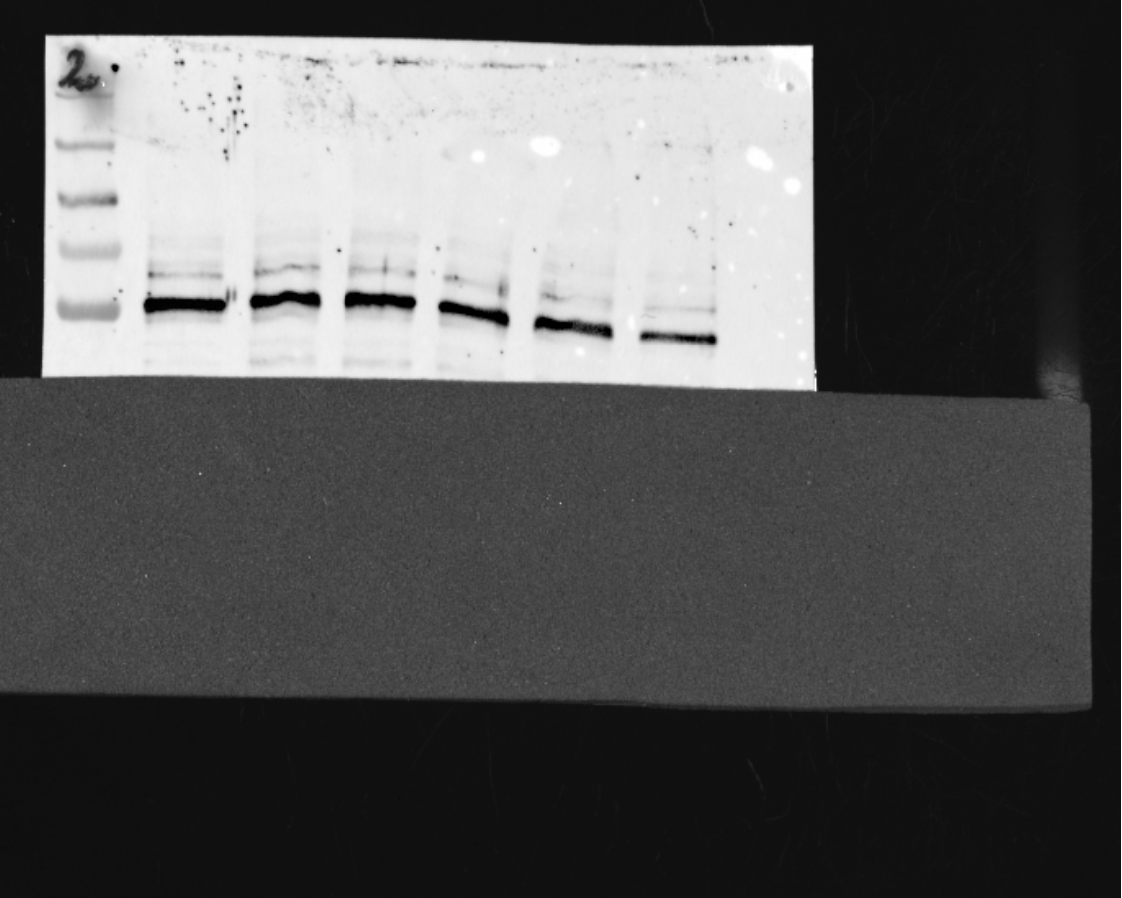

Supplement: Supplementary file 5 [file DataSheet6.zip › Capan2_ALDH1_antioxidants/TIF/ALDH1 antioxidants Szandra 1. (Capan2.2. sorozat.Mito.Pegc.ALDH1.2024.04.25_6).tif]

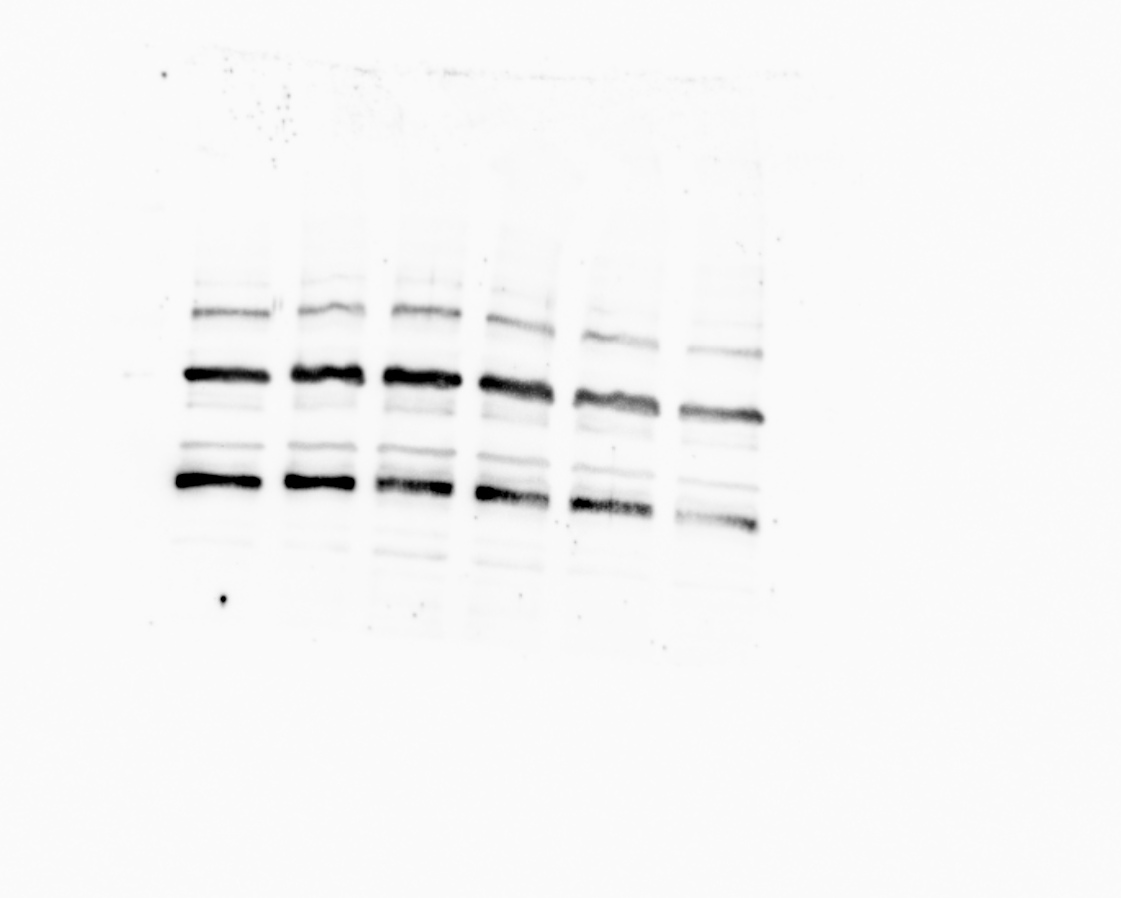

Supplement: Supplementary file 5 [file DataSheet6.zip › Capan2_ALDH1_antioxidants/TIF/ALDH1 antioxidants Szandra 1. Actin (Capan2.2. sorozat.Mito.Pegc.ALDH1.2024.04.25.AKTIN_1).tif]

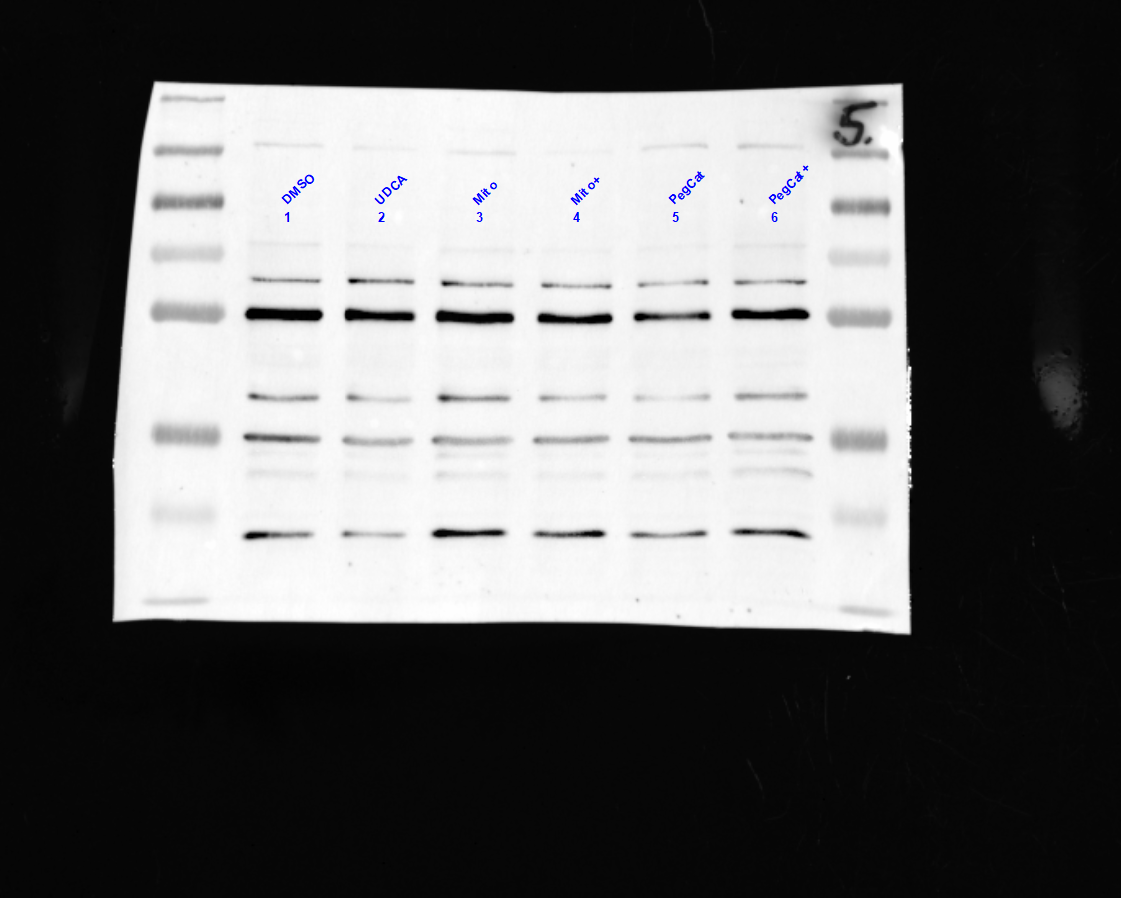

Supplement: Supplementary file 5 [file DataSheet6.zip › Capan2_ALDH1_antioxidants/TIF/ALDH1 antioxidants Szandra 2. (Capan2.5. sorozat.Mito.Pegc.ALDH1.2024.04.25_3).tif]

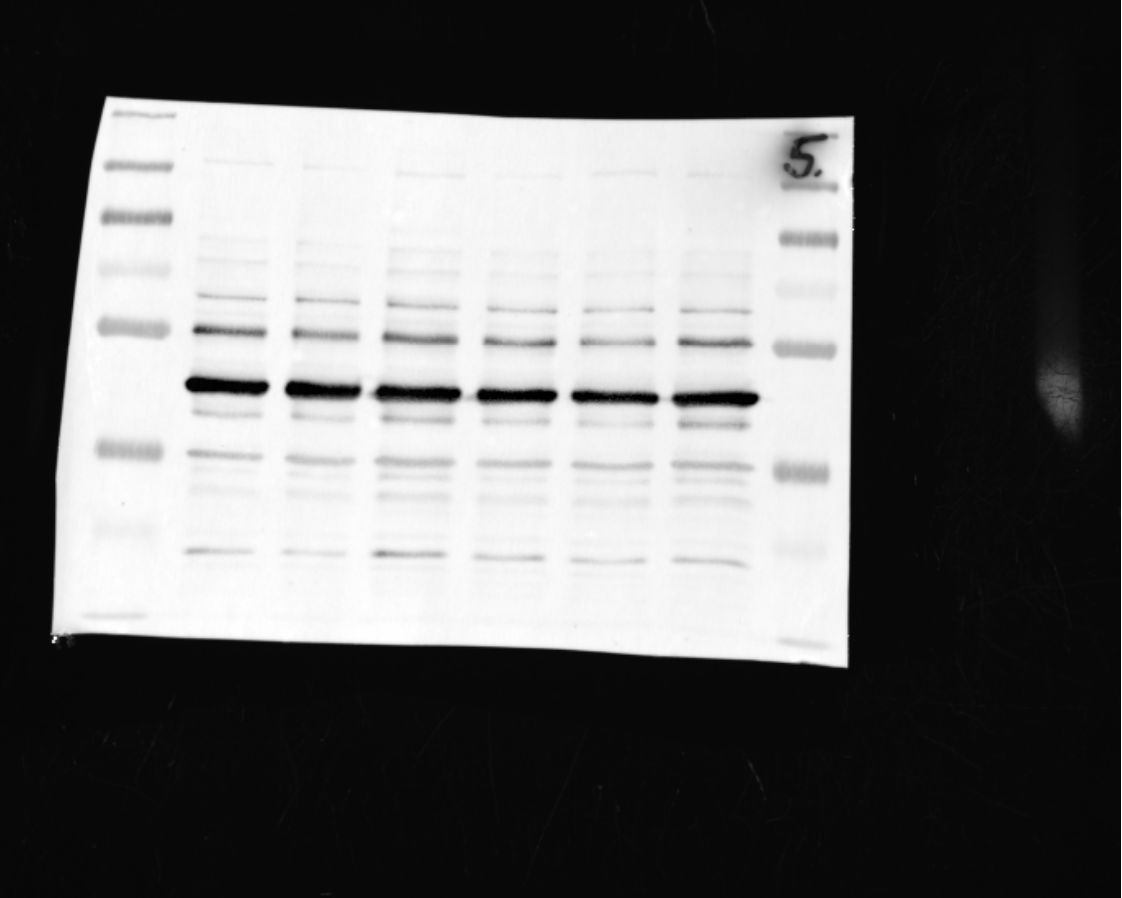

Supplement: Supplementary file 5 [file DataSheet6.zip › Capan2_ALDH1_antioxidants/TIF/ALDH1 antioxidants Szandra 2. Actin (Capan2.5. sorozat.Mito.Pegc.ALDH1.2024.04.25.AKTIN_3).tif]

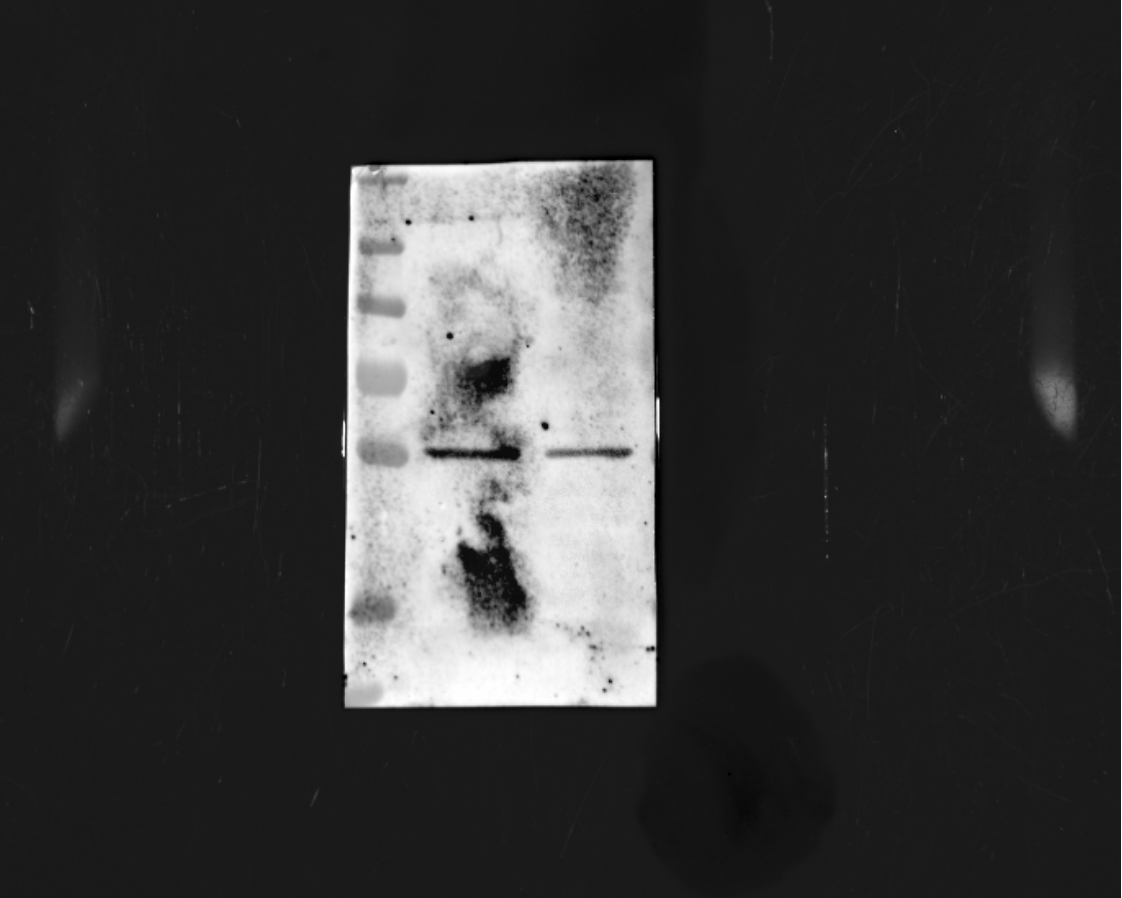

Supplement: Supplementary file 5 [file DataSheet6.zip › Capan2_CD24/TIF/CD24 1. (#22.27 CD24_3).tif]

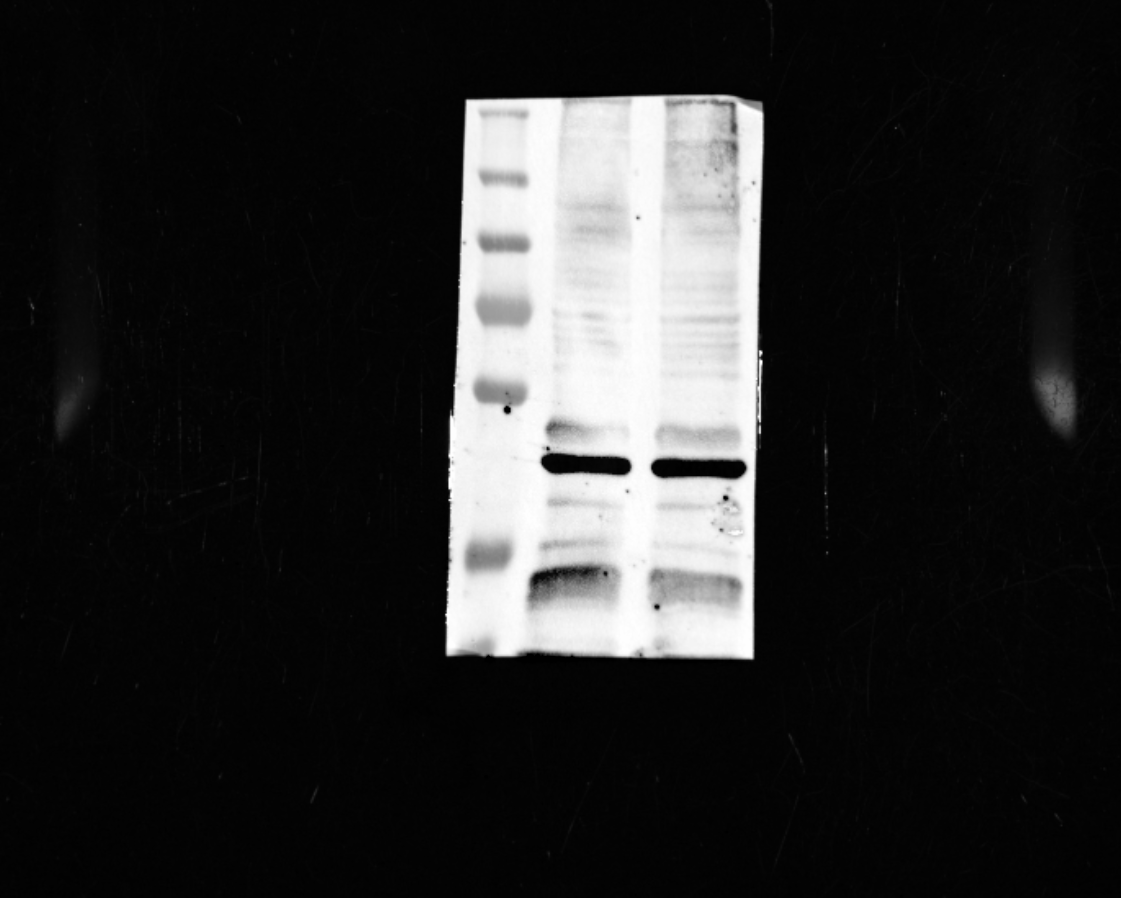

Supplement: Supplementary file 5 [file DataSheet6.zip › Capan2_CD24/TIF/CD24 1. Actin (03. 02. #22.27 CD24 Aktin_3).tif]

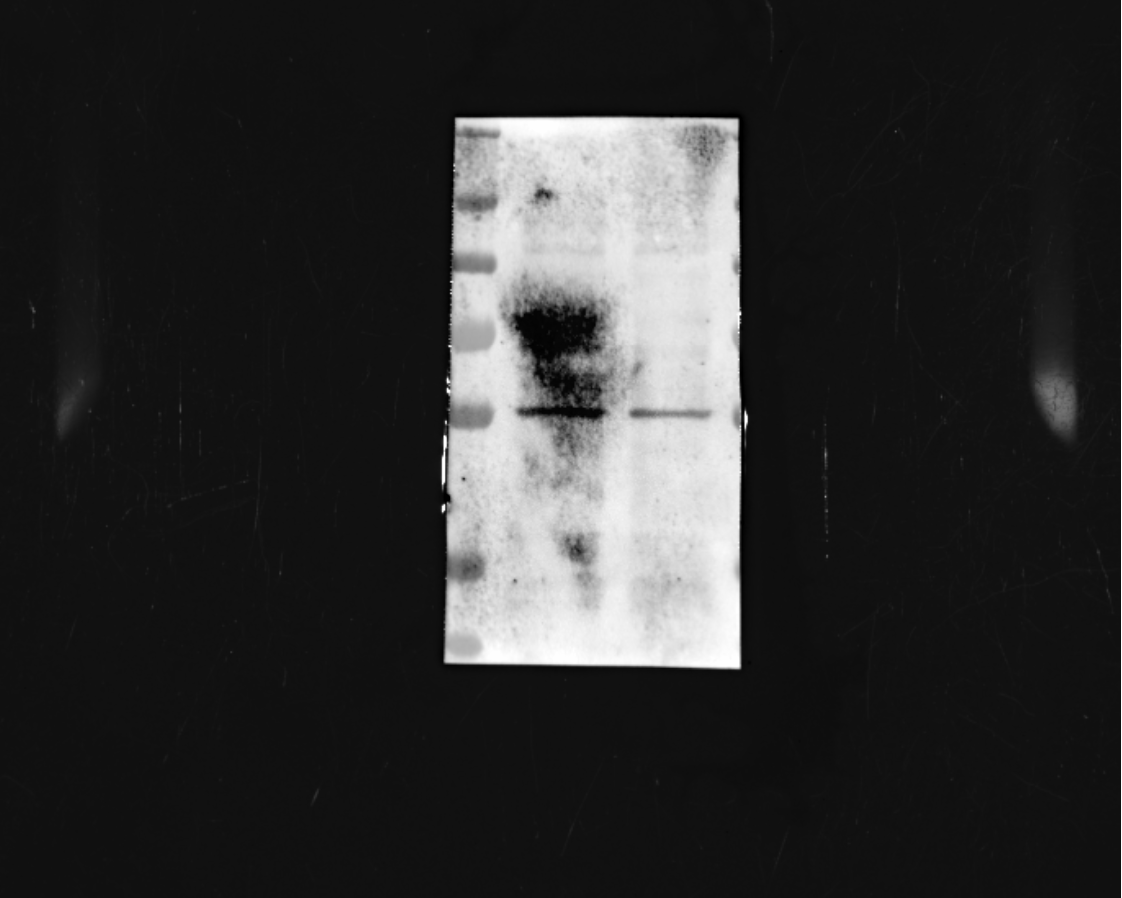

Supplement: Supplementary file 5 [file DataSheet6.zip › Capan2_CD24/TIF/CD24 2. (#22.28 CD24_3).tif]

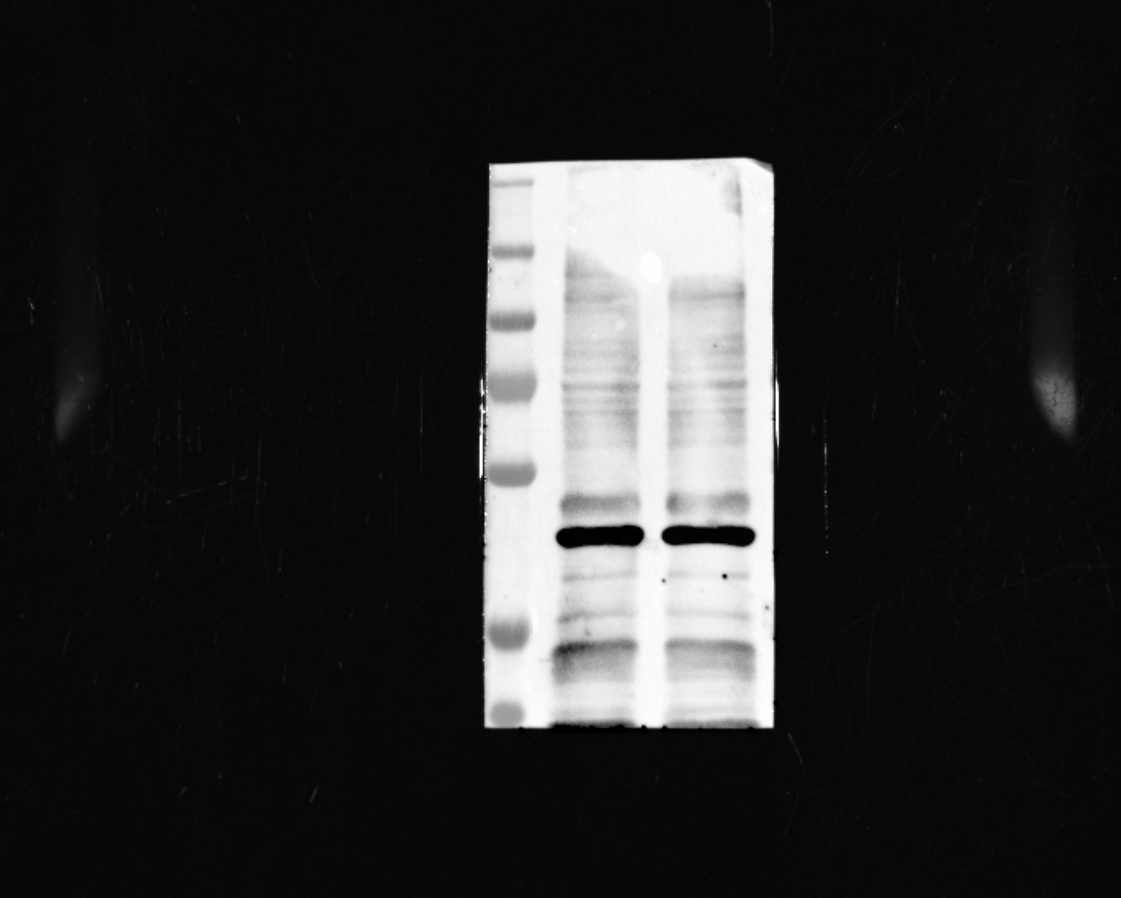

Supplement: Supplementary file 5 [file DataSheet6.zip › Capan2_CD24/TIF/CD24 2. Actin (03. 02. #22.28 CD24 Aktin_3).tif]

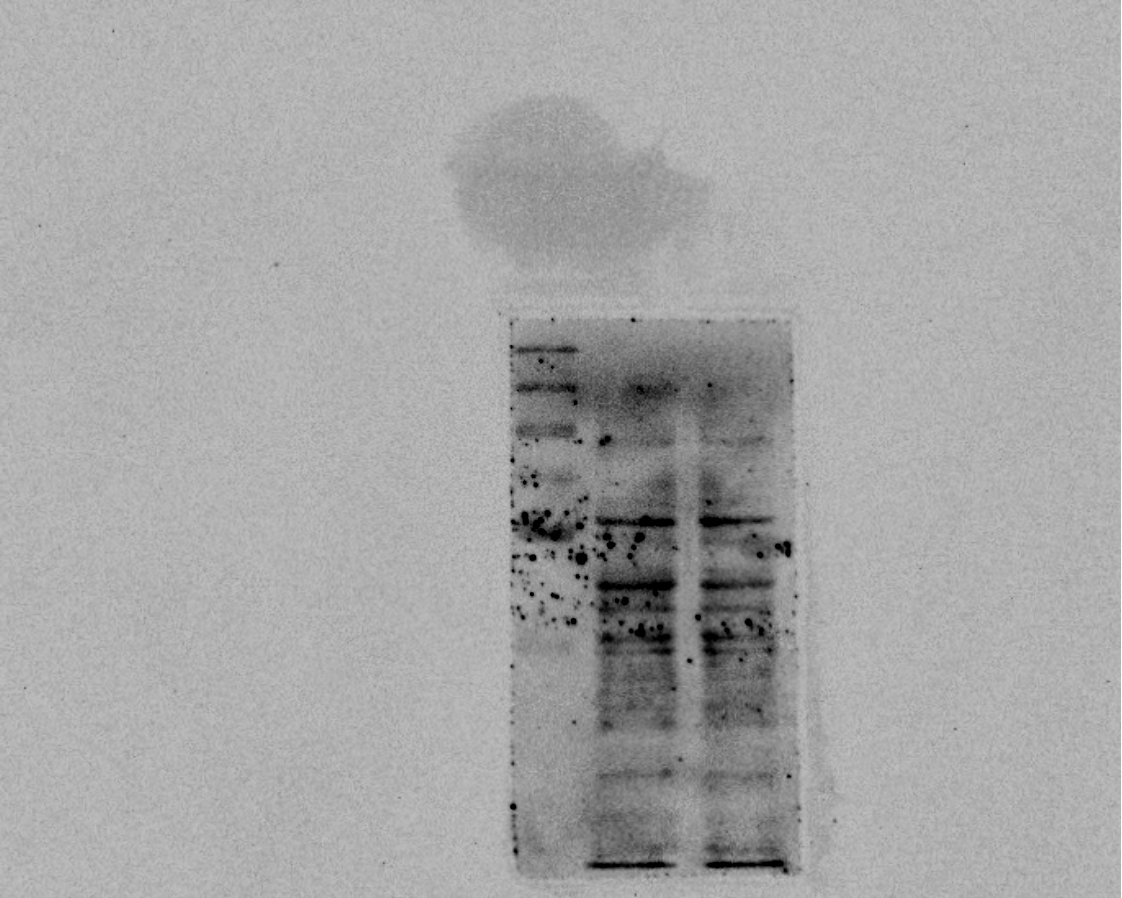

Supplement: Supplementary file 5 [file DataSheet6.zip › Capan2_CD24/TIF/CD24 3. (#23.51 CD24_06).tif]

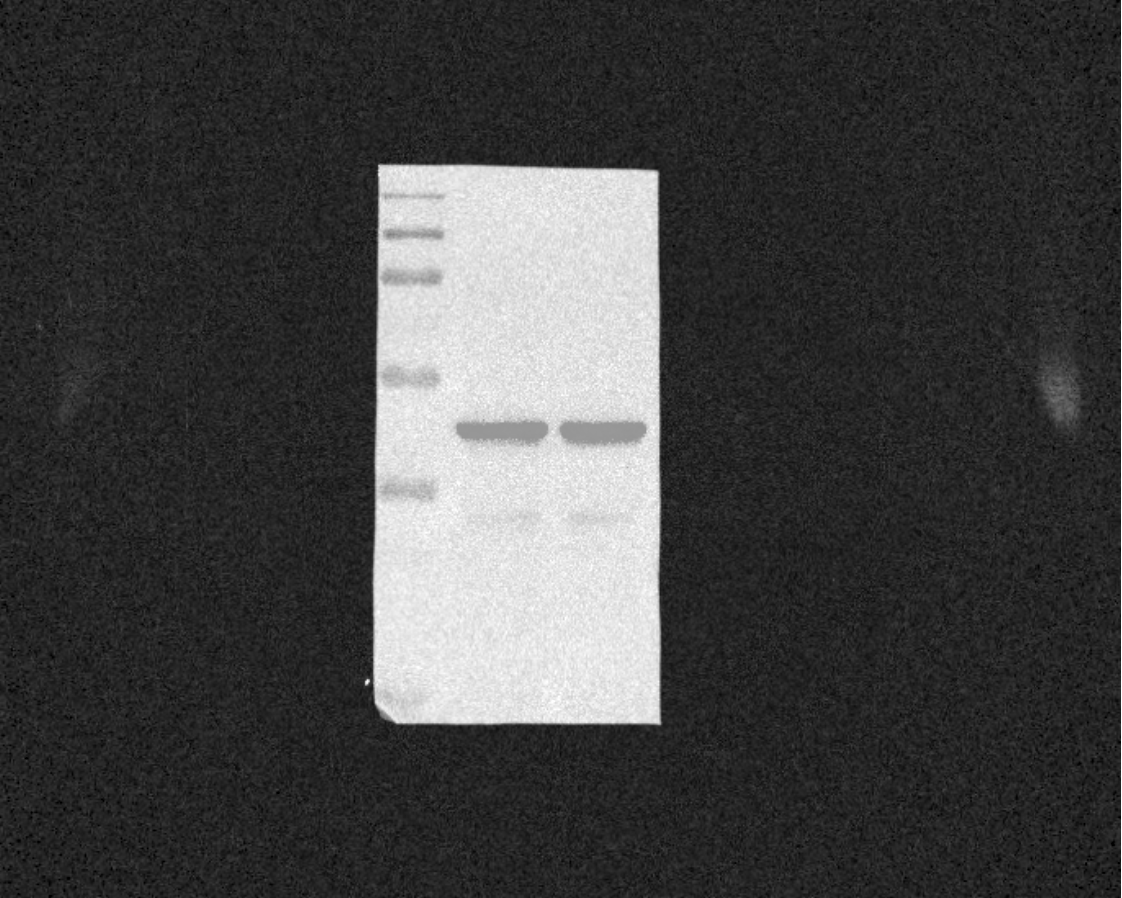

Supplement: Supplementary file 5 [file DataSheet6.zip › Capan2_CD24/TIF/CD24 3. Actin (#23.51 CD24 Aktin_9).tif]

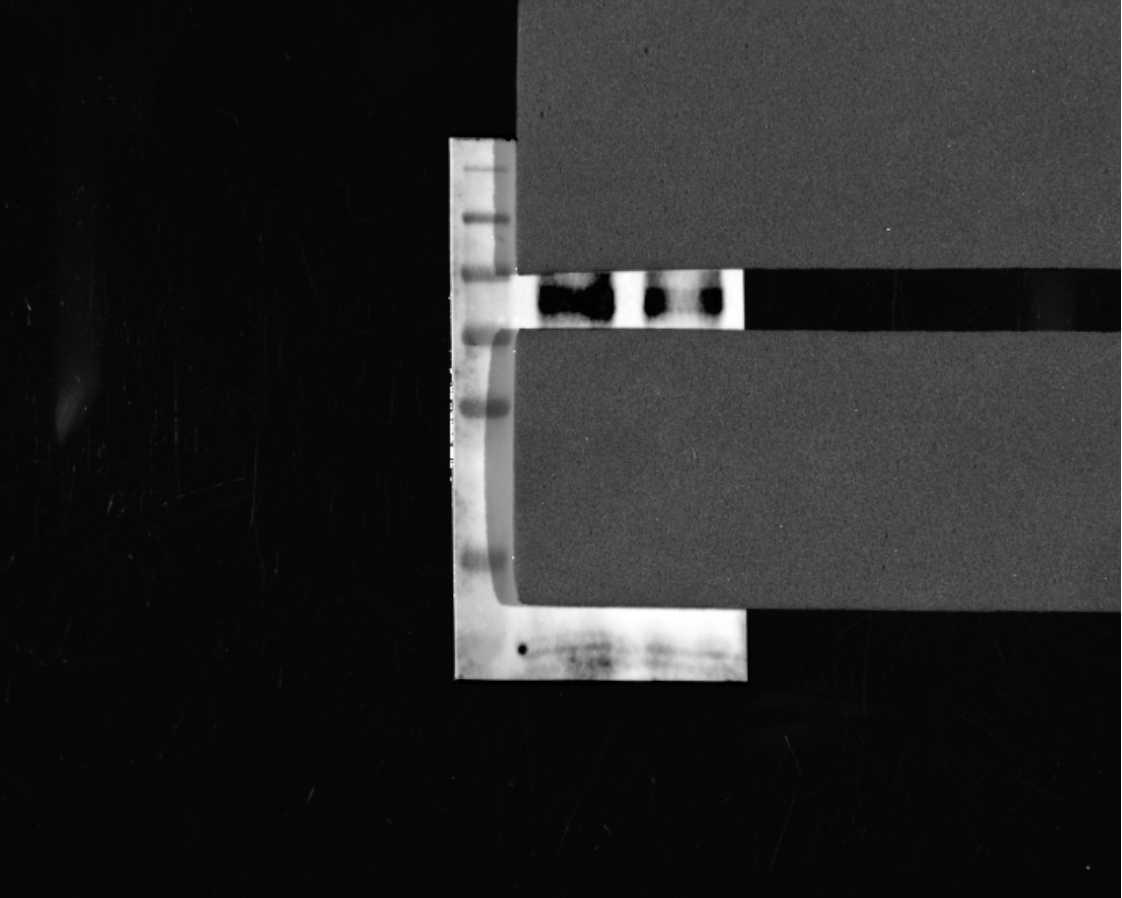

Supplement: Supplementary file 5 [file DataSheet6.zip › Capan2_CD44/TIF/CD44 1. (#23.05 Capan2 #22.29 CD44_3).tif]

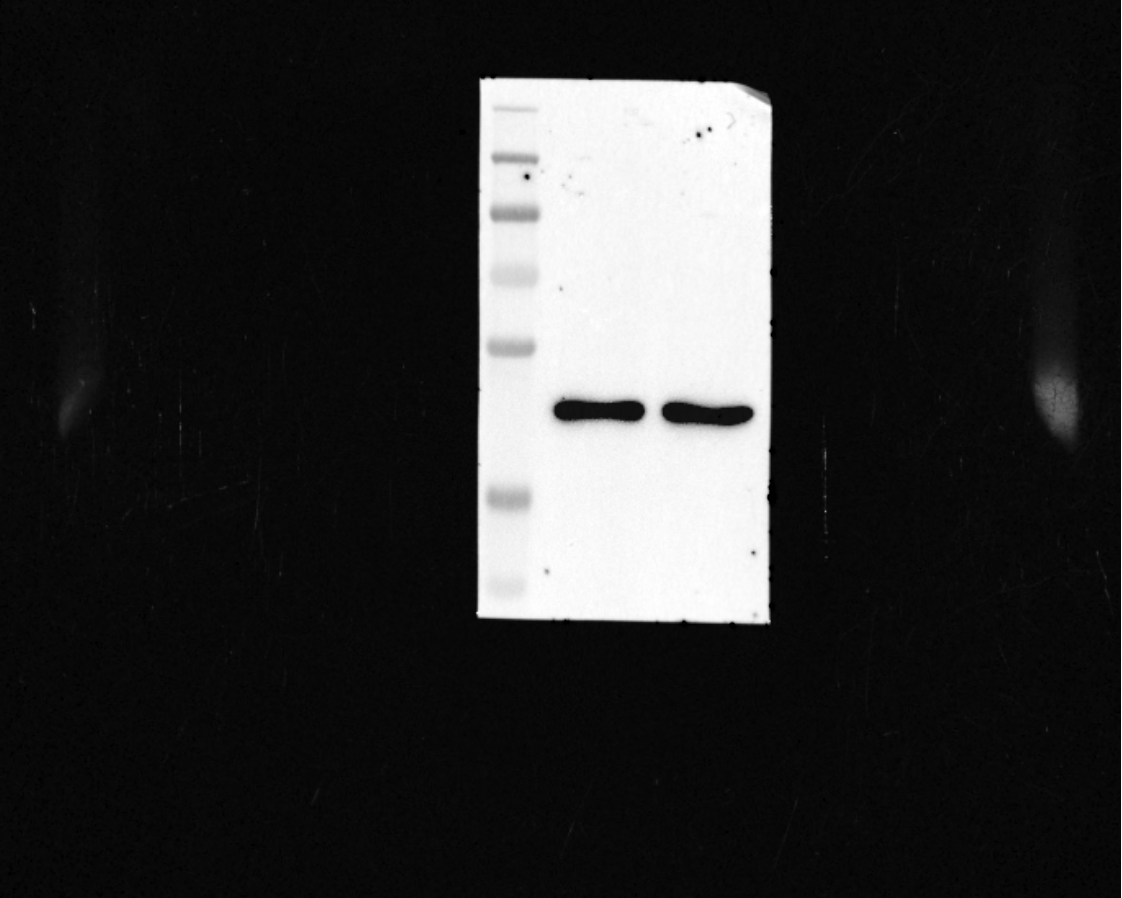

Supplement: Supplementary file 5 [file DataSheet6.zip › Capan2_CD44/TIF/CD44 1. Actin (#23.05 Capan2 #22.29 CD44 Aktin_3).tif]

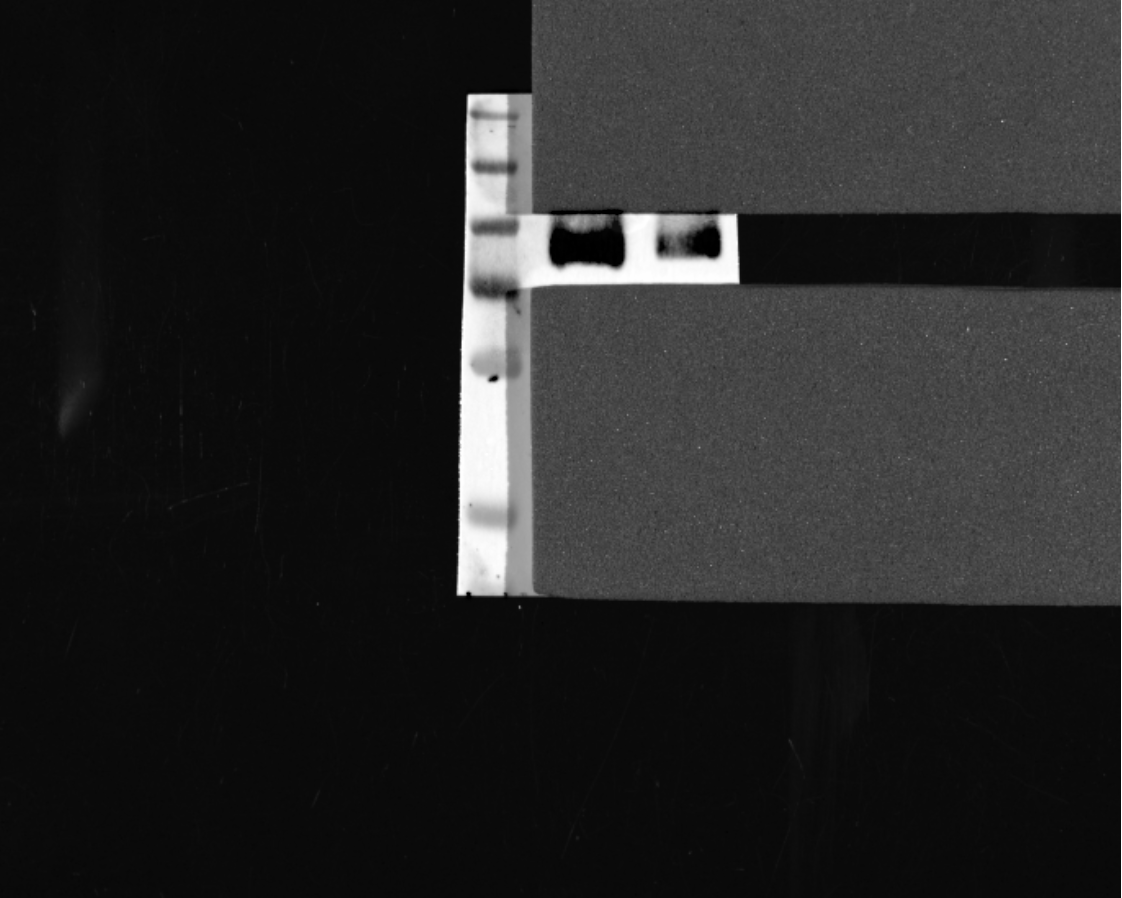

Supplement: Supplementary file 5 [file DataSheet6.zip › Capan2_CD44/TIF/CD44 2. (#23.12 CD44_3).tif]

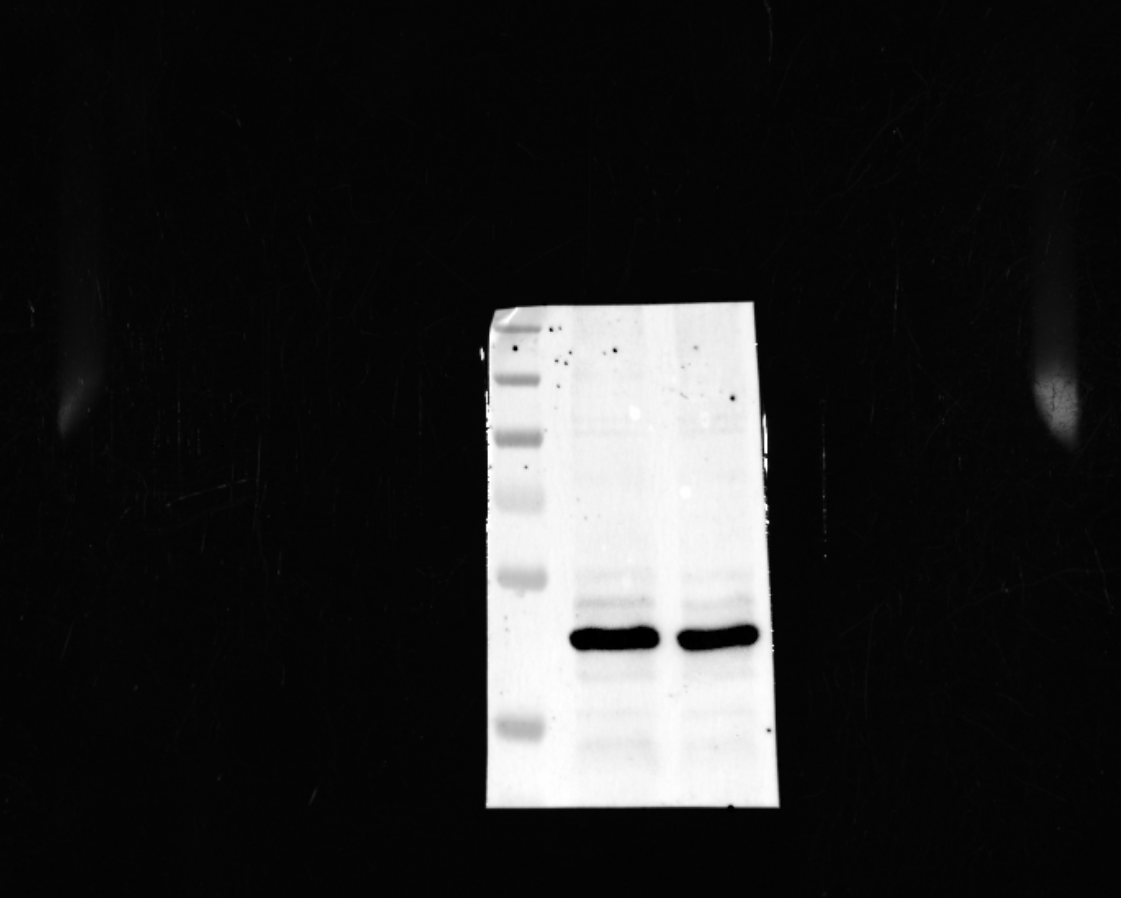

Supplement: Supplementary file 5 [file DataSheet6.zip › Capan2_CD44/TIF/CD44 2. Actin (#23.12 CD44 Aktin_3).tif]

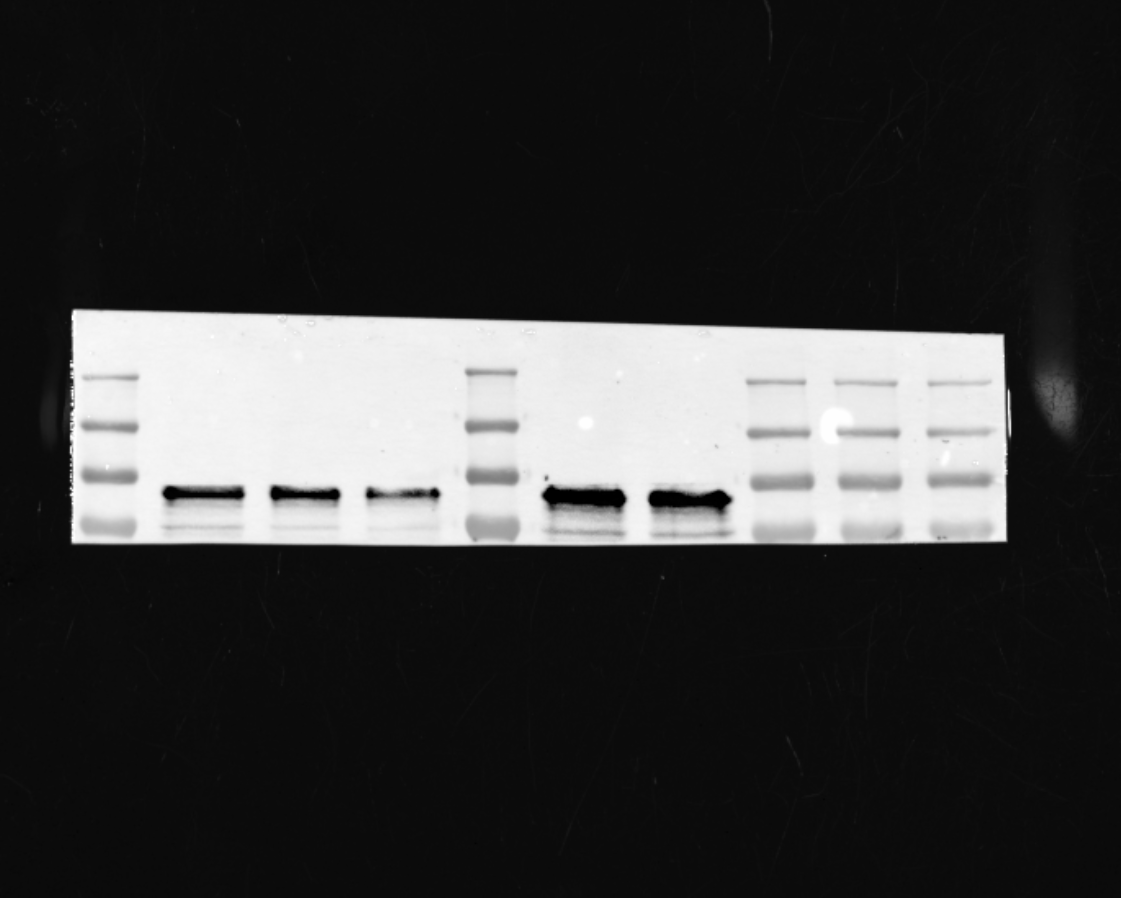

Supplement: Supplementary file 5 [file DataSheet6.zip › Capan2_CD44/TIF/CD44 3. (Viki 11.29. CD44_3).tif]

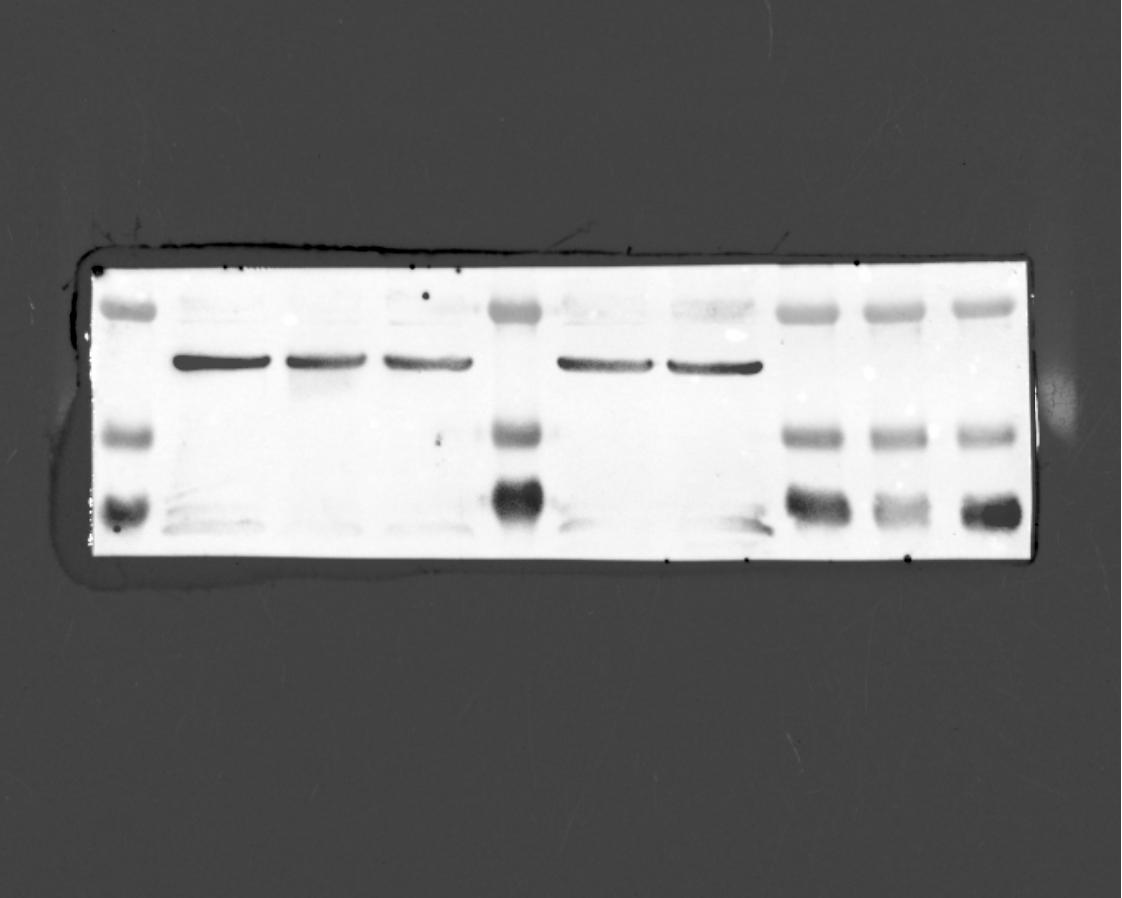

Supplement: Supplementary file 5 [file DataSheet6.zip › Capan2_CD44/TIF/CD44 3. Actin (Viki 11.30. CD44 B actin_3).tif]

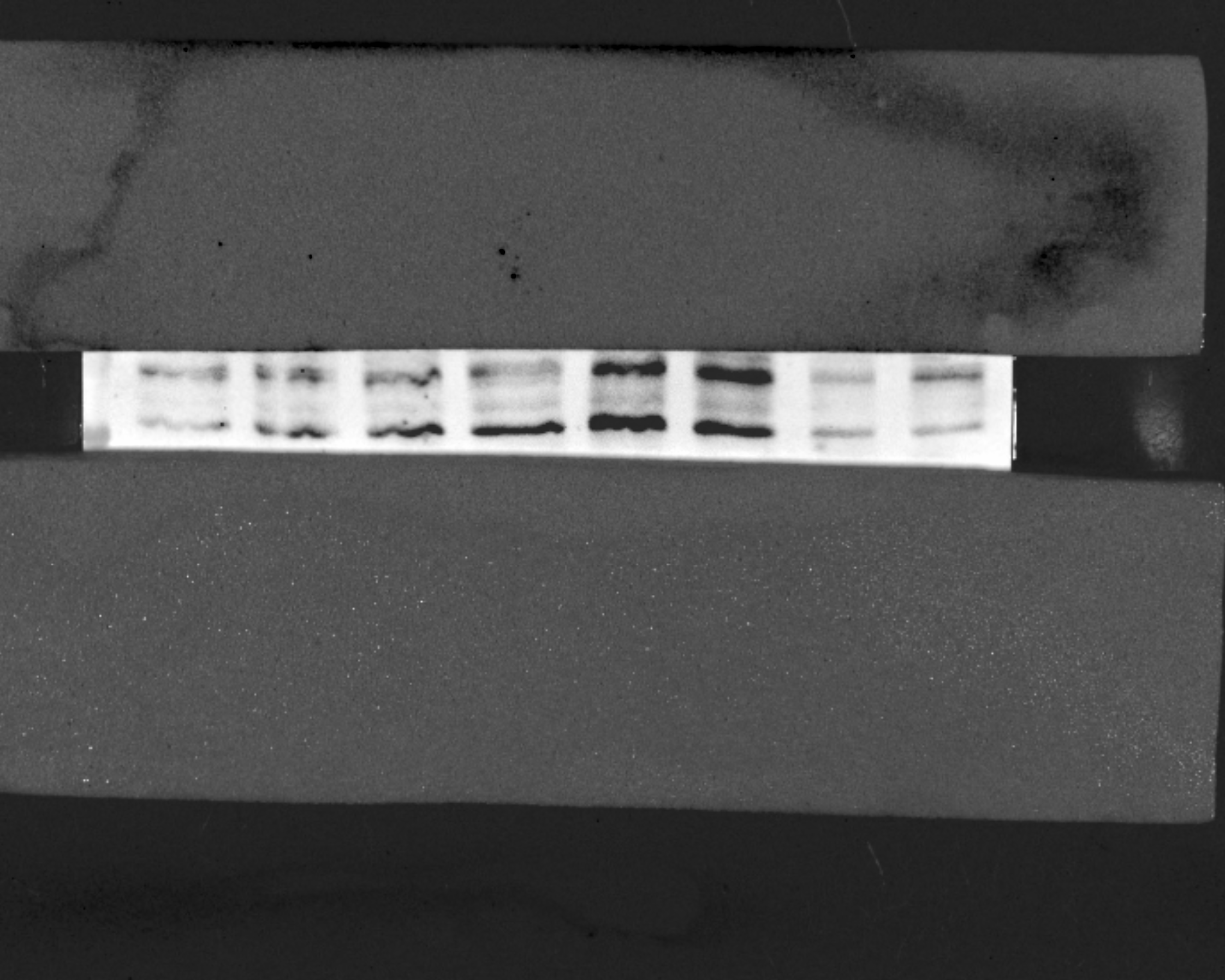

Supplement: Supplementary file 6 [file DataSheet2.zip › Capan2_Vimentin/TIF/Vimentin 1 2 (1017.vim0103).tif]

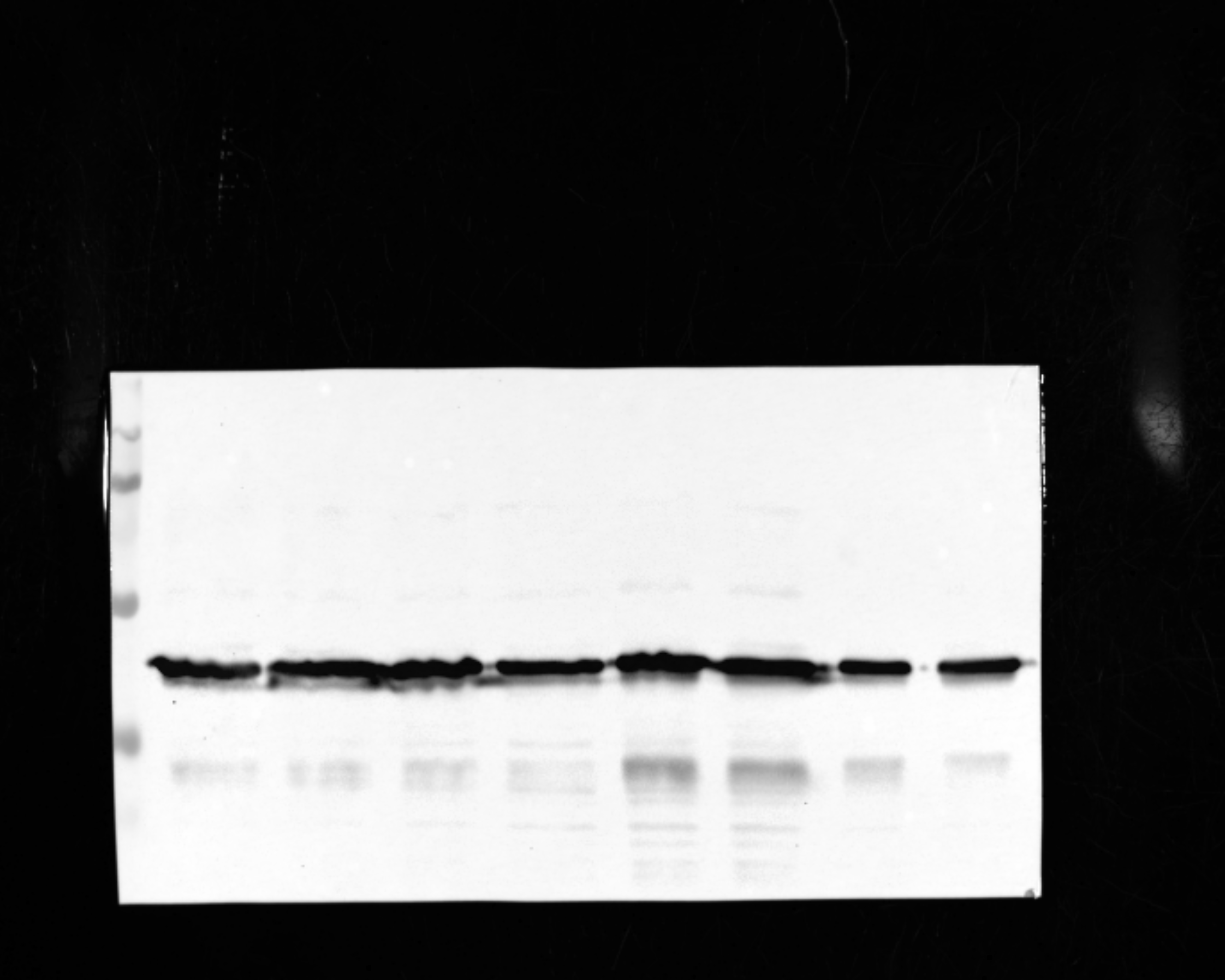

Supplement: Supplementary file 6 [file DataSheet2.zip › Capan2_Vimentin/TIF/Vimentin 1 2 Actin.tif]

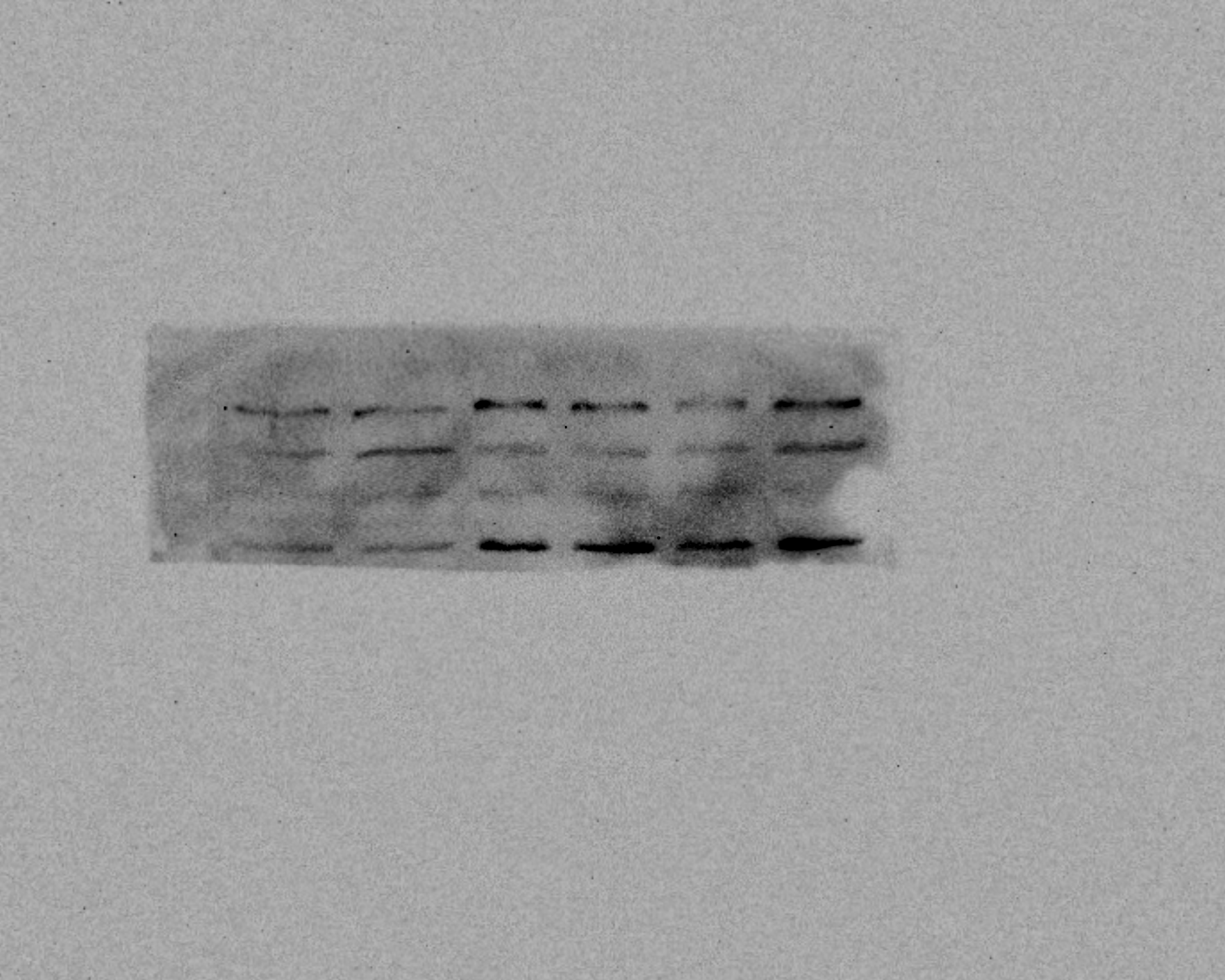

Supplement: Supplementary file 6 [file DataSheet2.zip › Capan2_Vimentin/TIF/Vimentin 3 (1017.vimIII).tif]

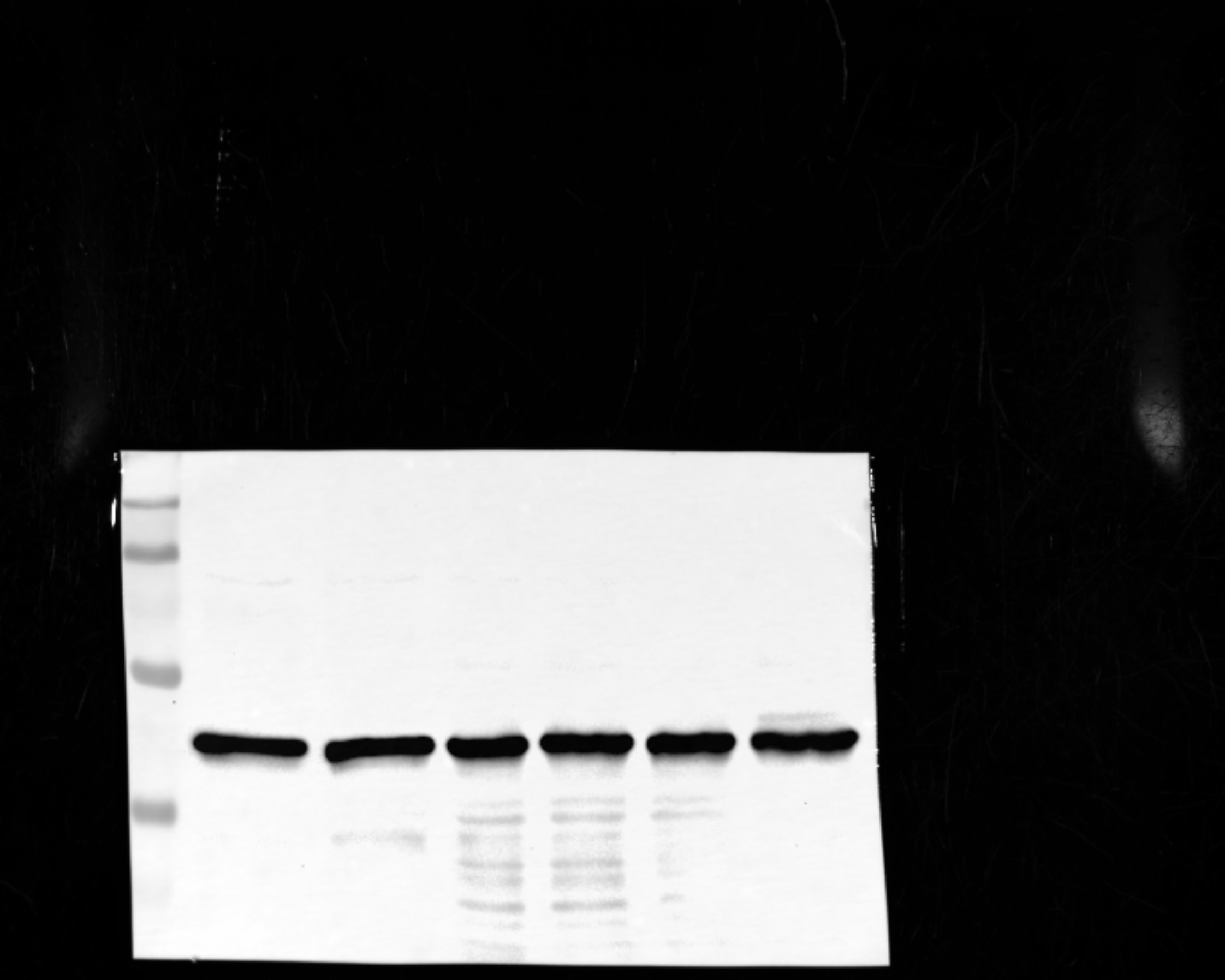

Supplement: Supplementary file 6 [file DataSheet2.zip › Capan2_Vimentin/TIF/Vimentin 3 4 Actin.tif]

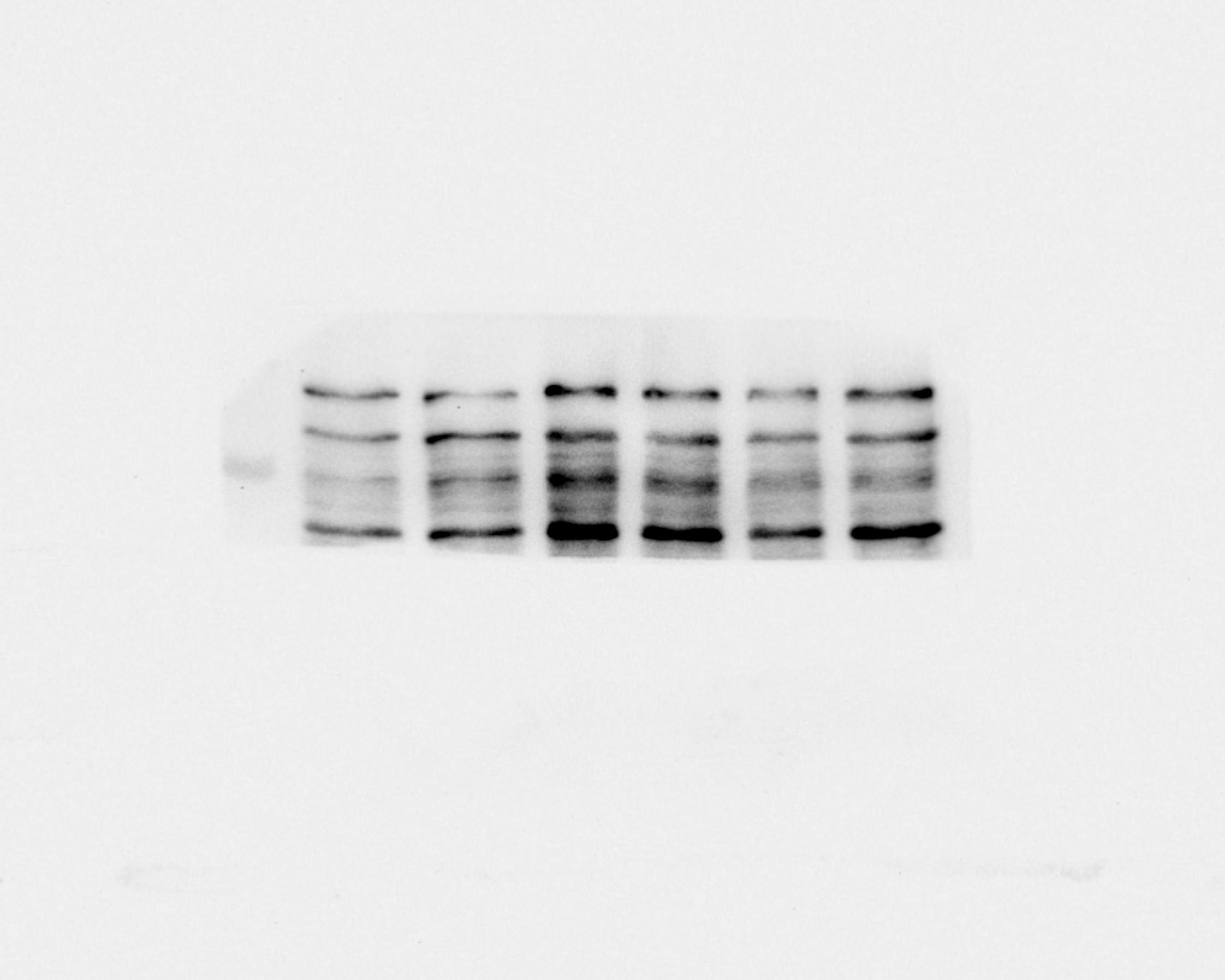

Supplement: Supplementary file 6 [file DataSheet2.zip › Capan2_Vimentin/TIF/Vimentin 4 (1017vimV).tif]

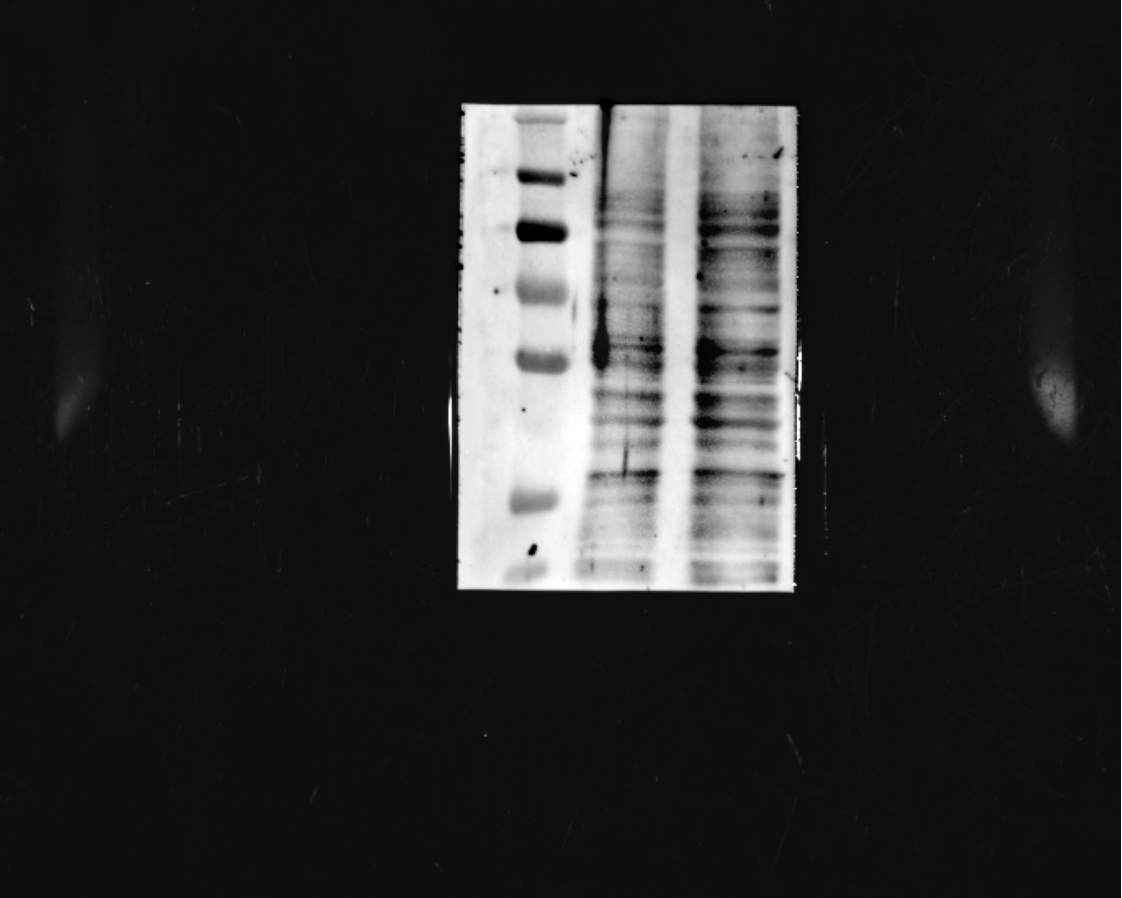

Supplement: Supplementary file 7 [file DataSheet5.zip › Capan2_4HNE/TIF/4HNE 1. (#22.30 Capan2 #22.27 4HNE_3).tif]

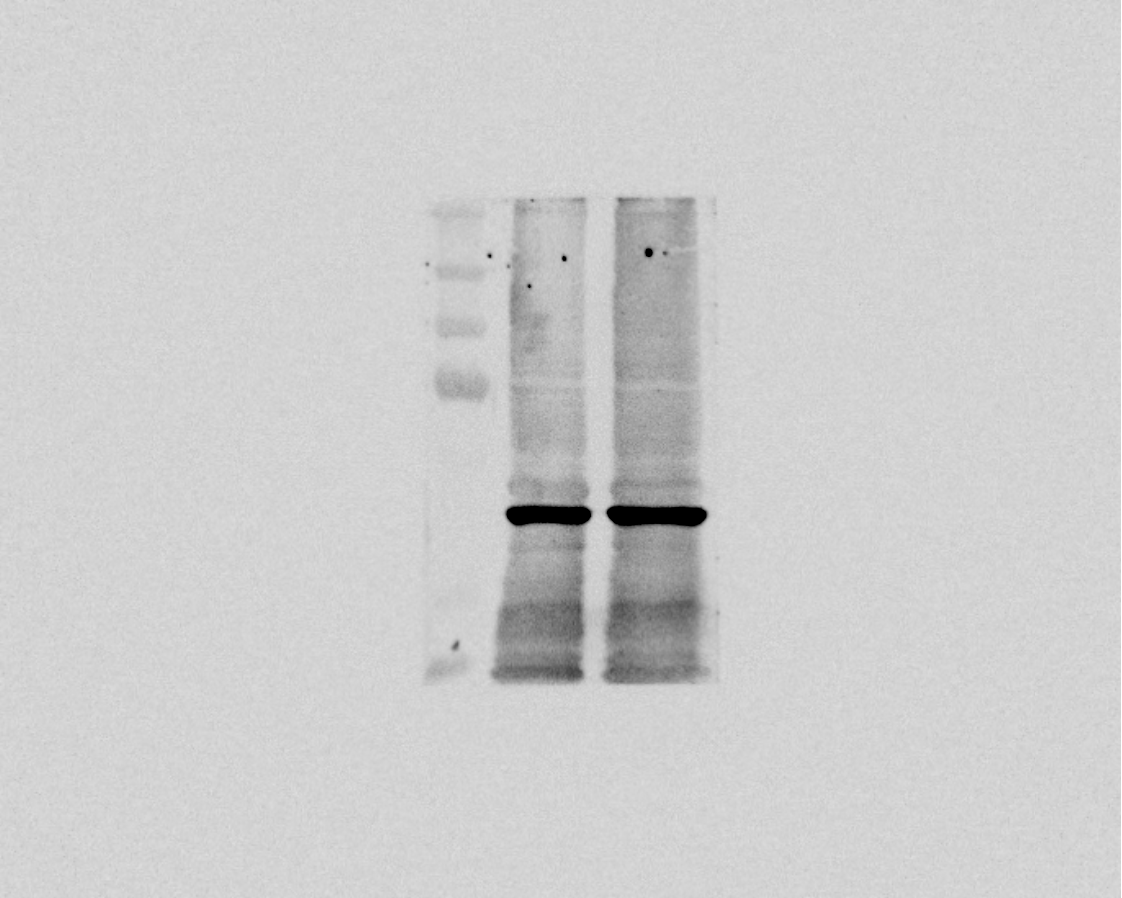

Supplement: Supplementary file 7 [file DataSheet5.zip › Capan2_4HNE/TIF/4HNE 1. Actin (#23.01Capan2 #22.27 4HNE Aktin_2).tif]

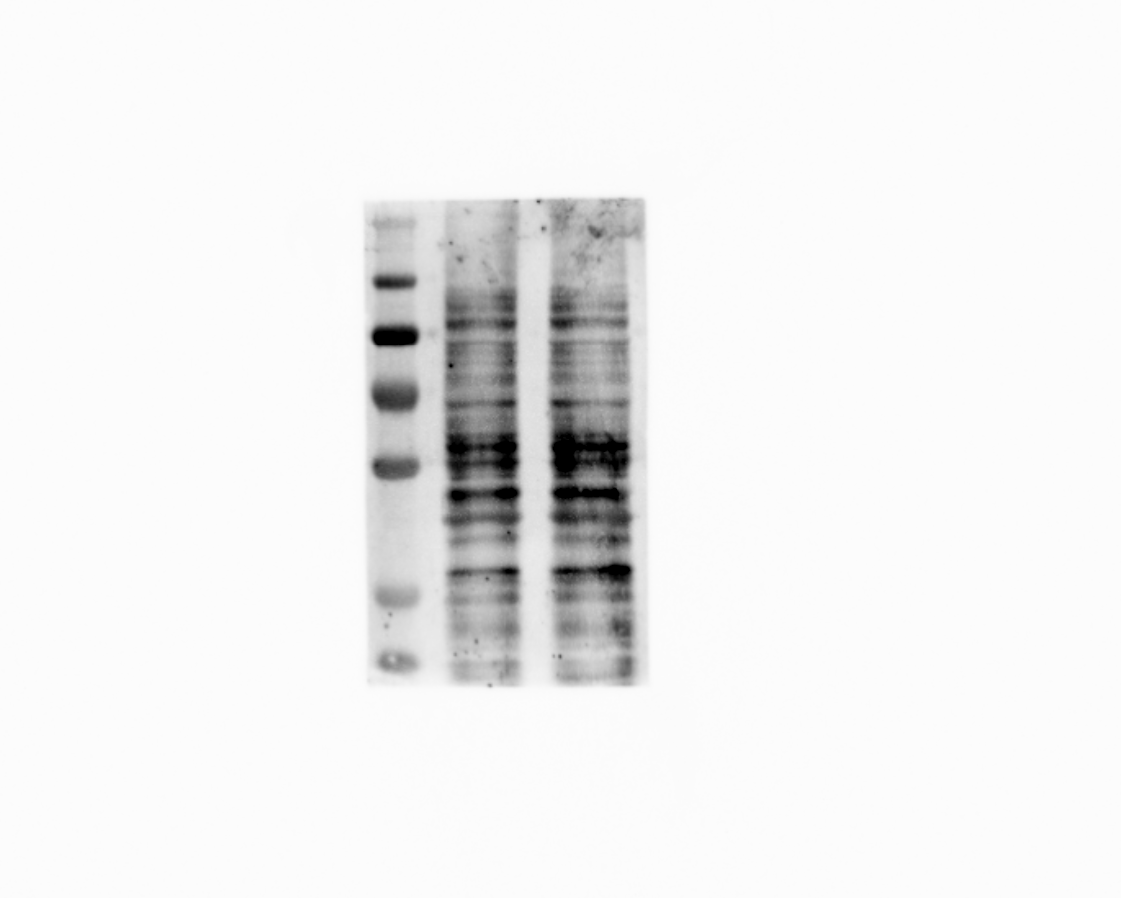

Supplement: Supplementary file 7 [file DataSheet5.zip › Capan2_4HNE/TIF/4HNE 2. (#22.30 Capan2 #22.28 4HNE_2).tif]

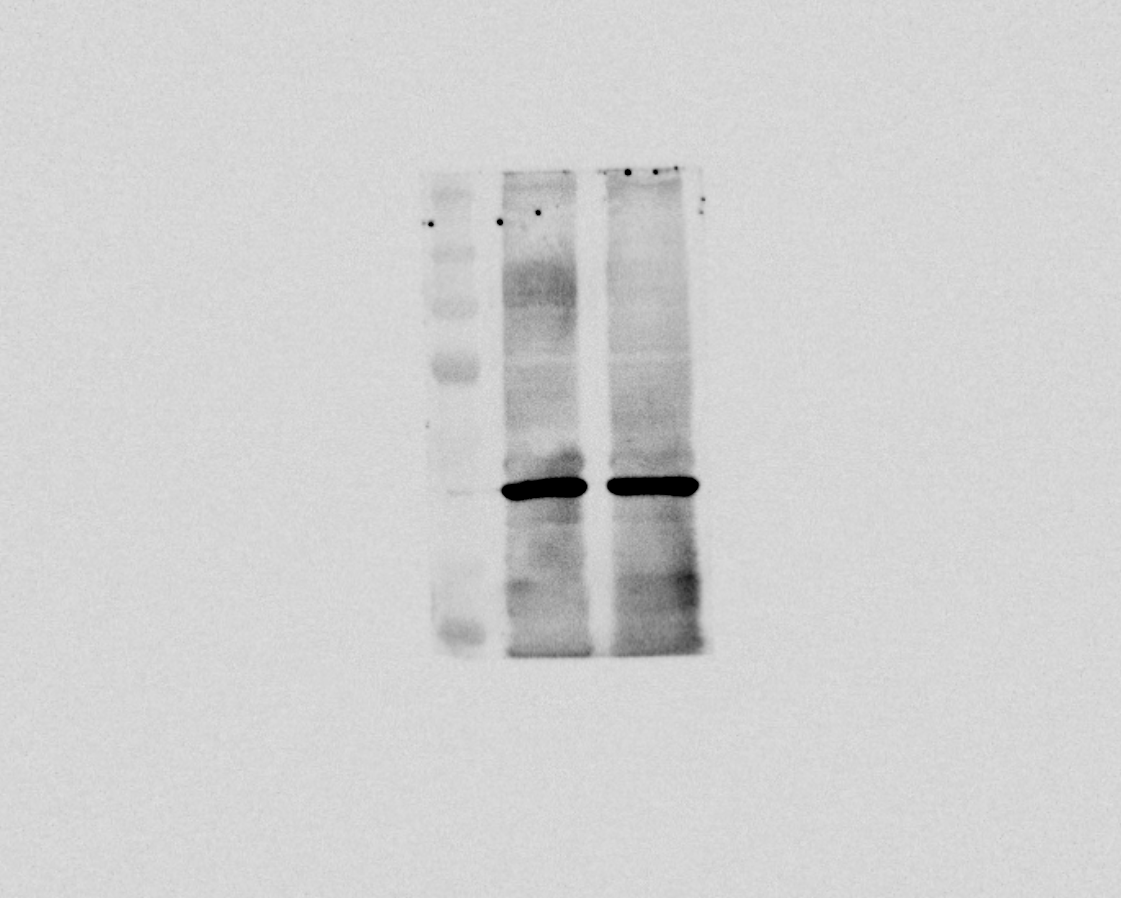

Supplement: Supplementary file 7 [file DataSheet5.zip › Capan2_4HNE/TIF/4HNE 2. Actin (#23.01Capan2 #22.28 4HNE Aktin_2).tif]

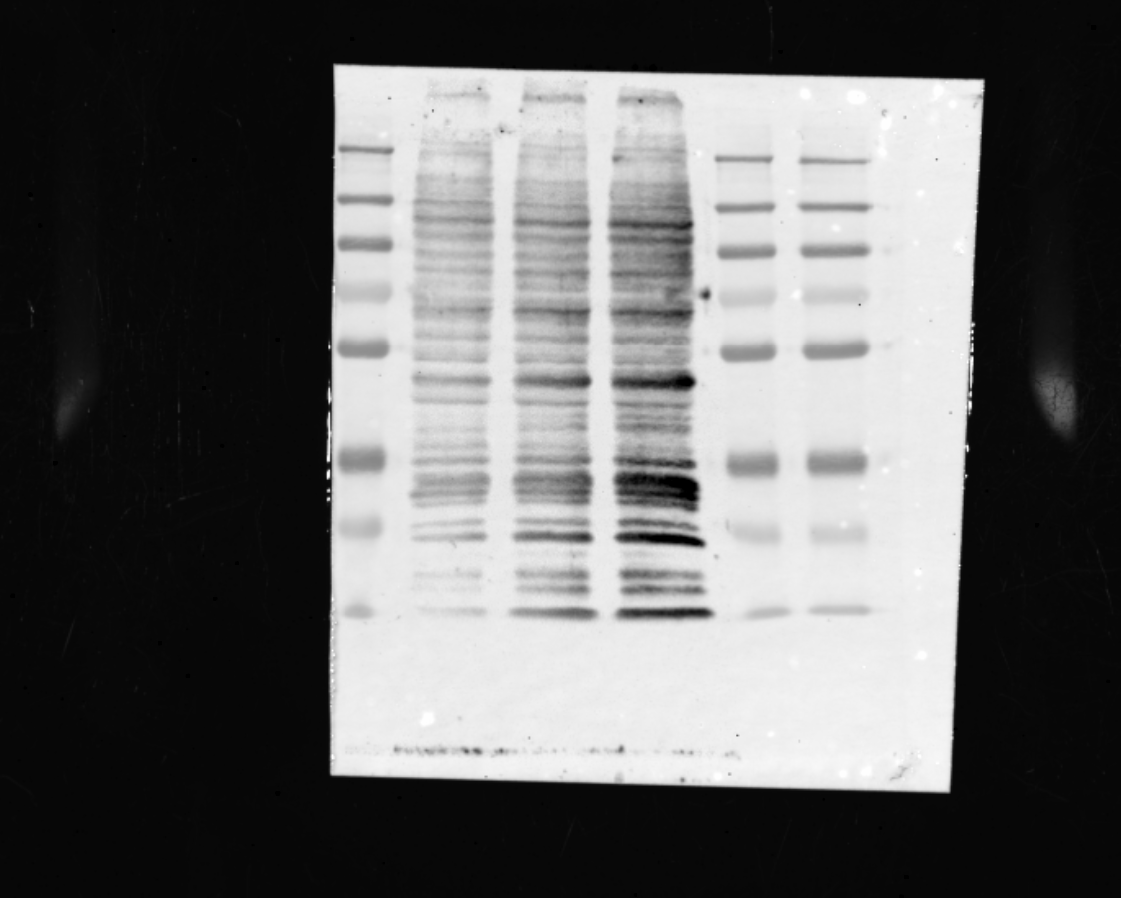

Supplement: Supplementary file 7 [file DataSheet5.zip › Capan2_4HNE/TIF/4HNE 3. (Viki 2023.10.05. 2. 4HNE_3).tif]

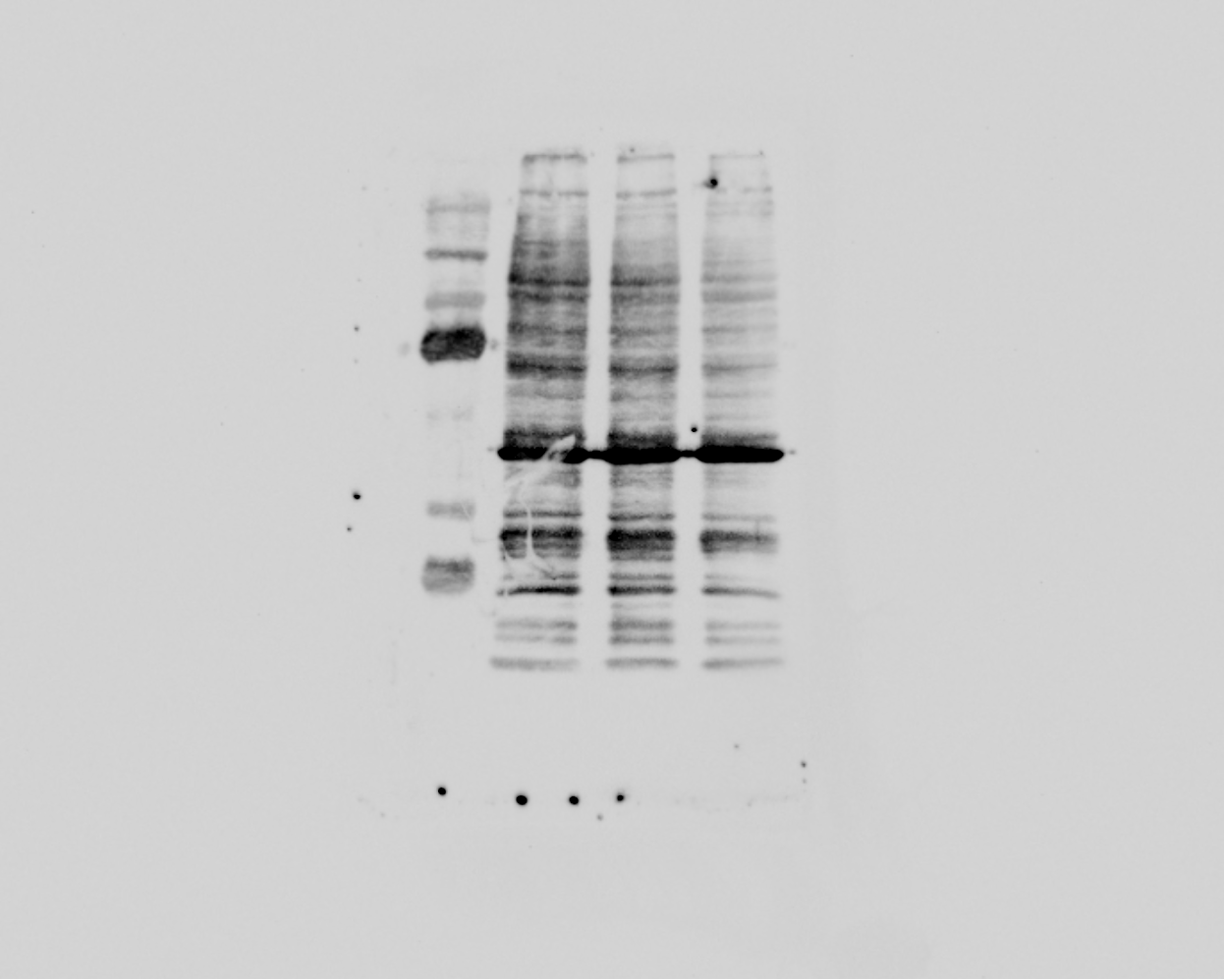

Supplement: Supplementary file 7 [file DataSheet5.zip › Capan2_4HNE/TIF/4HNE 3. Actin (Viki 2023.10.06. B actin 1. 4HNE_1).tif]

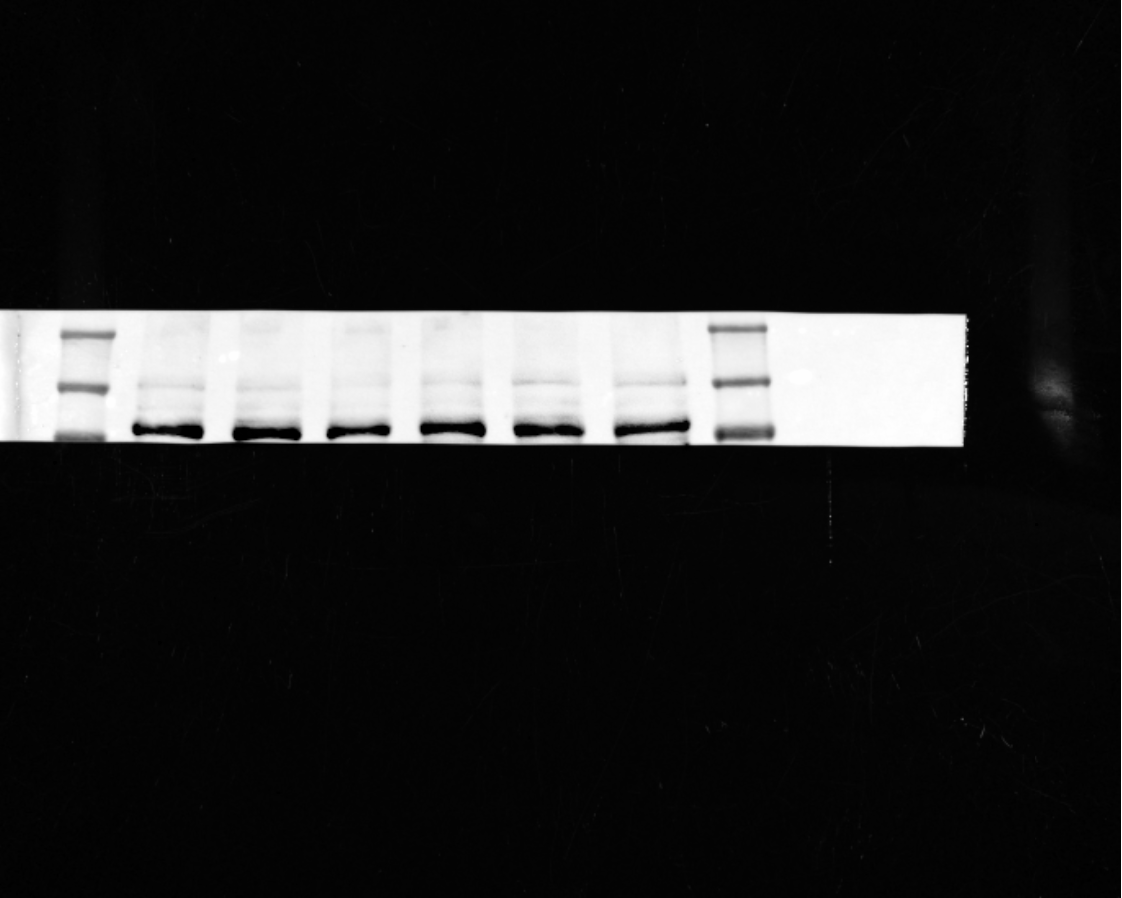

Supplement: Supplementary file 7 [file DataSheet5.zip › Capan2_iNOS/TIF/iNOS 1. (2020. 05. 27. Capan2 Edit I. sorozat iNOS_5).tif]

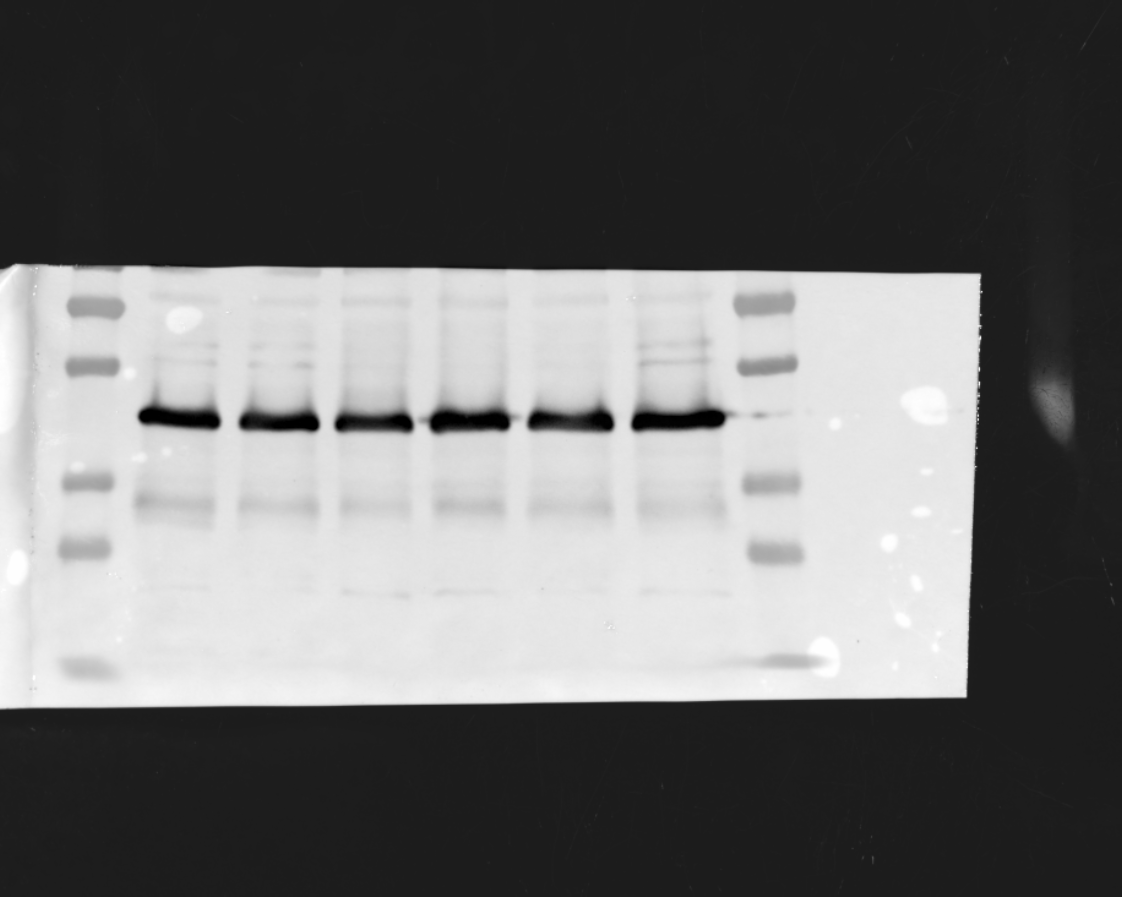

Supplement: Supplementary file 7 [file DataSheet5.zip › Capan2_iNOS/TIF/iNOS 1. Actin (2020. 05. 27. Aktin NRF2 (Abcam), iNOS_2+2020. 05. 27. Aktin NRF2 (Abcam), iNOS_1).tif]

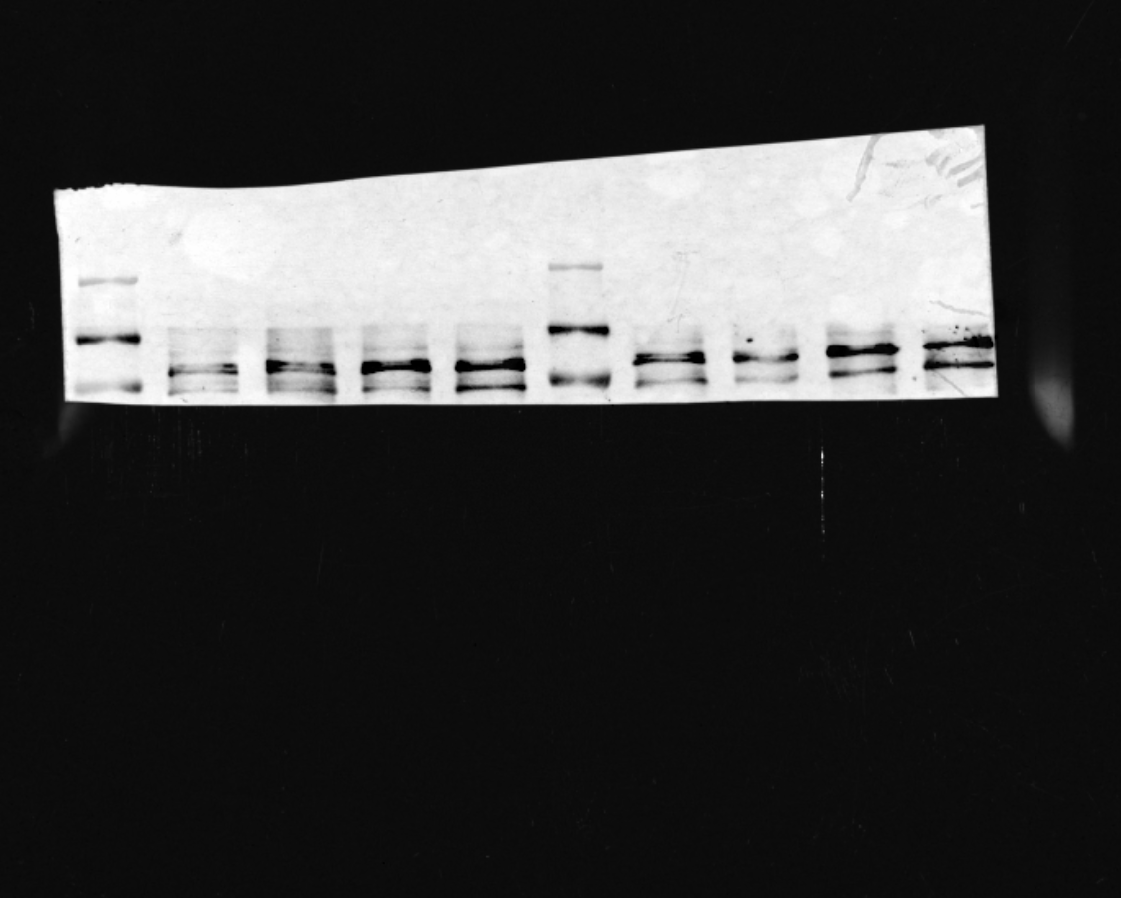

Supplement: Supplementary file 7 [file DataSheet5.zip › Capan2_iNOS/TIF/iNOS 2. (20181206 DCA UDCA 9.27. inos_4).tif]

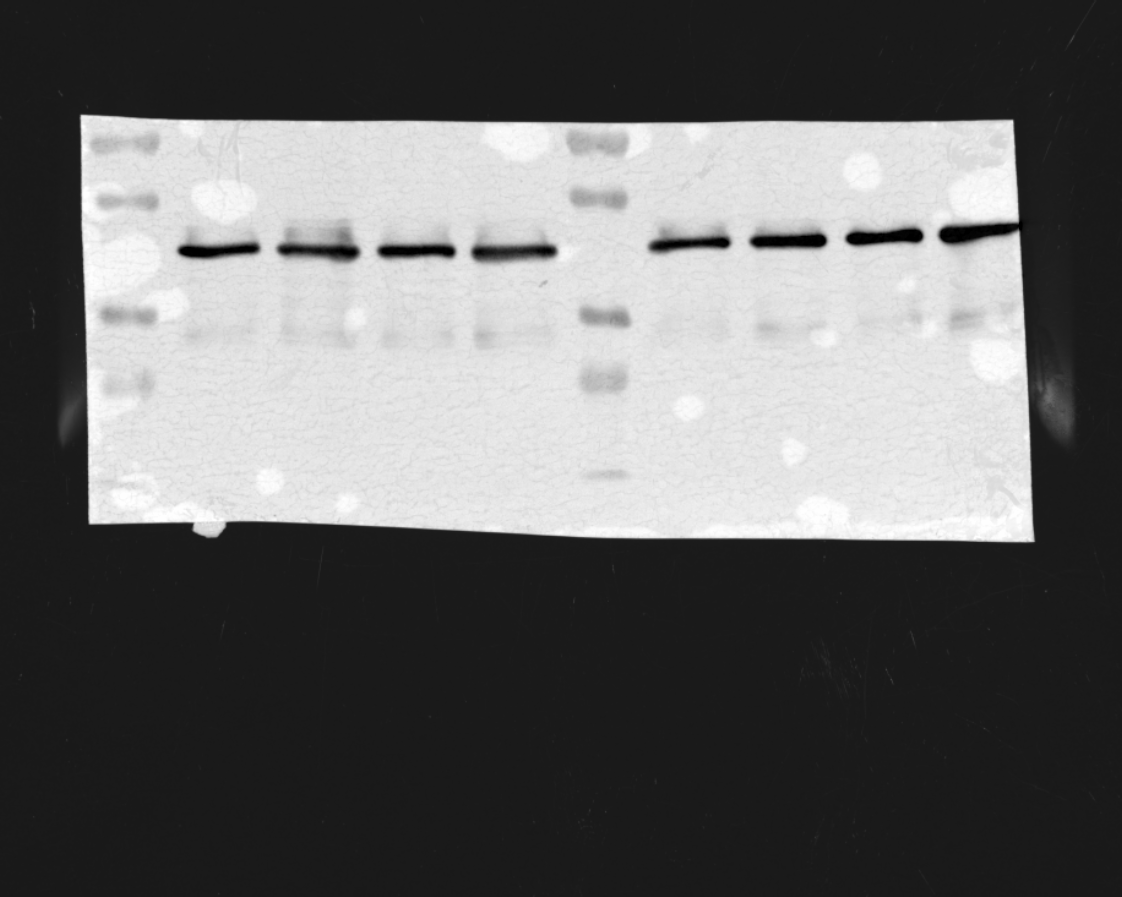

Supplement: Supplementary file 7 [file DataSheet5.zip › Capan2_iNOS/TIF/iNOS 2. Actin (20181207 DCA UDCA nrf2 9.27. aktin_3).tif]

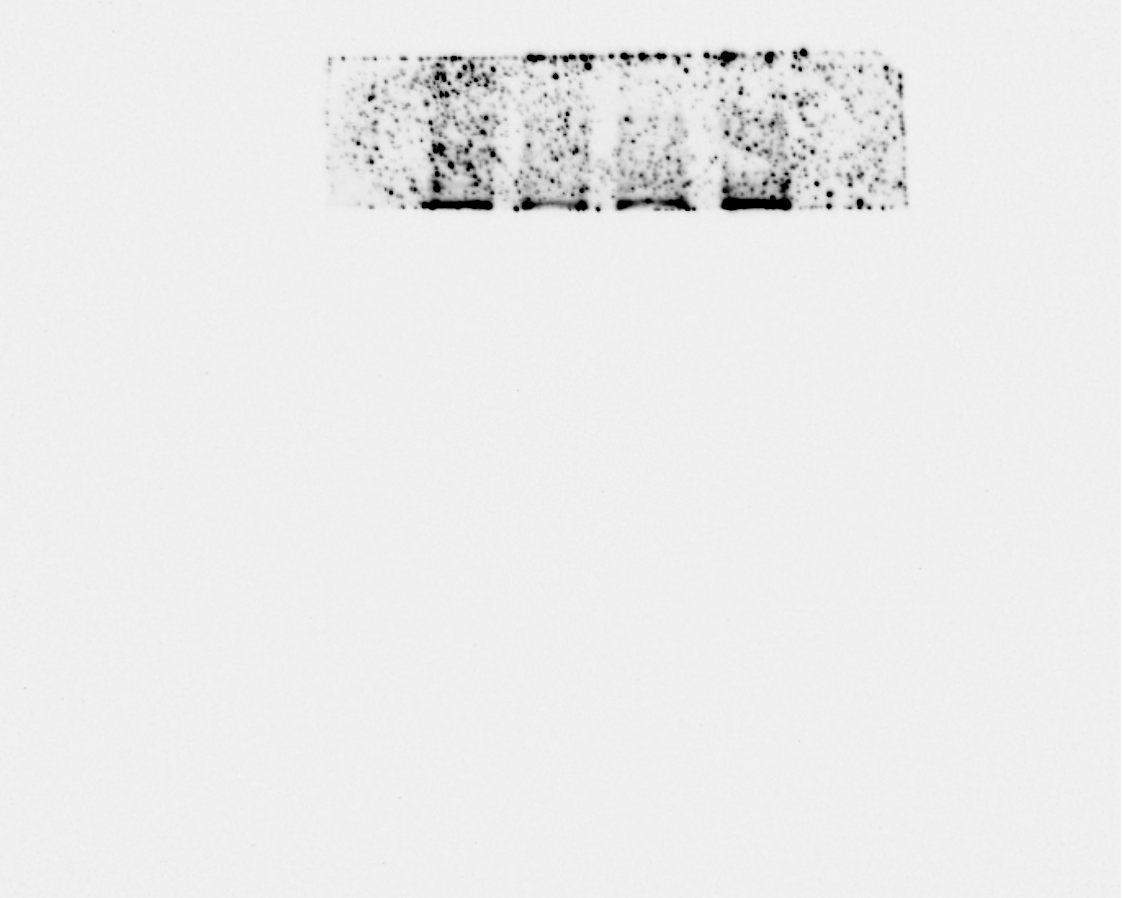

Supplement: Supplementary file 7 [file DataSheet5.zip › Capan2_iNOS/TIF/iNOS 3. (2021. 08. 18. Capan2 iNOS_1).tif]

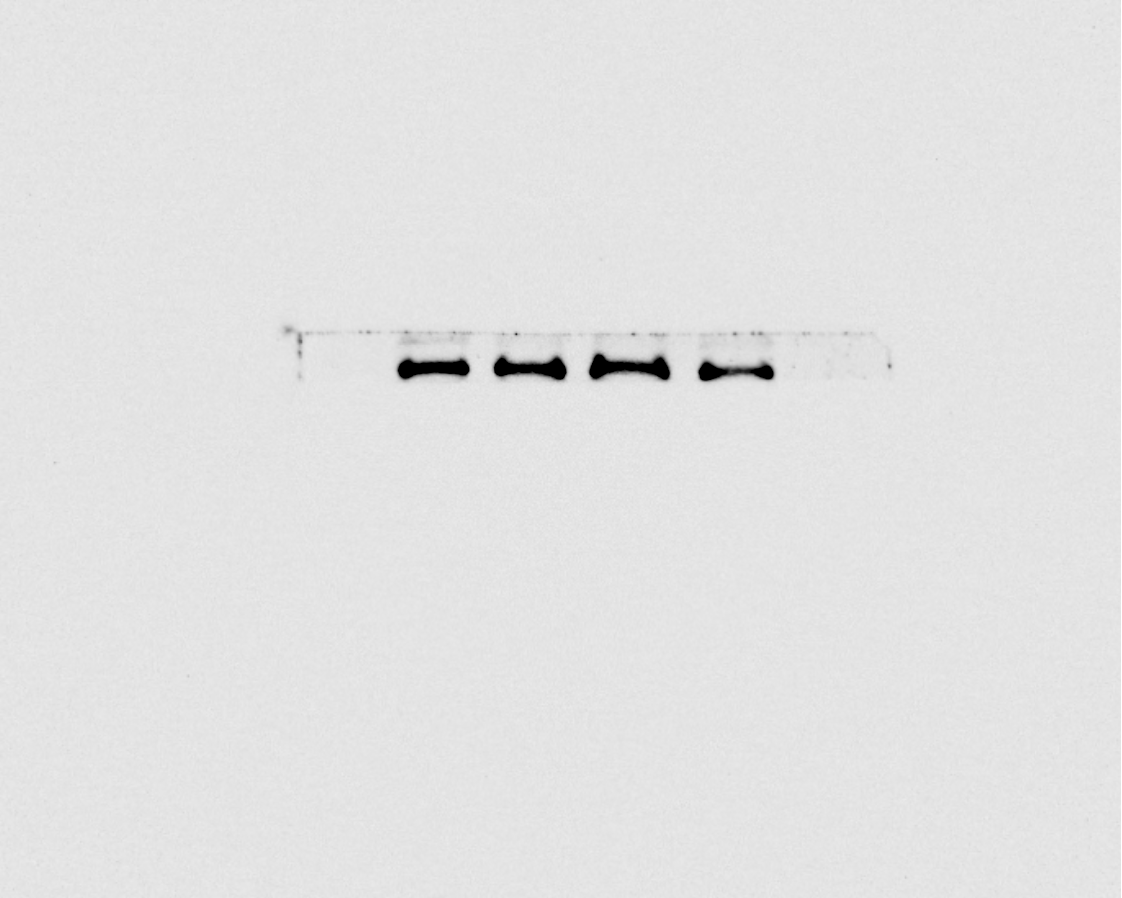

Supplement: Supplementary file 7 [file DataSheet5.zip › Capan2_iNOS/TIF/iNOS 3. Actin (2021. 08. 18. Capan2 Aktin iNOS, Keap1, Slug_2).tif]

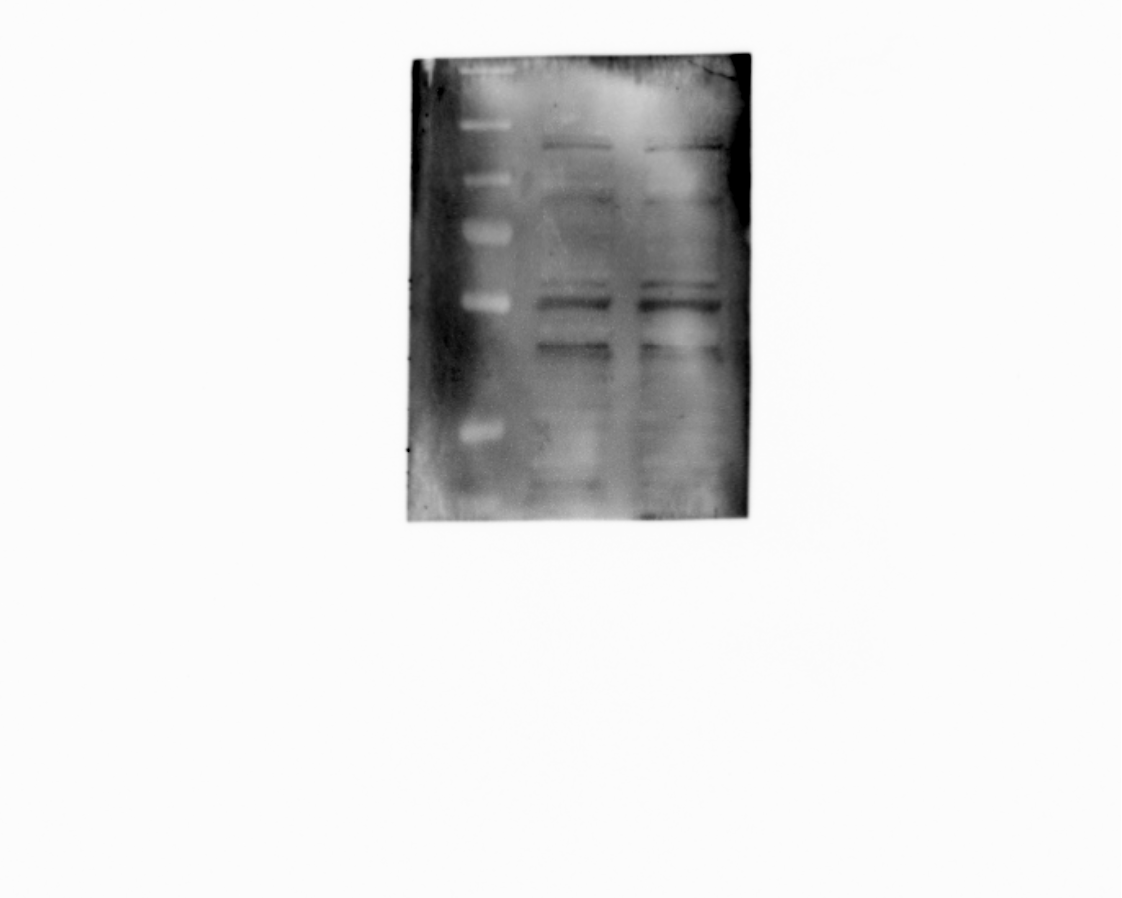

Supplement: Supplementary file 7 [file DataSheet5.zip › Capan2_Nitrotyrosine/TIF/Nitrotyrosine 1. (#22.30 Capan2 #22.27 Nitrotirozin_1).tif]

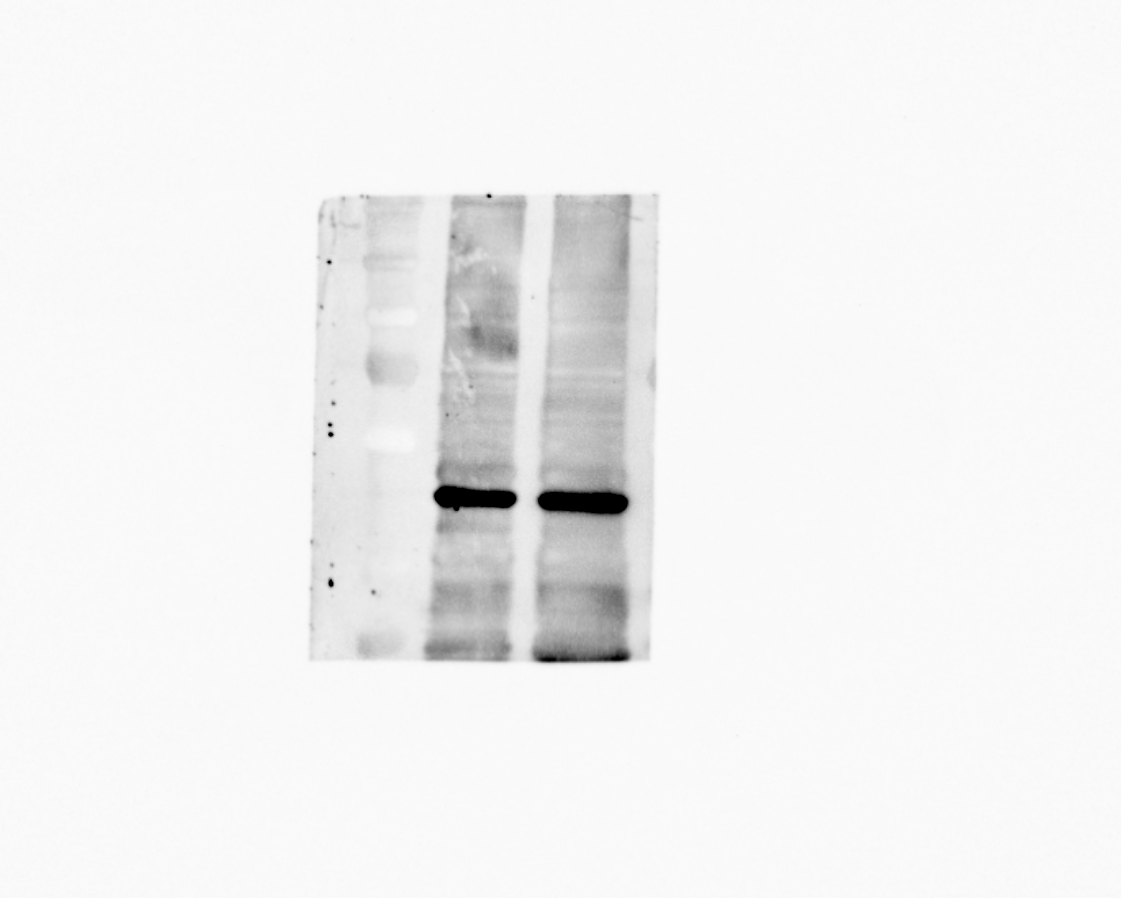

Supplement: Supplementary file 7 [file DataSheet5.zip › Capan2_Nitrotyrosine/TIF/Nitrotyrosine 1. Actin (#23.01Capan2 #22.27 Nitrotirozin Aktin_1).tif]

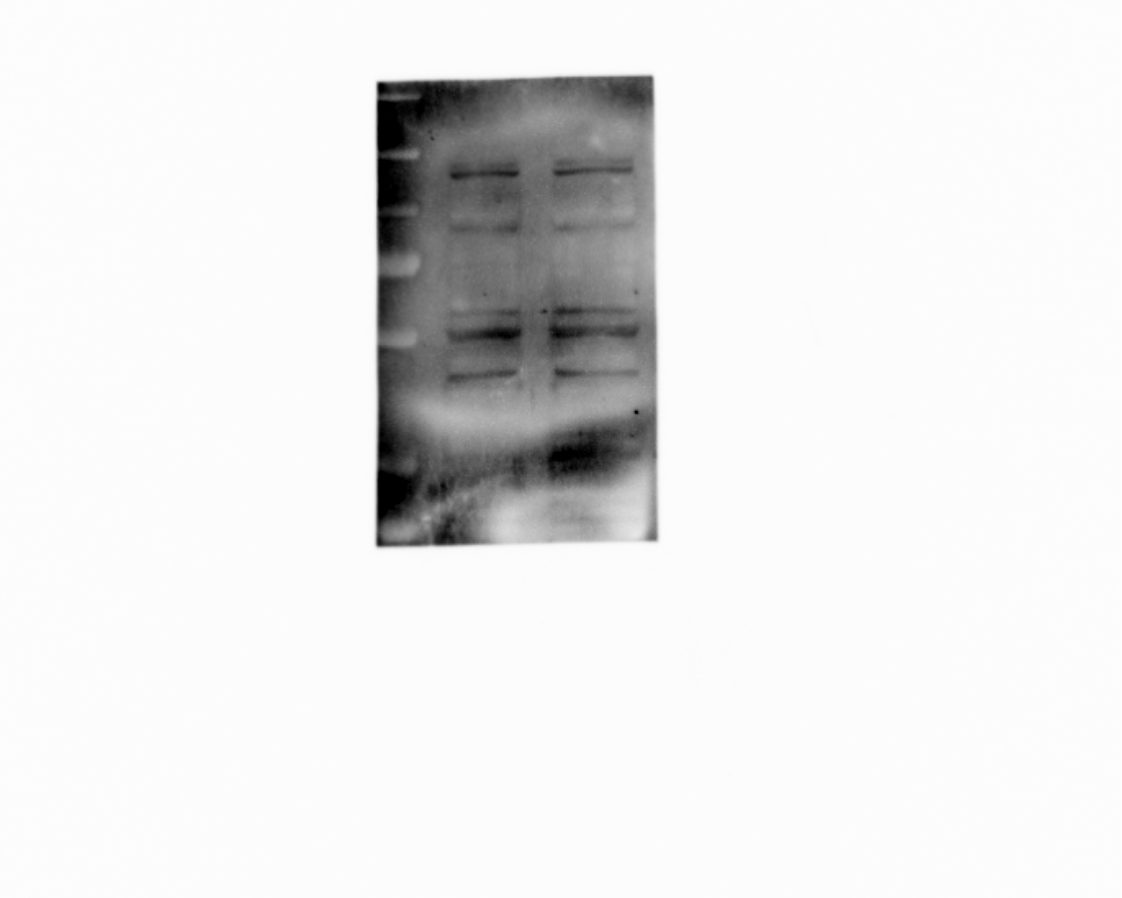

Supplement: Supplementary file 7 [file DataSheet5.zip › Capan2_Nitrotyrosine/TIF/Nitrotyrosine 2. (#22.30 Capan2 #22.28 Nitrotirozin_1).tif]

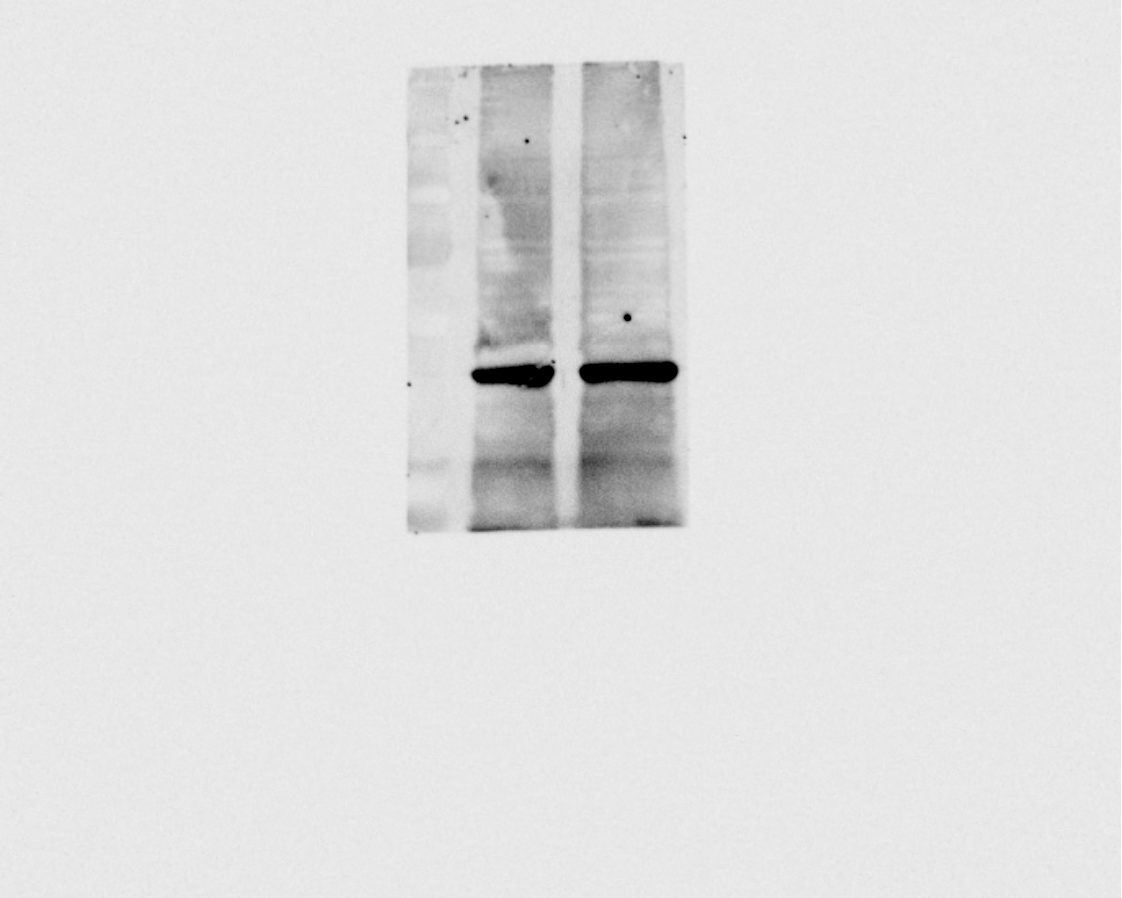

Supplement: Supplementary file 7 [file DataSheet5.zip › Capan2_Nitrotyrosine/TIF/Nitrotyrosine 2. Actin (#23.01Capan2 #22.28 Nitrotirozin Aktin_3).tif]

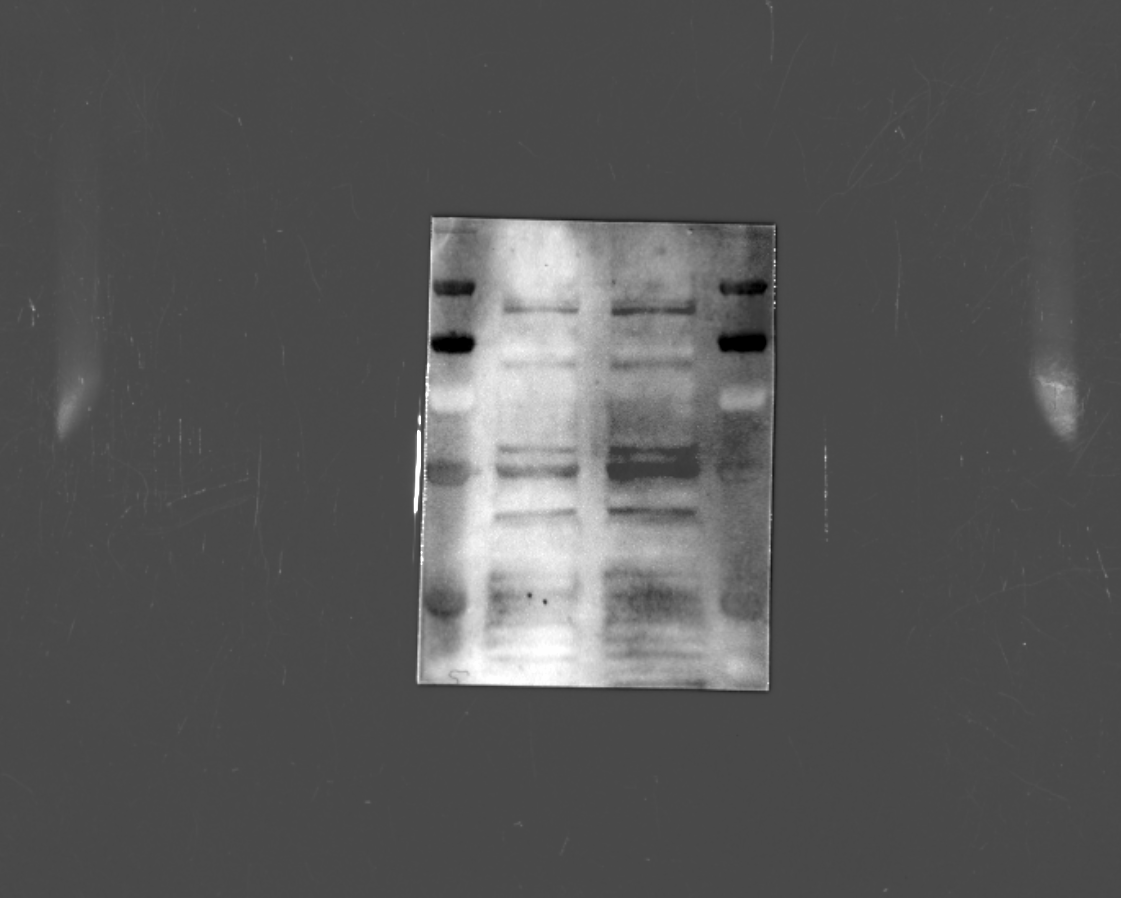

Supplement: Supplementary file 7 [file DataSheet5.zip › Capan2_Nitrotyrosine/TIF/Nitrotyrosine 3. (#22.30 Capan2 #22.29 Nitrotirozin_3).tif]

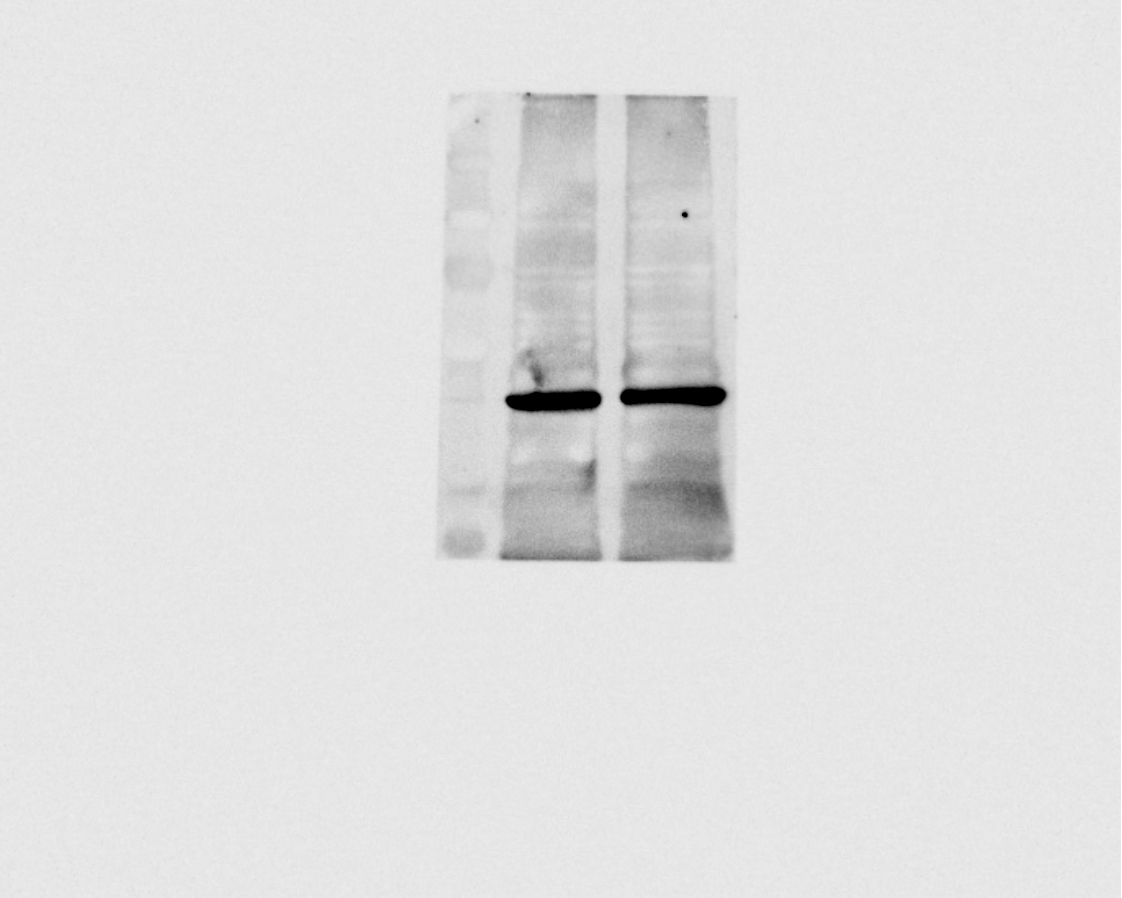

Supplement: Supplementary file 7 [file DataSheet5.zip › Capan2_Nitrotyrosine/TIF/Nitrotyrosine 3. Actin (#23.01Capan2 #22.29 Nitrotirozin Aktin_2).tif]

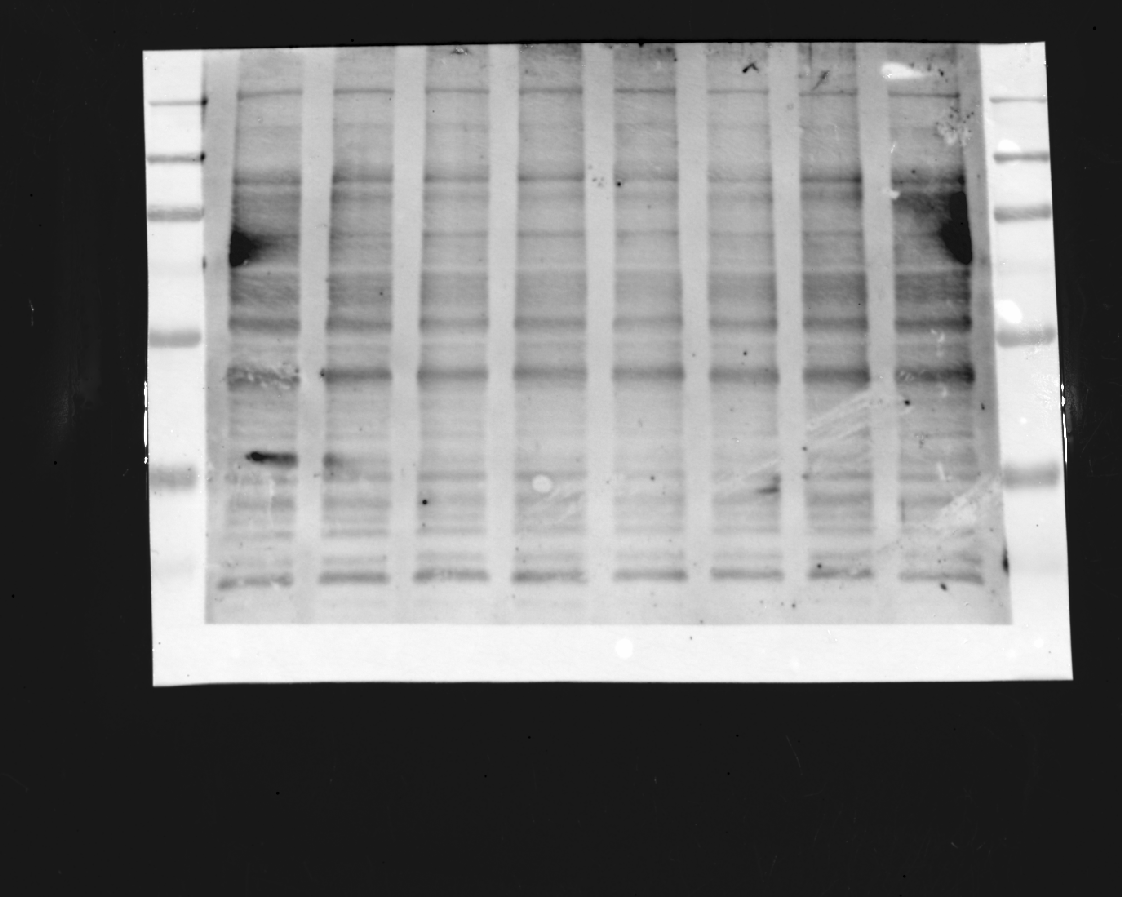

Supplement: Supplementary file 7 [file DataSheet5.zip › Capan2_Nitrotyrosine/TIF/Nitrotyrosine 4. (2023.07.20. Viki Nitrotirosyne 1. membran_7).tif]

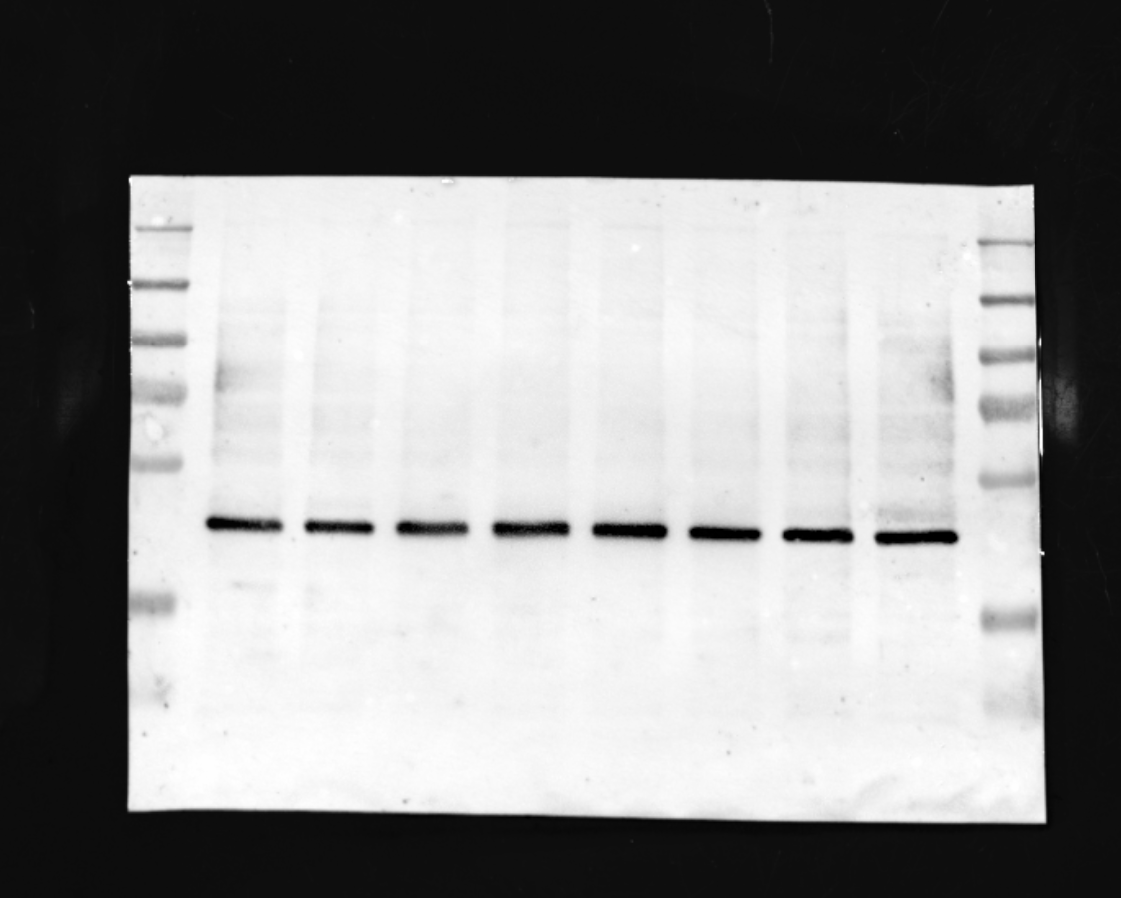

Supplement: Supplementary file 7 [file DataSheet5.zip › Capan2_Nitrotyrosine/TIF/Nitrotyrosine 4. Actin (2023.07.21. Viki B Aktin 1. membran_3).tif]

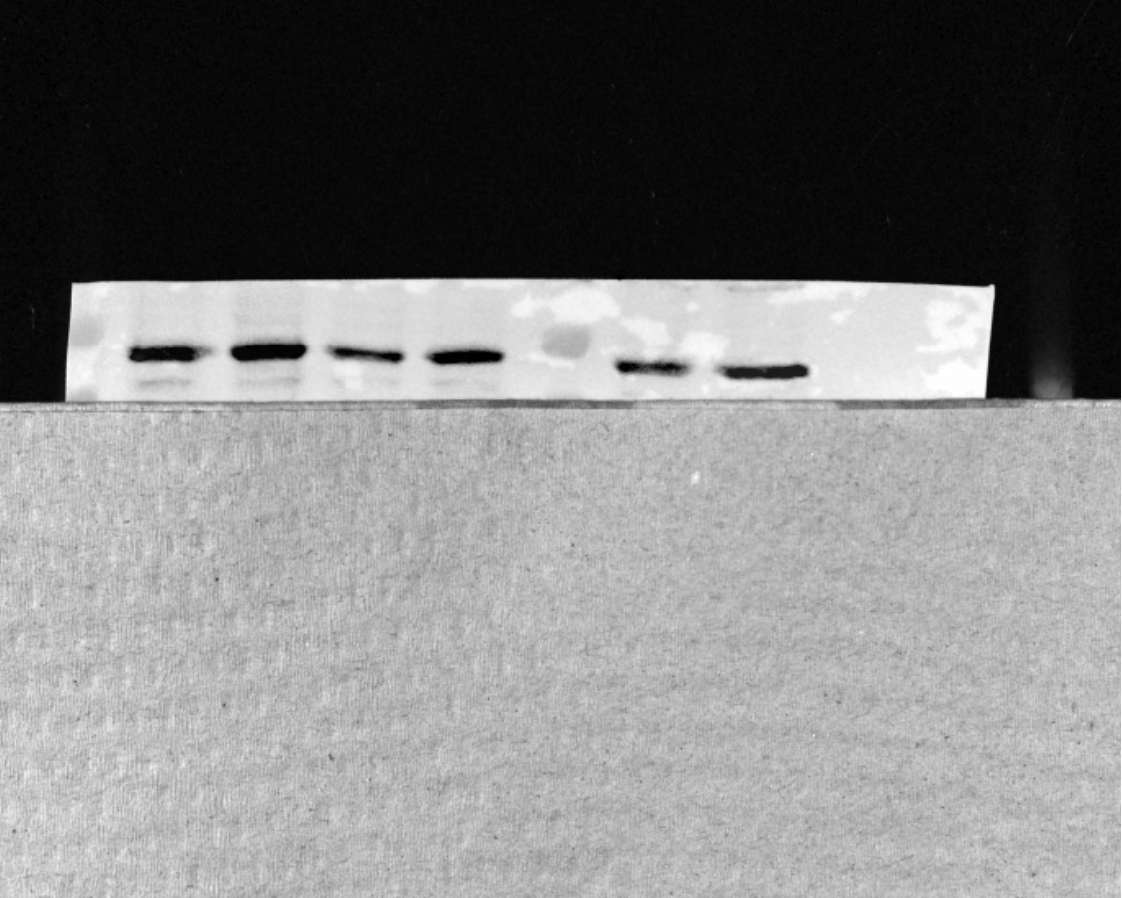

Supplement: Supplementary file 7 [file DataSheet5.zip › Capan2_NRF2/TIF/NRF2 1. (20190410 Capan2 ME1 sor NRF2_5).tif]

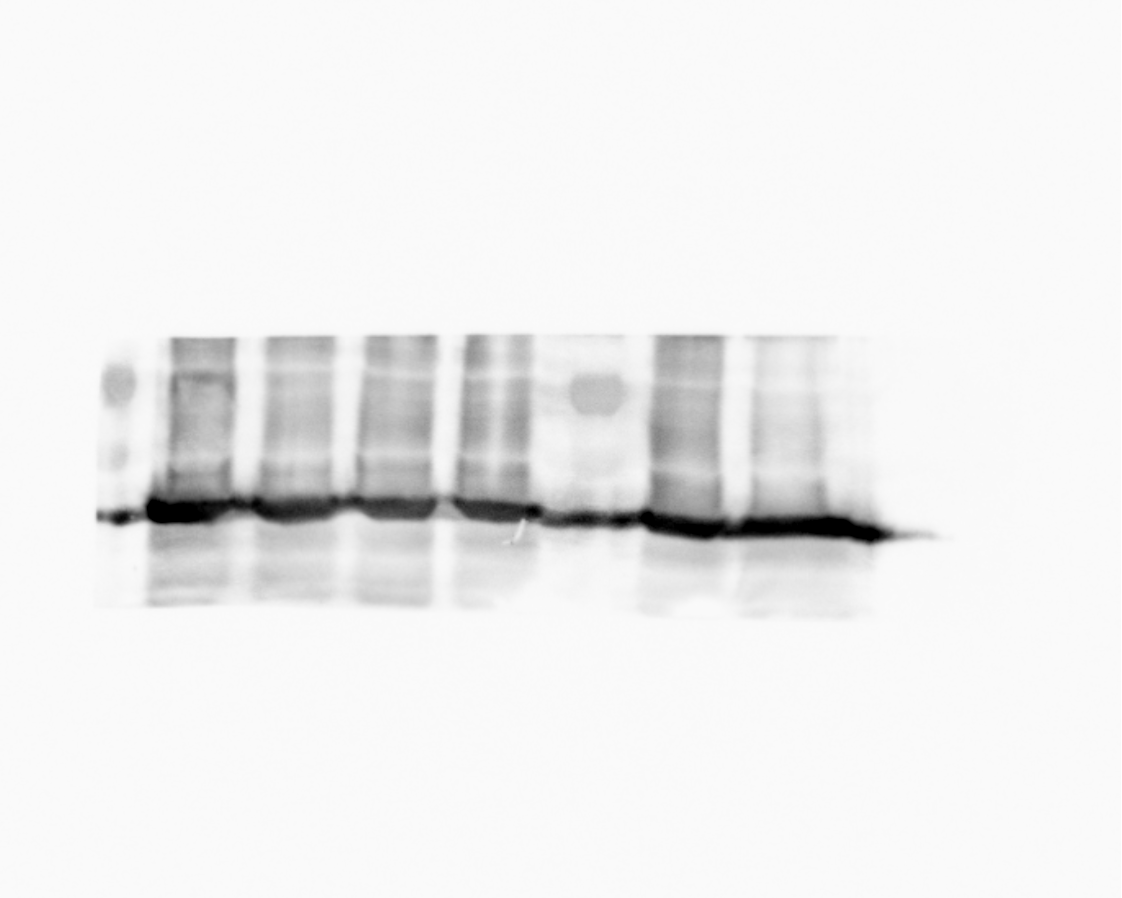

Supplement: Supplementary file 7 [file DataSheet5.zip › Capan2_NRF2/TIF/NRF2 1. Actin (2019-04-11 11hr 42min 11sec).tif]

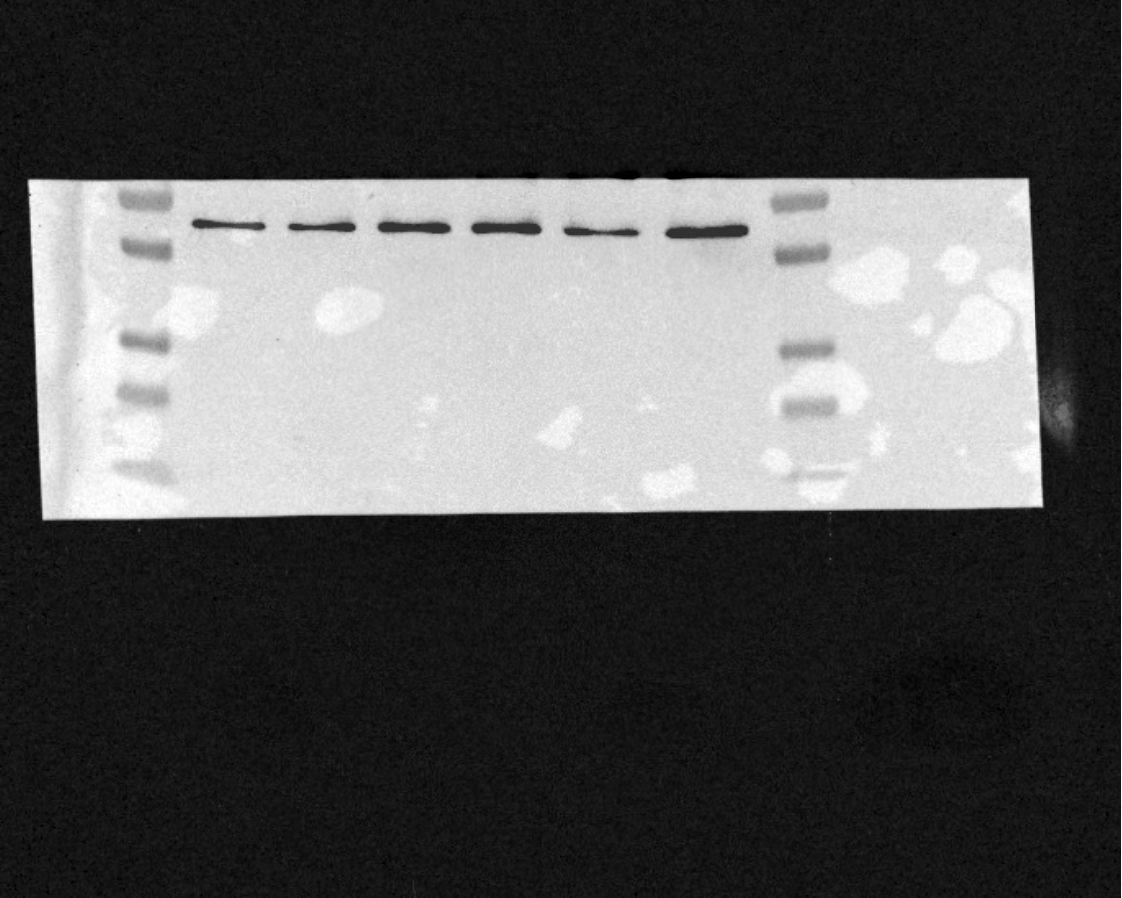

Supplement: Supplementary file 7 [file DataSheet5.zip › Capan2_NRF2/TIF/NRF2 2. (2020. 06. 03. Edit II. sorozat NRF2 (Abcam)_4+2020. 06. 03. Edit II. sorozat NRF2 (Abcam)_6).tif]

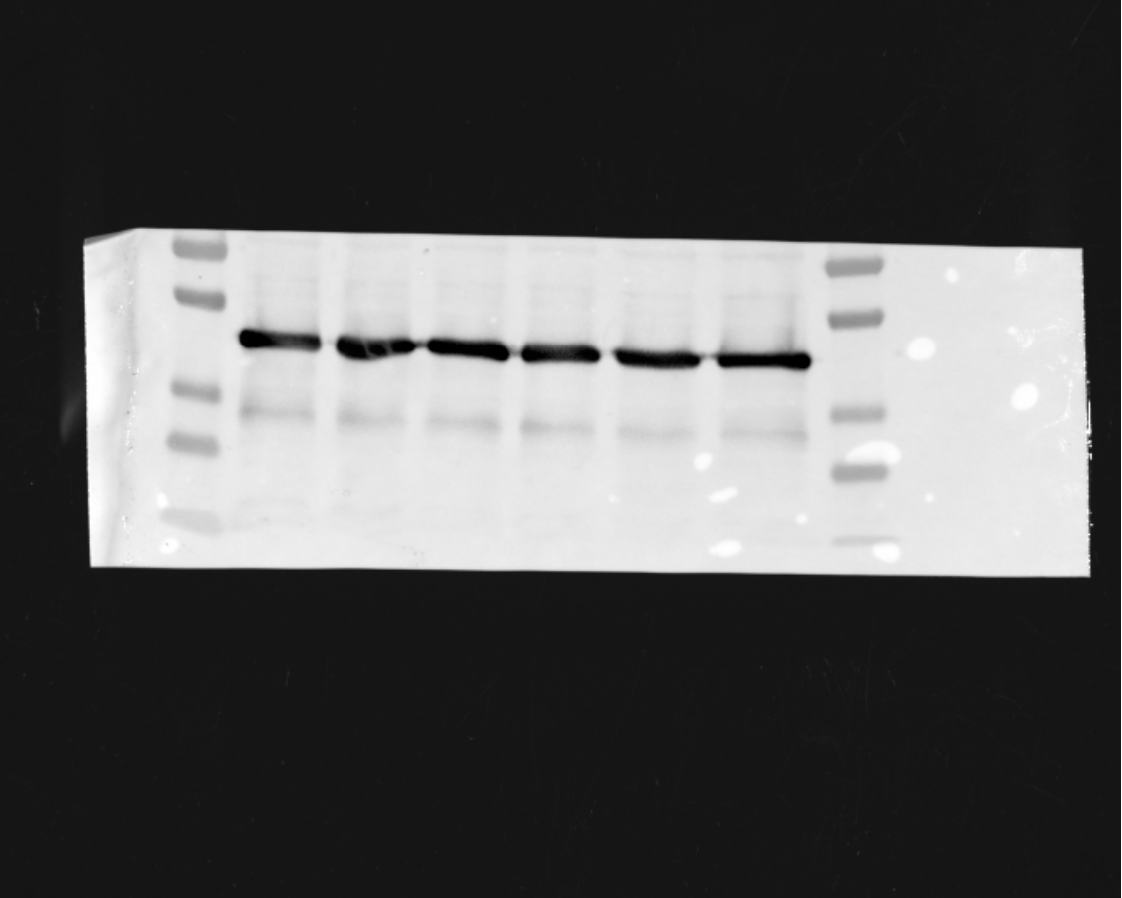

Supplement: Supplementary file 7 [file DataSheet5.zip › Capan2_NRF2/TIF/NRF2 2. Actin (2020. 06. 04. Edit II.sorozat Aktin NRF2 (Abcam), iNOS_4).tif]

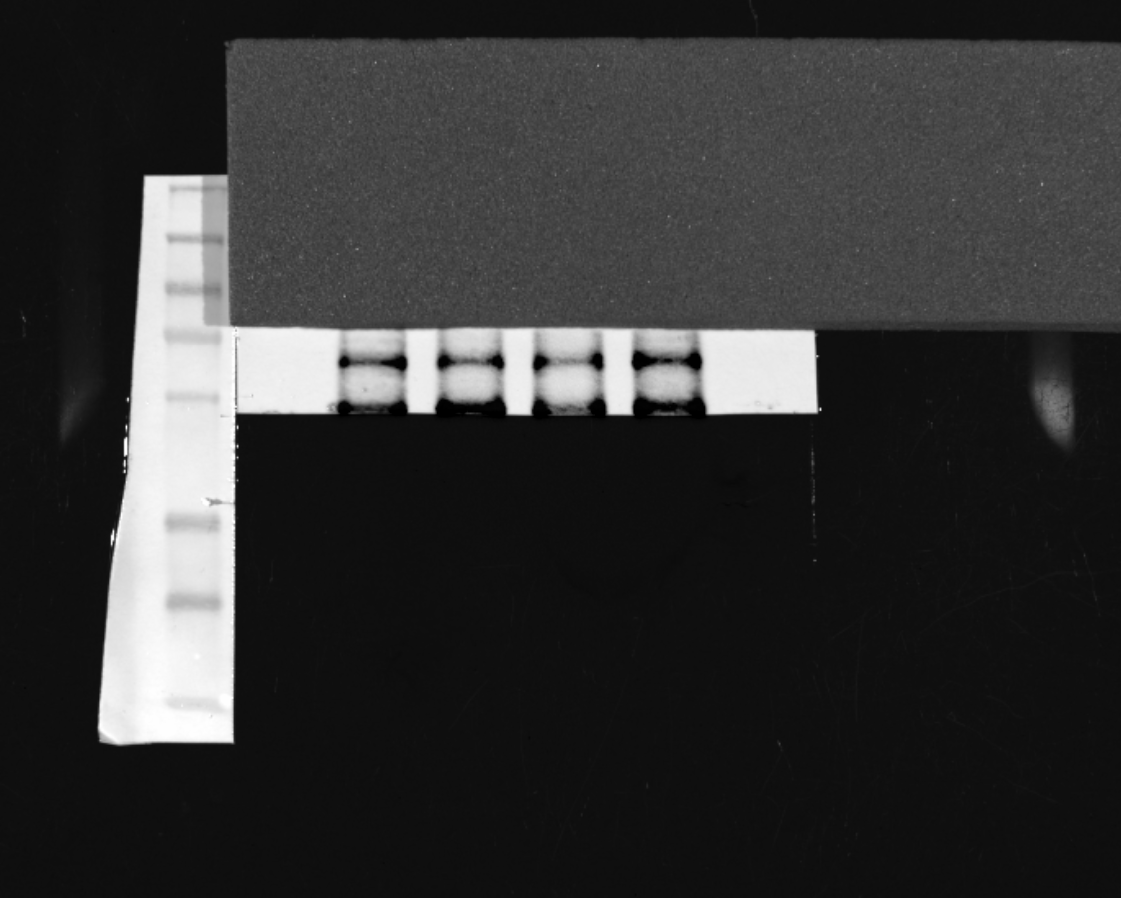

Supplement: Supplementary file 7 [file DataSheet5.zip › Capan2_NRF2/TIF/NRF2 3. (2021. 08. 18. Capan2 NRF2_7).tif]

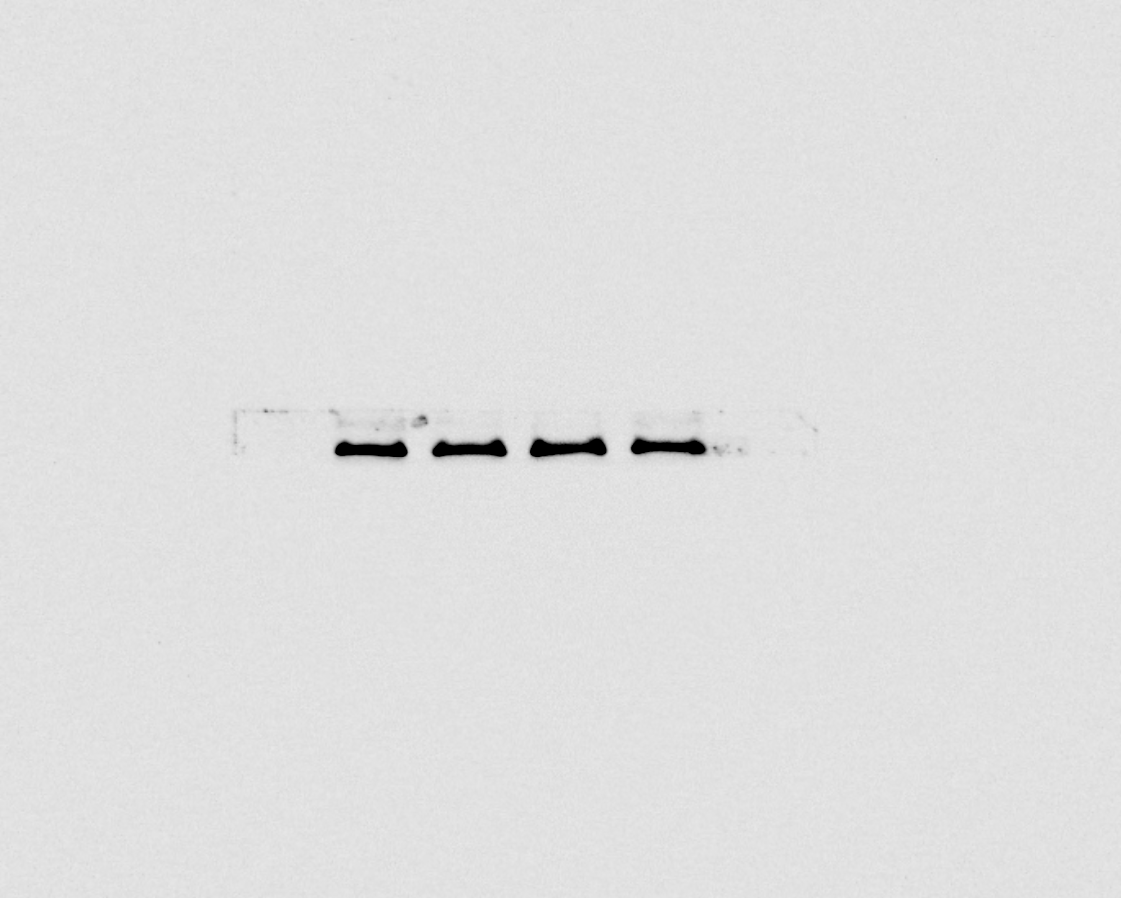

Supplement: Supplementary file 7 [file DataSheet5.zip › Capan2_NRF2/TIF/NRF2 3. Actin (2021. 08. 18. Capan2 Aktin ZO1, NRF2, Snai_1).tif]

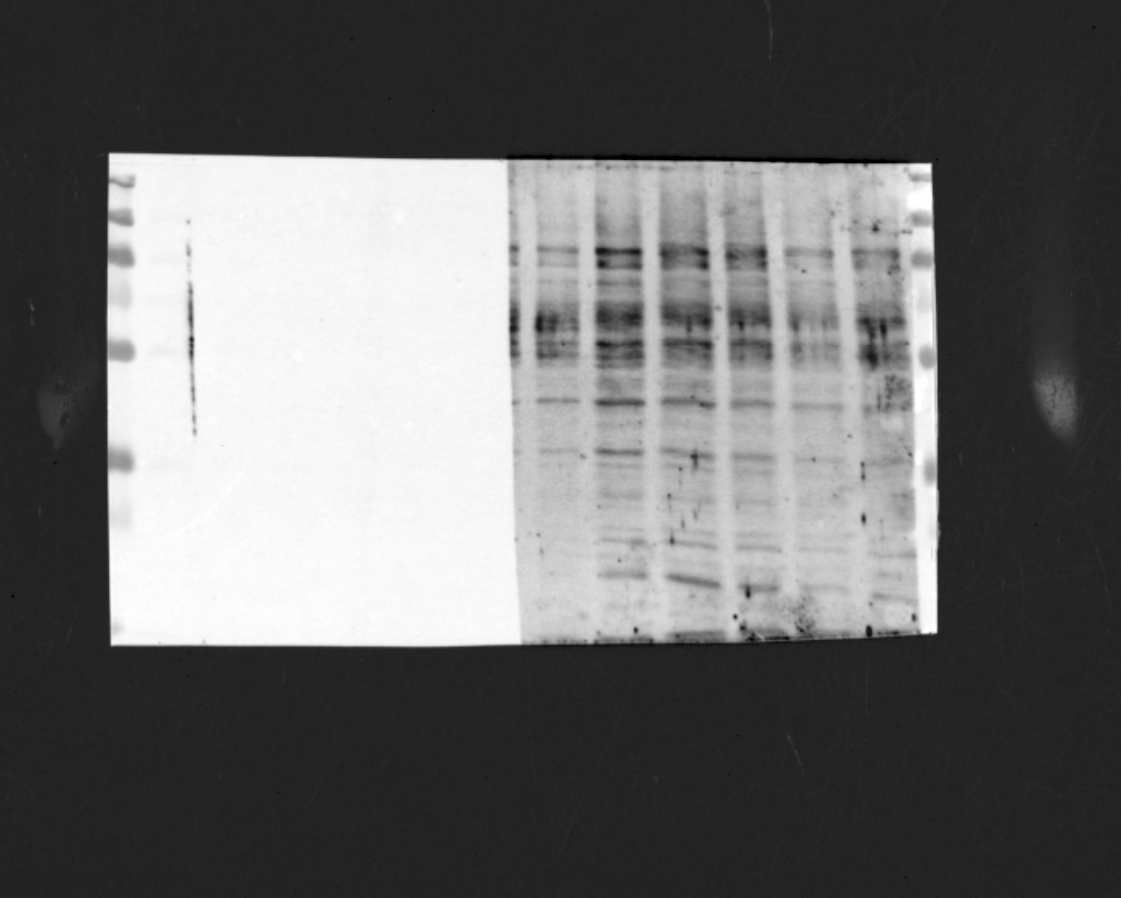

Supplement: Supplementary file 7 [file DataSheet5.zip › BxPC3_4HNE/TIF/4HNE 1. (kiértékelt_4hne_0130).tif]

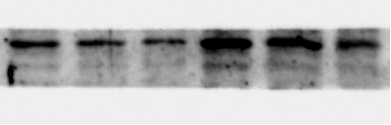

Supplement: Supplementary file 7 [file DataSheet5.zip › BxPC3_4HNE/TIF/4HNE 1. Actin (01.30.aktin_3_4_5).tif]
