# Supplementary material for: Anticarcinogenic effects of ursodeoxycholic acid in pancreatic adenocarcinoma cell models
Source: Front Cell Dev Biol. 2024 Dec 11;12:1487685. doi: 10.3389/fcell.2024.1487685 (PMC11668698; doi:10.3389/fcell.2024.1487685)

**Supplementary Figure 1.**

The Capan-2 cells were treated with UDCA for 48 h, then the mRNA expressions of NRF2 target genes, NQO1, GCLC, CAT, HMOX1 and TXN and were determined by RT-qPCR (n=2).

**Supplementary Method 1.**

## **Quantitative RT-PCR**

Total RNA was isolated from cells with TRIzol reagent (Thermo Scientific) and treated with DNase enzyme (cat # AM2222, Thermo Fisher Scientific). The cDNA was synthesized by a High Capacity cDNA Reverse Transcription Kit (Applied Biosystems, Waltham, MA, USA). The qPCR reactions were carried out in a 10 μL reaction volume containing 0.5 μM of each primer and qPCRBIO SyGreen Lo-ROX Supermix (PCR Biosystems Ltd., London, UK). The reaction was performed on the Light-Cycler 480 Detection System (Roche, Basel, Switzerland). Primer informations are listed in **Supplementary Table 1.**

**Supplementary Table 1.**

| **Gene Symbol** | **Forward primer (5’-3’)** | **Reverse primer (5’-3’)** |
| --- | --- | --- |
| NQO1 | ccaagcagcctctttgaccta | aagtcagggaagcctggaaag |
| GCLC | ccacaaattggcagacaatga | acattccctgcaagacagcat |
| CAT | tgaaaatttgtgcatccttca | attctggagaagtgcggaga |
| HMOX1 | gccaggtgctcaaaaagattg | gctcctgcaactcctcaaaga |
| TXN | ccatttccatcggtccttaca | tgttggcatgcatttgacttc |
| 36B4 | CCATTGAAATCCTGAGTGATGTG | GTCGAACACCTGCTGGATGAC |

**Supplementary Figure 2.**

**The effects of UDCA can be elicited in PancTu-1 cells**

**A-C** PancTu-1 cells (150.000 cells/well) were seeded into 6-well plates and were treated with 0.3 µM UDCA for 48 hours. Cellular lysates were analyzed by SDS-PAGE followed by Western blot. The blots were probed with the antibodies indicated and blots were subjected to densitometry. * and ** indicates statistically significant difference between vehicle-treated and UDCA-treated groups at p<0.05 or p<0.01, respectively. Abbreviations: DMSO – dimethyl sulfoxide, iNOS – inducible nitric oxide synthase, UDCA – ursodeoxycholic acid


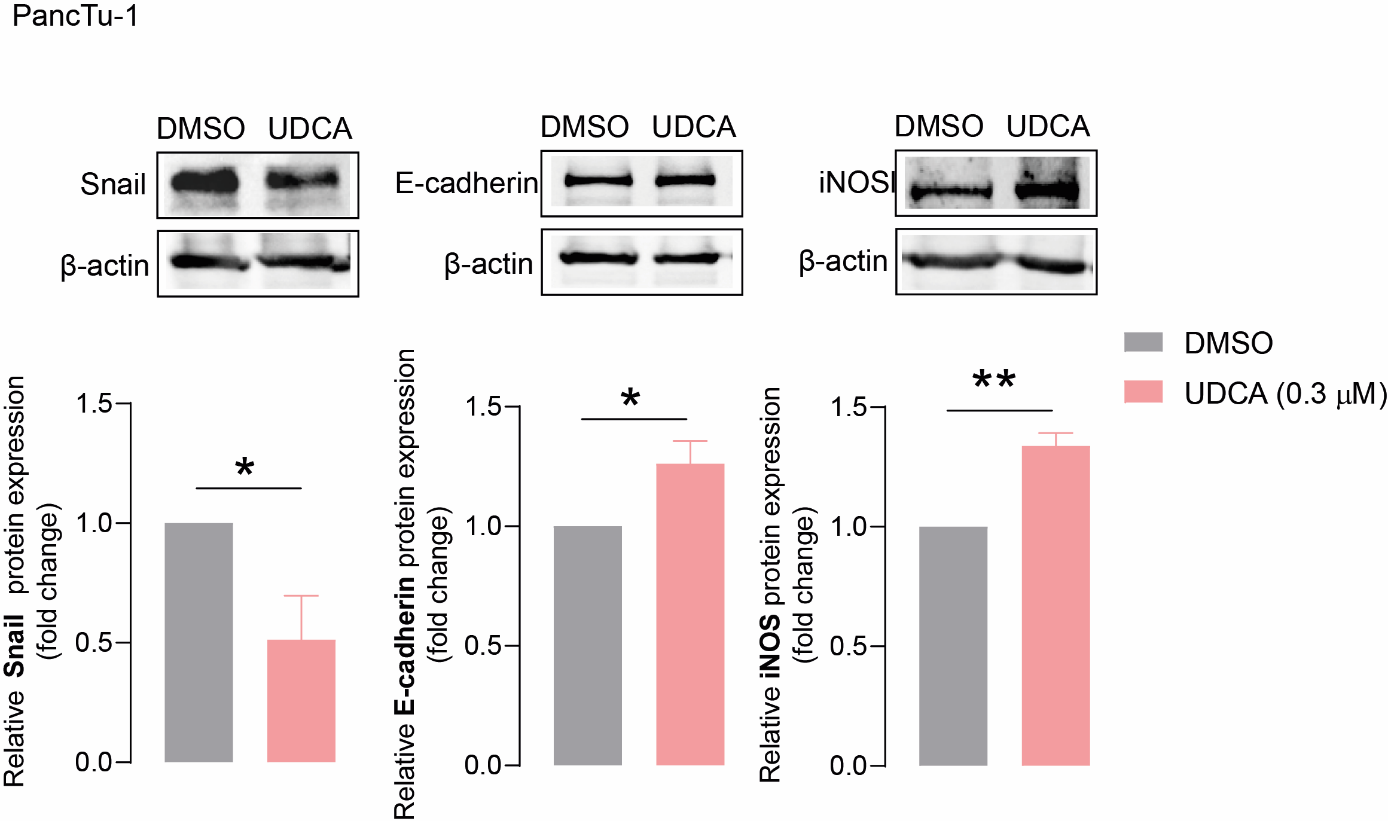

Supplement: Supplementary file 2 [file Supplementaryfile1.docx]
