# Supplementary material for: Anticarcinogenic effects of ursodeoxycholic acid in pancreatic adenocarcinoma cell models
Source: Front Cell Dev Biol. 2024 Dec 11;12:1487685. doi: 10.3389/fcell.2024.1487685 (PMC11668698; doi:10.3389/fcell.2024.1487685)
Supplement: Supplementary file 3 [file DataSheet4.zip › Western blots_1.pptx]

## Slide 1
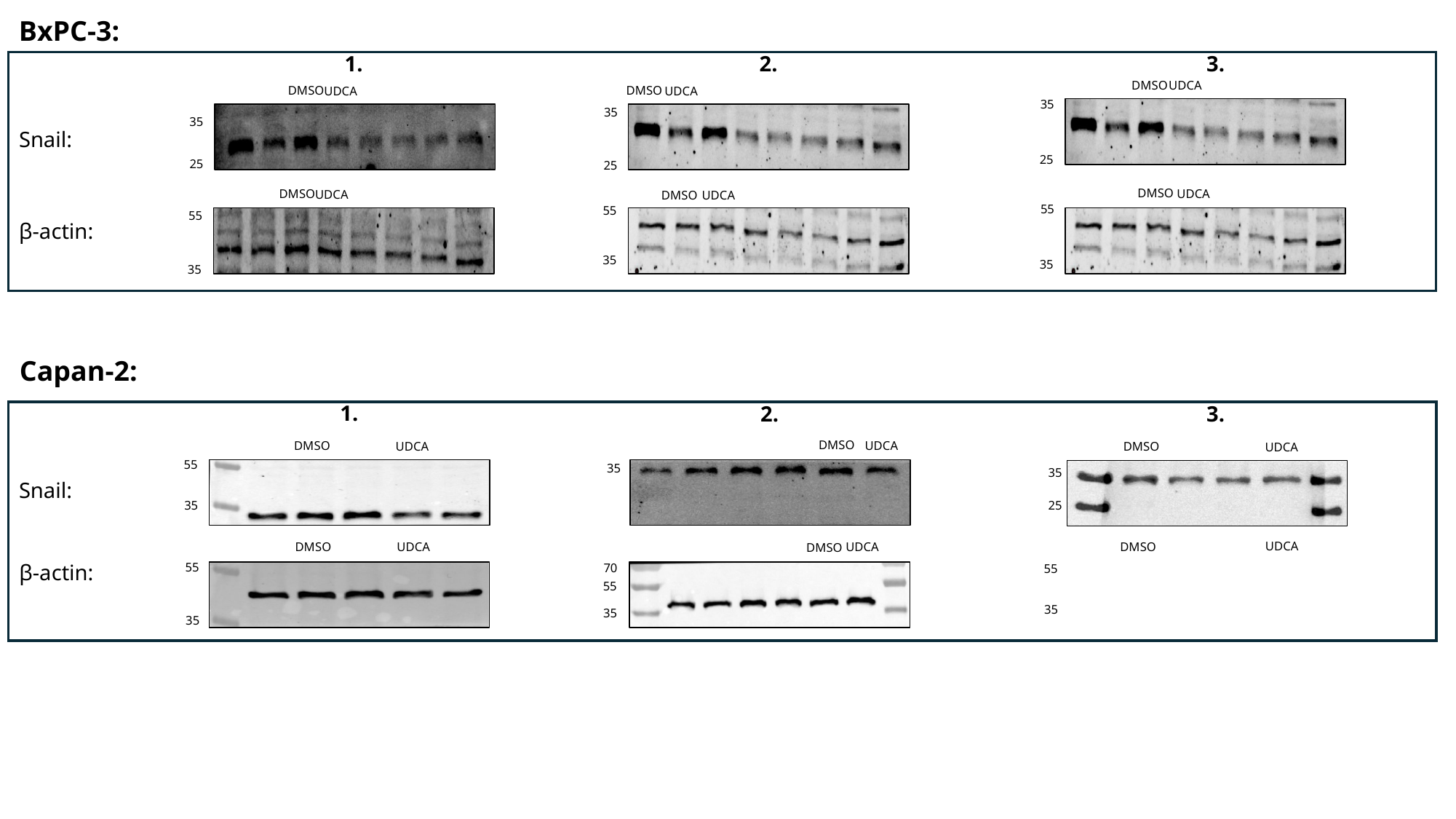

BxPC-3:
3.
1.
DMSO
UDCA
2.
Snail:
β-actin:
DMSO
UDCA
DMSO
UDCA
DMSO
UDCA
DMSO
UDCA
UDCA
DMSO
35
55
35
25
55
35
55
35
35
25
35
25
Capan-2:
1.
3.
2.
Snail:
β-actin:
DMSO
UDCA
55
35
UDCA
DMSO
55
35
35
DMSO
UDCA
UDCA
DMSO
70
55
35
DMSO
UDCA
35
25
UDCA
DMSO
55
35

## Slide 2
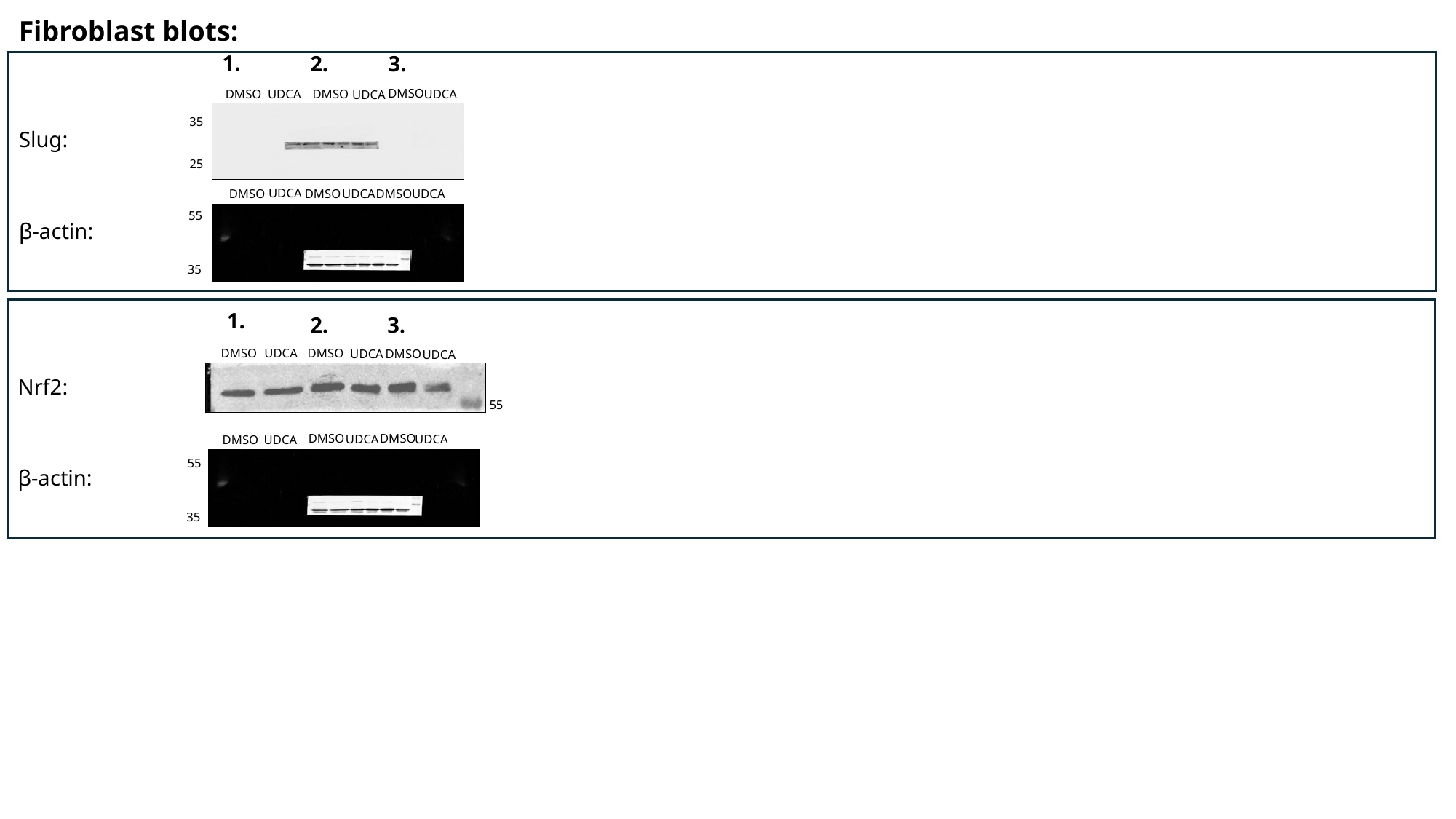

Fibroblast blots:
1.
DMSO
UDCA
2.
3.
Slug:
β-actin:
DMSO
UDCA
DMSO
UDCA
UDCA
DMSO
UDCA
UDCA
DMSO
DMSO
35
55
35
25
1.
DMSO
UDCA
3.
2.
Nrf2:
β-actin:
DMSO
UDCA
DMSO
UDCA
DMSO
DMSO
UDCA
UDCA
DMSO
UDCA
55
35
55

## Slide 3
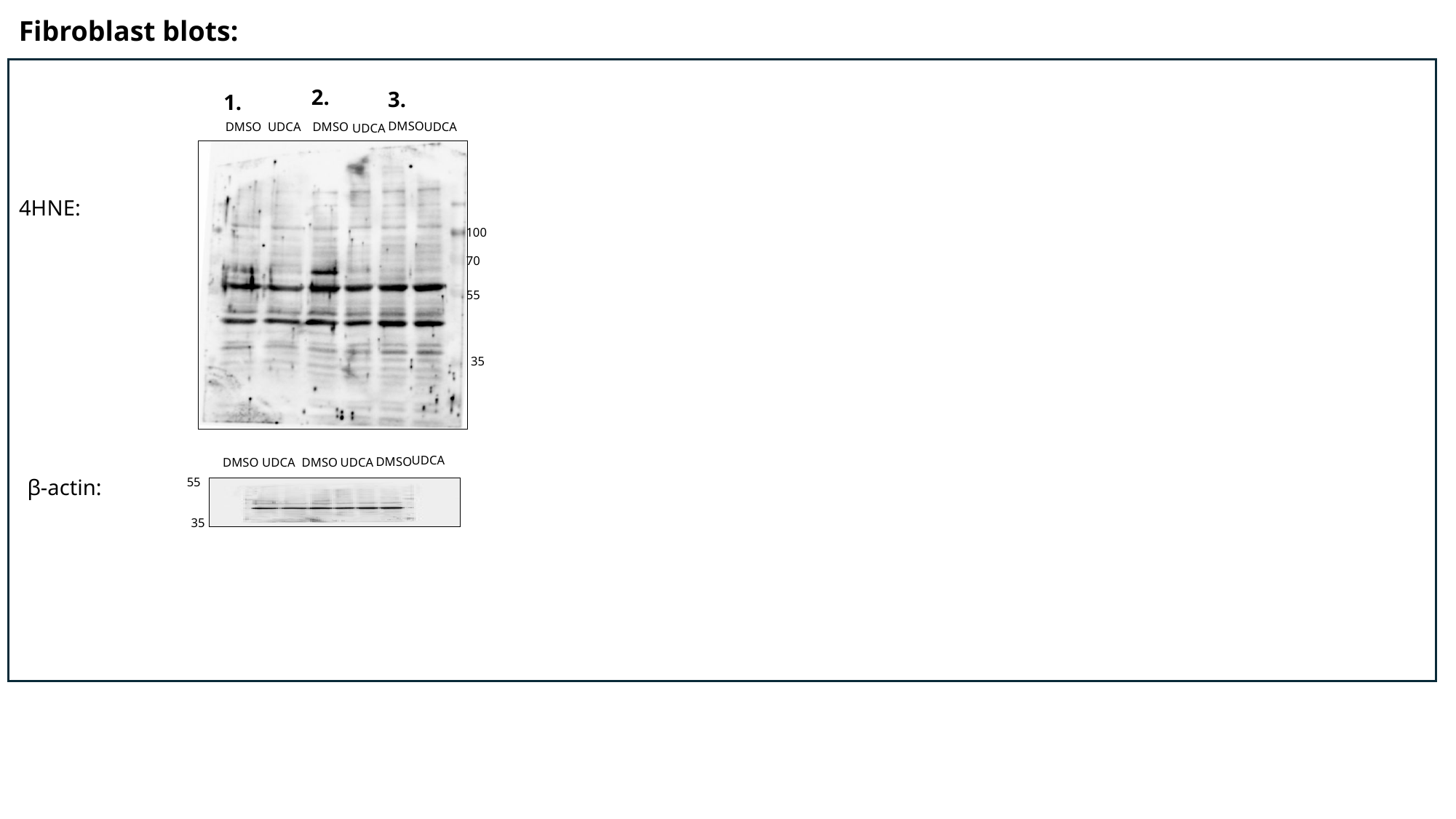

Fibroblast blots:
2.
3.
1.
DMSO
UDCA
4HNE:
β-actin:
DMSO
UDCA
DMSO
UDCA
UDCA
DMSO
DMSO
UDCA
DMSO
UDCA
35
55
35
55
100
70
