# Supplementary material for: Anticarcinogenic effects of ursodeoxycholic acid in pancreatic adenocarcinoma cell models
Source: Front Cell Dev Biol. 2024 Dec 11;12:1487685. doi: 10.3389/fcell.2024.1487685 (PMC11668698; doi:10.3389/fcell.2024.1487685)
Supplement: Supplementary file 4 [file DataSheet1.zip › Western blots_cadh.pptx]

## Slide 1
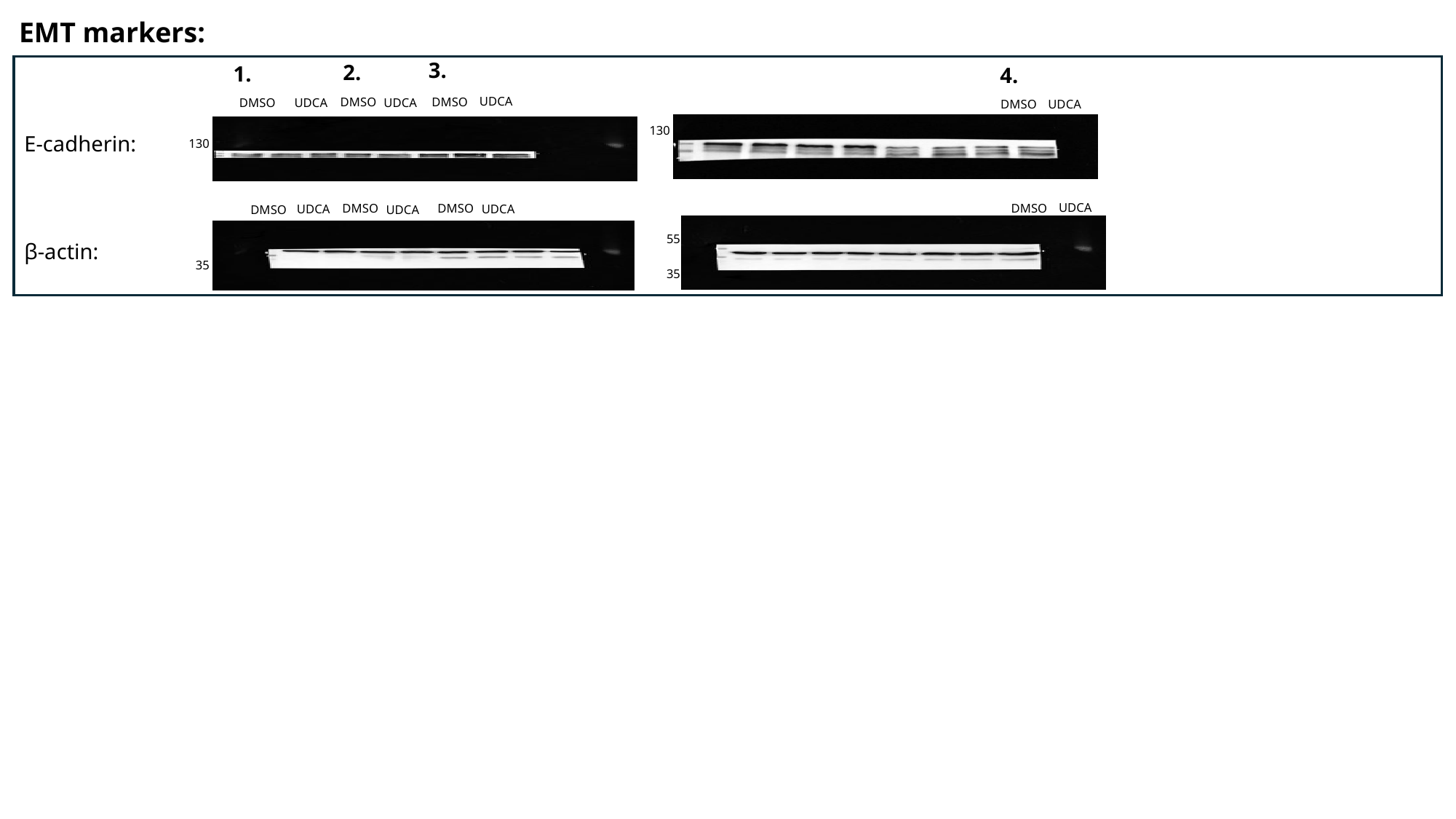

EMT markers:
3.
2.
1.
E-cadherin:
β-actin:
DMSO
UDCA
130
UDCA
DMSO
35
130
DMSO
UDCA
55
UDCA
DMSO
4.
DMSO
UDCA
UDCA
DMSO
DMSO
DMSO
UDCA
UDCA
35
