# Supplementary material for: Anticarcinogenic effects of ursodeoxycholic acid in pancreatic adenocarcinoma cell models
Source: Front Cell Dev Biol. 2024 Dec 11;12:1487685. doi: 10.3389/fcell.2024.1487685 (PMC11668698; doi:10.3389/fcell.2024.1487685)
Supplement: Supplementary file 5 [file DataSheet6.zip › Western blots_3.pptx]

## Slide 1
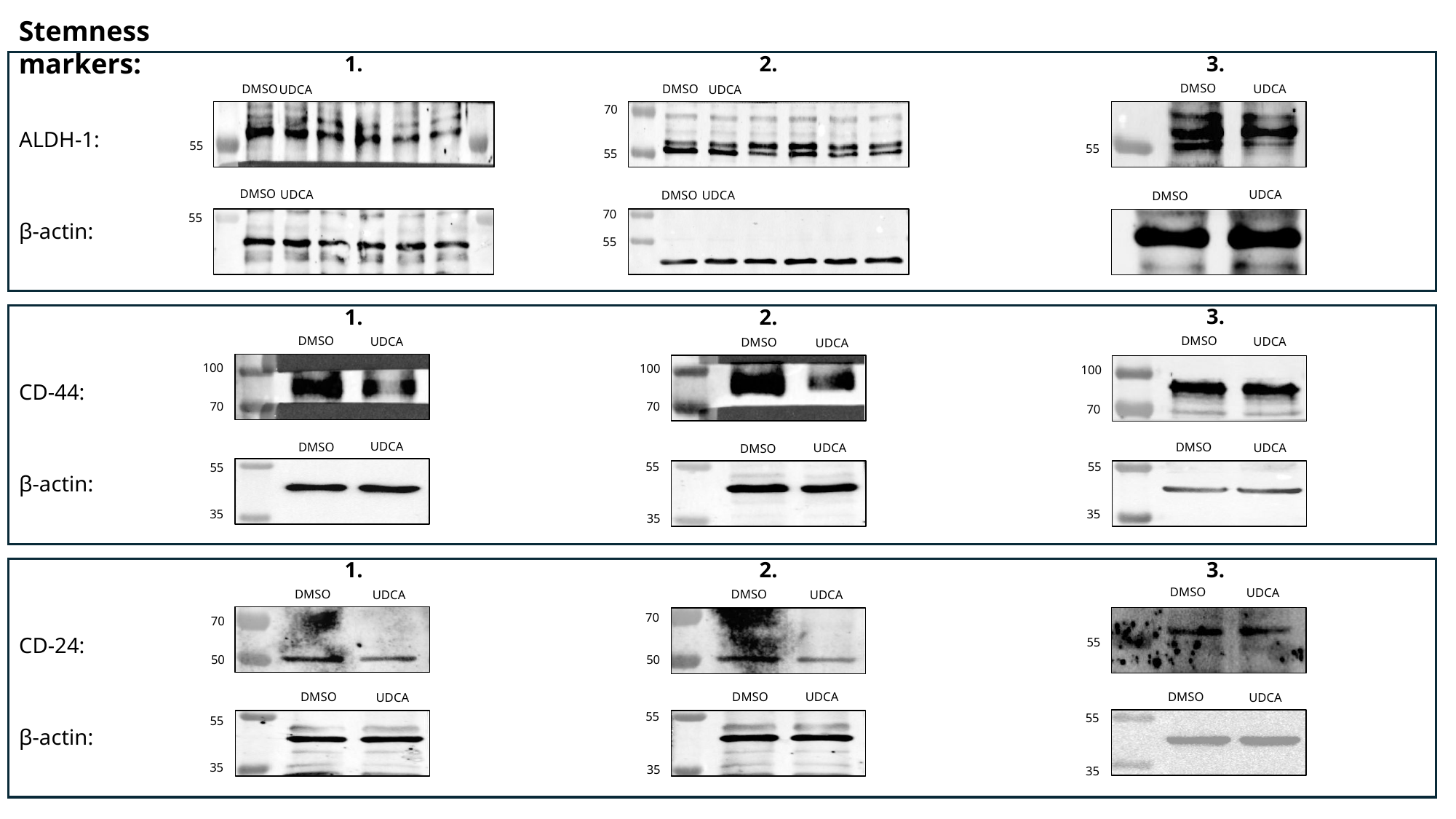

Stemness markers:
3.
1.
DMSO
UDCA
2.
ALDH-1:
β-actin:
DMSO
UDCA
DMSO
UDCA
DMSO
UDCA
UDCA
UDCA
DMSO
DMSO
55
55
70
55
70
55
55
3.
1.
DMSO
UDCA
2.
CD-44:
β-actin:
DMSO
UDCA
DMSO
UDCA
UDCA
DMSO
DMSO
UDCA
UDCA
DMSO
100
55
100
70
55
35
70
55
100
70
35
35
3.
1.
DMSO
UDCA
2.
CD-24:
β-actin:
DMSO
UDCA
DMSO
UDCA
UDCA
DMSO
DMSO
DMSO
UDCA
UDCA
70
55
70
50
55
35
55
55
50
35
35

## Slide 2
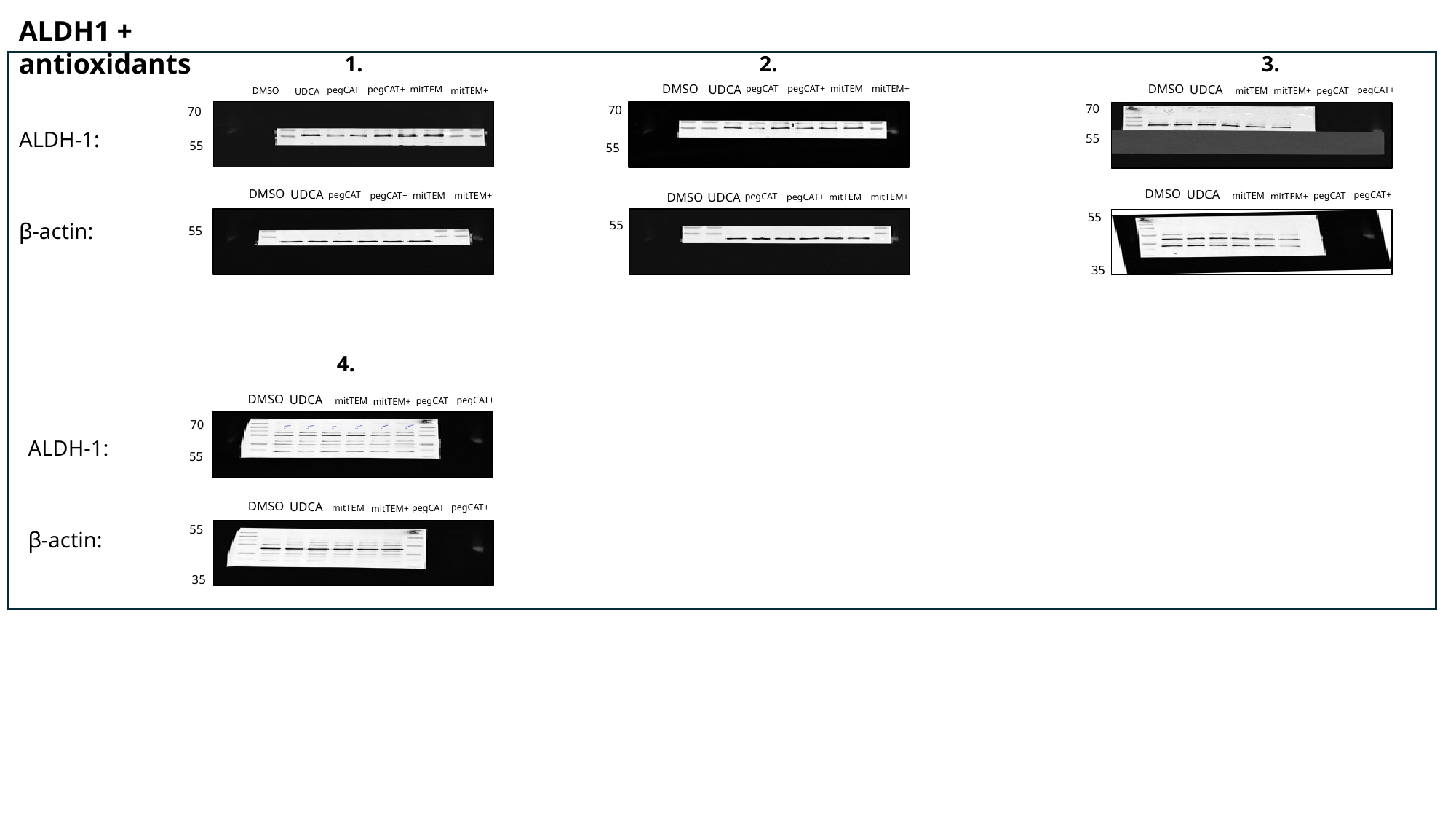

ALDH1 + antioxidants
3.
1.
DMSO
UDCA
2.
ALDH-1:
β-actin:
DMSO
UDCA
DMSO
UDCA
UDCA
DMSO
55
55
70
55
DMSO
UDCA
pegCAT+
pegCAT
mitTEM
mitTEM+
pegCAT
pegCAT+
mitTEM
mitTEM+
pegCAT+
mitTEM
pegCAT
mitTEM+
70
70
55
DMSO
UDCA
pegCAT+
pegCAT
mitTEM
mitTEM+
pegCAT
pegCAT+
mitTEM
mitTEM+
pegCAT
pegCAT+
mitTEM
mitTEM+
55
55
35
4.
DMSO
UDCA
pegCAT+
pegCAT
mitTEM
mitTEM+
70
ALDH-1:
55
DMSO
UDCA
pegCAT+
pegCAT
mitTEM
mitTEM+
55
β-actin:
35
