# Supplementary material for: Anticarcinogenic effects of ursodeoxycholic acid in pancreatic adenocarcinoma cell models
Source: Front Cell Dev Biol. 2024 Dec 11;12:1487685. doi: 10.3389/fcell.2024.1487685 (PMC11668698; doi:10.3389/fcell.2024.1487685)
Supplement: Supplementary file 6 [file DataSheet2.zip › Western blots_vim.pptx]

## Slide 1
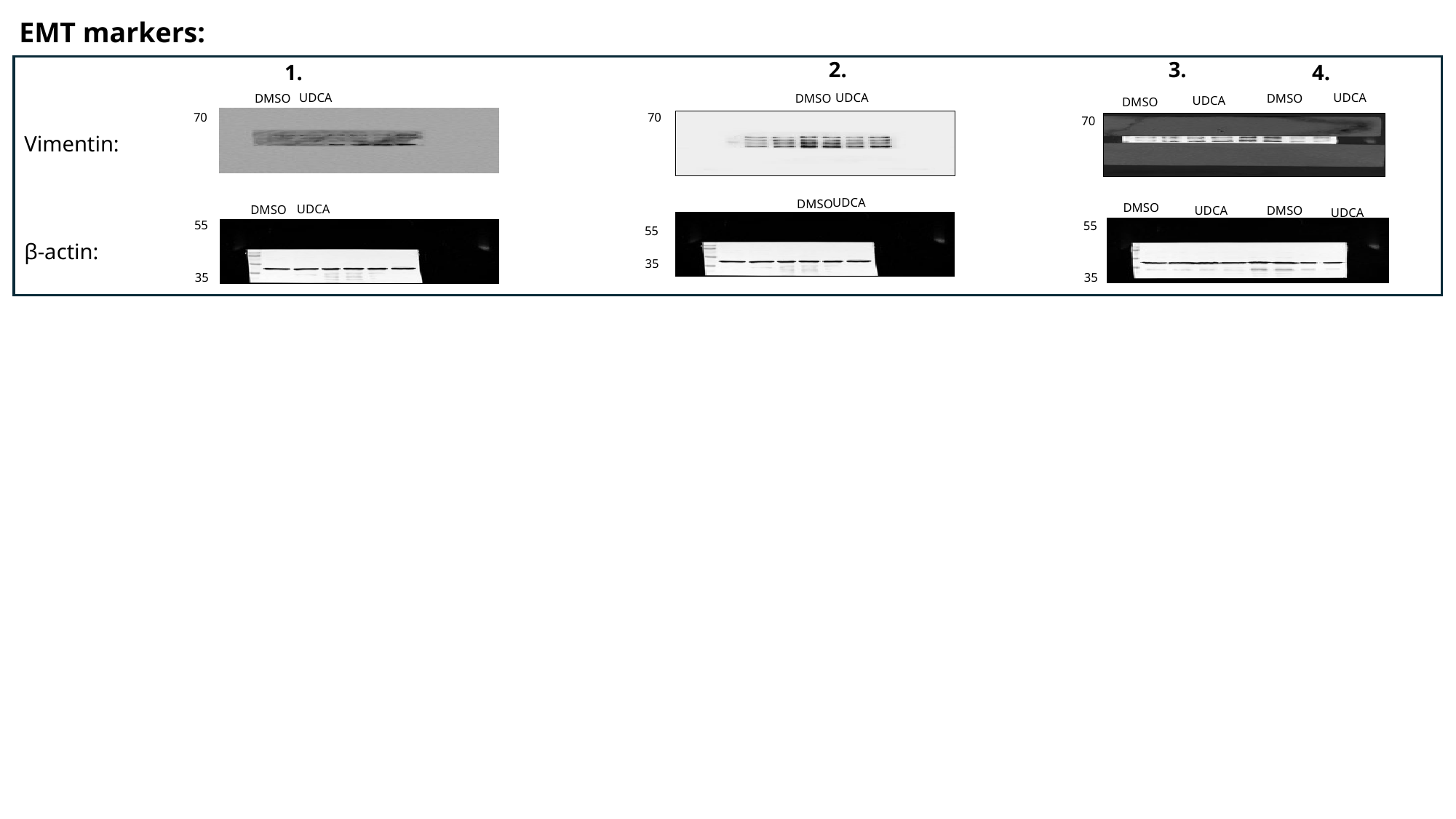

EMT markers:
2.
3.
1.
Vimentin:
β-actin:
UDCA
DMSO
70
UDCA
DMSO
55
35
70
UDCA
DMSO
UDCA
DMSO
55
UDCA
DMSO
55
35
DMSO
UDCA
4.
UDCA
DMSO
70
DMSO
UDCA
35
