# Supplementary material for: Anticarcinogenic effects of ursodeoxycholic acid in pancreatic adenocarcinoma cell models
Source: Front Cell Dev Biol. 2024 Dec 11;12:1487685. doi: 10.3389/fcell.2024.1487685 (PMC11668698; doi:10.3389/fcell.2024.1487685)
Supplement: Supplementary file 7 [file DataSheet5.zip › Western blots_2.pptx]

## Slide 1
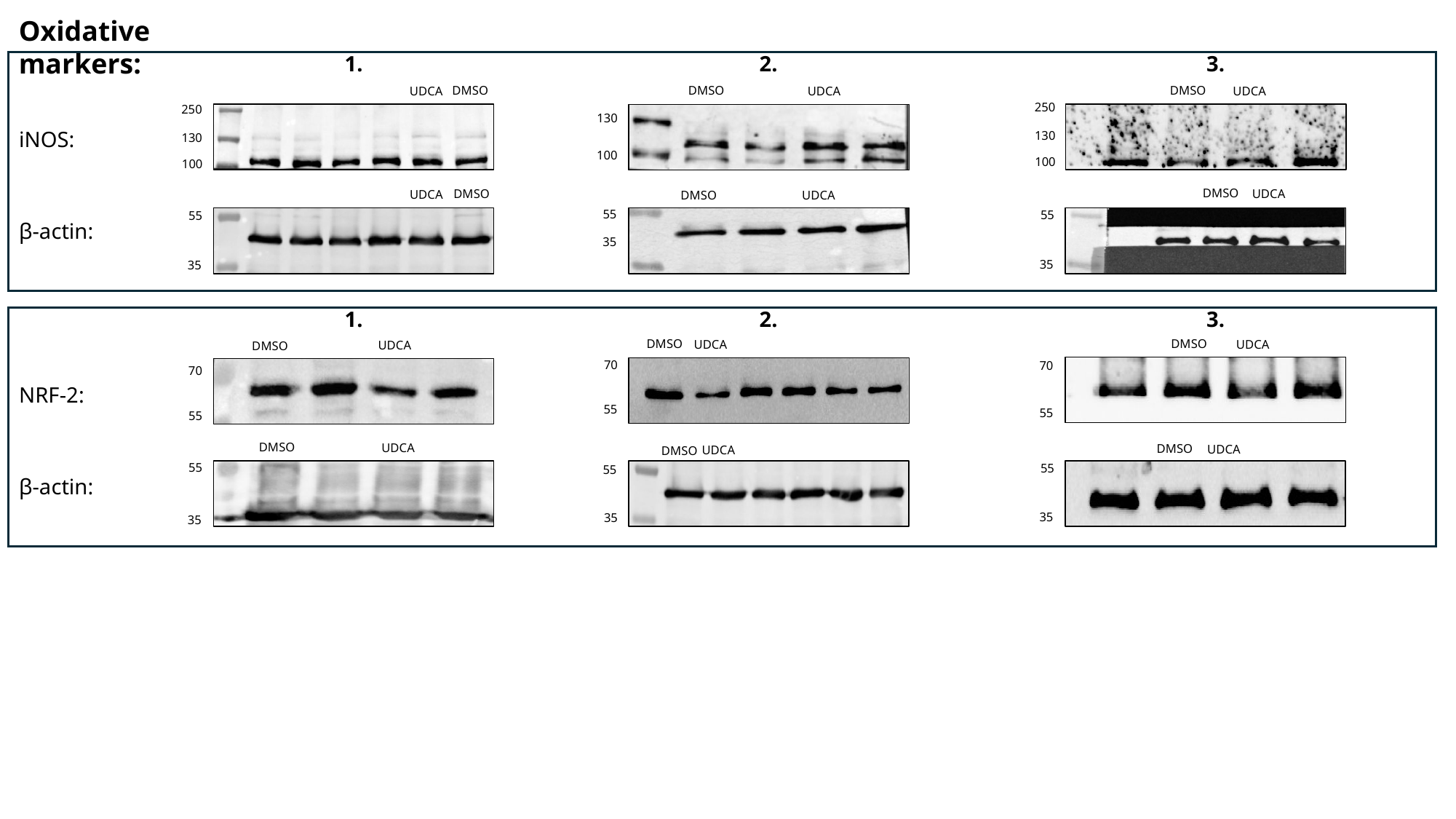

Oxidative markers:
3.
1.
DMSO
UDCA
2.
iNOS:
β-actin:
DMSO
UDCA
DMSO
UDCA
DMSO
UDCA
DMSO
UDCA
UDCA
DMSO
250
55
130
100
55
35
55
130
100
35
250
130
100
35
3.
1.
UDCA
DMSO
2.
NRF-2:
β-actin:
DMSO
UDCA
DMSO
UDCA
DMSO
UDCA
DMSO
UDCA
UDCA
DMSO
70
55
70
55
55
35
55
55
70
55
35
35

## Slide 2
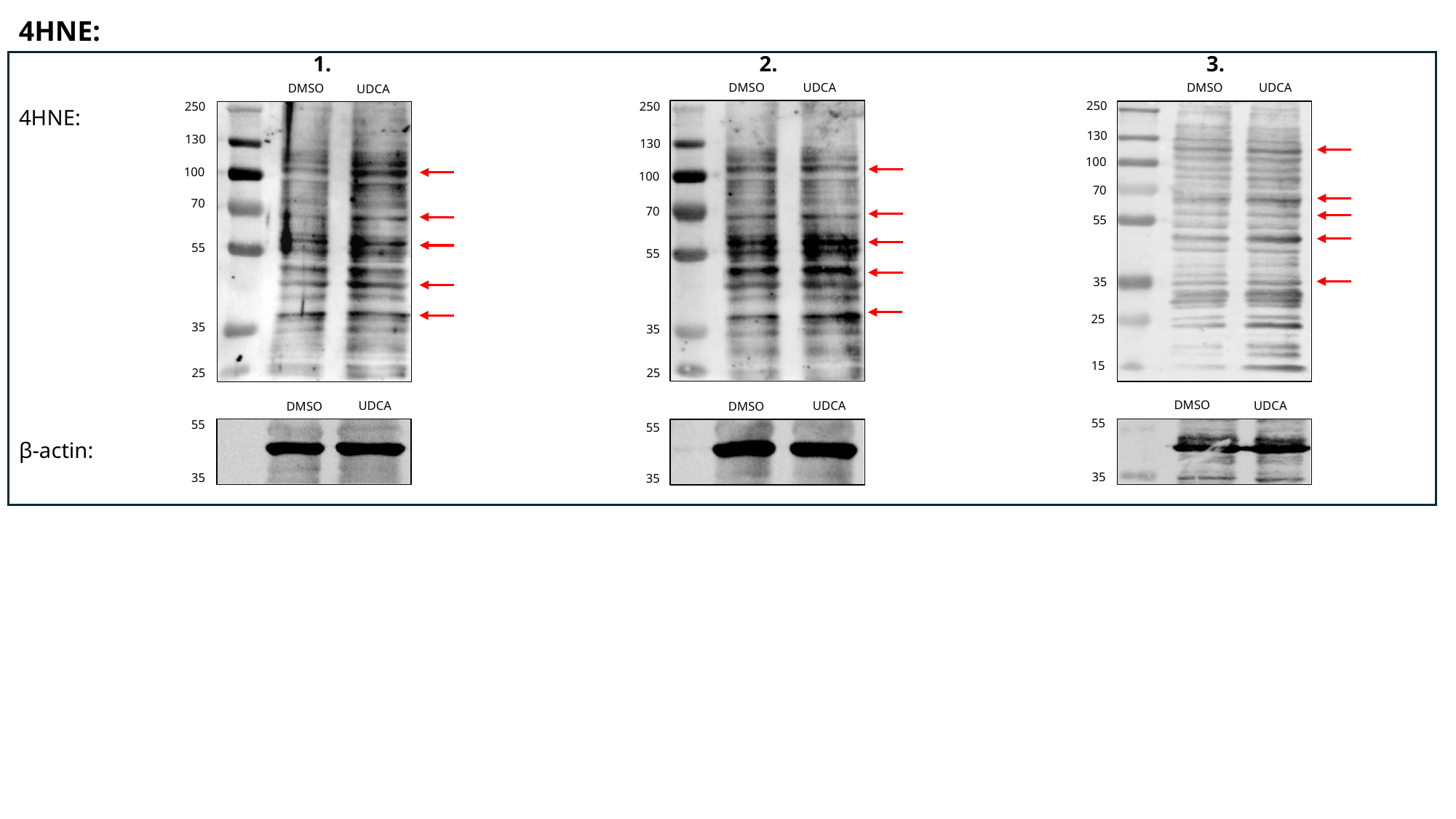

4HNE:
3.
1.
DMSO
UDCA
2.
4HNE:
β-actin:
DMSO
UDCA
DMSO
UDCA
DMSO
UDCA
UDCA
UDCA
DMSO
DMSO
250
55
55
35
55
130
100
35
35
70
55
35
25
250
130
100
70
55
35
25
250
130
100
70
55
35
25
15

## Slide 3
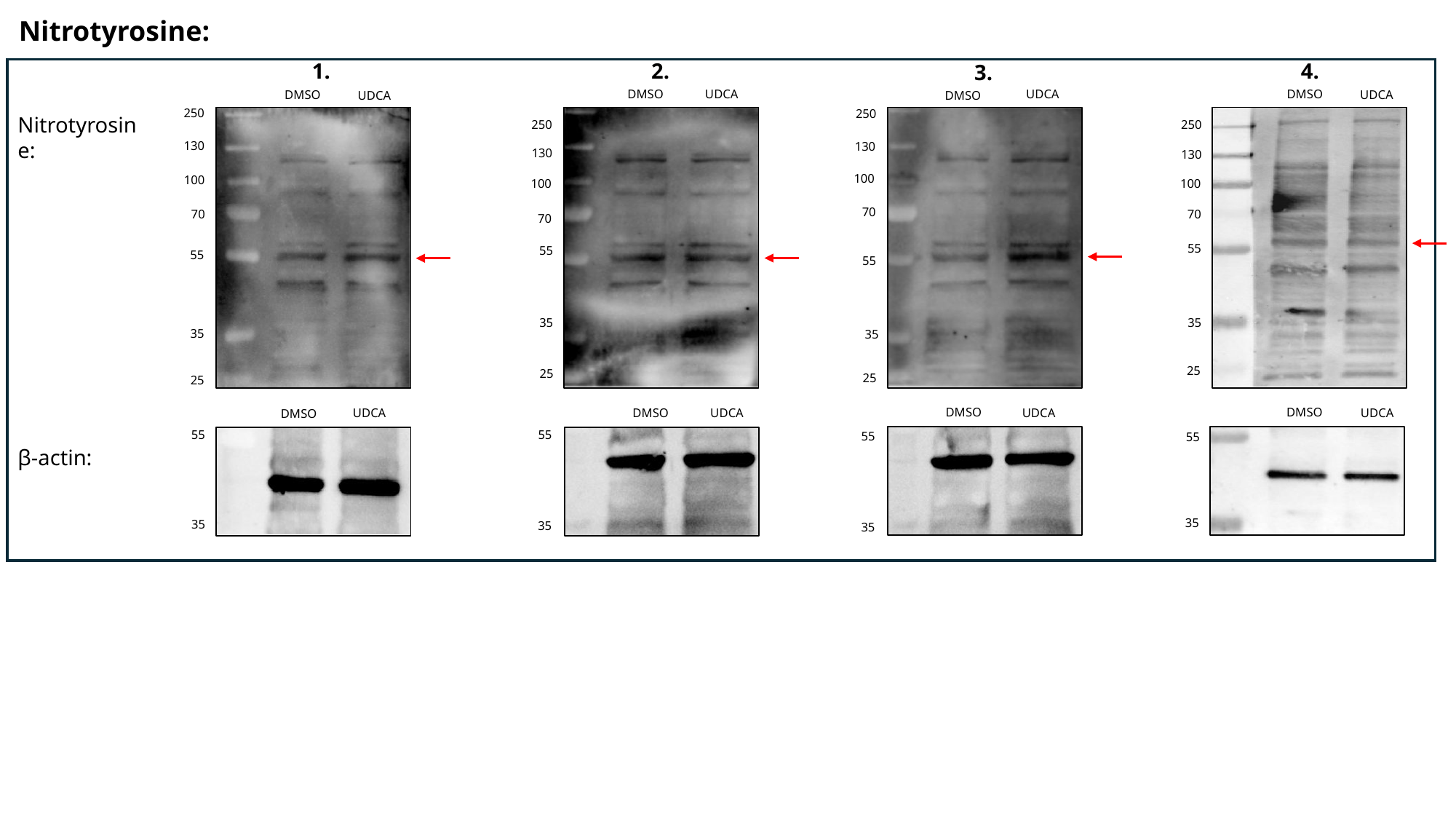

Nitrotyrosine:
4.
1.
DMSO
UDCA
2.
Nitrotyrosine:
β-actin:
UDCA
DMSO
DMSO
UDCA
DMSO
UDCA
UDCA
UDCA
DMSO
DMSO
250
55
55
35
55
130
100
35
35
70
55
35
25
250
130
100
70
55
35
25
250
130
100
70
55
35
25
3.
UDCA
DMSO
250
130
100
70
55
35
25
55
35
DMSO
UDCA

## Slide 4
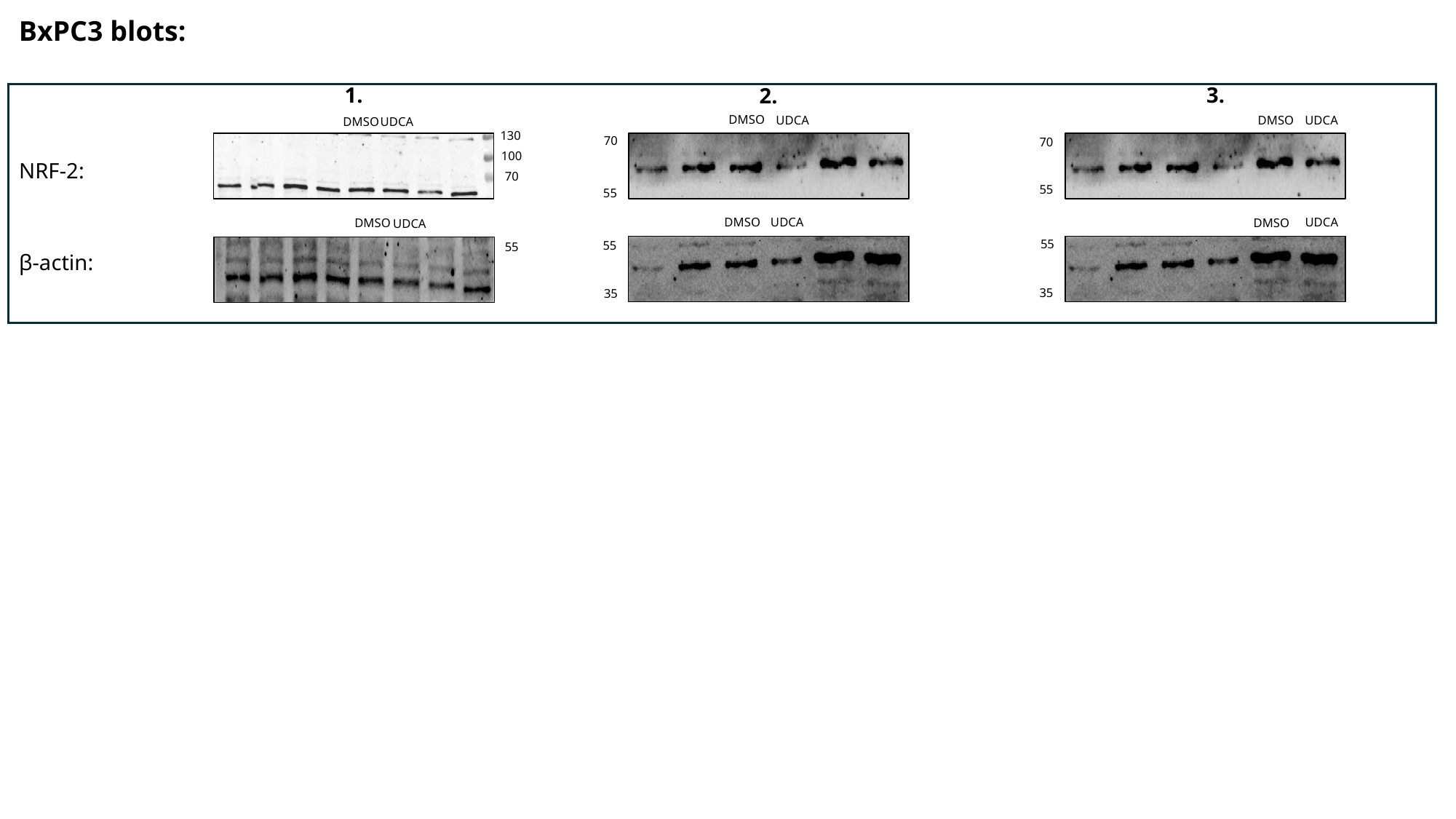

BxPC3 blots:
3.
1.
UDCA
DMSO
2.
NRF-2:
β-actin:
DMSO
UDCA
DMSO
UDCA
UDCA
UDCA
DMSO
DMSO
DMSO
UDCA
70
55
55
35
55
55
70
100
35
130
70
55

## Slide 5
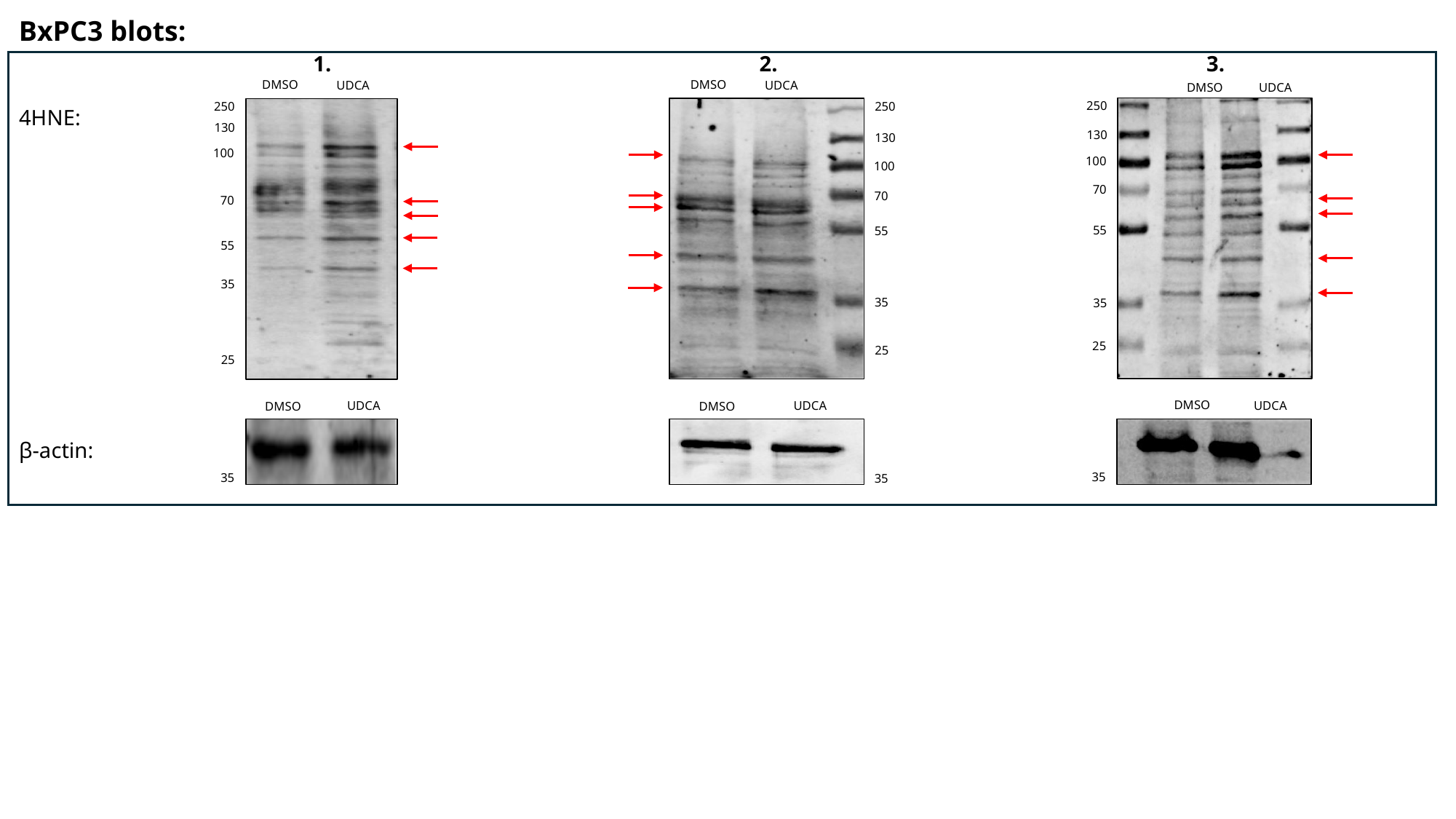

BxPC3 blots:
3.
1.
DMSO
UDCA
2.
4HNE:
β-actin:
DMSO
UDCA
DMSO
UDCA
DMSO
UDCA
UDCA
UDCA
DMSO
DMSO
250
35
130
100
35
35
70
55
35
25
250
130
100
70
55
35
25
250
130
100
70
55
35
25
