# Supplementary figures and images for: Anticarcinogenic effects of ursodeoxycholic acid in pancreatic adenocarcinoma cell models (part 2 of 2)
Source: Front Cell Dev Biol. 2024 Dec 11;12:1487685. doi: 10.3389/fcell.2024.1487685 (PMC11668698; doi:10.3389/fcell.2024.1487685)

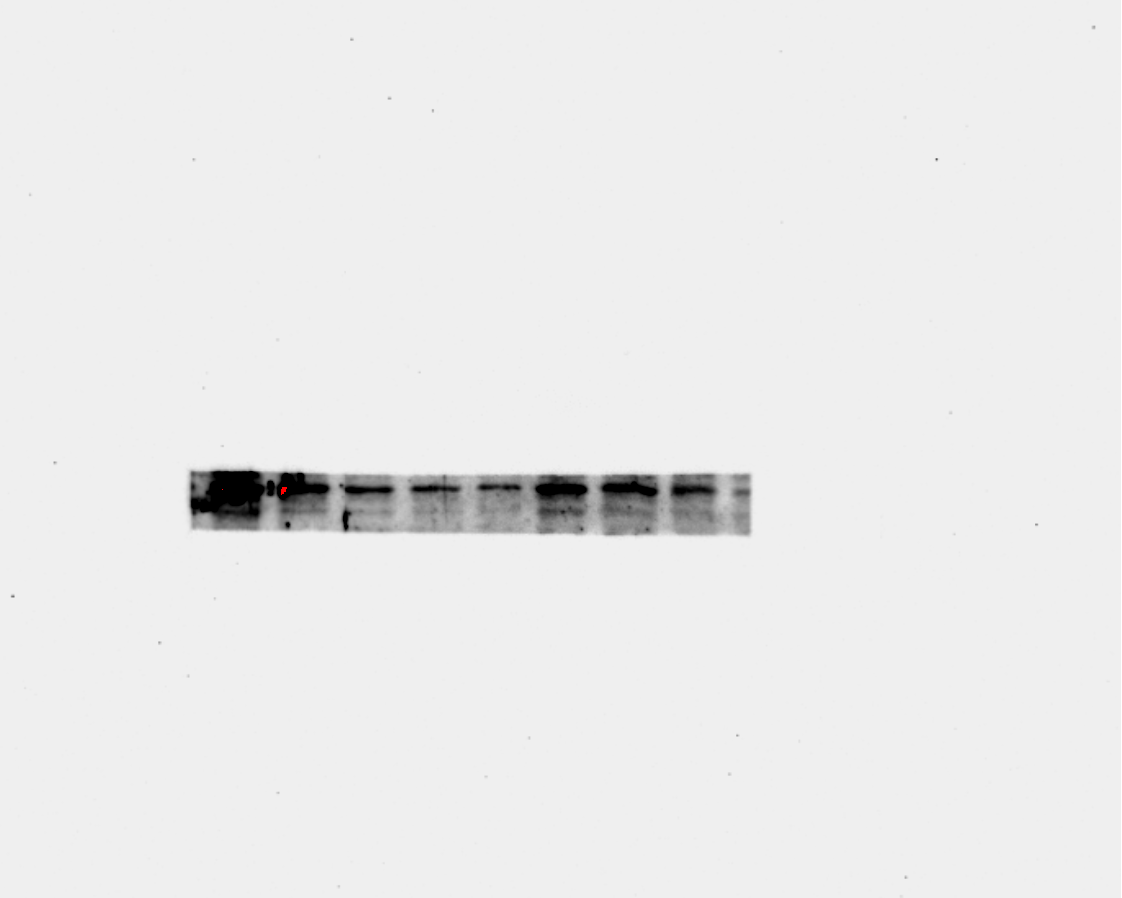

Supplement: Supplementary file 7 [file DataSheet5.zip › BxPC3_4HNE/TIF/4HNE 1. Actin (kiértékelt_aktin_4hne_4_0130).tif]

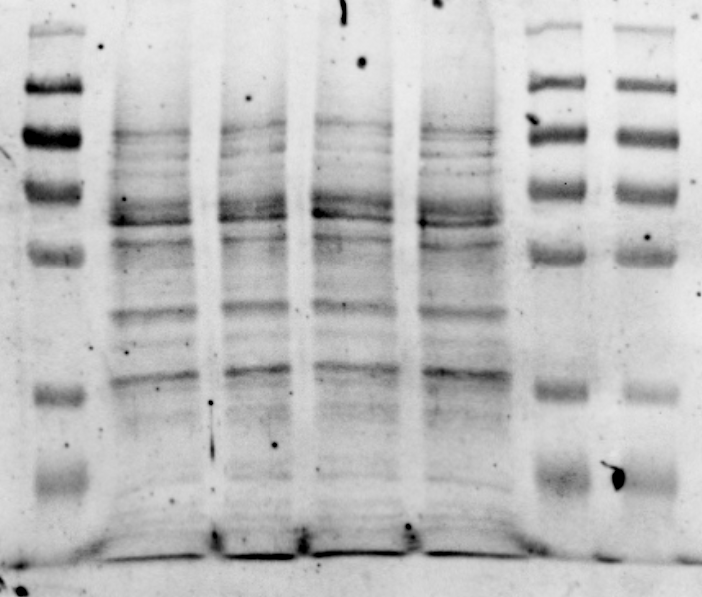

Supplement: Supplementary file 7 [file DataSheet5.zip › BxPC3_4HNE/TIF/4HNE 2. (01.23.4hne).tif]

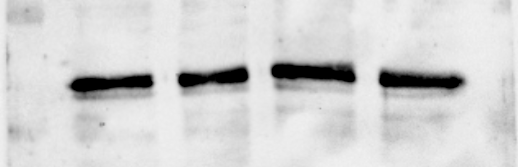

Supplement: Supplementary file 7 [file DataSheet5.zip › BxPC3_4HNE/TIF/4HNE 2. Actin (kiértékelt_aktin_4hne_0123).tif]

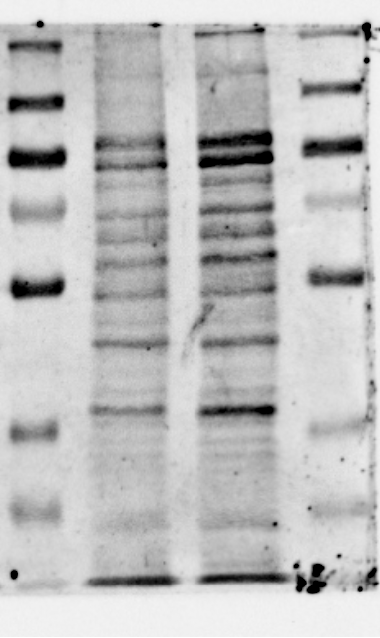

Supplement: Supplementary file 7 [file DataSheet5.zip › BxPC3_4HNE/TIF/4HNE 3. (kiértékelt_4hne_0313(7)).tif]

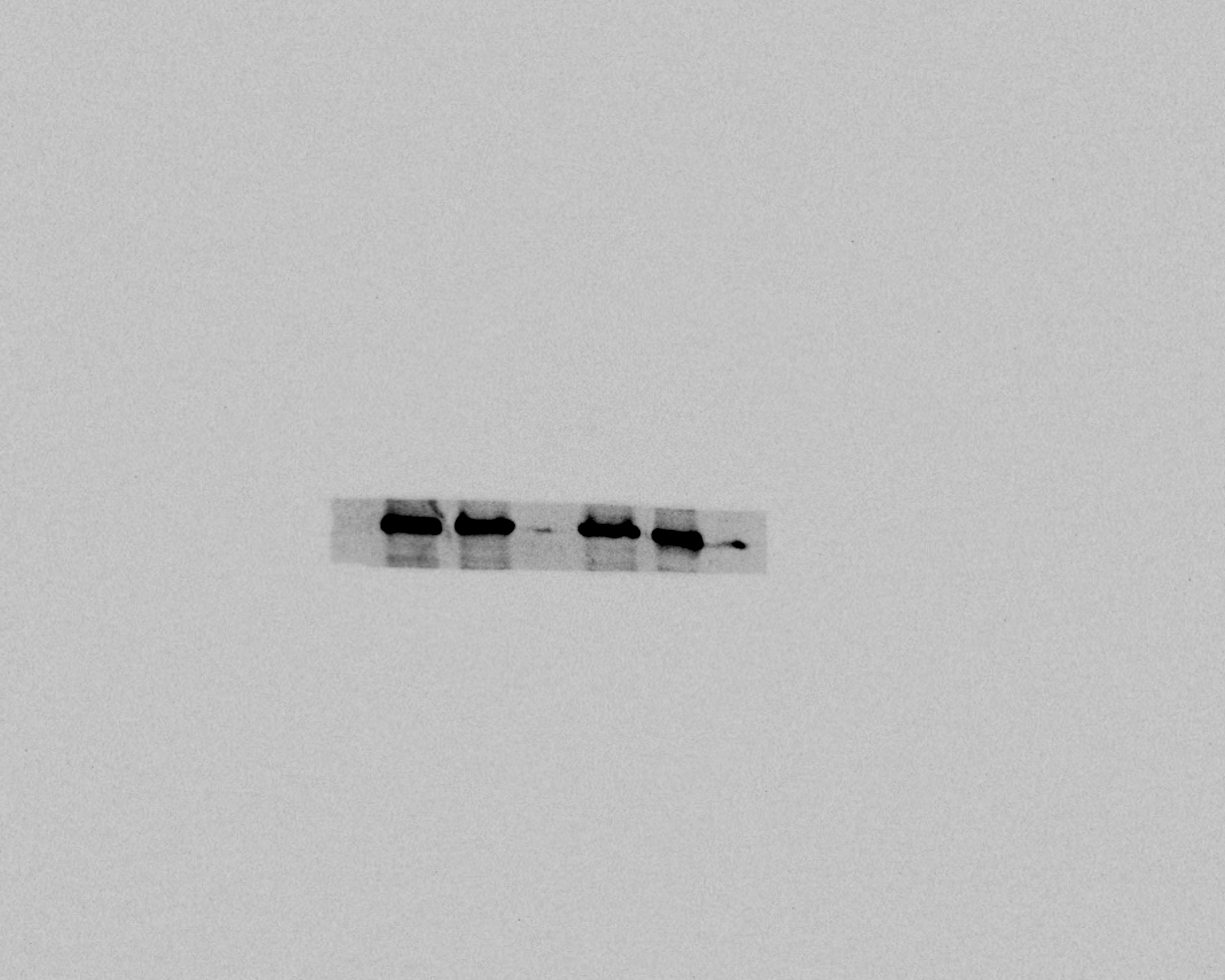

Supplement: Supplementary file 7 [file DataSheet5.zip › BxPC3_4HNE/TIF/4HNE 3. Actin (kiértékelt_aktin_4hne_0313).tif]

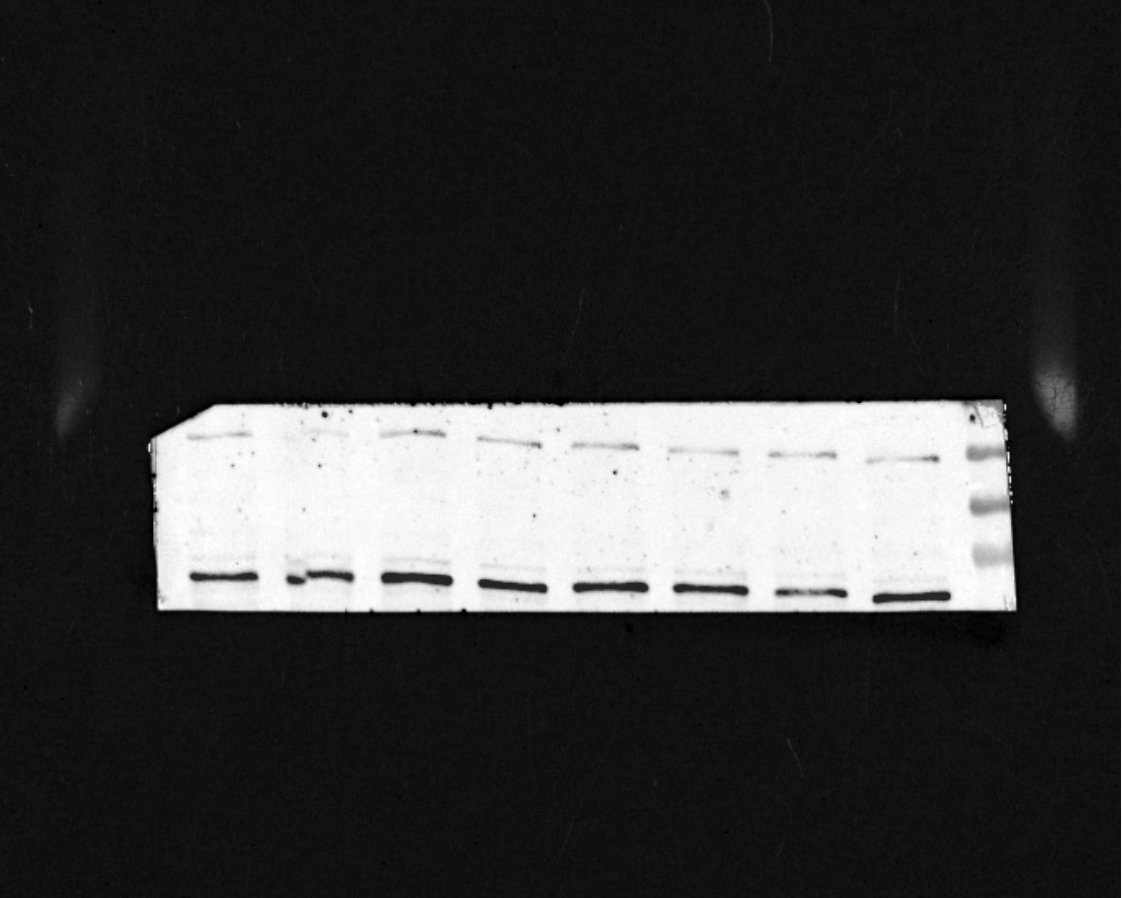

Supplement: Supplementary file 7 [file DataSheet5.zip › BxPC3_NRF2/TIF/NRF2 1. (merged_nrf2_1207).tif]

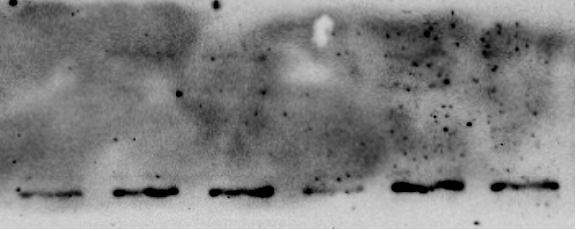

Supplement: Supplementary file 7 [file DataSheet5.zip › BxPC3_NRF2/TIF/NRF2 2. (kiértékelt_nrf2_0126).tif]

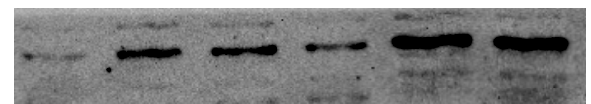

Supplement: Supplementary file 7 [file DataSheet5.zip › BxPC3_NRF2/TIF/NRF2 2. Actin (kiértékelt_aktin_nrf2_56_0127).tif]
